# Diversity Synthesis Using Glutarimides as Rhodium Carbene Precursors in Enantioselective C–H Functionalization and Cyclopropanation

**DOI:** 10.1021/jacs.5c00568

**Published:** 2025-03-18

**Authors:** William F. Tracy, Jack C. Sharland, Duc Ly, Geraint H. M. Davies, Djamaladdin G. Musaev, Hua Fang, Jesus Moreno, Emily C. Cherney, Huw M. L. Davies

**Affiliations:** † Department of Chemistry, Emory University, Atlanta, Georgia 30322, United States; ‡ Discovery and Development Sciences, Bristol Myers Squibb, Cambridge, Massachusetts 02143, United States; § Discovery and Development Sciences, Bristol Myers Squibb, San Diego, California 92121, United States; ∥ Discovery and Development Sciences, Bristol Myers Squibb, Princeton, New Jersey 08543, United States

## Abstract

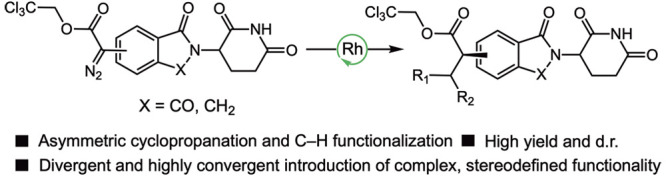

Cereblon E3 ligase modulatory drugs (CELMoDs) can be used to target proteins and mark them for proteasomal degradation by recruiting them to cereblon (CRBN), the substrate receptor of the CRL4^CRBN^ E3 ubiquitin ligase complex. Modifications to the stereochemistry and regiochemistry of distal functionality on CELMoDs have been shown to have large effects on degradation activity and selectivity; however, methods allowing the rapid and selective introduction of enantioenriched moieties are rare. Herein, we report that classical CRBN-binding glutarimide cores can be successfully derivatized to aryl diazoacetates. These diazo derivatives, when in the presence of a dirhodium catalyst, successfully undergo high-yielding and highly enantioselective C–H functionalization of hydrocarbons and cyclopropanation of styrene. These products can be used to create not only molecular glue degrader-like compounds but also intermediates that can be elaborated into effective bifunctional ligand-directed degraders. Our findings highlight both the effectiveness of dirhodium catalysis in a drug discovery context and a new method for preparing diverse and stereoenriched glutarimide-containing compounds.

## Introduction

Cereblon E3 ligase modulatory drugs (CELMoDs) are an important class of compounds capable of the highly effective targeted degradation of proteins of interest.^1^ Interest in the development of novel CELMoDs has expanded rapidly in recent years due to their utility in the treatment of cancer and other diseases.^2^ By mimicking post-translational modifications recognized endogenously by cereblon (CRBN) via the ubiquitous imide contained in these structures, CELMoDs can recruit and mark previously undruggable proteins for degradation by the proteasome.^1,3^ The central mechanism of this degradation, in which a CELMoD binds to CRBN, stabilizes protein–protein interactions between CRBN and a neosubstrate, and subsequently induces polyubiquitination and degradation, is well-studied.^3,4^ While in silico study of novel CELMoDs is still somewhat nascent, much progress has been made in recent years, both in the context of CELMoDs and bifunctional liganddirected degraders (LDDs).^5^ Discovery of new and effective CELMoDs is limited both by the design complexities of ternary complex formation and by the limitations of the current synthetic methodologies used to prepare the structures.

**Figure 1:**
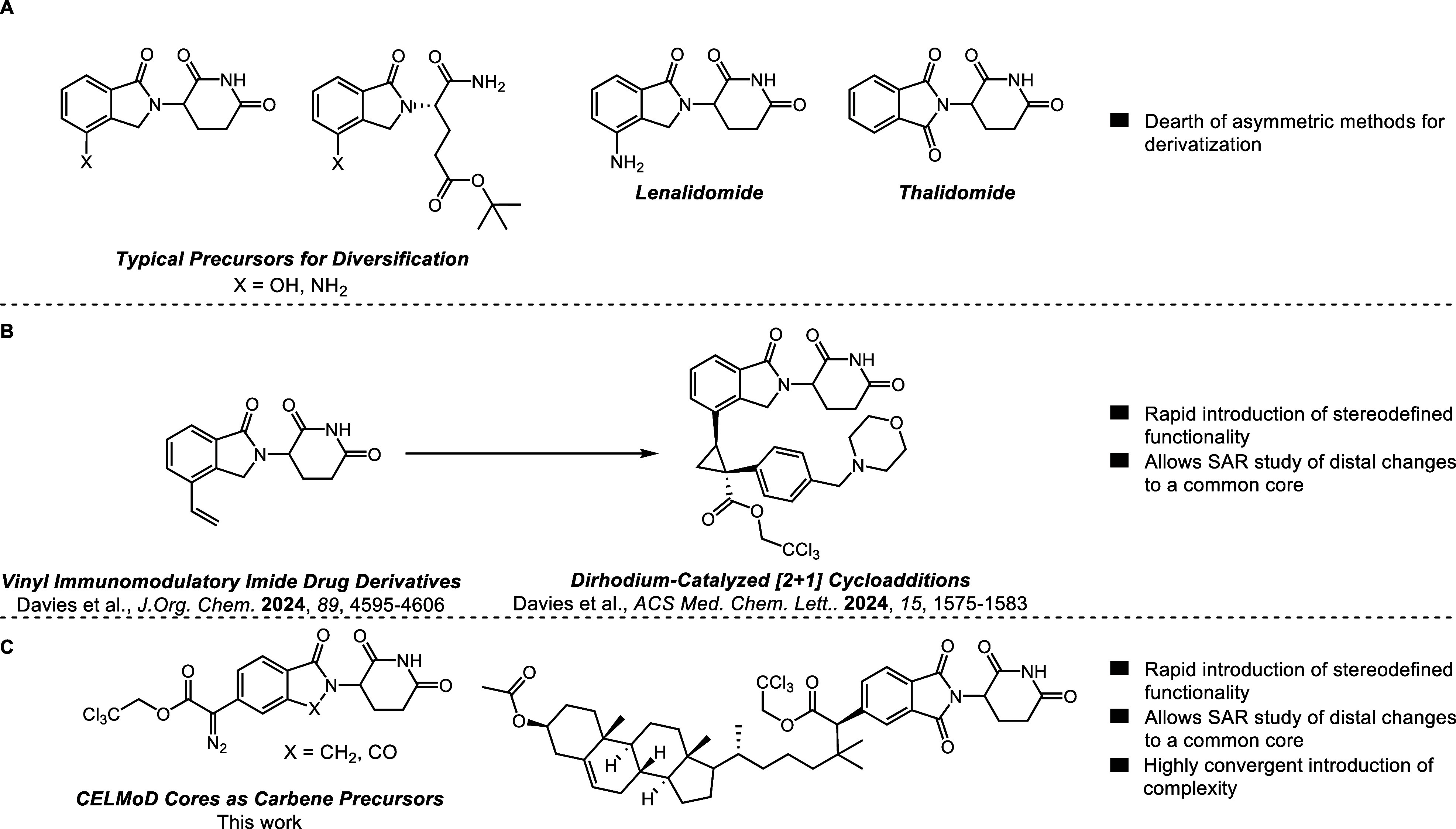
(A) Typical precursors for CELMoD diversification and representative CELMoDs in the clinic. (B) Previous efforts have expanded access to stereoenriched CELMoDs using carbene chemistry. (C) This work.

Glutarimide-containing compounds can be difficult to prepare due to synthetic challenges associated with their propensity to undergo hydrolytic ring-opening,^6^ their insolubility in many organic solvents, and their acidic glutarimide N–H proton, all of which limit their compatibility with many methodologies.^7^ Synthetic chemists have begun to address these factors, often including glutarimide-containing compounds as featured substrates for newly developed methods.^7,8^ While much of this research includes the well-precedented phthalimide (as in thalidomide) and isoindolinone (as in lenalidomide) cores, more recent research has focused on *N*- or *C*-linked (hetero)aryl glutarimides.^8a,8c,9^ However, many of the CELMoDs on the market or in development still contain the canonical phthalimide and isoindolinone cores (Figure 1A).^1^ Additionally, these canonical cores contain distal structural motifs, which are limited in stereochemical complexity and are derived from a relatively narrow set of precursors.^10^ The development of new methods for the convergent, enantioselective preparation of more complex glutarimide-containing structures, especially in the context of thalidomide-like and lenalidomide-like cores, remains important.^1^ In our own studies, we have expanded into new chemical space via dirhodium-catalyzed asymmetric cyclopropanation and cyclopropenation of CELMoD cores, enabled largely by an anhydrous, stereoretentive Suzuki–Miyaura coupling (Figure 1B).^11^ This allowed the synthesis of stereodefined, CRBN-modulating structures with a highly convergent, rapid introduction of diversity in a single step from vinyl and ethynyl CELMoD derivatives.^11b^ However, the diversity of these compounds is derived from the aryldiazoacetate component, which can be limiting. Since the discovery of effective glutarimide-containing degraders is often serendipitous, diversity-enabling methodologies are an especially important addition to the field.

The Davies laboratory has a long history of developing rhodium-carbene mediated C–H functionalization reactions.^12^ This has primarily involved the development of chiral dirhodium catalysts that function with aryldiazoacetates to perform highly stereo- and regioselective functionalization of primary, secondary, and tertiary C–H bonds in both activated and unactivated hydrocarbon substrates.^12,13^ We considered that utilizing CELMoD cores in a C–H functionalization context could both further increase chemical diversity and allow highly convergent syntheses. Herein, we show that aryldiazoacetatess can be successfully formed from CELMoD cores and serve as highly effective carbene precursors for diverse substrates in both C–H functionalization and cyclopropanation. This dirhodium-enabled method allows the preparation of both CELMoD-like structures and bifunctional LDDs with stereodefined functionality. We also demonstrate the positive influence of 1,1,1,3,3,3-hexafluoroisopropanol (HFIP) on dirhodium-catalyzed C–H functionalization, which we have previously shown to be both a nucleophile-deactivating agent in cyclopropanation of terminal olefins^14^ and, occasionally, enhance the enantioselectivity of carbene reactions.^15^ Finally, enantioselective transformations of glutarimide-containing compounds are rare in the literature. To our knowledge, our own previous work and an asymmetric reductive arylation reported by the Reisman laboratory represent some of the few examples in the literature.^9b,11b^

## Results and Discussion

**Scheme 1:**
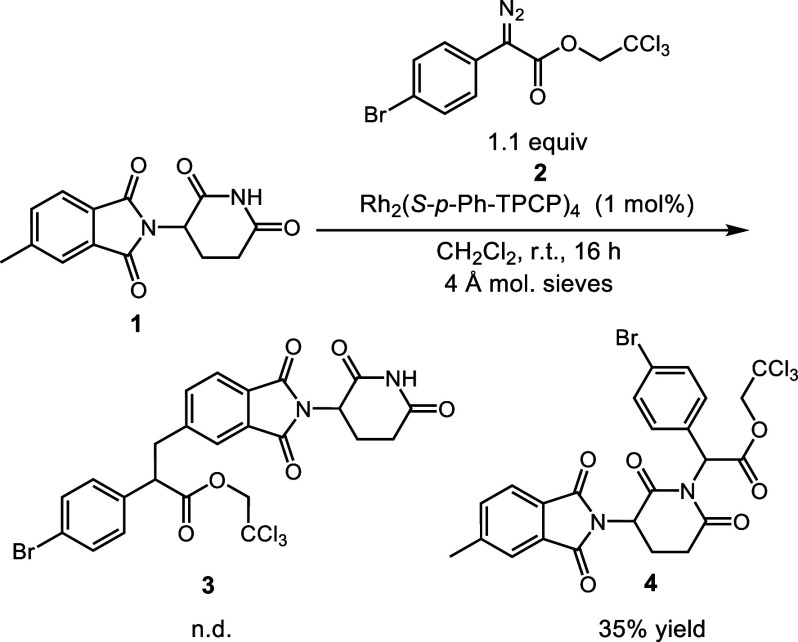
Unsuccessful C–H Functionalization of 5-Methylthalidomide

We initially envisioned the C–H functionalization of alkyl-substituted CELMoD cores using aryldiazoacetates as the carbene precursors (Scheme 1). To test this approach, we conducted a rhodium-catalyzed reaction between methyl-substituted CELMoD core **1** and aryldiazoacetate **2**. Unlike our previous cyclopropanation studies on vinyl or ethynyl CELMoD derivatives, we were unable to obtain any of the desired C–H functionalized product **3**. Instead, preferential insertion into the glutarimide N–H bond occurred, resulting in the formation of **4**.

To overcome this setback, we decided to change the strategy, incorporate the carbene functionality into the CELMoD core, and achieve diversity by the reaction of the carbene with a variety of substrates capable of undergoing C–H functionalization. At the outset of the project, we identified two potential challenges. First, it was unclear whether the compounds would be soluble enough in the halogenated or hydrocarbon solvents requisite in C–H functionalization reactions.^12^ Second, we wondered whether the glutarimide N–H would inhibit the reaction by poisoning the dirhodium catalyst or inserting into the carbenes (as seen in Scheme 1).

**Scheme 2:**
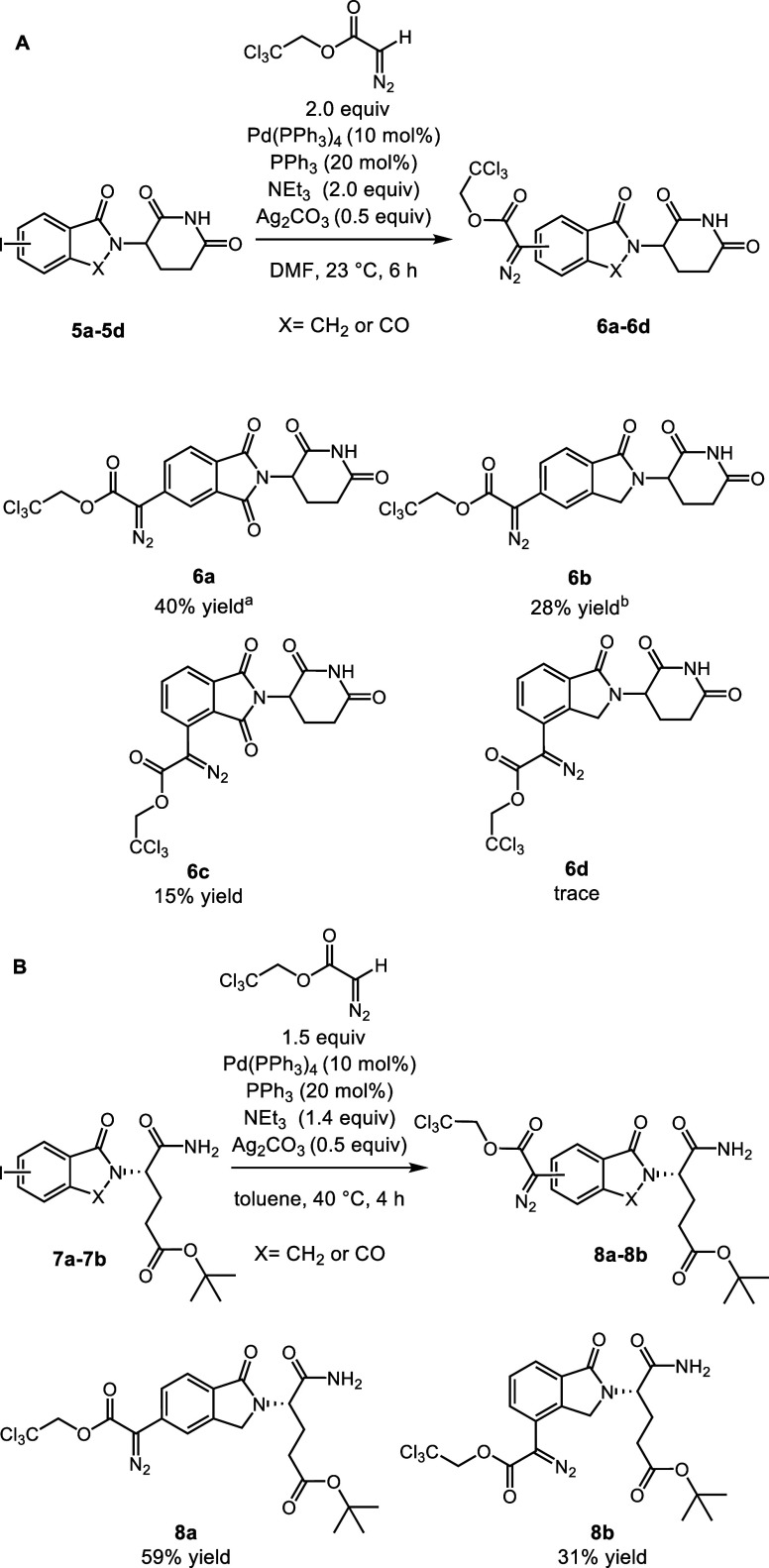
Synthesis of Carbene Precursors **6** and **8** When conducted in toluene, no product was observed. Reaction conducted with 6 equiv of diazo, 30 mol % Pd(PPh_3_)_4_, 60 mol % PPh_3_, 6 equiv NEt_3_, and 1 equiv Ag_2_CO_3_ in DMSO (0.20 M) for 16 h.

The first stage of the revised strategy was to prepare the trichloroethyl diazoacetate derivatives of the CELMoD core. Trichloroethyl esters were used because they tend to enhance C–H functionalization of unactivated C–H bonds and often result in higher levels of asymmetric induction.^16^ This was achieved via a slight modification of our previously published palladium-catalyzed cross-coupling of aryl iodides and 2,2,2-trichloroethyl diazoacetates (Scheme 2A).^17^ One difficulty with the iodo derivatives of ring-closed glutarimides (**5a**–**5d**) is their poor solubility in toluene, the typical solvent for the cross-coupling; however, DMF was a suitable alternative, resulting in the generation of the 5-substituted phthalimide and isoindolinone cores **6a** and **6b**. Compound **6a** was generated in 40% yield on a scale of 3.5 mmol. The 5-substituted diazo with an isoindolinone core (**6b**) required more forcing conditions than those of **6a** to give a usable yield. This cross-coupling is known to be difficult for *ortho*-substituted aryl iodides;^17b^ we previously reported that *ortho*-substituted aryl iodides were unsuccessful substrates under our conditions.^17a^ We were pleased to see that it was possible to extend the process to the 4-substituted phthalimide-core **6c**, albeit in low yield. Unfortunately, attempts to prepare the analogous 4-substituted isoindolinone core (**6d**) were unsuccessful. In addition to the challenge of the reaction at an *ortho*-substituted site, **6d** lacks the carbonyl of **6c**, which encourages oxidative addition by making the site more electron-deficient.

The ring-opened form of glutarimides is often used to circumvent the synthetic problems associated with ring-closed glutarimides and allow preparation of enantioenriched derivatives.^18^ Additionally, ring-opened forms of thalidomide (i.e., 4-pthalimidoglutaramic acid) are known to be less embryotoxic and neurotoxic relative to thalidomide.^19^ Since the enantioenriched ring-opened glutarimide can survive methods that might otherwise racemize the stereogenic center, we considered this to be a potential advantage over racemic ring-closed diazo compounds. As an added advantage, the corresponding ring-opened aryl iodide cores **7a** and **7b** are much more soluble, which allowed the easy preparation of the 4- and 5- substituted ring-opened diazo compounds **8a** and **8b** via the palladium cross-coupling (Scheme 2B). The formation of the 4-substituted isoindolinone **8b** is noteworthy because the corresponding ring-closed derivative **6d** could not be formed.

**Scheme 3:**
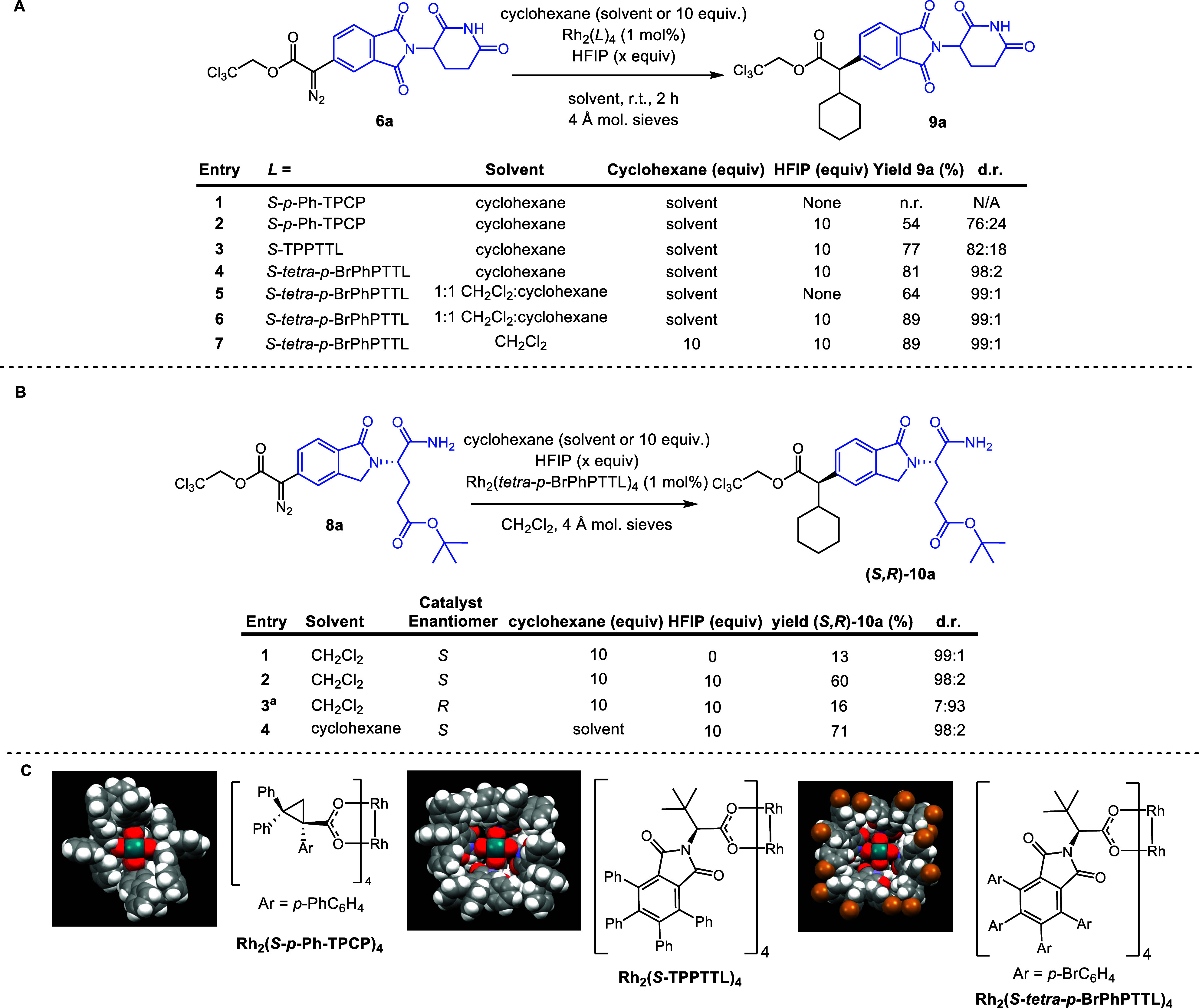
Development and Evaluation of Reaction Conditions for C–H Functionalization Reactions were conducted on a 0.1 mmol scale. Yields reported as isolated yields of purified material. Asymmetric induction determined by the SFC analysis. See the SI for details. ^a^Reaction with the *R* catalyst produced (*S*,*S*)-**10a** as the major diastereomer.

With precursors in hand, we began our C–H functionalization studies of cyclohexane using **6a** as the carbene precursor (Scheme 3A). Rh_2_(*S*-*p*-Ph-TPCP)_4_, when added as a solution to a suspension of the diazo compound in neat cyclohexane, does not react (entry 1) and remains a suspension. We hypothesized that the addition of HFIP might enable the solubilization of the diazo compounds. To our delight, when HFIP (10 equiv) is added to a reaction vessel before the addition of the catalyst, the C–H functionalized product **9a** is produced in 54% yield (entry 2).

Compounds **6a**–**c** are racemic. Both enantiomers react at essentially the same rate in the presence of the chiral catalysts, and the resulting diastereomeric products **9a** are formed with essentially the same levels of asymmetric induction. The reported diastereomeric ratio (d.r.) values represent the asymmetric induction generated at the carbene site by the chiral catalyst. While Rh_2_(*S*-*p*-PhTPCP)_4_ gave high stereoinduction in our previous study on cyclopropanation of vinyl CELMoD derivatives,^11b^**9a** was produced with modest asymmetric induction (76:24 d.r.). A better result was obtained with the C_4_-symmetric bowl-shaped catalyst Rh_2_(*S*-TPPTTL)_4_, which formed **9a** in a higher yield (77%) and asymmetric induction (82:18 d.r.) (entry 3). A more recently developed derivative of the TPPTTL scaffold, Rh_2_(*S*-*tetra*-*p-*BrPhPTTL)_4_,^20^ gave even better results, forming **9a** in excellent yield (81%) and with high levels of asymmetric induction (98:2 d.r.) (entry 4). The absolute configuration at the newly formed stereogenic center in **9a** is tentatively assigned as *R* by analogy to the assignments made in a related C–H functionalization with the same catalyst.^20^

After the identification of Rh_2_(*S*-*tetra*-*p-*BrPhPTTL)_4_ as a suitable catalyst, further optimization studies were conducted. When the reaction is conducted in a mixture of cyclohexane and dichloromethane, the reaction proceeds in the absence of HFIP (64% yield, entry 5); however, in the presence of HFIP, we obtained an 89% yield of **9a** (entry 6). The reaction was demonstrated to be equally effective when 10 equiv of cyclohexane was used (entry 7). In this case, the diazo compound **6a** was dissolved in dichloromethane with the aid of 10 equiv of HFIP and added over the course of 1 h to a solution of catalyst and 10 equiv of cyclohexane in dichloromethane.

Optimization studies were also carried out with the ring-opened derivative **8a**, which has a much greater solubility in dichloromethane compared with the ring-closed derivative **6a** (Scheme 3B). Consequently, slow addition of the substrate is possible without requiring HFIP to be present. However, in the absence of HFIP, **(***S,R***)***-***10a** is formed in only 13% yield (entry 1). In this reaction, we observed carbene dimerization as the major byproduct. When 10 equiv of HFIP was present in the reaction vessel, the desired reactivity was rescued, and **(***S,R***)***-***10a** was isolated in 60% yield with a high level of asymmetric induction at the site of the reaction (98:2 d.r.). In contrast, the reaction catalyzed by Rh_2_(*R*- *tetra*-*p-*BrPhPTTL)_4_ preferentially generates the other diastereomer of the product in only 16% yield (entry 3), compared with 60% yield for the matched reaction (entry 2). An improved yield (70%) of **(***S,R***)***-***10a** can be achieved in the Rh_2_(*S*-*tetra*-*p-*BrPhPTTL)_4_-catalyzed reaction by using cyclohexane as the solvent (entry 4).

**Scheme 4:**
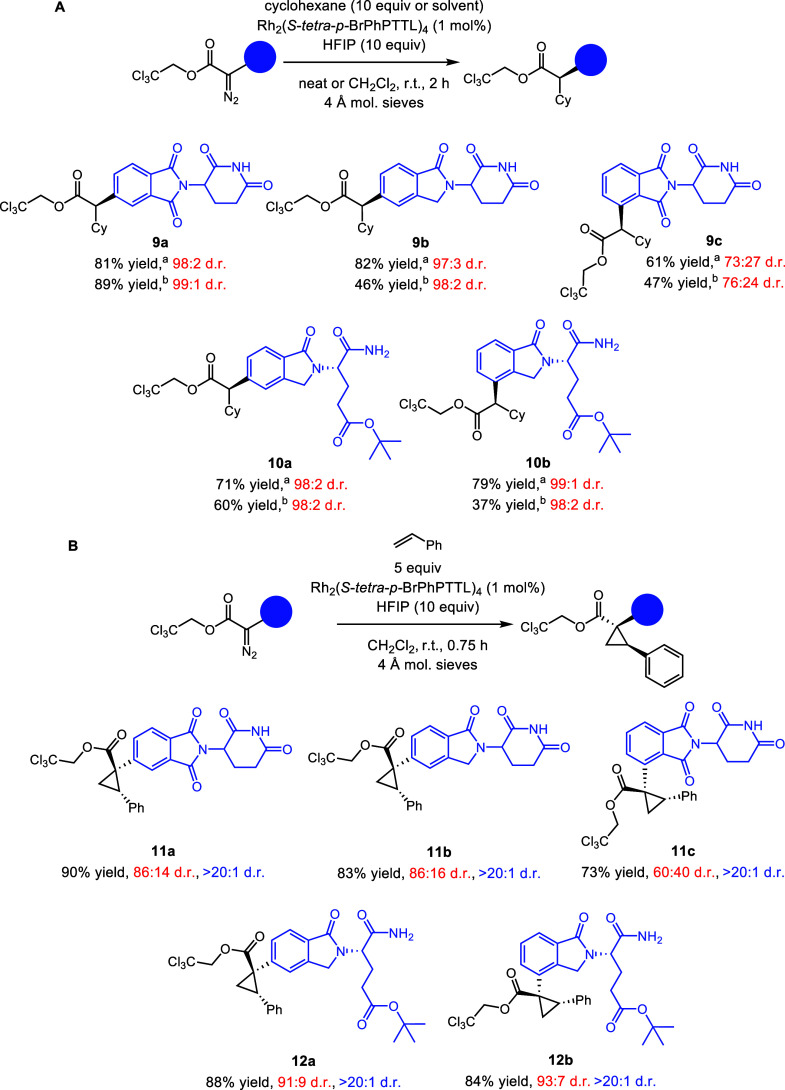
Scope of Diazo CELMoD Cores and Precursors in C–H Functionalization Reactions conducted on a 0.1 mmol scale. Yields reported as isolated yields of the purified material. The ring-closed glutarimide **6a** is a racemate, and the ring-opened derivative **8a** is the *S* enantiomer. Diastereomeric ratio (d.r.) in red represents asymmetric induction by catalysts and d.r. in blue represents the ratio for the two newly formed stereogenic centers. See the SI for details. ^a^Cyclohexane as the solvent. ^b^ Cyclohexane (10 equiv) as the trap.

Having established suitable reaction conditions for both the ring-closed and ring-opened CELMoD derivatives **6a** and **8a**, the other three carbene precursors **(6b**, **6c**, and **8b)** were tested in Rh_2_(*S*-*tetra*-*p-*BrPhPTTL)_4_-catalyzed reactions with cyclohexane. The C–H functionalization was robust with all three substrates, generating **9b**, **9c**, and **10b** in good yields with high levels of asymmetric induction (Scheme 4a). Catalyst addition to a stirred solution of the diazo precursor in neat cyclohexane is the superior method when compared to the reverse, i.e., the addition of the diazo solution slowly to 10 equiv of cyclohexane in CH_2_Cl_2_, which gives byproducts derived from carbene dimerization.

These precursors are also competent in the cyclopropanation reactions, as illustrated in the Rh_2_(*S*-*tetra*-*p-*BrPhPTTL)_4_-catalyzed reactions with styrene (Scheme 4b). As cyclopropanation of styrene is generally a much more favorable reaction than C–H functionalization, these reactions were carried out with just 5 equiv of styrene. In all cases, the desired cyclopropane **11a**–**c**, **12a**, and **12b** were produced. We observed lower levels of asymmetric induction for these products; however, due to the high reactivity of styrene, we saw excellent yields. The absolute configuration at the newly formed stereogenic centers in **11a** is tentatively assigned as 1*R*, 2*S* by analogy to assignments made previously from X-ray crystal structures of similar products formed from the reaction of aryldiazoacetates with Rh_2_(*S*-*tetra*-*p-*BrPhPTTL)_4_.^14a^ This study focused on styrene to illustrate the effectiveness of the reaction when the carbene is located at different positions on the core of the glutarimide derivative. However, previous studies have shown that a wide range of aryl- and heteroaryl vinyl derivatives can be employed in cyclopropanation with donor/acceptor carbenes.^14b^

**Scheme 5:**
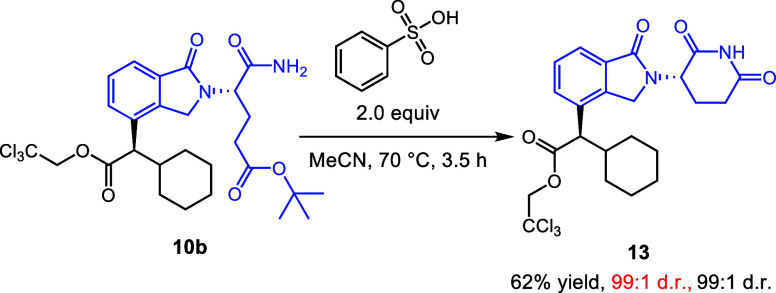
Stereoretentive Ring Closure of **10b**

Ring-opened products such as **10b** can be subjected to acid-mediated ring closure with retention of stereochemistry at the site of the carbene reaction (red d.r.) and at the glutarimide stereogenic center (black d.r.) (Scheme 5).^18a^ This allows the creation of C–H functionalization derivatives enriched at the glutarimide stereogenic center, which, in the case of **10b**, gives an enantioenriched product that could not be reached by direct synthesis of the corresponding diazo compound (see **6d**, Scheme 2). Cyclization was also successful with acyclic precursors **10a**, **12a**, and **12b** (see the Supporting Information, compounds **SI2–SI4**).

**Scheme 6:**
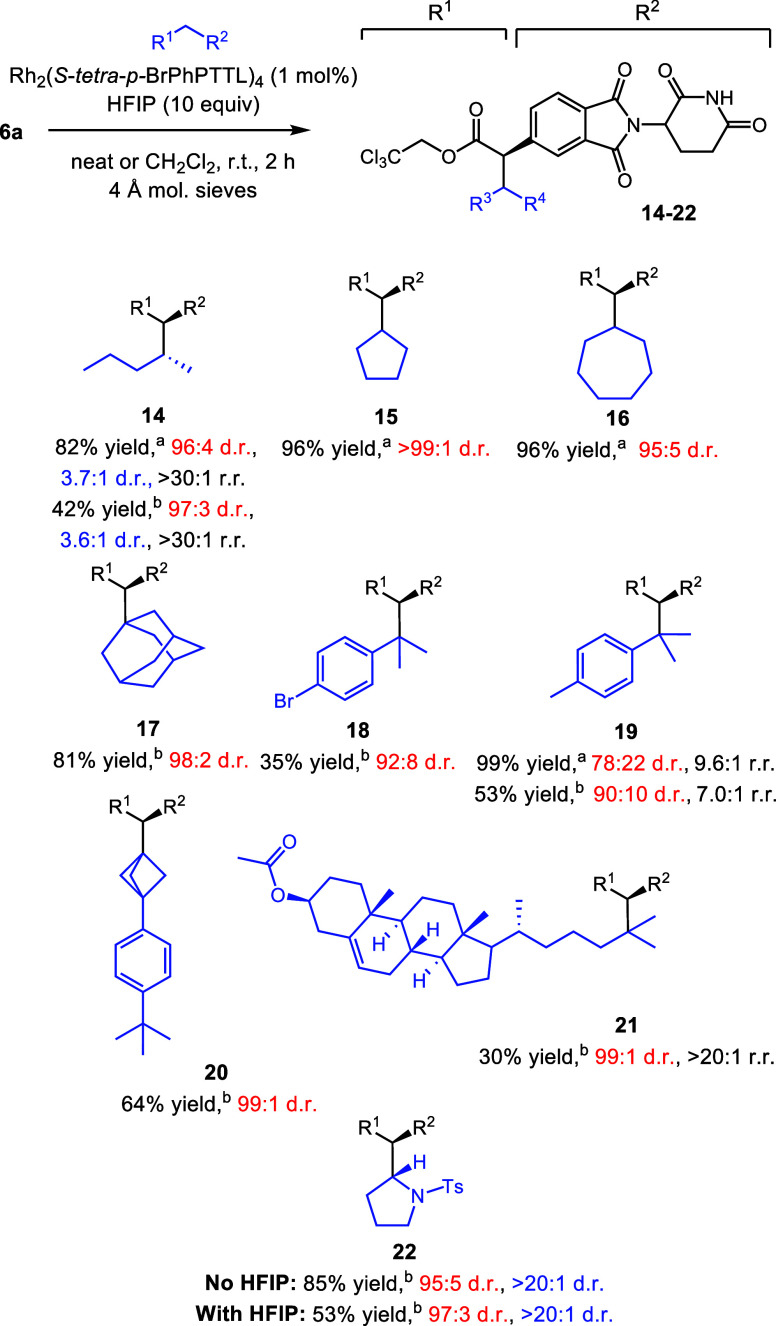
Scope of C–H Functionalization with **6a** Reactions conducted on a 0.1 mmol scale. Yields reported as isolated yields of the purified material. Diastereomeric ratio (d.r.) in red represents asymmetric induction by catalysts and d.r. in blue represents the ratio for the two newly formed stereogenic centers. See the SI for details. ^a^Substrate used as the solvent. ^b^10 equiv of the substrate used.

Our previous cyclopropanation studies indicated that the 5-substituted CELMoD cores tended to be among the more biologically active derivatives.^11b^ Therefore, we focused on the preparation of C-5-substituted products using phthalimide **6a** to investigate the scope of C–H functionalization (Scheme 6). Catalyst addition into a suspension of diazo compounds in the neat substrate and HFIP allows for the high-yielding functionalization of simple hydrocarbons, as illustrated in the formation of **14–16**. The reaction of pentane to form **14** is of note, showing exquisite selectivity for secondary C–H functionalization products, with high asymmetric induction (96:4 d.r.) and preference of C2 over C3 (>30:1 r.r.). The addition of the diazo compound in dichloromethane and HFIP into a solution of the catalyst and substrate allowed the facile generation of compounds **17–21**. While the mild electron-withdrawing nature of the bromide causes **18** to be produced in a lower 35% yield, it offers the potential for further functionalization. When a benzylic tertiary site is pitted against a benzylic primary site, the tertiary site is favored, as seen in the formation of **19**. The yield of **19** is essentially quantitative when *p*-cymene is used as the solvent; however, there is variation in selectivity depending on the amount of *p*-cymene used, with enhanced regioselectivity and decreased asymmetric induction observed when *p*-cymene is used as the solvent. The tertiary functionalization product of 1-(4-(*tert*-butyl)phenyl)bicyclo[1.1.1]pentane (**20**) and **6a** is produced in 64% yield and 99:1 dr while preserving the strained carbocycle. One particular highlight is the functionalization of cholesteryl acetate. Compound **6a** inserts only into the tertiary site of the long alkyl chain of cholesteryl acetate with high diasteroselectivity; we do not observe any other functionalization products in the reaction mixture (**21**). Compound **6a** is also a competent diazo precursor when it is used to functionalize nitrogen-containing heterocycles. The C2-functionalized *N*-tosyl pyrrolidine (product **22**) is produced in modest yield and excellent d.r. We hypothesized that HFIP hydrogen-bonding interactions with the substrate might be interfering with the reaction by making the α-proton less hydridic.^14a^ We modified the reaction to include only 10 equiv of the substrate in CH_2_Cl_2_ with no added HFIP and added a 20 mM solution of the catalyst. The higher reactivity of the C–H bond adjacent to nitrogen compared to a less activated C–H bond allows excellent reactivity, giving **22** in 85% yield and 95:5 d.r.

**Scheme 7:**
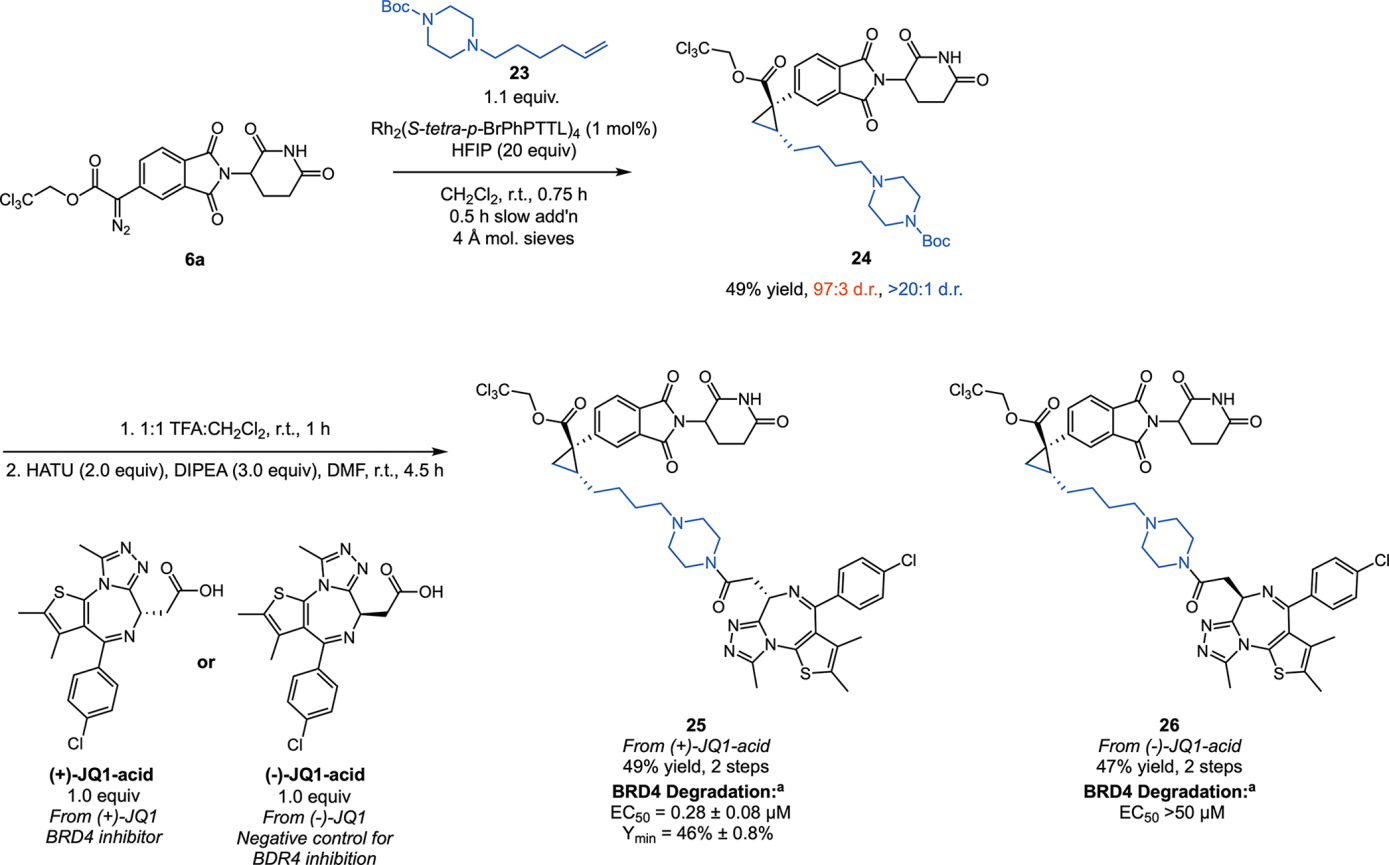
Synthesis of BRD4 Degrading Compounds Using Asymmetric Cyclopropanation EC_50_ indicates the concentration required to achieve 50% of total degradation effect, and *Y*_min_ indicates depth of degradation, with 100% representing no reduction in protein level and 0% representing complete degradation; data reported as an average of *N* = 3 test occasions.

CELMoD-based diazo compounds are demonstrably effective in the creation of relatively small and stereodefined structures. Glutarimides have not only been leveraged productively as molecular glue degraders but also within bifunctional LDDs.^1^ In the context of CRL4^CRBN^ -based LDDs, the inversion of linker stereochemistry proximal to the CRBN has been shown to significantly impact degradation selectivity.^21^ For this reason, we wondered whether our method could be leveraged to install a linker with suitable functionality to enable the synthesis of novel LDDs. We approached this by preparing a protected piperazine with a 6-carbon chain terminated by an alkene as a model substrate (Scheme 7, **23**). The cyclopropanation reaction can be carried out with 1.1 equiv of trap **23** to form **24** in 49% yield with excellent relative and absolute stereochemical control. Boc deprotection with trifluoroacetic acid and subsequent amide coupling with the carboxylic acid of potent bromodomain 4 (BRD4) inhibitor (+)-JQ1 and its inactive enantiomeric partner produce **25** and **26** in up to 49% yield over two steps. The ability of **25** and **26** to degrade BRD4 was assessed in a HiBiT assay in A549 cells. Gratifyingly, **25** displayed modest BRD4 degradation (EC_50_ = 0.28 μM, 46% *Y*_min_), while the negative control **26**, which contains a BRD4 ligand with substantially lower binding affinity, did not significantly degrade BRD4 at concentrations of up to 50 μM. These results demonstrate that this method is highly enabling for the rapid assembly of LDDs.

**Figure 2:**
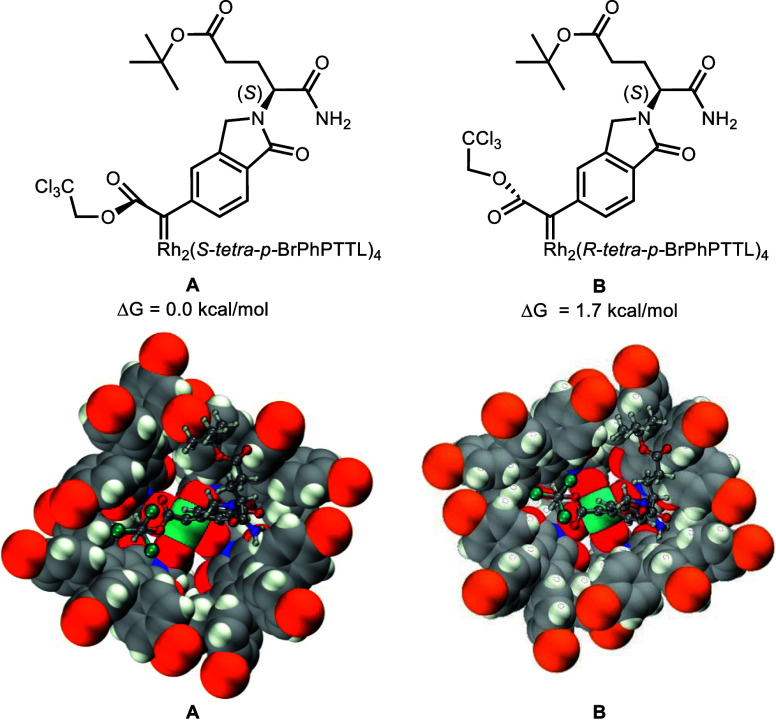
DFT-optimized structures of **8a** as a carbene complex with Rh_2_(*S*-*tetra*-*p-*BrPhPTTL)_4_ (A) and Rh_2_(*R*-*tetra*-*p-*BrPhPTTL)_4_ (B).

From a catalyst design perspective, one of the most intriguing features of these transformations was the behavior of enantiomerically pure ring-opened substrate **8a** in the presence of the two enantiomers of Rh_2_(*tetra*-*p-*BrPhPTTL)_4_ (Scheme 3B). Even though the stereocenter in **8a** is far removed from the diazoacetate, the efficiency of the C–H functionalization reaction is significantly impacted due to a matched/mismatched relationship between the catalyst and the substrate. The reaction with Rh_2_(*S*-*tetra*-*p-*BrPhPTTL)_4_ proceeds to form **10a** in high yield, whereas the reaction with Rh_2_(*R*-*tetra*-*p-*BrPhPTTL)_4_ is very low yielding. This is intriguing, as both enantiomers of the catalyst produce the C–H functionalization products with opposite but high levels of asymmetric induction. In the past, we have observed secondary interactions between the wall of the catalysts and the approaching substrate, which could cause a distal functionality to influence carbene reactivity.^22^ We hypothesize that the influence of the stereogenic center on the yield is due to the bowl shape of the catalysts favoring different orientations of **8a** within the catalyst. We confirmed this possibility by conducting density functional theory (DFT) calculations to model the relative stability of the rhodium carbene intermediates of **8a** in Rh_2_(*S*-*tetra*-*p-*BrPhPTTL)_4_ and Rh_2_(*R*-*tetra*-*p-*BrPhPTTL)_4_ (Figure 2). Given the size of the catalyst-carbene system (up to 400 atoms), we employed the two-layer ONIOM (B3LYP:UFF) approach (see the SI for details). The metal-carbene intermediate **A** resulting from Rh_2_(*S*-*tetra*-*p-*BrPhPTTL)_4_ is thermodynamically more stable than **B** (Rh_2_(*R*-*tetra*-*p-*BrPhPTTL)_4_) by 1.7 kcal/mol. A closer structural analysis revealed that the ring-opened side chain of the carbene fragment and the trichloroethyl acetate folded inside are arranged differently in **A** versus **B**. Specifically, the ring-opened side chain in **B** folds to the trichloroethyl acetate group (*Si* face) of the carbene, where the reaction with the substrate and Rh_2_(*R*-*tetra*-*p-*BrPhPTTL)_4_ occurs, while the ring-opened side chain in **A** folds to the opposite side of the trichloroethyl acetate group. As a result, the open face in **A** is less sterically demanding than that in **B**, allowing better reactivity. These results illustrate the subtle effects that the secondary interactions with the wall of these bowl-shaped catalysts can have in controlling the outcome of rhodium carbene transformations.

## Conclusions

In conclusion, CELMoD-core aryl diazoacetates are dirhodium carbene precursors of exceptional utility in C–H functionalization and cyclopropanation. High levels of diastereoselectivity and regioselectivity complement good yields and mild reaction conditions. We have demonstrated that diverse and stereodefined C*sp*^3^-rich functionality can rapidly be introduced into CELMoD cores in a single step. Additionally, this work shows the utility of HFIP in the context of dirhodium-catalyzed C–H functionalization, both as a solubilizing agent and as a nucleophile-deactivating agent. This enables not only the generation of molecular glue degrader-like structures but also, with further diversification, the creation of biologically active, stereodefined LDDs. Overall, this work brings together the synthetic utility of rhodium-carbene chemistry and a class of valuable compounds to redefine how novel, complex, and medicinally relevant structures can be made.

## Supporting Information Available

Detailed experimental procedures; ^1^H and ^13^C{^1^H} NMR spectra for all compounds; chromatograms; computational details; and biological assay protocols and data (PDF)

## Abbreviations

BRD4: Bromodomain-containing protein 4
CELMoD: Cereblon E3 ligase modulatory drug
CRBN: cereblon
DMF: dimethylformamide
HFIP: 1,1,1,3,3,3-hexafluoroisopropano
LDD: ligand-directed degrader
*p*-Ph-TPCP: *para*-phenyl 1,2,2-triarylcyclopropane-carboxylate
SFC: supercritical fluid chromatography
tetra-p-BrPPTTL: 4,5,6,7-tetrakis(4-bromophenyl)phthalimidotert-leucine
TPPTTL: tetraphenylphthalimido-tert-leucine
DFT: density functional theory

## References

[ref1] Tsai J. M.; Nowak R. P.; Ebert B. L.; Fischer E. S. Targeted protein degradation: from mechanisms to clinic. Nat. Rev. Mol. Cell Biol. 2024, 25, 740–757. 10.1038/s41580-024-00729-9. 38684868

[ref2] aBartlett J. B.; Dredge K.; Dalgleish A. G. The evolution of thalidomide and its IMiD derivatives as anticancer agents. Nat. Rev. Cancer 2004, 4, 314–322. 10.1038/nrc1323. 15057291

[ref3] Ichikawa S.; Flaxman H. A.; Xu W.; Vallavoju N.; Lloyd H. C.; Wang B.; Shen D.; Pratt M. R.; Woo C. M. The E3 ligase adapter cereblon targets the C-terminal cyclic imide degron. Nature 2022, 610, 775–782. 10.1038/s41586-022-05333-5. 36261529 PMC10316063

[ref4] aCao S.; Kang S.; Mao H.; Yao J.; Gu L.; Zheng N. Defining molecular glues with a dual-nanobody cannabidiol sensor. Nat. Commun. 2022, 13, 815 10.1038/s41467-022-28507-1. 35145136 PMC8831599

[ref5] aWeiss D. R.; Bortolato A.; Sun Y.; Cai X.; Lai C.; Guo S.; Shi L.; Shanmugasundaram V. On Ternary Complex Stability in Protein Degradation: In Silico Molecular Glue Binding Affinity Calculations. J. Chem. Inf. Model. 2023, 63, 2382–2392. 10.1021/acs.jcim.2c01386. 37037192

[ref6] aSchumacher H.; Smith R. L.; Williams R. T. The Metabolism of Thalidomide: The Fate of Thalidomide and Some of its Hydrolysis Products in Various Species. Br. J. Pharmacol. 1965, 25, 338–351. 10.1111/j.1476-5381.1965.tb02054.x. PMC1510729 5866716

[ref7] Sosič I.; Bricelj A.; Steinebach C. E3 ligase ligand chemistries: from building blocks to protein degraders. Chem. Soc. Rev. 2022, 51, 3487–3534. 10.1039/D2CS00148A. 35393989

[ref8] aNorris S.; Ba X.; Rhodes J.; Huang D.; Khambatta G.; Buenviaje J.; Nayak S.; Meiring J.; Reiss S.; Xu S.; Shi L.; Whitefield B.; Alexander M.; Horn E. J.; Correa M.; Tehrani L.; Hansen J. D.; Papa P.; Mortensen D. S. Design and Synthesis of Novel Cereblon Binders for Use in Targeted Protein Degradation. J. Med. Chem. 2023, 66, 16388–16409. 10.1021/acs.jmedchem.3c01848. 37991844

[ref9] aGu J. W.; Oderinde M. S.; Li H.; Roberts F.; Ganley J. M.; Palkowitz M. D. Expedited Aminoglutarimide C–N Cross-Coupling Enabled by High-Throughput Experimentation. J. Org. Chem. 2024, 89, 17738–17743. 10.1021/acs.joc.4c02536. 39545828

[ref10] Sasso J. M.; Tenchov R.; Wang D.; Johnson L. S.; Wang X.; Zhou Q. A. Molecular Glues: The Adhesive Connecting Targeted Protein Degradation to the Clinic. Biochemistry 2023, 62, 601–623. 10.1021/acs.biochem.2c00245. 35856839 PMC9910052

[ref11] aTracy W. F.; Davies G. H. M.; Grant L. N.; Ganley J. M.; Moreno J.; Cherney E. C.; Davies H. M. L. Anhydrous and Stereoretentive Fluoride-Enhanced Suzuki–Miyaura Coupling of Immunomodulatory Imide Drug Derivatives. J. Org. Chem. 2024, 89, 4595–4606. 10.1021/acs.joc.3c02873. 38452367 PMC11002932

[ref12] Davies H. M. L. Finding Opportunities from Surprises and Failures. Development of Rhodium-Stabilized Donor/Acceptor Carbenes and Their Application to Catalyst-Controlled C–H Functionalization. J. Org. Chem. 2019, 84, 12722–12745. 10.1021/acs.joc.9b02428. 31525891 PMC7232105

[ref13] Davies H. M. L.; Liao K. Dirhodium tetracarboxylates as catalysts for selective intermolecular C–H functionalization. Nat. Rev. Chem. 2019, 3, 347–360. 10.1038/s41570-019-0099-x. 32995499 PMC7521778

[ref14] aSharland J. C.; Dunstan D.; Majumdar D.; Gao J.; Tan K.; Malik H. A.; Davies H. M. L. Hexafluoroisopropanol for the Selective Deactivation of Poisonous Nucleophiles Enabling Catalytic Asymmetric Cyclopropanation of Complex Molecules. ACS Catal. 2022, 12, 12530–12542. 10.1021/acscatal.2c03909.

[ref15] aVaitla J.; Boni Y. T.; Davies H. M. L. Distal Allylic/Benzylic C–H Functionalization of Silyl Ethers Using Donor/Acceptor Rhodium(II) Carbenes. Angew. Chem., Int. Ed. 2020, 59, 7397–7402. 10.1002/anie.201916530. PMC7233467 31908146

[ref16] Guptill D. M.; Davies H. M. L. 2,2,2-Trichloroethyl Aryldiazoacetates as Robust Reagents for the Enantioselective C–H Functionalization of Methyl Ethers. J. Am. Chem. Soc. 2014, 136, 17718–17721. 10.1021/ja5107404. 25474724

[ref17] aFu L.; Mighion J. D.; Voight E. A.; Davies H. M. L. Synthesis of 2,2,2,-Trichloroethyl Aryl- and Vinyldiazoacetates by Palladium-Catalyzed Cross-Coupling. Chem. – Eur. J. 2017, 23, 3272–3275. 10.1002/chem.201700101. 28093820

[ref18] aZacuto M. J.; Traverse J. F.; Geherty M. E.; Bostwick K. F.; Jordan C.; Zhang C. Chirality Control in the Kilogram-Scale Manufacture of Single-Enantiomer CELMoDs: Synthesis of Iberdomide·BSA, Part 2. Org. Process Res. Dev. 2024, 28, 57–66. 10.1021/acs.oprd.3c00314.

[ref19] Fabro S.; Schumacher H.; Smith R. L.; Stagg R. B. L.; Williams R. T. The metabolism of thalidomide: Some biological effects of thalidomide and its metabolites. Br. J. Pharmacol. 1965, 25, 352–362. 10.1111/j.1476-5381.1965.tb02055.x. PMC1510743 5866717

[ref20] Garlets Z. J.; Boni Y. T.; Sharland J. C.; Kirby R. P.; Fu J.; Bacsa J.; Davies H. M. L. Design, Synthesis, and Evaluation of Extended C4–Symmetric Dirhodium Tetracarboxylate Catalysts. ACS Catal. 2022, 12, 10841–10848. 10.1021/acscatal.2c03041. 37274599 PMC10237630

[ref21] aRobbins D. W.; Noviski M. A.; Tan Y. S.; Konst Z. A.; Kelly A.; Auger P.; Brathaban N.; Cass R.; Chan M. L.; Cherala G.; Clifton M. C.; Gajewski S.; Ingallinera T. G.; Karr D.; Kato D.; Ma J.; McKinnell J.; McIntosh J.; Mihalic J.; Murphy B.; Panga J. R.; Peng G.; Powers J.; Perez L.; Rountree R.; Tenn-McClellan A.; Sands A. T.; Weiss D. R.; Wu J.; Ye J.; Guiducci C.; Hansen G.; Cohen F. Discovery and Preclinical Pharmacology of NX-2127, an Orally Bioavailable Degrader of Bruton’s Tyrosine Kinase with Immunomodulatory Activity for the Treatment of Patients with B Cell Malignancies. J. Med. Chem. 2024, 67, 2321–2336. 10.1021/acs.jmedchem.3c01007. 38300987

[ref22] aFu J.; Ren Z.; Bacsa J.; Musaev D. G.; Davies H. M. L. Desymmetrization of cyclohexanes by site- and stereoselective C–H functionalization. Nature 2018, 564, 395–399. 10.1038/s41586-018-0799-2. 30568203

[ref23] Green S. P.; Wheelhouse K. M.; Payne A. D.; Hallett J. P.; Miller P. W.; Bull J. A. Thermal Stability and Explosive Hazard Assessment of Diazo Compounds and Diazo Transfer Reagents. Org. Process Res. Dev. 2020, 24, 67–84. 10.1021/acs.oprd.9b00422. 31983869 PMC6972035

