# Supplementary material for: Diversity Synthesis Using Glutarimides as Rhodium Carbene Precursors in Enantioselective C–H Functionalization and Cyclopropanation
Source: J Am Chem Soc. 2025 Mar 18;147(13):11336–45. doi: 10.1021/jacs.5c00568 (PMC11969559; doi:10.1021/jacs.5c00568)
Supplement: Supplementary file 1 — ja5c00568_si_001.pdf [file ja5c00568_si_001.pdf]

Diversity Synthesis Using Glutarimides as Rhodium Carbene Precursors in Enantioselective C-H Functionalization and Cyclopropanation

Supporting Information
(140 Pages)

William F. Tracy,[a] Jack C. Sharland,[a, †] Duc Ly,[a] Geraint H. M. Davies,[b, †] Djamaladdin G. Musaev,[a] Hua Fang,[d] Jesus Moreno,*[,c] Emily C. Cherney,*[,d] Huw M. L. Davies*[,a]

[a] Department of Chemistry, Emory University, Atlanta, Georgia 30322, United States; Email: hmdavie@emory.edu

[b] Discovery & Development Sciences, Bristol Myers Squibb, Cambridge, Massachusetts 02143, United States

[c] Discovery & Development Sciences, Bristol Myers Squibb, San Diego, California 92121, United States; Email: jesus.moreno@bms.com

[d] Discovery & Development Sciences, Bristol Myers Squibb, Princeton, New Jersey 08543, United States; Email: emily.cherney@bms.com

*Corresponding authors:

Huw M. L. Davies − Department of Chemistry, Emory University, Atlanta, Georgia 30322, United States; Email: hmdavie@emory.edu
Emily C. Cherney – Discovery Sciences, Bristol Myers Squibb, Princeton, New Jersey 08543, United States; Email: emily.cherney@bms.com
Jesus Moreno – Discovery Sciences, Bristol Myers Squibb, San Diego, California 92121, United States; Email: jesus.moreno@bms.com

Present Addresses

†**Jack C. Sharland** – PTC Therapeutics, 350 North Bernardo Avenue; Mountain View, CA 94043, United States

†**Geraint H. M. Davies** − PostEra, One Broadway; 14th Floor; Cambridge, MA 02142, United States

Table of Contents

Section 1: General Information S2
Figure S1. Aryl iodides used in this study... S3

Section 2: Synthetic Procedures and Compound Characterization S4
Synthesis of Starting Materials S4
Synthesis of Final Compounds S8
General Procedure 1 (GP1) S8
General Procedure 2 (GP2) S8
General Procedure 3 (GP3) S8
General Procedure 4 (GP4) S8
General Procedure 5 (GP5) S9
General Procedure 6 (GP6) S9
General Procedure 7 (GP7) S9
General Procedure 8 (GP8) S9
Section 3: Computational Details S31
Section 4: Spectroscopic Data S72
^{1}H NMR Spectra S72
$^{13}C\{^{1}H\}$ NMR Spectra S90
Supplementary NMR Spectra S107
Section 5: Chromatographic Data S111
Chiral SFC Chromatograms S111
Section 6: Assay Protocols S138
Section 7: References S139

Section 1: General Information

All reactions were conducted in flame-dried glassware under an inert atmosphere of dry nitrogen or argon, unless otherwise noted. All reagents were purchased from commercial suppliers and used as received, unless otherwise noted. Anhydrous dichloromethane was obtained from a Grubbs-type solvent purification system and further dried under an argon atmosphere for 24 hours over 4 Å molecular sieves. Anhydrous *N,N*-dimethylformamide, dimethyl sulfoxide, and toluene were obtained from DriSolv® Supelco® bottles. Molecular sieves were activated by heating under vacuum (<1 torr) for three hours at 300 °C. 1,1,1,3,3,3-hexafluoroisopropanol was obtained from Oakwood Chemical, distilled, and stored under inert atmosphere over 4 Å molecular sieves. Pentane, cyclopentane, cyclohexane, cycloheptane, *p*-bromo cumene, and *p*-cymene were stored over 4 Å molecular sieves for at least 24 hours prior to reaction. The diazo compounds synthesized were stored at -20 °C and were stored for 9 months without any signs of degradation.

Proton (^{1}H) NMR spectra were recorded at 400 MHz on Varian and Bruker spectrometers, 500 MHz on an Inova-500 spectrometer, 600 MHz on an Inova-600 spectrometer, or 800 MHz on a Bruker-800 spectrometer. Proton-decoupled carbon ($^{13}C\{^1H\}$) NMR spectra were recorded at 100 MHz on Varian and Bruker spectrometers, 151 MHz on an Inova-600 spectrometer, or 201 MHz on a Bruker-800 spectrometer. NMR spectra were recorded in deuterated solvents and were referenced to tetramethylsilane or the respective residual solvent signal. NMR chemical shifts were reported in parts per million (ppm). Abbreviations for signal couplings are as follows: s, singlet; d, doublet; t, triplet; q, quartet; quin, quintet; sex, sextet; sep, septet; m, multiplet; dd, doublet of doublets; dt, doublet of triplets; dtd, doublet of triplet of doublets; ddt, doublet of doublet of triplets; and dq, doublet of quartets. The coupling constants were taken from the spectra directly and are uncorrected.

Mass spectrometric determinations were carried out on a Thermo Finnigan LTQ-FTMS spectrometer with atmospheric pressure chemical ionization (APCI) or electrospray ionization (ESI) using a Fourier transform ion cyclotron resonance mass analyzer. Optical rotations were measured on a Rudolph Research Analytical Automatic Polarimeter, APIV-1W. Analytical thin layer chromatography was performed on silica gel plates using ultraviolet light, iodine vapor, or potassium permanganate stain to visualize analytes. Normal phase column chromatography was performed with SiliCycle silica gel 60 Å (50 μm) or neutral alumina hand-packed in Biotage Sfär columns on Biotage Isolera Four chromatographs or hand-packed glass columns, with SiliCycle silica gel 60 Å or Celite® as a dry-loading absorbent. Reverse phase chromatography was conducted using Teledyne ISCO RediSep Gold 18 reverse-phase columns using Teledyne ISCO CombiFlash or Biotage Isolera chromatographs. Supercritical fluid chromatography (SFC) analysis was performed on a Waters Acquity UPC2 instrument.

Purity of biologically tested compounds was tested by high-performance liquid chromatography analysis (HPLC) on an Agilent 1260 Infinity HPLC. All tested compounds were ≥95% pure by HPLC analysis.

1-(4-(tert-butyl)phenyl)bicyclo[1.1.1]pentane was prepared following the literature procedure.[1] 2,2,2-trichloroethyl 2-(4-bromophenyl)-2-diazoacetate (**2**) was prepared via the literature procedure.[2] 2,2,2-trichloroethyl 2-diazoacetate was also prepared following the literature procedure.[3] Dirhodium catalysts Rh_2(*S*-p-Ph-TPCP)$_4$,[4] Rh_2(*S-tetra-p*-BrPhPTTL)$_4$,[5] Rh_2(*R-tetra-p*-BrPhPTTL)$_4$,[5] and Rh_2(*S*-TPPTTL)$_4$[6] were prepared according to their respective literature procedures.

Racemic standards for the products of reactions with ring-closed diazo compounds were prepared by using a 1:1 mixture of *R* and *S* catalyst under the same reaction conditions used to prepare enantioenriched compounds. Racemic standards for the cyclopropanation of styrene with ring-opened diazo compounds were prepared using $Rh_2(esp)_2$ under the same reaction conditions used to prepare enantioenriched diazo compounds. Racemic standards for the C-H insertion of cyclohexane using ring-opened diazo compounds were prepared from the enantioenriched material by epimerization with 1,8-diazabicyclo[5.4.0]undec-7-ene in acetonitrile. Racemic standards for **13** and **SI-3** were prepared from the racemic standards for **11b** and **12b** using **GP 8** (detailed below) and the resulting glutarimide stereogenic centers were epimerized with 1,8-diazabicyclo[5.4.0]undec-7-ene in acetonitrile.

Caution! Glutarimide-containing compounds such as thalidomide are known reproductive, neurological, and hematological toxins. Care must be exercised to avoid direct contact with glutarimide-containing compounds. The neat compounds and their solutions must only be handled in a chemical fume hood. Any glassware used with glutarimide-containing material should be treated with a 2 M aqueous solution of a strong base such as sodium hydroxide to destroy the material. Caution! Diazo compounds are potentially energetic and must be handled carefully; initiation temperatures for similar compounds are often below 100 °C.[7] Off-gassing of nitrogen during rhodium-catalyzed reactions with diazo compounds must be accounted for in reaction setup.

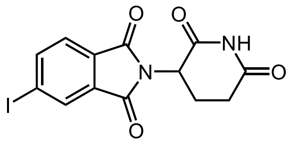

5a

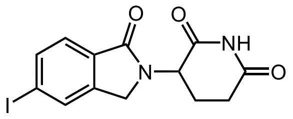

5b

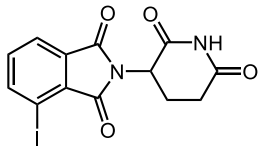

5c

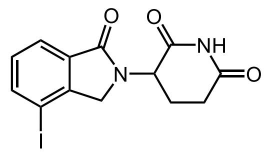

5d

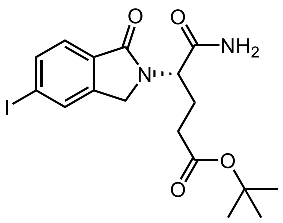

7a

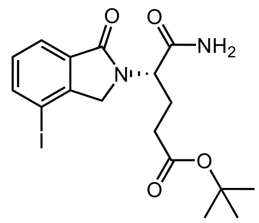

7b

Figure S1. Aryl iodides used in this study.

Section 2: Synthetic Procedures and Compound Characterization

Synthesis of Starting Materials

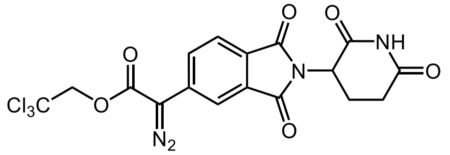

6a

2,2,2-trichloroethyl 2-diazo-2-(2-(2,6-dioxopiperidin-3-yl)-1,3-dioxoisoindolin-5-yl)acetate (6a). Compound **6a** was produced following a modified literature procedure.[3] Under inert atmosphere, a flame-dried round-bottom flask equipped with PTFE magnetic stir bar was charged with 2-(2,6-dioxopiperidin-3-yl)-5-iodoisoindoline-1,3-dione (1.33 g, 1.0 equiv, 3.46 mmol) (**5a**), triphenylphosphine (182 mg, 20 mol%, 0.69 mmol), silver (I) carbonate (477 mg, 50 mol%, 1.7 mmol), and $Pd(PPh_3)_4$ (400 mg, 10 mol%, 0.35 mmol). 2,2,2-trichloroethyl 2-diazoacetate (1.51 g, 2.0 equiv, 6.9 mmol), triethylamine (0.97 mL, 2.0 equiv, 6.9 mmol) and 19 mL dry DMF were charged via syringe. The reaction was stirred vigorously at room temperature for six hours, after which it was poured into saturated aqueous sodium chloride solution and filtered through Celite® with 200 mL ethyl acetate. The organic phase was separated and washed thrice with saturated aqueous sodium chloride solution, then dried over sodium sulfate. The solution was filtered and concentrated onto Celite®. The residue was purified via flash column chromatography (SiO_2, gradient of 20% to 60% ethyl acetate in hexanes with 3% v/v triethylamine). The obtained material was dissolved in acetone, then concentrated in vacuuo until a viscous red liquid was obtained. The solution was placed in a -20 °C freezer overnight. The precipitated yellow solid was collected by vacuum filtration, washing with minimal -20 °C acetone, which gave 2,2,2-trichloroethyl 2-diazo-2-(2-(2,6-dioxopiperidin-3-yl)-1,3-dioxoisoindolin-5-yl)acetate **(6a)** as an amorphous light yellow solid (655 mg, 1.38 mmol, 40% yield).

HRMS (APCI) *m/z*: $[M+H]^+$ calcd for $C_{17}H_{12}O_6N_4{}^{35}Cl_3$ 472.9817; Found 472.9818

^{1}H NMR (400 MHz, Acetone-*d6*) δ 9.93 (s, 1H), 8.19 – 8.17 (m, 1H), 8.03 (dd, *J* = 8.1, 1.8 Hz, 1H), 7.94 (dd, *J* = 8.0, 0.7 Hz, 1H), 5.16 (dd, *J* = 12.7, 5.4 Hz, 1H), 5.12 (s, 2H), 3.09 – 2.88 (m, 1H), 2.86 – 2.68 (m, 2H, partially obscured by residual water peak at 2.82), 2.31 – 2.19 (m, 1H).

^{13}C{^{1}H} NMR (151 MHz, Acetone-*d6*) δ 172.6, 169.9, 167.7, 167.6, 163.1, 134.2, 133.8, 129.4, 129.3, 124.7, 118.8, 96.0, 74.6, 66.5, 50.4, 32.0, 23.3.

FT-IR (film) ν_{max}/cm^{-1} 2100 (N=N).

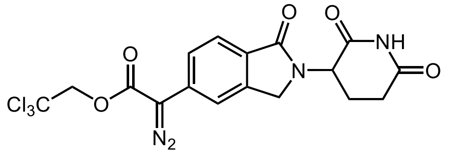

6b

2,2,2-trichloroethyl 2-diazo-2-(2-(2,6-dioxopiperidin-3-yl)-1-oxoisoindolin-5-yl)acetate (6b). Under inert atmosphere, a flame-dried round-bottom flask was charged with a PTFE magnetic stir bar, Compound **6b** was produced following a modified literature procedure.[3] 5-iodo-1-oxoisoindolin-2-yl)piperidine-2,6-dione (370 mg, 1.0 equiv, 1.0 mmol) (**5b**), triphenylphosphine (71 mg, 10 mol%, 0.10 mmol), silver(I) carbonate (138 mg, 50 mol%, 0.50 mmol), and $Pd(PPh_3)_4$ (58 mg, 5.0 mol%, 0.05 mmol). 2,2,2-trichloroethyl 2-diazoacetate (261 mg, 1.2 equiv, 1.2 mmol), triethylamine (0.21 mL, 1.5 equiv, 1.5 mmol), and dry DMSO (5.6 mL) were charged via syringe. After two hours of stirring at room temperature, the reaction was recharged with $Pd(PPh_3)_4$ (58 mg, 5.0 mol%, 0.05 mmol) and

triphenylphosphine (71 mg, 10 mol%, 0.10 mmol). After five hours of stirring at room temperature, the reaction was recharged with $Pd(PPh_3)_4$ (58 mg, 5.0 mol%, 0.05 mmol), triphenylphosphine (71 mg, 10 mol%, 0.10 mmol), and triethylamine (0.21 mL, 1.5 equiv, 1.5 mmol). The reaction was stirred for a further 12 hours at room temperature. The reaction was recharged with $Pd(PPh_3)_4$ (58 mg, 5.0 mol%, 0.05 mmol), triphenylphosphine (71 mg, 10 mol%, 0.10 mmol), triethylamine triethylamine (0.21 mL, 1.5 equiv, 1.5 mmol), silver(I) carbonate (138 mg, 50 mol%, 0.50 mmol) and 2,2,2-trichloroethyl 2-diazoacetate (261 mg, 1.2 equiv, 1.2 mmol), then stirred for a further two hours (total reaction time, 19 hours). The reaction was poured into 100 mL ethyl acetate with 1% v/v triethylamine and filtered through a layered plug of Celite®, silica gel, and neutral alumina. The solution was washed with 100 mL 10% w/w aqueous LiCl solution once, and twice with saturated aqueous NaCl solution. The organic layer was dried over sodium sulfate and filtered, then concentrated in vacuuo onto Celite®. The reaction was purified via flash column chromatography on neutral alumina using a gradient of 0-10% MeOH in CH_2Cl_2. The product-containing fractions were concentrated in vacuuo, and the obtained residue was sonicated with 4 mL acetone and stored in a -20 °C freezer overnight in the acetone. The yellow insoluble material was collected via vacuum filtration, rinsing with an additional 4 mL of acetone cooled to -20 °C, which gave 2,2,2-trichloroethyl 2-diazo-2-(2-(2,6-dioxopiperidin-3-yl)-1-oxoisoindolin-5-yl)acetate **(6b)** as a pale yellow amorphous solid (130 mg, 0.28 mmol, 28% yield).

HRMS (APCI): *m/z* $[M+H]^+$ calcd for $C_{17}H_{14}O_5N_4{}^{35}Cl_3$, 459.0024; Found 459.0021
^{1}H NMR (600 MHz, DMSO-*d*6): δ 10.99 (s, 1H), 7.82 (dd, *J* = 1.7, 0.8 Hz, 1H), 7.78 (dd, *J* = 8.1, 0.7 Hz, 1H), 7.69 (dd, *J* = 8.1, 1.7 Hz, 1H), 5.11 (dd, *J* = 13.3, 5.2 Hz, 1H, partially obscured by signal at 5.10), 5.10 (s, 2H) 4.48 (d, *J* = 17.4 Hz, 1H), 4.35 (d, *J* = 17.3 Hz, 1H), 2.91 (ddd, *J* = 17.4, 13.7, 5.4 Hz, 1H), 2.60 (ddd, *J* = 17.2, 4.4, 2.4 Hz, 1H), 2.40 (qd, *J* = 12.6, 4.5 Hz, 1H), 2.01 (dtd, *J* = 12.4, 5.3, 2.4 Hz, 1H).
^{13}C{^{1}H} NMR (151 MHz, DMSO-*d*6): δ 172.9, 171.0, 167.6, 162.5, 143.0, 129.4, 128.8, 123.6, 123.3, 118.4, 95.3, 73.2, 51.6, 47.2, 31.2, 30.7, 22.5.
FT-IR (film) ν_{max}/cm^{-1} 2103 (N=N).

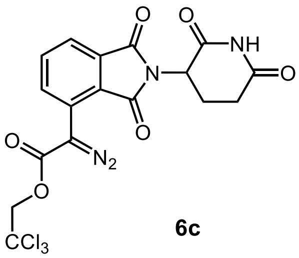

6c

2,2,2-trichloroethyl 2-diazo-2-(2-(2,6-dioxopiperidin-3-yl)-1,3-dioxoisoindolin-4-yl)acetate (6c). Compound **6c** was produced following a modified literature procedure.[3] Under inert atmosphere, a flame-dried round-bottom flask equipped with PTFE magnetic stir bar was charged with 2-(2,6-dioxopiperidin-3-yl)-4-iodoisoindoline-1,3-dione (768 mg, 1.0 equiv, 2.0 mmol) (**5c**), triphenylphosphine (105 mg, 20 mol%, 0.4 mmol), silver (I) carbonate (276 mg, 50 mol%, 1.0 mmol), and $Pd(PPh_3)_4$ (231 mg, 10 mol%, 0.20 mmol). 2,2,2-trichloroethyl 2-diazoacetate (870 mg, 2.0 equiv, 4.0 mmol), triethylamine (0.56 mL, 2.0 equiv, 4.0 mmol) and 11 mL dry DMF were charged via syringe. The reaction was stirred vigorously at room temperature for six hours, after which it was poured into saturated aqueous sodium chloride solution and filtered through Celite® with 100 mL ethyl acetate. The organic phase was separated and washed thrice with saturated aqueous sodium chloride solution, then dried over sodium sulfate. The solution was filtered and concentrated onto Celite®. The residue was purified via flash column chromatography (SiO_2, gradient of 20% to 60% ethyl acetate in hexanes with 3% v/v triethylamine) and further purified via flash column chromatography (SiO_2, gradient of 0% to 5% acetone in CH_2Cl_2 with 3% v/v triethylamine). The obtained material was further purified via reverse phase flash column chromatography (C18, gradient of 20% to 90% EtOH in H_2O with 10 mM NH_4OAc buffer), which gave

2,2,2-trichloroethyl 2-diazo-2-(2-(2,6-dioxopiperidin-3-yl)-1,3-dioxoisoindolin-4-yl)acetate **(6c)** as an amorphous light yellow solid (145 mg, 0.30 mmol, 15% yield).

HRMS (ESI) *m/z*: $[M+Na]^+$ calcd for $C_{17}H_{11}O_6N_4{}^{35}Cl_3{}^{23}Na$ 494.9636; Found 494.9640

^{1}H NMR (400 MHz, Acetone-*d6*) δ 9.94 (s, 1H), 8.13 (dd, *J* = 8.0, 1.0 Hz, 1H), 7.94 (dd, *J* = 8.1, 7.3 Hz, 1H), 7.87 (dd, *J* = 7.4, 1.0 Hz, 1H), 5.19 (dd, *J* = 12.6, 5.4 Hz, 1H), 5.07 (s, 2H), 3.07 – 2.91 (m, 1H), 2.86 – 2.70 (m, 2H), 2.30 – 2.20 (m, 1H).

^{13}C{^{1}H} NMR (151 MHz, Acetone-*d6*) δ 206.1, 172.5, 169.8, 167.6, 167.4, 163.7, 135.9, 135.3, 133.7, 126.3, 125.3, 123.1, 96.1, 74.7, 50.4, 31.9, 23.2.

FT-IR (film) ν_{max}/cm^{-1} 2100 (N=N).

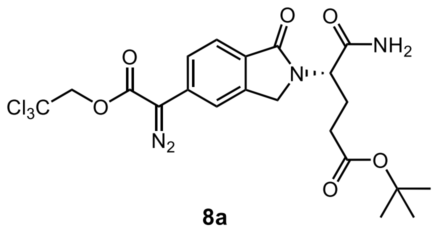

8a

***tert*-butyl (*S*)-5-amino-4-(5-(1-diazo-2-oxo-2-(2,2,2-trichloroethoxy)ethyl)-1-oxoisoindolin-2-yl)-5-oxopentanoate (8a).**

Compound **8a** was produced following a modified literature procedure.[3] Under inert atmosphere, a flame-dried 16 mL vial equipped with PTFE magnetic stir bar was charged with *tert*-butyl (*S*)-5-amino-4-(5-iodo-1-oxoisoindolin-2-yl)-5-oxopentanoate (**7a**) (89 mg, 1.0 equiv, 0.20 mmol), triphenylphosphine (28 mg, 20 mol%, 0.04 mmol), silver (I) carbonate (28 mg, 50 mol%, 0.10 mmol), and $Pd(PPh_3)_4$ (23 mg, 10 mol%, 0.02 mmol). 2,2,2-trichloroethyl 2-diazoacetate (65 mg, 1.5 equiv, 0.30 mmol), triethylamine (42 mL, 1.5 equiv, 0.30 mmol) and 4.0 mL dry toluene were charged via syringe. The reaction was placed in a preheated aluminum heating block (40 °C) and stirred vigorously at this temperature for four hours. The reaction was then cooled, rinsed through a silica plug with 20 mL ethyl acetate, and concentrated in vacuuo. The crude residue was mounted into Celite® and purified by flash column chromatography (SiO_2, gradient of 20% to 90% ethyl acetate in hexanes with 1% v/v triethylamine). The obtained material was further purified by reverse-phase flash column chromatography (C18, 20-90% acetonitrile in H_2O, with 10 mM NH_4OAc as buffer), which afforded *tert*-butyl (*S*)-5-amino-4-(5-(1-diazo-2-oxo-2-(2,2,2-trichloroethoxy)ethyl)-1-oxoisoindolin-2-yl)-5-oxopentanoate (**8a**) as an amorphous light yellow solid (63 mg, 0.12 mmol, 59% yield).

HRMS (APCI) *m/z*: $[M+H]^+$ calcd for $C_{21}H_{24}O_6N_4{}^{35}Cl_3$ 533.0756; Found 533.0759.

^{1}H NMR (400 MHz, $CDCl_3$) δ 7.86 (d, *J* = 8.0 Hz, 1H), 7.79 (d, *J* = 1.7 Hz, 1H), 7.48 (dd, *J* = 8.1, 1.7 Hz, 1H), 6.24 (s, 1H), 5.30 (s, 1H), 4.93 (s, 2H), 4.89 (dd, *J* = 8.7, 6.3 Hz, 1H), 4.55 (d, *J* = 17.2 Hz, 1H), 4.46 (d, *J* = 17.2 Hz, 1H), 2.50 – 2.08 (m, 4H), 1.42 (s, 9H).

^{13}C{^{1}H} NMR (101 MHz, $CDCl_3$) δ 171.8, 168.8, 142.9, 129.8, 129.4, 124.5, 123.1, 118.4, 94.9, 81.0, 74.1, 54.1, 47.3, 32.0, 28.2, 24.4.

FT-IR (film) ν_{max}/cm^{-1} 2099 (N=N).

Specific Rotation: $[\alpha]_D^{22}$ -55.2 (c 1.0, $CHCl_3$).

Note: *The degree of enantioenrichment of the stereogenic center was not measured by SFC analysis due to the instability of the compound under the conditions of analysis.*

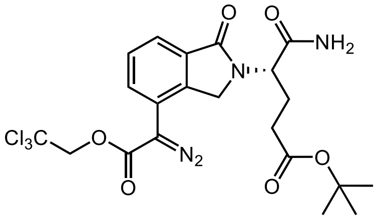

8b

***tert*-butyl (*S*)-5-amino-4-(4-(1-diazo-2-oxo-2-(2,2,2-trichloroethoxy)ethyl)-1-oxoisoindolin-2-yl)-5-oxopentanoate (8b).**

Compound **1b** was produced following a modified literature procedure.[3] Under inert atmosphere, a flame-dried 16 mL vial equipped with PTFE magnetic stir bar was charged with *tert*-butyl (*S*)-5-amino-4-(4-iodo-1-oxoisoindolin-2-yl)-5-oxopentanoate (**7b**) (89 mg, 1.0 equiv, 0.20 mmol), triphenylphosphine (28 mg, 20 mol%, 0.04 mmol), silver (I) carbonate (28 mg, 50 mol%, 0.10 mmol), and $Pd(PPh_3)_4$ (23 mg, 10 mol%, 0.02 mmol). 2,2,2-trichloroethyl 2-diazoacetate (65 mg, 1.5 equiv, 0.30 mmol), triethylamine (42 mL, 1.5 equiv, 0.30 mmol) and 4.0 mL dry toluene were charged via syringe. The reaction was placed in a preheated aluminum heating block (40 °C) and stirred vigorously at this temperature for four hours. The reaction was then cooled, rinsed through a silica plug with 20 mL ethyl acetate, and concentrated in vacuuo. The crude residue was mounted into Celite® and purified by flash column chromatography (SiO_2, gradient of 20% to 90% ethyl acetate in hexanes with 1% v/v triethylamine). The obtained material was further purified by reverse-phase flash column chromatography (C18, 20-90% acetonitrile in H_2O, with 10 mM NH_4OAc as buffer), which afforded *tert*-butyl (*S*)-5-amino-4-(4-(1-diazo-2-oxo-2-(2,2,2-trichloroethoxy)ethyl)-1-oxoisoindolin-2-yl)-5-oxopentanoate **(8b)** as an amorphous light yellow solid (33 mg, 0.06 mmol, 31% yield).

HRMS (ESI) *m/z*: $[M+H]^+$ calcd for $C_{21}H_{24}O_6N_4{}^{35}Cl_3$ 533.0763; Found 533.0756.

^{1}H NMR (400 MHz, $CDCl_3$) δ 7.83 (dd, *J* = 7.5, 1.2 Hz, 1H), 7.64 (dd, *J* = 7.8, 1.1 Hz, 1H), 7.57 (t, *J* = 7.6 Hz, 1H), 6.32 (s, 1H), 5.35 (s, 1H), 4.97 – 4.89 (m, 3H), 4.55 (d, *J* = 17.5 Hz, 1H), 4.45 (d, *J* = 17.5 Hz, 1H), 2.46 – 2.21 (m, 3H), 2.20 – 2.09 (m, 1H), 1.42 (s, 9H).

^{13}C{^{1}H} NMR: (151 MHz, $CDCl_3$) δ 171.9, 171.6, 168.7, 140.0, 133.3, 129.3, 123.9, 120.5, 95.0, 81.1, 74.3, 54.2, 47.0, 32.0, 28.2, 24.4.

FT-IR (film) ν_{max}/cm^{-1} 2099 (N=N).

Specific Rotation: $[\alpha]_D^{22}$ -43.2 (*c* 0.5, $CHCl_3$).

Note: *The degree of enantioenrichment of the stereogenic center was not measured by SFC analysis due to the instability of the compound under the conditions of analysis.*

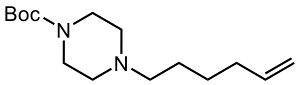

23

***tert*-butyl 4-(hex-5-en-1-yl)piperazine-1-carboxylate (23).**

Under inert atmosphere, a flame-dried round-bottom flask fitted with reflux condenser was charged with sodium iodide (75 mg, 10 mol%, 0.50 mmol), tert-butyl piperazine-1-carboxylate (0.93 g, 1.0 equiv, 5.0 mmol), and potassium carbonate (1.5 g, 2.2 equiv, 11 mmol). 6-bromohex-1-ene (0.90 g, 1.1 equiv, 5.5 mmol) and 23 mL dry acetonitrile were charged by syringe. The reaction was stirred overnight at reflux then cooled, filtered through Celite® with CH_2Cl_2, and concentrated in vacuuo. The crude residue was purified via flash column chromatography (SiO_2, gradient of 0-10% methanol in CH_2Cl_2), which produced tert-butyl 4-(hex-5-en-1-yl)piperazine-1-carboxylate (**23**) (1.23 g, 92% yield, 4.6 mmol) as a translucent yellow oil.

HRMS (APCI) *m/z*: $[M+H]^+$ calcd for $C_{15}H_{29}O_2N$ 269.2224; Found 269.2223.
^{1}H NMR (400 MHz, $CDCl_3$): δ 5.79 (ddt, *J* = 16.9, 10.2, 6.7 Hz, 1H), 5.00 (dq, *J* = 17.1, 1.7 Hz, 1H), 4.94 (ddt, *J* = 10.2, 2.2, 1.2 Hz, 1H), 3.42 (t, *J* = 5.1 Hz, 4H), 2.47 – 2.27 (m, 6H), 2.06 (qt, *J* = 7.1, 1.4 Hz, 2H), 1.55 – 1.35 (m, 13H).

^{13}C{^{1}H} NMR (151 MHz, $CDCl_3$) δ 154.9, 138.8, 114.7, 79.7, 58.7, 53.2, 33.8, 28.6, 26.9, 26.4.

Synthesis of Final Compounds

General Procedure 1 (GP1)
A flame-dried 4 mL vial under inert atmosphere was equipped with ca. 200 wt% 4Å mol. sieves and a PTFE magnetic stir bar. The vial was charged with diazo (1.0 equiv, 0.10 mmol) and 1,1,1,3,3,3-hexafluoropropan-2-ol (0.10 mL, 10.0 equiv, 1.0 mmol). 1.0 mL substrate was charged via syringe, and a 20 mM solution of Rh_2(*S*-*tetra*-*p*-BrPhPTTL)$_4$ (50 µL, 1 mol%, 1.0 µmol) in dry CH_2Cl_2 was charged via syringe at room temperature to the stirred reaction. The reaction was allowed to stir at r.t. for 0.5 h and was filtered through Celite®, then concentrated in vacuuo, removing excess substrate by Kugelrohr distillation if possible. The reaction was dry-mounted into Celite® and purified by flash column chromatography.

General Procedure 2 (GP2)
A flame-dried 4 mL vial under inert atmosphere was equipped with ca. 200 wt% 4Å mol. sieves and a PTFE magnetic stir bar. The vial was charged with diazo (1.0 equiv, 0.10 mmol) and 1,1,1,3,3,3-hexafluoropropan-2-ol (0.10 mL, 10.0 equiv, 1.0 mmol). 0.5 mL substrate and 0.5 mL CH_2Cl_2 were charged via syringe, and a 20 mM solution of Rh_2(*S*-*tetra*-*p*-BrPhPTTL)$_4$ (50 µL, 1 mol%, 1.0 µmol) in dry CH_2Cl_2 was charged via syringe at room temperature to the stirred reaction. The reaction was allowed to stir at r.t. for 0.5 h and was filtered through Celite®, then concentrated in vacuuo, removing excess substrate by Kugelrohr distillation if possible. The reaction was dry-mounted into Celite® and purified by flash column chromatography.

General Procedure 3 (GP3)
A flame-dried 4 mL vial equipped with 200 w/w% 4 Å mol. sieves and PTFE magnetic stir bar was charged with Rh_2(*S*-*tetra*-*p*-BrPhPTTL)$_4$ (1 mol%, 1.0 µmol) and substrate (10.0 equiv, 1.0 mmol) under inert atmosphere. 0.5 mL dry CH_2Cl_2 was charged by syringe. A solution of diazo (1.0 equiv, 0.10 mmol) in 0.5 mL dry CH_2Cl_2 and 1,1,1,3,3,3-hexafluoropropan-2-ol (0.10 mL, 10.0 equiv, 1.0 mmol) was prepared under inert atmosphere in a flame-dried vial. The diazoacetate solution was added over the course of 1 hour using a syringe pump to the stirred reaction vial at room temperature. The reaction was stirred for 0.5 h further, after which the reaction was filtered through a pad of Celite®, rinsing with CH_2Cl_2. The solution was concentrated in vacuuo. If possible, remaining substrate was removed via Kugelrohr distillation. The crude residue was analyzed by ^{1}H NMR. The crude material was dry-mounted on Celite® and purified by flash column chromatography.

General Procedure 4 (GP4)
A flame-dried 4 mL vial equipped with 200 w/w% 4 Å mol. sieves and PTFE magnetic stir bar was charged with Rh_2(*S*-*tetra*-*p*-BrPhPTTL)$_4$ (1 mol%, 1.0 µmol) and substrate (10.0 equiv, 1.0 mmol) under inert atmosphere. 0.5 mL dry CH_2Cl_2 and 1,1,1,3,3,3-hexafluoropropan-2-ol (0.10 mL, 10.0 equiv, 1.0 mmol) were charged by syringe. A solution of diazo (1.0 equiv, 0.10 mmol) in 0.5 mL dry CH_2Cl_2 was prepared under inert atmosphere in a flame-dried vial. The diazoacetate solution was added over the course of 3 hours using a syringe pump to the stirred reaction vial at room temperature. The reaction was stirred for 0.5 h further, after which the reaction was filtered through a pad of Celite®, rinsing with CH_2Cl_2. The solution was concentrated in vacuuo. If possible, remaining substrate was removed via Kugelrohr distillation. The crude residue was analyzed by ^{1}H NMR. The crude material was dry-mounted on Celite® and purified by flash column chromatography.

General Procedure 5 (GP5)

A flame-dried 4 mL vial equipped with 200 w/w% 4 Å mol. sieves and PTFE magnetic stir bar was charged with Rh_2(*S-tetra-p*-BrPhPTTL)$_4$ (1 mol%, 1.0 µmol) and substrate (5.0 equiv, 0.5 mmol) under inert atmosphere. 0.5 mL dry CH_2Cl_2 was charged by syringe. A solution of diazo (1.0 equiv, 0.10 mmol) in 0.5 mL dry CH_2Cl_2 and 1,1,1,3,3,3-hexafluoropropan-2-ol (0.10 mL, 10.0 equiv, 1.0 mmol) was prepared under inert atmosphere in a flame-dried vial. The diazoacetate solution was added over the course of 0.25 h using a syringe pump to the stirred reaction vial at room temperature. The reaction was stirred for 0.5 h further, after which the reaction was filtered through a pad of Celite®, rinsing with CH_2Cl_2. The solution was concentrated in vacuuo. The crude residue was analyzed by ^{1}H NMR. The crude material was dry-mounted on Celite® and purified by flash column chromatography.

General Procedure 6 (GP6)

A flame-dried 4 mL vial equipped with 200 w/w% 4 Å mol. sieves and PTFE magnetic stir bar was charged with Rh_2(*S-tetra-p*-BrPhPTTL)$_4$ (1 mol%, 1.0 µmol) and substrate (5.0 equiv, 0.5 mmol) under inert atmosphere. 0.5 mL dry CH_2Cl_2 and 1,1,1,3,3,3-hexafluoropropan-2-ol (0.10 mL, 10.0 equiv, 1.0 mmol) were charged by syringe. A solution of diazo (1.0 equiv, 0.10 mmol) in 0.5 mL dry CH_2Cl_2 was prepared under inert atmosphere in a flame-dried vial. The diazoacetate solution was added over the course of 0.25 h using a syringe pump to the stirred reaction vial at room temperature. The reaction was stirred for 0.5 h further, after which the reaction was filtered through a pad of Celite®, rinsing with CH_2Cl_2. The solution was concentrated in vacuuo. The crude residue was analyzed by ^{1}H NMR. The crude material was dry-mounted on Celite® and purified by flash column chromatography.

General Procedure 7 (GP7)

Under inert atmosphere, a flame-dried vial equipped with PTFE magnetic stir bar was charged with ring-opened product (1.0 equiv). Dry acetonitrile (0.2 M) was charged via syringe. Benzenesulfonic acid (2.0 equiv) was added to the reaction mixture neat, and the reaction was placed in a preheated aluminum heating block (70 °C). The reaction was stirred at this temperature for 3.5 h, after which the reaction was allowed to cool, and was filtered through a short plug of silica with 4 mL ethyl acetate. The reaction was concentrated in vacuuo, dry mounted to Celite®, and purified via flash column chromatography (SiO_2) to afford the product.

General Procedure 8 (GP8)

A flame-dried 4 mL vial under inert atmosphere was equipped with ca. 200 wt% 4Å mol. sieves and a PTFE magnetic stir bar. The vial was charged with diazo (1.0 equiv, 0.10 mmol) and substrate (10.0 equiv, 1.0 mmol). 1.0 mL dry CH_2Cl_2 was charged via syringe, and a 20 mM solution of Rh_2(*S-tetra-p*-BrPhPTTL)$_4$ (50 µL, 1 mol%, 1.0 µmol) in dry CH_2Cl_2 was charged via syringe at room temperature to the stirred reaction. The reaction was allowed to stir at r.t. for 0.5 h and was filtered through Celite®, then concentrated in vacuuo, removing excess substrate by Kugelrohr distillation if possible. The reaction was dry-mounted into Celite® and purified by flash column chromatography.

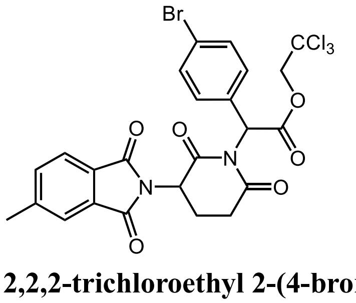

2,2,2-trichloroethyl 2-(4-bromophenyl)-2-(3-(5-methyl-1,3-dioxoisoindolin-2-yl)-2,6-dioxopiperidin-1-yl)acetate (4).

2-(2,6-dioxopiperidin-3-yl)-5-methylisoindoline-1,3-dione (54 mg, 1.0 equiv, 0.20 mmol) and Rh_2(*S*-p-Ph-TPCP)$_4$ (3.5 mg, 1 mol%, 2.0 µmol) were charged to an oven-dried vial under inert atmosphere along

with ~200% w/w 4Å molecular sieves. The vial was charged with 1.0 mL dry CH_2Cl_2 via syringe. A solution of 2,2,2-trichloroethyl 2-(4-bromophenyl)-2-diazoacetate (82 mg, 1.1 Eq, 0.22 mmol) was prepared under inert atmosphere in 1.0 mL dry CH_2Cl_2 and added to the stirred reaction at room temperature over the course of thirty minutes, using a syringe pump. The reaction was allowed to stir for 17 hours following addition, after which the reaction was dry-mounted onto Celite® and purified by flash column chromatography (SiO_2, 1:1 ethyl acetate:hexanes), which produced 2,2,2-trichloroethyl 2-(4-bromophenyl)-2-(3-(5-methyl-1,3-dioxoisoindolin-2-yl)-2,6-dioxopiperidin-1-yl)acetate as an amorphous white solid (43 mg, 70 μmol, 35% yield).

HRMS (APCI) *m/z*: $[M+H]^+$ calcd for $C_{24}H_{19}O_6N_2{}^{79}Br^{35}Cl_3$ 614.9487; Found 614.9488

^{1}H NMR (500 MHz, $CDCl_3$, reported as mixture of diastereomers) 7.75 – 7.69 (m, 1H), 7.67 – 7.63 (m, 1H), 7.561 and 7.556 (d, *J* = 8.6 Hz, 1H), 7.53 – 7.50 (m, 2H), 7.431 and 7.427 (d, *J* = 8.6 Hz, 1H), 6.45 and 6.41 (s, 1H), 4.92 and 4.86 (dd, *J* = 13.3, 5.7 Hz, 1H, partially obscured by signals at 4.904 and 4.902), 4.904 and 4.902 (d, *J* = 11.9 Hz, 1H, partially obscured by signal at 4.92), 4.67 and 4.60 (d, *J* = 11.9 Hz), 3.09 – 2.72 (m, 3H), 2.51 (s, 3H), 2.27 – 2.13 (m, 1H).

^{13}C{^{1}H} NMR (151 MHz, $CDCl_3$, reported as a mixture of diastereomers**)** δ 179.2, 179.1, 175.6, 175.4, 167.9, 167.84, 167.75, 167.71, 166.5, 166.3, 145.7, 134.9, 132.4, 132.3, 132.3, 131.7, 131.5, 129.7, 129.6, 129.5, 124.29, 124.27, 123.7, 94.28, 94.26, 76.02, 76.98, 74.7, 74.6, 49.93, 49.85, 27.7, 27.6, 23.0, 22.9, 22.2.

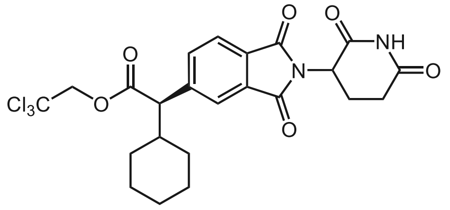

9a

2,2,2-trichloroethyl (2*R*)-2-cyclohexyl-2-(2-(2,6-dioxopiperidin-3-yl)-1,3-dioxoisoindolin-5-yl)acetate (9a).

Compound **9a** was produced via **GP1** using **6a** (47 mg, 1.0 equiv, 0.10 mmol), 1,1,1,3,3,3-hexafluoroisopropanol (0.11 mL, 10.0 equiv, 1.00 mmol), Rh_2(*S*-*tetra*-*p*-BrPhPTTL)$_4$ (20 mM in CH_2Cl_2, 50 μL, 1 mol%, 1.0 μmol), and cyclohexane (1.0 mL). Purification via flash column chromatography (SiO_2, 20% to 50% ethyl acetate in hexanes) produced 2,2,2-trichloroethyl (2*R*)-2-cyclohexyl-2-(2-(2,6-dioxopiperidin-3-yl)-1,3-dioxoisoindolin-5-yl)acetate **(9a)** as an amorphous white solid (47 mg, 89 μmol, 89% yield).

Compound **9a** was produced via **GP2** using **6a** (47 mg, 1.0 equiv, 0.10 mmol), 1,1,1,3,3,3-hexafluoroisopropanol (0.11 mL, 10.0 equiv, 1.00 mmol), Rh_2(*S*-*tetra*-*p*-BrPhPTTL)$_4$ (20 mM in CH_2Cl_2, 50 μL, 1 mol%, 1.0 μmol), CH_2Cl_2 (0.5 mL), and cyclohexane (0.5 mL). Purification via flash column chromatography (SiO_2, 20% to 50% ethyl acetate in hexanes) produced 2,2,2-trichloroethyl (2*R*)-2-cyclohexyl-2-(2-(2,6-dioxopiperidin-3-yl)-1,3-dioxoisoindolin-5-yl)acetate **(9a)** as an amorphous white solid (47 mg, 89 μmol, 89% yield).

Compound **9a** was prepared via **GP3** using **6a** (47 mg, 1.0 equiv, 0.10 mmol), Rh_2(*S*-*tetra*-p-Br-PPTTL)$_4$ (3.7 mg, 1 mol%, 1.0 μmol), 2,2,2-trichloroethyl 2-diazo-2-(2-(2,6-dioxopiperidin-3-yl)-1,3-dioxoisoindolin-5-yl)acetate (**1c**) (47 mg, 1.0 equiv, 0.10 mmol), 1,1,1,3,3,3-hexafluoropropan-2-ol (0.10 mL, 10.0 equiv, 1.0 mmol), and 1.0 mL CH_2Cl_2 at room temperature with a one hour slow addition of diazo and 0.5 h additional stirring. Purification via flash column chromatography (SiO_2, gradient of 20%

to 50% EtOAc in hexanes) afforded 2,2,2-trichloroethyl (2*R*)-2-cyclohexyl-2-(2-(2,6-dioxopiperidin-3-yl)-1,3-dioxoisoindolin-5-yl)acetate **(9a)** as an amorphous white solid (47 mg, 89 μmol, 89% yield).

HRMS (APCI) *m/z*: $[M+H]^+$ calcd for $C_{23}H_{24}O_6N_2{}^{35}Cl_3$ 529.0695; Found 529.0697
^{1}H NMR (400 MHz, $CDCl_3$, reported as a mixture of diastereomers) δ 8.09 (s, 1H), 7.94 (d, *J* = 1.6 Hz, 1H), 7.84 (d, *J* = 7.7 Hz, 1H), 7.76 (dd, *J* = 7.8, 1.7 Hz, 1H), 4.99 (dd, *J* = 12.5, 5.3 Hz, 1H), 4.79 (d, *J* = 11.9 Hz, 1H), 4.65 (d, *J* = 12.0 Hz, 1H), 3.58 (d, *J* = 10.5 Hz, 1H), 3.01 – 2.87 (m, 1H), 2.87 – 2.68 (m, 2H), 2.26 – 2.09 (m, 2H), 1.99 – 1.83 (m, 1H), 1.82 – 1.73 (m, 1H), 1.69 – 1.61 (m, 2H), 1.41 – 1.29 (m, 2H), 1.22 – 1.08 (m, 3H), 0.94 – 0.75 (m, 1H).
^{13}C{^{1}H} NMR (151 MHz, $CDCl_3$, reported as a mixture of diastereomers) δ 171.2, 171.0, 168.2, 167.1, 167.0, 144.7, 135.2, 132.3, 131.0, 124.27, 124.25, 124.1, 94.7, 74.4, 58.9, 49.5, 41.4, 31.8, 31.5, 30.4, 26.1, 25.9, 25.8, 22.7.
SFC analysis: Using **GP1**, **9a** (Trefoil® AMY1, 30% 1:1 MeOH:^{i}PrOH with 0.2% formic acid in CO_2, 2.5 mL/min, 210 nm) indicated 98:2 d.r. for asymmetric induction observed at the newly formed stereogenic center: t_R (major diastereomers) = 2.43 and 6.85 min., t_R (minor diastereomers) = 2.2 and 5.4 min. Using **GP2**, **9a** indicated 99:1 d.r. Using **GP3**, **9a** indicated 99:1 d.r.

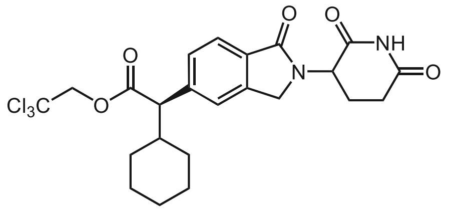

9b

2,2,2-trichloroethyl (2*R*)-2-cyclohexyl-2-(2-(2,6-dioxopiperidin-3-yl)-1-oxoisoindolin-5-yl)acetate (9b).

Compound **9b** was produced via **GP1** using **6b** (46 mg, 1.0 equiv, 0.10 mmol), 1,1,1,3,3,3-hexafluoroisopropanol (0.11 mL, 10.0 equiv, 1.00 mmol), Rh_2(*S*-*tetra*-*p*-BrPhPTTL)$_4$ (20 mM in CH_2Cl_2, 50 μL, 1 mol%, 1.0 μmol), and cyclohexane (1.0 mL). Purification via flash column chromatography (SiO_2, 40% to 100% ethyl acetate in hexanes) produced 2,2,2-trichloroethyl (2*R*)-2-cyclohexyl-2-(2-(2,6-dioxopiperidin-3-yl)-1-oxoisoindolin-5-yl)acetate **(9b)** as an amorphous white solid (42 mg, 82 μmol, 82% yield).

Compound **9b** was prepared via **GP3** using **6b** (46 mg, 1.0 equiv, 0.10 mmol), Rh_2(*S*-*tetra*-*p*-BrPhPTTL)$_4$ (3.7 mg, 1 mol%, μ.0 mmol), 1,1,1,3,3,3-hexafluoropropan-2-ol (0.10 mL, 10.0 equiv, 1.0 mmol), and 1.0 mL CH_2Cl_2 at room temperature with a one hour slow addition of diazo and 0.5 h additional stirring. Purification via flash column chromatography (SiO_2, gradient of 40% to 100% EtOAc in hexanes) afforded 2,2,2-trichloroethyl (2*R*)-2-cyclohexyl-2-(2-(2,6-dioxopiperidin-3-yl)-1-oxoisoindolin-5-yl)acetate **(9b)** as an amorphous white solid (24 mg, 46 μmol, 46% yield).

HRMS (APCI) *m/z*: $[M+H]^+$ calcd for $C_{23}H_{26}O_5N_2{}^{35}Cl_3$ 515.0902; Found 515.0902
^{1}H NMR (400 MHz, $CDCl_3$, reported as a mixture of diastereomers) δ 8.12 and 8.09 (s, 1H), 7.84 (d, *J* = 7.9 Hz, 1H), 7.53 and 7.50 (s, 1H), 7.47 (d, *J* = 8.6 Hz, 1H), 5.23 and 5.22 (dd, *J* = 13.4, 5.1 Hz, 1H), 4.799 and 4.796 (d, *J* = 12.0 Hz, 1H), 4.62 and 4.61 (d, *J* = 11.9 Hz, 1H), 4.50 and 4.48 (d, *J* = 16.1 Hz, 1H), 4.35 and 4.32 (d, *J* = 16.0 Hz, 1H), 3.51 and 3.50 (d, *J* = 10.6 Hz, 1H), 3.01 – 2.76 (m, 2H), 2.45 – 2.28 (m, 1H), 2.28 – 2.16 (m, 1H), 2.15 – 2.06 (m, 1H), 1.94 – 1.85 (m, 1H), 1.81 – 1.73 (m, 1H), 1.68 – 1.54 (m, 2H), 1.39 – 1.04 (m, 5H), 0.88 – 0.74 (m, 1H).
^{13}C{^{1}H} NMR (151 MHz, $CDCl_3$, reported as a mixture of diastereomers) δ 171.8, 171.7, 171.2, 169.69, 169.67, 169.1, 142.0, 141.7, 131.0, 129.5, 129.3, 124.40, 124.37, 123.3, 123.1, 94.8, 74.3, 59.0, 58.9, 52.0, 51.9, 47.09, 47.06, 32.0, 31.7, 30.5, 26.2, 26.0, 25.9, 23.56, 23.55.

SFC analysis: Using **GP1**, **9b** (CHIRALCEL® OZ-3, 20% 1:1 MeOH:^{i}PrOH with 0.2% formic acid in CO_2, 2.5 mL/min, 210 nm) indicated 97:3 d.r. for asymmetric induction observed at the newly formed stereogenic center: t_R (major diastereomers) = 6.7 and 15.8 min., t_R (minor diastereomers) = 7.2 and 15.0 min. Using **GP3**, **9a** indicated 98:2 d.r.

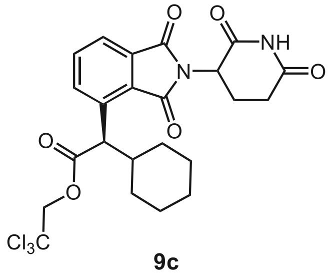

9c

2,2,2-trichloroethyl (2*R*)-2-cyclohexyl-2-(2-(2,6-dioxopiperidin-3-yl)-1,3-dioxoisoindolin-4-yl)acetate (9c).

Compound **9c** was produced via **GP1** using **6c** (47 mg, 1.0 equiv, 0.10 mmol), 1,1,1,3,3,3-hexafluoroisopropanol (0.11 mL, 10.0 equiv, 1.00 mmol), Rh_2(*S*-*tetra*-*p*-BrPhPTTL)$_4$ (20 mM in CH_2Cl_2, 50 µL, 1 mol%, 1.0 µmol), and cyclohexane (1.0 mL). Purification via flash column chromatography (SiO_2, gradient of 20% to 50% EtOAc in hexanes) afforded 2,2,2-trichloroethyl (2*R*)-2-cyclohexyl-2-(2-(2,6-dioxopiperidin-3-yl)-1,3-dioxoisoindolin-4-yl)acetate **(9c)** as an amorphous white solid (32 mg, 61 µmol, 61% yield).

Compound **9c** was prepared via **GP3** using cyclohexane (0.11 mL, 10.0 equiv, 1.0 mmol), Rh_2(*S*-*tetra*-*p*-BrPhPTTL)$_4$ (3.7 mg, 1 mol%, 1.0 µmol), **6c** (47 mg, 1.0 equiv, 0.10 mmol), 1,1,1,3,3,3-hexafluoropropan-2-ol (0.10 mL, 10.0 equiv, 1.0 mmol), and 1.0 mL CH_2Cl_2 at room temperature with a one hour slow addition of diazo and 0.5 h additional stirring. Purification via flash column chromatography (SiO_2, gradient of 20% to 50% EtOAc in hexanes) afforded 2,2,2-trichloroethyl (2*R*)-2-cyclohexyl-2-(2-(2,6-dioxopiperidin-3-yl)-1,3-dioxoisoindolin-4-yl)acetate **(9c)** as an amorphous white solid (25 mg, 47 µmol, 47% yield).

HRMS (APCI) *m/z*: $[M+H]^+$ calcd for $C_{23}H_{24}O_6N_2{}^{35}Cl_3$ 529.0695; Found 529.0700
^{1}H NMR (600 MHz, $CDCl_3$, reported as a mixture of diastereomers) δ 8.10 (s, 1H), 7.95 (d, *J* = 7.9 Hz, 1H), 7.80 (ddd, *J* = 7.4, 2.9, 1.0 Hz, 1H), 7.72 (ddd, *J* = 8.0, 7.3, 1.0 Hz, 1H), 5.00 (d, *J* = 10.2 Hz, 1H, overlapping with dd at 4.99), 4.99 (dd, *J* = 15.7, 4.8 Hz, 1H, overlapping with d at 5.00), 4.76 – 4.59 (m, 2H), 2.94 – 2.88 (m, 1H), 2.87 – 2.69 (m, 2H), 2.22 – 2.12 (m, 2H), 1.97 – 1.90 (m, 1H), 1.82 – 1.74 (m, 1H), 1.68 – 1.61 (m, 2H), 1.37 – 1.28 (m, 2H), 1.22 – 1.12 (m, 3H), 1.01 – 0.91 (m, 1H).
^{13}C{^{1}H} NMR (151 MHz, $CDCl_3$, reported as a mixture of diastereomers) δ 171.11, 171.08, 170.9, 167.93, 167.90, 167.8, 167.7, 166.8, 137.6, 134.8, 134.7, 134.59, 134.57, 132.1, 129.2, 129.1, 123.99, 123.97, 94.82, 94.81, 74.2, 50.4, 50.3, 49.41, 49.37, 41.3, 41.2, 31.68, 31.66, 31.51, 31.49, 30.1, 30.0, 26.1, 26.00, 25.98, 22.77, 22.76.
SFC analysis: Using **GP1**, **9c** (CHIRALPAK® AS-3, 15% 1:1 MeOH:^{i}PrOH with 0.2% formic acid in CO_2, 2.5 mL/min, 210 nm) indicated 73:27 d.r. for asymmetric induction observed at the newly formed stereogenic center: t_R (major diastereomers) = 2.5 and 3.1 min., t_R (minor diastereomers) = 1.6 and 1.8 min. Using **GP3**, **9c** indicated 76:24 d.r.

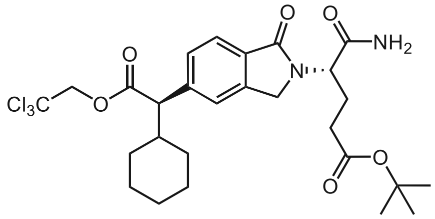

(S,R)-10a

tert-butyl (S)-5-amino-4-(5-((R)-1-cyclohexyl-2-oxo-2-(2,2,2-trichloroethoxy)ethyl)-1-oxoisoindolin-2-yl)-5-oxopentanoate ((S,R)-10a).

Compound **(*S,R*)-10a** was produced via **GP1** using **8a** (53 mg, 1.0 equiv, 0.10 mmol), 1,1,1,3,3,3-hexafluoroisopropanol (0.11 mL, 10.0 equiv, 1.00 mmol), Rh_2(*S-tetra-p*-BrPhPTTL)$_4$ (20 mM in CH_2Cl_2, 50 µL, 1 mol%, 1.0 µmol), and cyclohexane (1.0 mL). Purification via flash column chromatography (SiO_2, 40% to 100% ethyl acetate in hexanes) produced *tert*-butyl (*S*)-5-amino-4-(5-((*R*)-1-cyclohexyl-2-oxo-2-(2,2,2-trichloroethoxy)ethyl)-1-oxoisoindolin-2-yl)-5-oxopentanoate (**(*S,R*)-10a**) as an amorphous white solid (42 mg, 71 µmol, 71% yield).

Compound **(*S,R*)-10a** was prepared via **GP4** using **8a** (53 mg, 1.0 equiv, 0.10 mmol), Rh_2(*S-tetra-p*-BrPhPTTL)$_4$ (3.7 mg, 1 mol%, 1.0 µmol), 1,1,1,3,3,3-hexafluoropropan-2-ol (0.10 mL, 10.0 equiv, 1.0 mmol), and 1.0 mL CH_2Cl_2 at room temperature with a three hour slow addition of diazo and 0.5 h additional stirring.. Purification via flash column chromatography (SiO_2, gradient of 40% to 100% EtOAc in hexanes) afforded *tert*-butyl (*S*)-5-amino-4-(5-((*R*)-1-cyclohexyl-2-oxo-2-(2,2,2-trichloroethoxy)ethyl)-1-oxoisoindolin-2-yl)-5-oxopentanoate (**(*S,R*)-10a**) as an amorphous white solid (36 mg, 60 µmol, 60% yield).

HRMS (APCI) *m/z*: $[M+H]^+$ calcd for $C_{27}H_{36}O_6N_2{}^{35}Cl_3$ 589.1634; Found 589.1638
^{1}H NMR (600 MHz, $CDCl_3$) δ 7.78 (d, *J* = 7.9 Hz, 1H), 7.50 (s, 1H), 7.46 (dd, *J* = 7.9, 1.5 Hz, 1H), 6.34 (s, 1H), 5.46 (s, 1H), 4.90 (dd, *J* = 8.9, 6.3 Hz, 1H), 4.79 (d, *J* = 12.0 Hz, 1H), 4.61 (d, *J* = 12.0 Hz, 1H), 4.54 (d, *J* = 17.0 Hz, 1H), 4.43 (d, *J* = 17.0 Hz, 1H), 3.49 (d, *J* = 10.6 Hz, 1H), 2.42 – 2.29 (m, 2H), 2.28 – 2.19 (m, 1H), 2.19 – 2.07 (m, 2H), 1.92 – 1.85 (m, 1H), 1.79 – 1.74 (m, 1H), 1.68 – 1.60 (m, 2H), 1.41 (s, 9H), 1.36 – 1.27 (m, 2H), 1.21 – 1.09 (m, 3H), 0.80 (qd, *J* = 11.9, 3.6 Hz, 1H).
^{13}C{^{1}H} NMR (151 MHz, $CDCl_3$) δ 171.9, 171.8, 171.7, 169.1, 142.2, 141.6, 131.3, 129.3, 124.1, 123.3, 94.8, 81.0, 74.3, 59.0, 54.1, 47.3, 41.3, 32.02, 31.99, 30.5, 28.2, 26.2, 26.0, 25.9, 24.3.
SFC analysis: Using **GP1**, **(*S,R*)-10a** (CHIRALCEL® OJ-3, 5% 1:1 MeOH:^{i}PrOH with 0.2% formic acid in CO_2, 2.5 mL/min, 210 nm) indicated 98:2 d.r. for asymmetric induction observed at the newly formed stereogenic center: t_R (major diastereomer) = 7.2 min., t_R (minor diastereomer) = 5.6 min. Using **GP4**, **(*S,R*)-10a** indicated 98:2 d.r.
Specific Rotation: Specific Rotation: $[\alpha]_D^{22}$ -49.5 (*c* 1, $CHCl_3$)

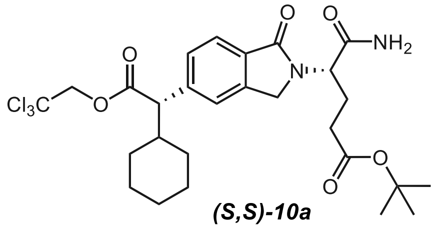

(S,S)-10a

tert-butyl (S)-5-amino-4-(5-((S)-1-cyclohexyl-2-oxo-2-(2,2,2-trichloroethoxy)ethyl)-1-oxoisoindolin-2-yl)-5-oxopentanoate ((S,S)-10a)

Compound **(*S,S*)-10a** was prepared via **GP4** using **8a** (53 mg, 1.0 equiv, 0.10 mmol), Rh_2(*R-tetra-p*-BrPhPTTL)$_4$ (3.7 mg, 1 mol%, 1.0 µmol), 1,1,1,3,3,3-hexafluoropropan-2-ol (0.10 mL, 10.0 equiv, 1.0

mmol), and 1.0 mL CH_2Cl_2 at room temperature with a three hour slow addition of diazo and 0.5 h additional stirring.. Purification via flash column chromatography (SiO_2, gradient of 40% to 100% EtOAc in hexanes) afforded *tert*-butyl (*S*)-5-amino-4-(5-((*S*)-1-cyclohexyl-2-oxo-2-(2,2,2-trichloroethoxy)ethyl)-1-oxoisoindolin-2-yl)-5-oxopentanoate ***(S,S)*-10a** as an amorphous white solid (9.3 mg, 16 µmol, 16% yield).

HRMS (APCI) *m/z*: $[M+H]^+$ calcd for $C_{27}H_{36}O_6N_2{}^{35}Cl_3$ 589.1634; Found 589.1633
^{1}H NMR (600 MHz, $CDCl_3$) δ 7.79 (d, *J* = 7.9 Hz, 1H), 7.51 (d, *J* = 1.4 Hz, 1H), 7.47 (dd, *J* = 7.9, 1.5 Hz, 1H), 6.25 (s, 1H), 5.35 (s, 1H), 4.89 (dd, *J* = 9.0, 6.3 Hz, 1H), 4.79 (d, *J* = 12.0 Hz, 1H), 4.62 (d, *J* = 12.0 Hz, 1H), 4.50 (d, *J* = 17.0 Hz, 1H), 4.45 (d, *J* = 17.0 Hz, 1H), 3.50 (d, *J* = 10.7 Hz, 1H), 2.42 – 2.30 (m, 2H), 2.29 – 2.21 (m, 1H), 2.20 – 2.10 (m, 2H), 1.93 – 1.86 (m, 1H), 1.80 – 1.72 (m, 1H), 1.67 – 1.63 (m, 2H), 1.41 (s, 9H), 1.35 – 1.27 (m, 2H), 1.22 – 1.04 (m, 3H), 0.84 – 0.74 (m, 1H).
^{13}C{^{1}H} NMR (151 MHz, $CDCl_3$) δ 171.9, 171.9, 171.8, 171.7, 169.1, 142.2, 141.5, 131.3, 129.2, 124.0, 123.4, 94.8, 81.0, 74.3, 59.0, 54.1, 47.3, 41.3, 32.0, 32.0, 30.5, 28.2, 26.2, 26.0, 25.9, 24.3.
SFC analysis: Using **GP4**, ***(S,S)*-10a** (CHIRALCEL® OJ-3, 5% 1:1 MeOH:^{i}PrOH with 0.2% formic acid in CO_2, 2.5 mL/min, 210 nm) indicated 93:7 d.r. for asymmetric induction observed at the newly formed stereogenic center: t_R (major diastereomer) = 5.6 min., t_R (minor diastereomer) = 7.2 min.
Specific Rotation: $[\alpha]_D^{22}$ -52.7 (*c* 0.5, $CHCl_3$)

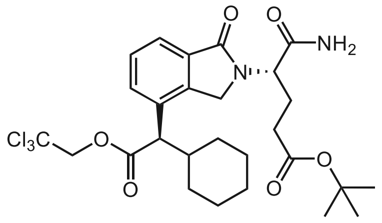

10b

***tert*-butyl (*S*)-5-amino-4-(4-((*R*)-1-cyclohexyl-2-oxo-2-(2,2,2-trichloroethoxy)ethyl)-1-oxoisoindolin-2-yl)-5-oxopentanoate (10b).**

Compound **10b** was produced via **GP1** using **8b** (53 mg, 1.0 equiv, 0.10 mmol), 1,1,1,3,3,3-hexafluoroisopropanol (0.11 mL, 10.0 equiv, 1.00 mmol), Rh_2(*S-tetra-p*-BrPhPTTL)$_4$ (20 mM in CH_2Cl_2, 50 µL, 1 mol%, 1.0 µmol), and cyclohexane (1.0 mL). Purification via flash column chromatography (SiO_2, gradient of 20% to 100% EtOAc in hexanes) afforded *tert*-butyl (*S*)-5-amino-4-(4-((*R*)-1-cyclohexyl-2-oxo-2-(2,2,2-trichloroethoxy)ethyl)-1-oxoisoindolin-2-yl)-5-oxopentanoate **(10b)** as an amorphous white solid (47 mg, 79 µmol, 79% yield).

Compound **10b** was prepared via **GP4** using cyclohexane (0.10 mL, 10.0 equiv, 0.94 mmol), Rh_2(*S-tetra-p*-BrPhPTTL)$_4$ (3.5 mg, 1 mol%, 0.94 µmol), **8b** (50 mg, 1.0 equiv, 94 mmol), 1,1,1,3,3,3-hexafluoropropan-2-ol (99 µL, 10.0 equiv, 0.94 mmol), and 0.94 mL CH_2Cl_2 at room temperature with a three hour slow addition of diazo and 0.5 h additional stirring. Purification via flash column chromatography (SiO_2, gradient of 20% to 100% EtOAc in hexanes) afforded *tert*-butyl (*S*)-5-amino-4-(4-((*R*)-1-cyclohexyl-2-oxo-2-(2,2,2-trichloroethoxy)ethyl)-1-oxoisoindolin-2-yl)-5-oxopentanoate **(10b)** as an amorphous white solid (21 mg, 35 µmol, 37% yield).

HRMS (ESI) *m/z*: $[M+H]^+$ calcd for $C_{27}H_{36}O_6N_2{}^{35}Cl_3$ 589.1634, Found 589.1632
^{1}H NMR (400 MHz, $CDCl_3$) δ 7.76 (dd, *J* = 7.5, 1.0 Hz, 1H), 7.67 (dd, *J* = 7.8, 1.0 Hz, 1H), 7.48 (t, *J* = 7.6 Hz, 1H), 6.25 (s, 1H), 5.30 (s, 1H), 4.90 (dd, *J* = 8.2, 6.7 Hz, 1H), 4.70 (d, *J* = 11.9 Hz, 1H), 4.64 (d, *J* = 12.0 Hz, 1H), 4.50 (d, *J* = 16.9 Hz, 1H), 3.46 (d, *J* = 11.0 Hz, 1H), 2.48 – 2.33 (m, 2H), 2.32 – 2.10 (m,

3H), 1.99 – 1.88 (m, 1H), 1.85 – 1.75 (m, 1H), 1.72 – 1.52 (m, 3H), 1.41 (s, 9H), 1.23 – 1.12 (m, 4H), 0.92 – 0.62 (m, 1H).

^{13}C{^{1}H} NMR (151 MHz, $CDCl_3$) δ 171.9, 171.5, 171.1, 169.3, 141.4, 132.4, 132.2, 131.2, 129.1, 123.2, 94.7, 81.0, 74.2, 54.6, 54.2, 46.8, 39.9, 32.0, 30.3, 28.2, 26.2, 25.95, 25.92, 24.2.

SFC analysis: Using **GP1**, **10b** (Trefoil® AMY1, 20% 1:1 MeOH:^{i}PrOH with 0.2% formic acid in CO_2, 1.5 mL/min, 210 nm) indicated 99:1 d.r. for asymmetric induction observed at the newly formed stereogenic center: t_R (major diastereomer) = 2.7 min., t_R (minor diastereomer) = 1.2 min. Using **GP4**, **10b** indicated 98:2 d.r.

Specific Rotation: $[\alpha]_D^{24}$ -106.2 (c 0.25, $CHCl_3$)

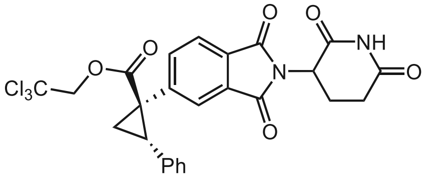

11a

2,2,2-trichloroethyl (1*R*,2*S*)-1-(2-(2,6-dioxopiperidin-3-yl)-1,3-dioxoisoindolin-5-yl)-2-phenylcyclopropane-1-carboxylate (11a).

Compound **11a** was prepared via **GP5** using styrene (57 µL, 5.0 equiv, 0.50 mmol), Rh_2(*S-tetra-p-*BrPhPTTL)$_4$ (3.7 mg, 1 mol%, 1.0 µmol), **6a** (47 mg, 1.0 equiv, 0.10 mmol), 1,1,1,3,3,3-hexafluoropropan-2-ol (0.10 mL, 10.0 equiv, 1.0 mmol), and 1.0 mL CH_2Cl_2 at room temperature with a 0.25 hour slow addition of diazo and 0.5 h additional stirring. Purification via flash column chromatography (SiO_2, gradient of 30% to 50% EtOAc in hexanes) afforded 2,2,2-trichloroethyl (1*R*,2*S*)-1-(2-(2,6-dioxopiperidin-3-yl)-1,3-dioxoisoindolin-5-yl)-2-phenylcyclopropane-1-carboxylate **(11a)** as an amorphous white solid (50 mg, 90 µmol, 90% yield).

HRMS (APCI) *m/z*: $[M+H]^+$ calcd for $C_{25}H_{20}O_6N_2{}^{35}Cl_3$ 549.0382; Found 549.0381

^{1}H NMR (400 MHz, $CDCl_3$, reported as a mixture of diastereomers) δ 8.15 (s, 1H), 7.74 – 7.67 (m, 1H), 7.59 (dd, *J* = 7.8, 2.4 Hz, 1H), 7.38 (dt, *J* = 7.8, 1.7 Hz, 1H), 7.17 – 7.07 (m, 3H), 6.92 – 6.80 (m, 2H), 4.94 and 4.93 (dd, *J* = 12.3, 5.4 Hz, 1H), 4.87 and 4.86 (d, *J* = 12.0 Hz, 1H), 4.64 (d, *J* = 11.9 Hz, 1H), 3.36 (dd, *J* = 9.4, 7.6 Hz, 1H), 2.94 – 2.85 (m, 1H), 2.84 – 2.64 (m, 2H), 2.39 (dd, *J* = 9.4, 5.5 Hz, 1H), 2.15-2.09 (m, 1H, obfuscated by dd at 2.13), 2.13 (dd, *J* = 7.8, 5.6 Hz, 1H, obfuscated by m from 2.15-2.09).

^{13}C{^{1}H} NMR (151 MHz, $CDCl_3$, reported as a mixture of diastereomers) δ 171.0, 170.8, 168.1, 167.09, 167.06, 167.02, 167.00, 141.9, 138.82, 138.76, 134.4, 131.6, 131.5, 130.7, 128.6, 128.2, 127.5, 127.1, 127.0, 123.2, 94.9, 74.6, 49.41, 49.39, 37.1, 34.7, 31.47, 31.45, 22.8, 22.7, 20.04, 20.03.

SFC analysis: Using **GP5**, **11a** (CHIRALCEL® OZ-3, 20% 1:1 MeOH:^{i}PrOH with 0.2% formic acid in CO_2, 2.5 mL/min, 210 nm) indicated 86:14 d.r. for asymmetric induction observed from formation of the major relative diasteromer: t_R (major diastereomers) = 7.3 and 8.8 min., t_R (minor diastereomers) = 6.1 and 6.8 min.

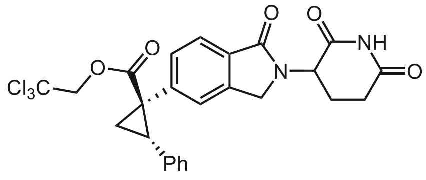

11b

2,2,2-trichloroethyl (1*R*,2*S*)-1-(2-(2,6-dioxopiperidin-3-yl)-1-oxoisoindolin-5-yl)-2-phenylcyclopropane-1-carboxylate (11b).

Compound **11b** was prepared via **GP5** using styrene (57 µL, 5.0 equiv, 0.50 mmol), Rh_2(*S*-*tetra*-*p*-BrPhPTTL)$_4$ (3.7 mg, 1 mol%, 1.0 µmol), **6b** (47 mg, 1.0 equiv, 0.10 mmol), 1,1,1,3,3,3-hexafluoropropan-2-ol (0.10 mL, 10.0 equiv, 1.0 mmol), and 1.0 mL CH_2Cl_2 at room temperature with a 0.25 hour slow addition of diazo and 0.5 h additional stirring. Purification via flash column chromatography (SiO_2, gradient of 40% to 100% EtOAc in hexanes) afforded 2,2,2-trichloroethyl (1*R*,2*S*)-1-(2-(2,6-dioxopiperidin-3-yl)-1-oxoisoindolin-5-yl)-2-phenylcyclopropane-1-carboxylate **(11b)** as an amorphous white solid (45 mg, 83 µmol, 83% yield).

HRMS (APCI) *m/z*: $[M+H]^+$ calcd for $C_{25}H_{22}O_5N_2{}^{35}Cl_3$ 535.0589; Found 535.0591
^{1}H NMR (600 MHz, $CDCl_3$) δ 8.01 and 8.00, (s, 1H), 7.66 and 7.63 (d, *J* = 7.9 Hz, 1H), 7.24 and 7.17 (d, *J* = 8.0 Hz, 1H), 7.21 and 7.15 (s, 1H), 7.12 – 7.05 (m, 3H), 6.89 – 6.76 (m, 2H), 5.17 and 5.16 (dd, *J* = 13.6, 5.0 Hz, 1H), 4.855 and 4.849 (d, *J* = 11.9 Hz, 1H), 4.64 (d, *J* = 11.9 Hz, 1H), 4.39 and 4.29 (d, *J* = 15.8 Hz, 1H), 4.21 and 4.14 (d, *J* = 15.9 Hz, 1H), 3.29 (t, *J* = 8.4 Hz, 1H), 2.95 – 2.86 (m, 1H), 2.85 – 2.74 (m, 1H), 2.41 – 2.33 (m, 1H), 2.33 – 2.25 (m, 1H), 2.21 – 2.12 (m, 1H), 2.10 – 2.03 (m, 1H).
^{13}C{^{1}H} NMR (151 MHz, $CDCl_3$, reported as a mixture of diastereomers) δ 171.5, 171.0, 169.55, 169.51, 169.1, 141.1, 141.0, 138.71, 138.69, 135.14, 135.09, 132.6, 132.5, 130.7, 130.6, 128.3, 128.19, 128.17, 127.2, 127.1, 126.64, 126.67, 123.6, 123.6, 95.1, 74.5, 52.0, 51.9, 46.94, 46.89, 37.4, 37.3, 34.4, 34.3, 31.6, 23.54, 23.51, 20.5, 20.4.
SFC analysis: **11b** (CHIRALPAK® AS-3, 20% 1:1 MeOH:^{i}PrOH with 0.2% formic acid in CO_2, 2.5 mL/min, 210 nm) indicated 86:16 d.r. for asymmetric induction arising from formation of the major relative diastereomer using **GP5**: t_R (major diastereomer) = 2.50 min., t_R (minor diastereomer) = 2.84 min, t_R (inseparable major + minor) = 1.92 min.
Note: The other two diastereomers represented by the peak at 1.92 min were inseparable, and the d.r. was acquired from the diastereomers at 2.50 and 2.84 min. only. Suppression of the peak at 1.92 min. in the chromatogram for **SI4** indicates that these diastereomers correspond to both diastereomers of the cyclopropane with *R* glutarimide stereogenic centers.

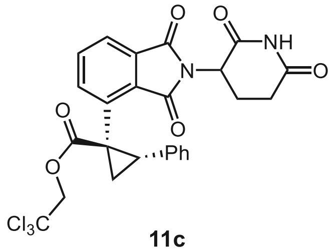

11c

2,2,2-trichloroethyl (1*R*,2*S*)-1-(2-(2,6-dioxopiperidin-3-yl)-1,3-dioxoisoindolin-4-yl)-2-phenylcyclopropane-1-carboxylate (11c).

Compound **11c** was prepared via **GP5** using styrene (57 µL, 5.0 equiv, 0.50 mmol), Rh_2(*S*-*tetra*-*p*-BrPhPTTL)$_4$ (3.7 mg, 1 mol%, 1.0 µmol), **6c** (47 mg, 1.0 equiv, 0.10 mmol), 1,1,1,3,3,3-hexafluoropropan-2-ol (0.10 mL, 10.0 equiv, 1.0 mmol), and 1.0 mL CH_2Cl_2 at room temperature with a

0.25 hour slow addition of diazo and 0.5 h additional stirring. Purification via flash column chromatography (SiO_2, gradient of 20% to 40% EtOAc in hexanes) afforded 2,2,2-trichloroethyl (1*R*,2*S*)-1-(2-(2,6-dioxopiperidin-3-yl)-1,3-dioxoisoindolin-4-yl)-2-phenylcyclopropane-1-carboxylate **(11c)** as an amorphous white solid (40 mg, 73 μmol, 73% yield).

HRMS (APCI) *m/z*: $[M+H]^+$ calcd for $C_{25}H_{20}O_6N_2{}^{35}Cl_3$ 549.0382; Found 549.0385
^{1}H NMR (400 MHz, $CDCl_3$, reported as a mixture of diastereomers) δ 8.00 (s, 1H), 7.73 – 7.61 (m, 1H), 7.61 – 7.37 (m, 2H), 7.08 – 6.90 (m, 3H), 6.88 – 6.71 (m, 2H), 4.82 (dd, *J* = 12.0, 5.7 Hz, 2H), 4.75 (d, *J* = 11.9 Hz, 1H), 4.64 and 4.62 (d, *J* = 11.9 Hz, 1H), 3.59 – 3.43 (m, 1H), 2.95 – 2.80 (m, 1H), 2.77 – 2.58 (m, 2H), 2.38 – 2.26 (m, 1H), 2.22 and 2.17 (t, *J* = 6.6 Hz, 1H), 2.03 – 1.86 (m, 1H).
^{13}C{^{1}H} NMR (151 MHz, $CDCl_3$, reported as a mixture of diastereomers) δ 170.99, 170.97, 170.6, 170.5, 167.63, 167.57, 166.9, 166.8, 166.7, 133.93, 133.90, 132.02, 131.99, 131.7, 128.4, 128.2, 127.94, 127.86, 127.12, 127.09, 122.94, 122.88, 94.6, 94.5, 75.3, 75.1, 49.1, 49.0, 34.3, 34.2, 34.1, 31.5, 31.4, 22.8, 22.5.
SFC analysis: Using **GP5**, **11c** (CHIRALPAK® AD-3, 20% 1:1 MeOH:^{i}PrOH with 0.2% formic acid in CO_2, 2.5 mL/min, 210 nm) indicated 60:40 d.r. for asymmetric induction observed from formation of the major relative diasteromer: t_R (major diastereomers) = 2.0 and 2.5 min., t_R (minor diastereomers) = 2.2 and 3.5 min.

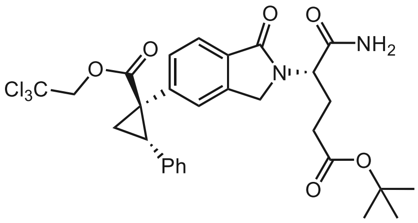

12a

2,2,2-trichloroethyl (1*R*,2*S*)-1-(2-((*S*)-1-amino-5-(*tert*-butoxy)-1,5-dioxopentan-2-yl)-1-oxoisoindolin-5-yl)-2-phenylcyclopropane-1-carboxylate (12a).

Compound **12a** was prepared via **GP6** using styrene (57 μL, 5.0 equiv, 0.50 mmol), Rh_2(*S*-*tetra*-*p*-BrPhPTTL)$_4$ (3.7 mg, 1 mol%, 1.0 μmol), **8a** (53 mg, 1.0 equiv, 0.10 mmol), 1,1,1,3,3,3-hexafluoropropan-2-ol (0.10 mL, 10.0 equiv, 1.0 mmol), and 1.0 mL CH_2Cl_2 at room temperature with a 0.25 hour slow addition of diazo and 0.5 h additional stirring. Purification via flash column chromatography (SiO_2, gradient of 40% to 100% EtOAc in hexanes) afforded 2,2,2-trichloroethyl (1*R*,2*S*)-1-(2-((*S*)-1-amino-5-(*tert*-butoxy)-1,5-dioxopentan-2-yl)-1-oxoisoindolin-5-yl)-2-phenylcyclopropane-1-carboxylate **(12a)** as an amorphous white solid (53 mg, 88 μmol, 88% yield).

HRMS (APCI): *m/z* $[M+H]^+$ calcd for $C_{29}H_{32}O_6N_2{}^{35}Cl_3$, 609.1320; Found 609.1320.

^{1}H NMR (400 MHz, $CDCl_3$) δ 7.58 (d, *J* = 8.2 Hz, 1H), 7.20 – 7.14 (m, 2H), 7.11 – 7.03 (m, 3H), 6.81 (dd, *J* = 6.7, 2.9 Hz, 2H), 6.40 (s, 1H), 5.49 (s, 1H), 4.84 (d, *J* = 12.0 Hz) 4.83 (dd, *J* = 3.3, 2.8 Hz), 4.63 (d, *J* = 11.9 Hz, 1H), 4.32 (s, 2H), 3.29 (dd, *J* = 9.4, 7.5 Hz, 1H), 2.38 – 2.09 (m, 5H), 2.06 (dd, *J* = 7.4, 5.4 Hz, 1H), 1.40 (s, 9H).

^{13}C NMR (101 MHz, $CDCl_3$) δ 171.9, 171.7, 171.5, 169.1, 141.2, 138.5, 135.0, 132.4, 130.9, 128.3, 128.2, 127.2, 126.7, 123.2, 95.0, 81.0, 74.5, 54.0, 47.1, 37.3, 34.3, 32.0, 28.2, 24.2, 20.4.

SFC analysis: Using **GP6**, **12a** (Trefoil® CEL1, 10% 1:1 MeOH:^{i}PrOH with 0.2% formic acid in CO_2, 2.5 mL/min, 210 nm) indicated 91:9 d.r. for asymmetric induction observed from formation of the major relative diasteromer: t_R (major diastereomer) = 5.1 min., t_R (minor diastereomer) = 5.6 min.
Specific Rotation: $[\alpha]_D^{22}$ -40.5 (*c* 1.0, $CHCl_3$)

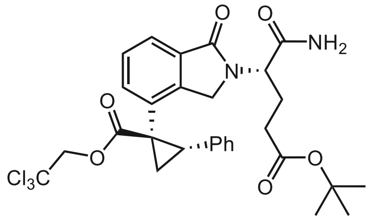

12b

2,2,2-trichloroethyl (1*R*,2*S*)-1-(2-((S)-1-amino-5-(*tert*-butoxy)-1,5-dioxopentan-2-yl)-1-oxoisoindolin-4-yl)-2-phenylcyclopropane-1-carboxylate (12b).

Compound **12b** was prepared via **GP6** using styrene (57 µL, 5.0 equiv, 0.50 mmol), Rh_2(*S*-*tetra*-*p*-BrPhPTTL)$_4$ (3.7 mg, 1 mol%, 1.0 µmol), **8b** (53 mg, 1.0 equiv, 0.10 mmol), 1,1,1,3,3,3-hexafluoropropan-2-ol (0.10 mL, 10.0 equiv, 1.0 mmol), and 1.0 mL CH_2Cl_2 at room temperature with a 0.25 hour slow addition of diazo and 0.5 h additional stirring. Purification via flash column chromatography (SiO_2, gradient of 40% to 100% EtOAc in hexanes) afforded 2,2,2-trichloroethyl (1*R*,2*S*)-1-(2-((*S*)-1-amino-5-(*tert*-butoxy)-1,5-dioxopentan-2-yl)-1-oxoisoindolin-4-yl)-2-phenylcyclopropane-1-carboxylate **(12b)** as an amorphous white solid (51 mg, 84 µmol, 84% yield).

HRMS (ESI): *m/z* $[M+H]^+$ calcd for $C_{29}H_{32}O_6N_2{}^{35}Cl_3$, 609.1321; Found 609.1316.

^{1}H NMR (400 MHz, $CDCl_3$) δ 7.75 – 7.68 (m, 1H), 7.48 – 7.35 (m, 2H), 7.11 – 6.97 (m, 3H), 6.84 – 6.66 (m, 2H), 5.72 (s, 1H), 5.11 (s, 1H), 4.79 (d, *J* = 11.9 Hz, 1H), 4.74 (dd, *J* = 9.0, 6.1 Hz, 1H), 4.68 (d, *J* = 11.9 Hz, 1H), 4.37 (d, *J* = 17.1 Hz, 1H), 3.65 (d, *J* = 17.1 Hz, 1H), 3.27 (dd, *J* = 9.6, 7.4 Hz, 1H), 2.39 (dd, *J* = 9.5, 5.1 Hz, 1H), 2.35 – 2.21 (m, 2H), 2.19 – 2.08 (m, 1H), 2.01 – 1.91 (m, 1H, partially obscured by signal at 2.00), 2.00 (dd, *J* = 7.4, 5.3 Hz, 1H), 1.41 (s, 8H).

^{13}C{^{1}H} NMR (151 MHz, $CDCl_3$) δ 171.8, 171.2, 170.7, 169.0, 143.8, 135.23, 135.16, 131.9, 129.7, 128.5, 128.2, 127.6, 127.1, 123.7, 94.8, 80.9, 74.7, 54.1, 46.8, 35.1, 33.8, 32.1, 28.2, 24.0, 21.5.

SFC analysis: Using **GP6**, **12b** (Trefoil® CEL1, 10% 1:1 MeOH:^{i}PrOH with 0.2% formic acid in CO_2, 2.5 mL/min, 210 nm) indicated 93:7 d.r. for asymmetric induction observed from formation of the major relative diasteromer: t_R (major diastereomer) = 2.4 min., t_R (minor diastereomer) = 2.8 min.
Specific Rotation: $[\alpha]_D^{22}$ -76.5 (*c* 1, $CHCl_3$)

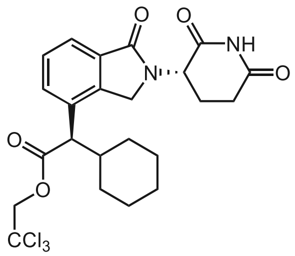

13

2,2,2-trichloroethyl (*R*)-2-cyclohexyl-2-(2-((*S*)-2,6-dioxopiperidin-3-yl)-1-oxoisoindolin-4-yl)acetate (13).

Compound **13** was produced following **GP 7**, using **10b** (15 mg, 1.0 equiv, 25 µmol) and benzenesulfonic acid (7.8 mg, 2.0 equiv, 49 µmol) in acetonitrile (0.12 mL) at 70 °C for 3.5 h. Purification via flash column chromatography (SiO_2, 65% ethyl acetate in hexanes) gave 2,2,2-trichloroethyl (*R*)-2-cyclohexyl-2-(2-((*S*)-2,6-dioxopiperidin-3-yl)-1-oxoisoindolin-4-yl)acetate **(13)** as an amorphous white solid (7.9 mg, 62% yield, 15 µmol).

HRMS (APCI) *m/z*: $[M+H]^+$ calcd for $C_{23}H_{26}O_5N_2{}^{35}Cl_3$ 515.0902; Found 515.0902

^{1}H NMR (400 MHz, $CDCl_3$) δ 8.05 (s, 1H), 7.82 (dd, *J* = 7.5, 1.0 Hz, 1H), 7.68 (dd, *J* = 7.7, 1.1 Hz, 1H), 7.51 (t, *J* = 7.6 Hz, 1H), 5.22 (dd, *J* = 13.3, 5.1 Hz, 1H), 4.72 (d, *J* = 11.9 Hz, 1H), 4.64 (d, *J* = 12.0 Hz, 1H), 4.59 (d, *J* = 16.0 Hz, 1H), 4.40 (d, *J* = 16.0 Hz, 1H), 3.44 (d, *J* = 11.1 Hz, 1H), 3.11 – 2.64 (m, 2H), 2.43 (qd, *J* = 13.1, 5.0 Hz, 1H), 2.30 – 2.18 (m, 2H), 2.00 – 1.90 (m, 1H), 1.85 – 1.74 (m, 1H), 1.71 – 1.59 (m, 2H), 1.47 – 1.32 (m, 2H), 1.22 – 1.05 (m, 3H), 0.82 – 0.66 (m, 1H).
^{13}C{^{1}H} NMR (101 MHz, $CDCl_3$) δ 171.1, 169.33, 169.27, 141.0, 132.1, 131.9, 131.6, 129.3, 123.7, 94.7, 74.3, 55.1, 52.0, 46.7, 39.7, 32.1, 31.7, 30.4, 26.2, 25.9, 23.6.
SFC analysis: **13** (CHIRALPAK® AD-3, 20% 1:1 MeOH:^{i}PrOH with 0.2% formic acid in CO_2, 2.5 mL/min, 210 nm) indicated 99:1 d.r. for retained asymmetric induction arising from formation of the major relative diastereomer and 99:1 d.r. for major relative configuration of the glutarimide stereogenic center: t_R (major diastereomer) = 2.9 min., t_R (minor diastereomers) = 2.1, 2.3, and 3.3 min.
Specific Rotation: $[\alpha]_D^{24}$ -37.9 (c 0.5, $CHCl_3$)

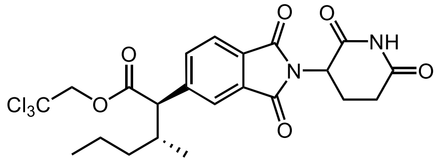

(2*R*,3*S*)-14

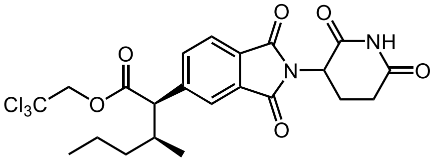

(2*R*,3*R*)-14

2,2,2-trichloroethyl (2*R*,3*S*)-2-(2-(2,6-dioxopiperidin-3-yl)-1,3-dioxoisoindolin-5-yl)-3-methylhexanoate ((2*R*,3*S*)-14).

2,2,2-trichloroethyl (2*R*,3*S*)-2-(2-(2,6-dioxopiperidin-3-yl)-1,3-dioxoisoindolin-5-yl)-3-methylhexanoate ((2*R*,3*R*)-14).

Compound **14** was produced via **GP1** using **6a** (47 mg, 1.0 equiv, 0.10 mmol), 1,1,1,3,3,3-hexafluoroisopropanol (0.11 mL, 10.0 equiv, 1.00 mmol), Rh_2(*S*-*tetra*-*p*-BrPhPTTL)$_4$ (20 mM in CH_2Cl_2, 50 µL, 1 mol%, 1.0 µmol), and pentane (1.0 mL). Purification via flash column chromatography (SiO_2, 20% to 50% ethyl acetate in hexanes) produced a mixture of 2,2,2-trichloroethyl (2*R*,3*S*)-2-(2-(2,6-dioxopiperidin-3-yl)-1,3-dioxoisoindolin-5-yl)-3-methylhexanoate **((2*R*,3*S*)-14)** and 2,2,2-trichloroethyl (2*R*,3*S*)-2-(2-(2,6-dioxopiperidin-3-yl)-1,3-dioxoisoindolin-5-yl)-3-methylhexanoate **((2*R*,3*R*)-14)** as an amorphous white solid (42 mg, 82 µmol, 82% yield). **((2*R*,3*S*)-14)** was generated as the major diastereomer in 3.7:1 d.r.

HRMS (APCI) *m/z*: $[M+H]^+$ calcd for $C_{22}H_{24}O_6N_2{}^{35}Cl_3$ 517.0695; Found 517.0696
^{1}H NMR (600 MHz, $CDCl_3$, reported as a mixture of diastereomers) δ 8.07 (s, 1H), 7.94 (s, 1H), 7.852 and 7.848 (d, *J* = 7.8 Hz, 1H), 7.775, 7.772, and 7.770 (d, *J* = 7.8 Hz, 1H), 4.991 and 4.987 (dd, *J* = 12.7, 5.4 Hz, 1H), 4.80 and 4.79 (d, *J* = 11.9 Hz, 1H), 4.65 (d, *J* = 12.0 Hz, 1H), 3.61 and 3.60 (d, *J* = 10.5 Hz, 1H), 2.96 – 2.89 (m, 1H), 2.88 – 2.70 (m, 2H), 2.41 – 2.30 (m, 1H), 2.21 – 2.11 (m, 1H), 1.54-1.44 and 1.30 – 1.23 (m, 1H), 1.41 – 1.31 (m, 1H), 1.22 – 1.15 (m, 1H), 1.14 – 1.06 and 0.99 – 0.95 (m, 1H, partially occluded by signal at 1.09), 1.09 and 0.74 (d, *J* = 6.6 Hz, 3H), 0.92 and 0.74 (t, *J* = 6.8 Hz, 3H)
^{13}C{^{1}H} NMR (151 MHz, $CDCl_3$, reported as a mixture of diastereomers) δ 171.13, 171.06, 170.8, 167.99, 167.97, 167.1, 167.0, 145.04, 145.01, 135.3, 135.21, 135.24, 132.34, 132.32, 131.07, 131.05, 124.4, 124.3, 124.2, 124.1, 94.7, 74.4, 59.0, 49.5, 37.4, 36.7, 36.6, 35.8, 31.5, 22.8, 19.9, 19.6, 17.8, 16.8, 14.3, 14.2.
SFC analysis: Using **GP1**, **(2*R*,3*S*)-14 and (2*R*,3*R*)-14** (CHIRALCEL® OX-3, 15% 1:1 MeOH:^{i}PrOH with 0.2% formic acid in CO_2, 2.0 mL/min, 210 nm) indicated 96:4 d.r. for the asymmetric induction arising from formation of the major relative diastereomer: t_R (major diastereomers) = 9.3 and 11.4 min., t_R (minor diastereomers) = 14.2 and 16.6 min., and 95:5 d.r. for the asymmetric induction arising from formation of the minor relative diastereomer: t_R (major diastereomers) = 10.5 and 12.3 min., t_R (minor diastereomers) = 13.3 and 15.2 min.

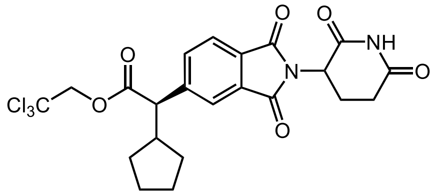

15

2,2,2-trichloroethyl (2*R*)-2-cyclopentyl-2-(2-(2,6-dioxopiperidin-3-yl)-1,3-dioxoisoindolin-5-yl)acetate (15).

Compound **15** was produced via **GP1** using **6a** (47 mg, 1.0 equiv, 0.10 mmol), 1,1,1,3,3,3-hexafluoroisopropanol (0.11 mL, 10.0 equiv, 1.00 mmol), Rh_2(*S*-*tetra*-*p*-BrPhPTTL)$_4$ (20 mM in CH_2Cl_2, 50 µL, 1 mol%, 1.0 µmol), and cyclopentane (1.0 mL). Purification via flash column chromatography (SiO_2, 20% to 50% ethyl acetate in hexanes) produced 2,2,2-trichloroethyl (2*R*)-2-cyclopentyl-2-(2-(2,6-dioxopiperidin-3-yl)-1,3-dioxoisoindolin-5-yl)acetate **(15)** as an amorphous white solid (50 mg, 50 µmol, 96% yield).

HRMS (APCI): *m/z* $[M+H]^+$ calcd for $C_{22}H_{22}O_6N_2{}^{35}Cl_3$ 515.0538, Found 515.0546

^{1}H NMR (400 MHz, $CDCl_3$, reported as a mixture of diastereomers) δ 8.05 (s, 1H), 7.96 – 7.94 (m, 1H), 7.85 (dd, *J* = 7.7, 0.7 Hz, 1H), 7.78 (dd, *J* = 7.8, 1.5 Hz, 1H), 4.99 (dd, *J* = 12.5, 5.3 Hz, 1H), 4.79 (d, *J* = 12.0 Hz, 1H), 4.66 (d, *J* = 12.0 Hz, 1H), 3.61 (d, *J* = 11.1 Hz, 1H), 3.03 – 2.72 (m, 3H), 2.72 – 2.59 (m, 1H), 2.23 – 2.08 (m, 1H), 2.06 – 1.95 (m, 1H), 1.76 – 1.58 (m, 3H), 1.56 – 1.42 (m, 2H), 1.41-1.31 (m, 1H) 1.08 – 0.93 (m, 1H).

^{13}C{^{1}H} NMR (151 MHz, $CDCl_3$, reported as a mixture of diastereomers) δ 171.04, 171.02, 168.1, 167.1, 167.0, 145.7, 134.8, 132.4, 131.0, 124.2, 124.0, 94.7, 74.4, 57.9, 49.5, 43.6, 43.6, 31.6, 31.5, 30.9, 25.2, 25.0, 22.7.

SFC analysis: Using **GP1**, **15** (CHIRALCEL® OX-3, 30% 1:1 MeOH:^{i}PrOH with 0.2% formic acid in CO_2, 2.5 mL/min, 230 nm) indicated >99:1 d.r. for the asymmetric induction observed at the newly formed stereogenic center: t_R (major diastereomers) = 2.6 and 3.0 min., t_R (minor diastereomers) = 3.3 and 3.6 min.

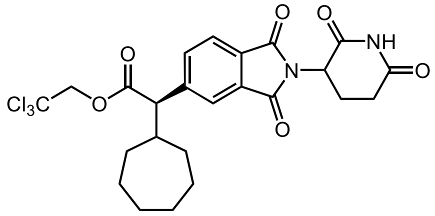

16

2,2,2-trichloroethyl (2*R*)-2-cycloheptyl-2-(2-(2,6-dioxopiperidin-3-yl)-1,3-dioxoisoindolin-5-yl)acetate (16).

Compound **16** was produced via **GP1** using **6a** (47 mg, 1.0 equiv, 0.10 mmol), 1,1,1,3,3,3-hexafluoroisopropanol (0.11 mL, 10.0 equiv, 1.00 mmol), Rh_2(*S*-*tetra*-*p*-BrPhPTTL)$_4$ (20 mM in CH_2Cl_2, 50 µL, 1 mol%, 1.0 µmol), and cycloheptane (1.0 mL). Purification via flash column chromatography (SiO_2, 20% to 50% ethyl acetate in hexanes) produced 2,2,2-trichloroethyl (2*R*)-2-cycloheptyl-2-(2-(2,6-dioxopiperidin-3-yl)-1,3-dioxoisoindolin-5-yl)acetate **(16)** as an amorphous white solid (52 mg, 96 µmol, 96% yield).

HRMS (APCI): *m/z* $[M+H]^+$ calcd for $C_{24}H_{26}O_6N_2{}^{35}Cl_3$, 543.0851; Found 543.0857.

^{1}H NMR (400 MHz, $CDCl_3$, reported as a mixture of diastereomers) δ 8.07 (s, 1H), 7.94 (dd, *J* = 1.7, 0.6 Hz, 1H), 7.84 (dd, *J* = 7.7, 0.7 Hz, 1H), 7.77 (dd, *J* = 7.8, 1.5 Hz, 1H), 4.99 (dd, *J* = 12.3, 5.2 Hz, 1H), 4.77 (d, *J* = 12.0 Hz, 1H), 4.655 and 4.653 (d, *J* = 12.0 Hz, 1H), 3.65 (d, *J* = 10.8 Hz, 1H), 2.97 – 2.66 (m, 3H), 2.47 – 2.28 (m, 1H), 2.20 – 2.09 (m, 1H), 1.93 – 1.77 (m, 1H), 1.76 – 1.66 (m, 1H), 1.64 – 1.30 (m, 9H), 1.10 – 0.98 (m, 1H).

^{13}C{^{1}H} NMR (151 MHz, $CDCl_3$, reported as a mixture of diastereomers) δ 171.2, 170.8, 168.0, 167.1, 167.0, 145.3, 135.3, 132.3, 131.0, 124.4, 124.2, 94.7, 74.5, 59.1, 49.5, 42.6, 33.1, 31.6, 31.5, 28.3, 28.2, 26.31, 26.29, 26.2, 22.8.

SFC analysis: Using **GP1**, **16** (Trefoil® AMY1, 30% 1:1 MeOH:^{i}PrOH with 0.2% formic acid in CO_2, 2.5 mL/min, 230 nm) indicated 95:5 d.r. for the asymmetric induction observed at the newly formed stereogenic center: t_R (major diastereomers) = 2.9 and 7.9 min., t_R (minor diastereomers) = 2.4 and 5.3 min.

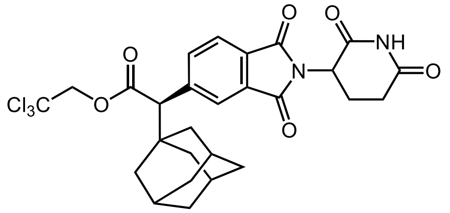

17

2,2,2-trichloroethyl (2*R*)-2-((3*R*,5*R*,7*R*)-adamantan-1-yl)-2-(2-(2,6-dioxopiperidin-3-yl)-1,3-dioxoisoindolin-5-yl)acetate (17).

Compound **17** was prepared via **GP3** using admantane (140 mg, 10.0 equiv, 1.0 mmol), Rh_2(*S-tetra-p*-BrPhPTTL)$_4$ (3.7 mg, 1 mol%, 1.0 μmol), **6a** (47 mg, 1.0 equiv, 0.10 mmol), 1,1,1,3,3,3-hexafluoropropan-2-ol (0.10 mL, 10.0 equiv, 1.0 mmol), and 1.0 mL CH_2Cl_2 at room temperature with a one hour slow addition of diazo and 0.5 h additional stirring. Excess adamantane was removed after reaction by sublimation via Kugelrohr (100 °C, <10 torr, 10 min). Purification via flash column chromatography (SiO_2, gradient of 20% to 50% EtOAc in hexanes) afforded 2,2,2-trichloroethyl (2*R*)-2-((3*R*,5*R*,7*R*)-adamantan-1-yl)-2-(2-(2,6-dioxopiperidin-3-yl)-1,3-dioxoisoindolin-5-yl)acetate **(17)** as an amorphous white solid (47 mg, 81 μmol, 81% yield).

HRMS (APCI) *m/z*: $[M+H]^+$ calcd for $C_{27}H_{28}O_6N_2{}^{35}Cl_3$ 581.1008; Found 581.1006

^{1}H NMR (600 MHz, $CDCl_3$, reported as a mixture of diastereomers) δ 8.05 – 7.93 (m, 2H), 7.84 (d, *J* = 7.7 Hz, 1H), 7.80 (dt, *J* = 7.8, 1.9 Hz, 1H), 5.00 (dd, *J* = 12.6, 5.4 Hz, 1H), 4.812 and 4.811 (d, *J* = 12.0 Hz, 1H), 4.665 and 4.663 (d, *J* = 12.0 Hz, 1H), 3.61 (s, 1H), 2.97 – 2.89 (m, 1H), 2.89 – 2.81 (m, 1H), 2.79 – 2.69 (m, 1H), 2.18 – 2.13 (m, 1H), 2.04 – 1.81 (m, 2H), 1.82 – 1.63 (m, 3H), 1.61 – 1.38 (m, 3H).

^{13}C{^{1}H} NMR (151 MHz, $CDCl_3$, reported as a mixture of diastereomers) δ 170.9, 170.0, 168.1, 167.3, 167.2, 141.9, 136.4, 131.6, 130.8, 125.6, 123.3, 94.7, 74.5, 63.2, 49.5, 40.0, 37.4, 36.6, 31.5, 28.6, 22.8.

SFC analysis: Using **GP3**, **17** (CHIRALCEL® OX-3, 30% 1:1 MeOH:^{i}PrOH with 0.2% formic acid in CO_2, 2.5 mL/min, 230 nm) indicated 98:2 d.r. for the asymmetric induction observed at the newly formed stereogenic center: t_R (major diastereomers) = 3.8 and 4.7 min., t_R (minor diastereomers) = 5.3 and 5.8 min.

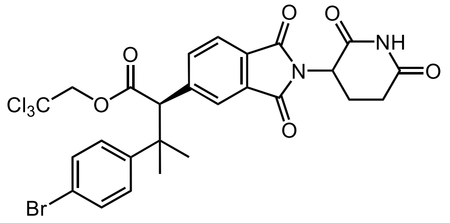

18

2,2,2-trichloroethyl (2*S*)-3-(4-bromophenyl)-2-(2-(2,6-dioxopiperidin-3-yl)-1,3-dioxoisoindolin-5-yl)-3-methylbutanoate (18).

Compound **18** was prepared via **GP3** using 1-bromo-4-isopropylbenzene (0.16 mL, 10.0 equiv, 1.0 mmol), Rh_2(*S*-*tetra*-*p*-BrPhPTTL)$_4$ (3.7 mg, 1 mol%, 1.0 µmol), **6a** (47 mg, 1.0 equiv, 0.10 mmol), 1,1,1,3,3,3-hexafluoropropan-2-ol (0.10 mL, 10.0 equiv, 1.0 mmol), and 1.0 mL CH_2Cl_2 at room temperature with a one hour slow addition of diazo and 0.5 h additional stirring. Excess 1-bromo-4-isopropylbenzene was removed from the reaction mixture via Kugelrohr distillation (100 °C, <10 torr, 10 min). Purification via flash column chromatography (SiO_2, gradient of 20% to 50% EtOAc in hexanes) followed by further purification (C18, 40-90% MeCN in H_2O, 0.1% v/v as buffer) afforded 2,2,2-trichloroethyl (2*S*)-3-(4-bromophenyl)-2-(2-(2,6-dioxopiperidin-3-yl)-1,3-dioxoisoindolin-5-yl)-3-methylbutanoate **(18)** as an amorphous white solid (23 mg, 35 µmol, 35% yield).

HRMS (APCI) *m/z*: $[M+H]^+$ calcd for $C_{26}H_{23}O_6N_2{}^{79}Br^{35}Cl_3$ 642.9800; Found 642.9805
^{1}H NMR (400 MHz, $CDCl_3$, reported as a mixture of diastereomers) δ 8.09 (s, 1H), 7.96 – 7.84 (m, 1H), 7.77 – 7.71 (m, 1H), 7.53 – 7.46 (m, 1H), 7.42 (d, *J* = 8.7 Hz, 2H), 7.19 (d, *J* = 8.6 Hz, 2H), 4.99 (dd, *J* = 12.3, 5.3 Hz, 1H), 4.62 (d, *J* = 12.0 Hz, 1H), 4.49 (d, *J* = 12.0 Hz, 1H), 4.15 (s, 1H), 2.96 – 2.88 (m, 1H), 2.87 – 2.63 (m, 2H), 2.20 – 2.11 (m, 1H), 1.55 (s, 3H), 1.38 (s, 3H).

^{13}C{^{1}H} NMR (151 MHz, $CDCl_3$, reported as a mixture of diastereomers) δ 170.8, 169.7, 168.0, 167.03, 167.02, 166.95, 144.7, 142.0, 136.44, 136.40, 131.7, 131.5, 131.1, 128.4, 125.31, 125.30, 123.4, 121.1, 94.4, 74.5, 62.3, 49.5, 41.8, 31.5, 26.40, 26.37, 25.2, 25.1, 22.7.

SFC analysis: Using **GP3**, **18** (CHIRALPAK® AD-3, 20% 1:1 MeOH:^{i}PrOH with 0.2% formic acid in CO_2, 2.5 mL/min, 210 nm) indicated 92:8 d.r. for the asymmetric induction observed at the newly formed stereogenic center: t_R (major diastereomers) = 2.4 and 2.7 min., t_R (minor diastereomers) = 4.4 and 15.0 min.

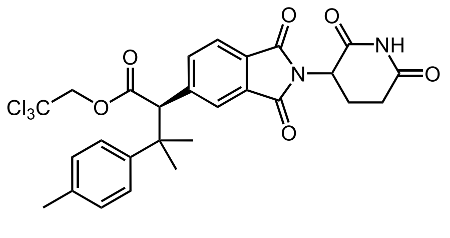

19

2,2,2-trichloroethyl (2*S*)-2-(2-(2,6-dioxopiperidin-3-yl)-1,3-dioxoisoindolin-5-yl)-3-methyl-3-(p-tolyl)butanoate (19).

Compound **19** was produced via **GP1** using **6a** (47 mg, 1.0 equiv, 0.10 mmol), 1,1,1,3,3,3-hexafluoroisopropanol (0.11 mL, 10.0 equiv, 1.00 mmol), Rh_2(*S*-*tetra*-*p*-BrPhPTTL)$_4$ (20 mM in CH_2Cl_2, 50 µL, 1 mol%, 1.0 µmol), and *p*-cymene (1.0 mL). Purification via flash column chromatography (SiO_2, 20% to 50% ethyl acetate in hexanes) produced afforded 2,2,2-trichloroethyl (2*S*)-2-(2-(2,6-

dioxopiperidin-3-yl)-1,3-dioxoisoindolin-5-yl)-3-methyl-3-(p-tolyl)butanoate **(19)** as an amorphous white solid (58 mg, 99 μmol, 99% yield).

Compound **19** was prepared via **GP3** using *p*-cymene (0.16 mL, 10.0 equiv, 1.0 mmol), Rh_2(*S*-*tetra*-*p*-BrPhPTTL)$_4$ (3.7 mg, 1 mol%, 1.0 μmol), **6a** (47 mg, 1.0 equiv, 0.10 mmol), 1,1,1,3,3,3-hexafluoropropan-2-ol (0.10 mL, 10.0 equiv, 1.0 mmol), and 1.0 mL CH_2Cl_2 at room temperature with a one hour slow addition of diazo and 0.5 h additional stirring. Excess *p*-cymene was removed from the reaction mixture via Kugelrohr distillation (100 °C, <10 torr, 10 min). Purification via flash column chromatography (SiO_2, gradient of 20% to 50% EtOAc in hexanes) afforded 2,2,2-trichloroethyl (2*S*)-2-(2-(2,6-dioxopiperidin-3-yl)-1,3-dioxoisoindolin-5-yl)-3-methyl-3-(p-tolyl)butanoate **(19)** as an amorphous white solid (31 mg, 53 μmol, 53% yield).

NMR spectra contaminated with ca. 10% primary insertion product. Yields reported as a combined yield of diastereomers and regioisomers.
HRMS (APCI) *m/z*: $[M+H]^+$ calcd for $C_{27}H_{26}O_6N_2{}^{35}Cl_3$ 579.0851; Found 579.0850
^{1}H NMR (400 MHz, $CDCl_3$, reported as a mixture of diastereomers) δ 8.05 (s, 1H), 7.89 (dd, *J* = 5.1, 1.4 Hz, 1H), 7.71 (dt, *J* = 7.8, 1.0 Hz, 1H), 7.47 (ddd, *J* = 7.7, 6.0, 1.6 Hz, 1H), 7.17 (d, *J* = 8.4 Hz, 2H), 7.10 (d, *J* = 8.6 Hz, 2H), 4.98 (dd, *J* = 12.2, 5.3 Hz, 1H), 4.62 (d, *J* = 12.0 Hz, 1H), 4.52 (d, *J* = 12.0 Hz, 1H), 4.17 (s, 1H), 2.96 – 2.87 (m, 1H), 2.88 – 2.67 (m, 2H), 2.31 (s, 3H), 1.55 (s, 3H), 1.39 (s, 3H).

^{13}C{^{1}H} NMR (151 MHz, $CDCl_3$, reported as a mixture of diastereomers) δ δ 171.02, 171.00, 169.9, 168.1, 167.13, 167.12, 167.09, 142.52, 142.50, 136.6, 136.43, 136.40, 131.5, 130.8, 129.1, 128.9, 126.9, 126.4, 125.33, 125.31, 123.2, 94.55, 94.48, 74.4, 62.6, 49.52, 49.46, 41.8, 31.5, 26.0, 25.6, 22.7, 21.0.

SFC analysis: Using **GP1**, **19** (Trefoil® AMY1, 20% 1:1 MeOH:^{i}PrOH with 0.2% formic acid in CO_2, 2.5 mL/min, 210 nm) indicated 78:22 d.r. for the asymmetric induction observed for the formation of the major regioisomer: t_R (major diastereomers) = 4.2 and 5.2 min., t_R (minor diastereomers) = 8.6 and 21.1 min. Using **GP3**, **19** indicated 90:10 d.r.

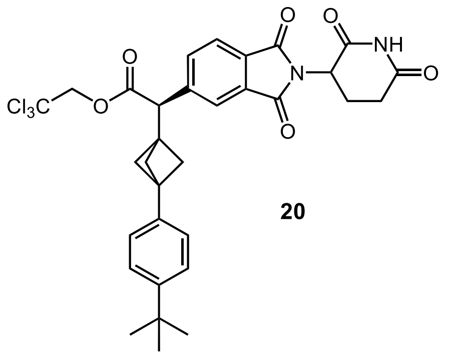

20

2,2,2-trichloroethyl (2*R*)-2-(3-(4-(*tert*-butyl)phenyl)bicyclo[1.1.1]pentan-1-yl)-2-(2-(2,6-dioxopiperidin-3-yl)-1,3-dioxoisoindolin-5-yl)acetate (20).

Compound **20** was prepared via **GP3** using 1-(4-(tert-butyl)phenyl)bicyclo[1.1.1]pentane (168 mg, 10.0 equiv, 1.0 mmol), Rh_2(*S*-*tetra*-*p*-BrPhPTTL)$_4$ (3.7 mg, 1 mol%, 1.0 μmol), **6a** (47 mg, 1.0 equiv, 0.10 mmol), 1,1,1,3,3,3-hexafluoropropan-2-ol (0.10 mL, 10.0 equiv, 1.0 mmol), and 1.0 mL CH_2Cl_2 at room temperature with a one hour slow addition of diazo and 0.5 h additional stirring. Purification via flash column chromatography (SiO_2, gradient of 20% to 50% EtOAc in hexanes) afforded 2,2,2-trichloroethyl (2*R*)-2-(3-(4-(*tert*-butyl)phenyl)bicyclo[1.1.1]pentan-1-yl)-2-(2-(2,6-dioxopiperidin-3-yl)-1,3-dioxoisoindolin-5-yl)acetate **(20)** as an amorphous white solid (40 mg, 64 μmol, 46% yield).

HRMS (APCI) *m/z*: $[M+H]^+$ calcd for $C_{32}H_{32}O_6N_2{}^{35}Cl_3$ 645.1321; Found 645.1323

^{1}H NMR (400 MHz, $CDCl_3$, reported as a mixture of diastereomers) δ 8.00 (s, 1H), 7.94 (s, 1H), 7.89 (d, *J* = 7.7 Hz, 1H), 7.77 (dt, *J* = 7.8, 1.6 Hz, 1H), 7.32 (d, *J* = 8.4 Hz, 1H), 7.10 (d, *J* = 8.3 Hz, 1H), 5.00 (dd, *J* = 12.2, 5.2 Hz, 1H), 4.87 (d, *J* = 12.0 Hz, 1H), 4.756 and 4.755 (dd, *J* = 12.0 Hz, 1H), 4.17 (s, 1H), 2.97 – 2.89 (m, 1H), 2.88 – 2.70 (m, 1H), 2.17 (ddd, *J* = 10.3, 5.1, 2.9 Hz, 1H), 2.08 – 1.99 (m, 6H), 1.29 (s, 9H).

^{13}C{^{1}H} NMR (151 MHz, $CDCl_3$, reported as a mixture of diastereomers) δ 170.9, 169.3, 168.0, 167.1, 167.0, 149.9, 143.4, 136.8, 135.02, 135.00, 132.3, 131.0, 125.8, 125.3, 124.2, 124.1, 94.6, 74.6, 53.6, 52.0, 49.5, 42.5, 39.5, 34.6, 31.53, 31.47, 22.8.

SFC analysis: Using **GP3**, **20** (CHIRALCEL® OX-3, 20% 1:1 MeOH:^{i}PrOH with 0.2% formic acid in CO_2, 2.5 mL/min, 210 nm) indicated 99:1 d.r. for the asymmetric induction observed at the newly formed stereogenic center: t_R (major diastereomers) = 10.6 and 11.8 min., t_R (minor diastereomers) = 10.3 and 14.0 min.

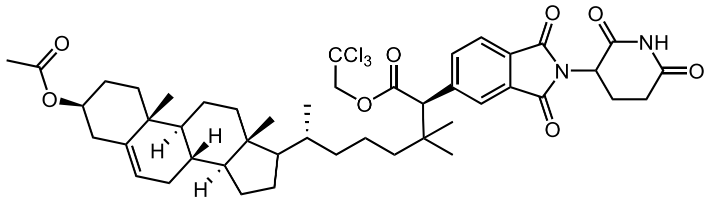

21

2,2,2-trichloroethyl (2*R*,7*R*)-7-((3*S*,8*S*,9*S*,10*R*,13*R*,14*S*)-3-acetoxy-10,13-dimethyl-2,3,4,7,8,9,10,11,12,13,14,15,16,17-tetradecahydro-1H-cyclopenta[*a*]phenanthren-17-yl)-2-(2-(2,6-dioxopiperidin-3-yl)-1,3-dioxoisoindolin-5-yl)-3,3-dimethyloctanoate (21).

Compound **21** was prepared via **GP3** using (3*S*,8*S*,9*S*,10*R*,13*R*,14*S*)-10,13-dimethyl-17-((*R*)-6-methylheptan-2-yl)-2,3,4,7,8,9,10,11,12,13,14,15,16,17-tetradecahydro-1H-cyclopenta[*a*]phenanthren-3-yl acetate (430 mg, 10.0 equiv, 1.0 mmol), Rh_2(*S*-*tetra*-*p*-BrPhPTTL)$_4$ (3.7 mg, 1 mol%, 1.0 μmol), **6a** (47 mg, 1.0 equiv, 0.10 mmol), 1,1,1,3,3,3-hexafluoropropan-2-ol (0.10 mL, 10.0 equiv, 1.0 mmol), and 1.0 mL CH_2Cl_2 at room temperature with a one hour slow addition of diazo and 0.5 h additional stirring. Purification via flash column chromatography (SiO_2, gradient of 20% to 50% EtOAc in hexanes) afforded 2,2,2-trichloroethyl (2*R*,7*R*)-7-((3*S*,8*S*,9*S*,10*R*,13*R*,14*S*)-3-acetoxy-10,13-dimethyl-2,3,4,7,8,9,10,11,12,13,14,15,16,17-tetradecahydro-1H-cyclopenta[*a*]phenanthren-17-yl)-2-(2-(2,6-dioxopiperidin-3-yl)-1,3-dioxoisoindolin-5-yl)-3,3-dimethyloctanoate **(21)** as an amorphous white solid (28 mg, 32 μmol, 32% yield).

HRMS (APCI) *m/z*: $[M+Na]^+$ calcd for $C_{46}H_{59}O_8N_2{}^{35}Cl_3{}^{23}Na$ 895.3229; Found 895.3244

^{1}H NMR (600 MHz, $CDCl_3$, reported as a mixture of diastereomers) δ 8.04 (s, 1H), 8.00 (d, *J* = 4.8 Hz, 1H), 7.89 – 7.79 (m, 2H), 5.37 (d, *J* = 5.1 Hz, 1H), 4.99 (dd, *J* = 12.6, 5.4 Hz, 1H), 4.84 (d, *J* = 12.0 Hz, 1H), 4.66 – 4.52 (m, 2H), 3.84 (s, 1H), 2.99 – 2.89 (m, 1H), 2.89 – 2.64 (m, 2H), 2.38-2.29 (m, 2H), 2.21 – 2.12 (m, 1H), 2.03 (s, 3H), 2.02 – 1.92 (m, 2H), 1.89-1.82 (m, 1H), 1.81 – 1.75 (m, 1H), 1.61 – 1.34 (m, 10H), 1.34 – 1.27 (m, 1H), 1.26 – 1.10 (m, 6H), 1.09 (s, 3H), 1.07 – 1.03 (m, 1H), 1.01 (s, 3H), 1.00 – 0.96 (m, 2H), 0.95 (s, 3H), 0.92 (d, *J* = 6.5 Hz, 3H), 0.67 (s, 3H).

^{13}C{^{1}H} NMR (151 MHz, $CDCl_3$, reported as a mixture of diastereomers) δ 170.9, 170.7, 170.5, 168.1, 167.2, 167.1, 143.1, 139.8, 136.41, 136.38, 131.7, 130.9, 125.6, 125.5, 123.4, 122.7, 94.7, 74.4, 74.1, 60.6, 56.8, 56.2, 50.1, 49.5, 42.5, 41.4, 39.8, 38.3, 37.1, 36.73, 36.71, 35.9, 32.01, 31.98, 31.5, 28.4, 27.9, 24.85, 24.83, 24.43, 24.41, 24.39, 22.8, 21.6, 21.2, 20.4, 19.4, 18.9, 12.0.

SFC analysis: Using **GP3**, **20** (CHIRALCEL® OX-3, 35% 1:1 MeOH:^{i}PrOH with 0.2% formic acid in CO_2, 2.5 mL/min, 230 nm) indicated 99:1 d.r. for the asymmetric induction observed at the newly formed

stereogenic center: t_R (major diastereomers) = 6.8 and 8.0 min., t_R (minor diastereomers) = 9.7 and 11.0 min.

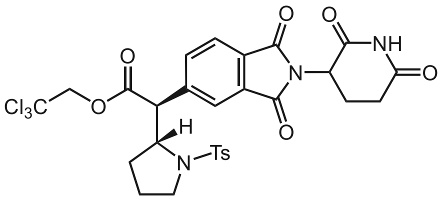

22

2,2,2-trichloroethyl (2*S*)-2-(2-(2,6-dioxopiperidin-3-yl)-1,3-dioxoisoindolin-5-yl)-2-((*R*)-1-tosylpyrrolidin-2-yl)acetate (22).

Compound **22** was prepared via **GP3** using 1-*N*-tosylpyrrolidine (0.23 g, 10.0 equiv, 1.0 mmol), Rh_2(*S*-*tetra*-*p*-BrPhPTTL)$_4$ (3.7 mg, 1 mol%, 1.0 µmol), **6a** (47 mg, 1.0 equiv, 0.10 mmol), 1,1,1,3,3,3-hexafluoropropan-2-ol (0.10 mL, 10.0 equiv, 1.0 mmol), and 1.0 mL CH_2Cl_2 at room temperature with a one hour slow addition of diazo and 0.5 h additional stirring. Purification via flash column chromatography (SiO_2, gradient of 10% to 65% EtOAc in hexanes) afforded 2,2,2-trichloroethyl (2*S*)-2-(2-(2,6-dioxopiperidin-3-yl)-1,3-dioxoisoindolin-5-yl)-2-((*R*)-1-tosylpyrrolidin-2-yl)acetate (**22**) as an amorphous white solid (36 mg, 53 µmol, 53% yield).

Compound **12** was prepared via **GP8** using 1-*N*-tosylpyrrolidine (0.23 g, 10.0 equiv, 1.0 mmol), Rh_2(*S*-*tetra*-*p*-BrPhPTTL)$_4$ (20 mM in CH_2Cl_2, 50 µL, 1 mol%, 1.0 µmol), **6a** (47 mg, 1.0 equiv, 0.10 mmol), and 1.0 mL CH_2Cl_2 at room temperature for 0.5 h. Purification via flash column chromatography (SiO_2, gradient of 10% to 65% EtOAc in hexanes) afforded 2,2,2-trichloroethyl (2*S*)-2-(2-(2,6-dioxopiperidin-3-yl)-1,3-dioxoisoindolin-5-yl)-2-((*R*)-1-tosylpyrrolidin-2-yl)acetate (**22**) as an amorphous white solid (57 mg, 85 µmol, 85% yield).

HRMS (APCI): *m/z* $([M+H]^+)$ calcd for $C_{29}H_{32}O_6N_2{}^{35}Cl_3$ 670.0579; Found 670.0579

^{1}H NMR (400 MHz, $CDCl_3$, reported as a mixture of diastereomers) δ 8.14 (s, 1H), 7.98 – 7.90 (m, 1H), 7.92 – 7.78 (m, 3H), 7.64 (d, *J* = 8.2 Hz, 2H), 7.30 (d, *J* = 8.0 Hz, 2H), 5.00 (dd, *J* = 12.2, 5.2 Hz, 1H), 4.83 – 4.79 (m, 2H), 4.37 (m, 1H), 4.27 (d, *J* = 6.8 Hz, 1H), 3.42 – 3.34 (m, 1H), 3.32 – 3.18 (m, 1H), 2.99 – 2.63 (m, 3H), 2.41 (s, 3H), 2.23 – 2.12 (m, 1H), 1.99 – 1.88 (m, 1H), 1.77 – 1.63 (m, 1H), 1.51 – 1.33 (m, 1H).

^{13}C{^{1}H} NMR (101 MHz, $CDCl_3$, reported as a mixture of diastereomers) δ 171.0, 169.35 and 169.33, 168.1, 167.04 and 166.99, 144.2, 144.1, 142.1, 136.0, 134.6, 134.5, 132.1, 131.4, 130.0, 127.7, 127.6, 125.0, 124.0, 94.6, 74.6, 62.94 and 62.92, 56.0, 49.5, 49.4, 31.5, 29.8, 24.2, 22.7, 21.7.

SFC analysis: **22** (Trefoil® AMY1, 30% 1:1 MeOH:^{i}PrOH with 0.2% formic acid in CO_2, 1.5 mL/min, 210 nm) indicated 97:3 d.r. for asymmetric induction arising from formation of the major relative diastereomer using **GP3**: t_R (major diastereomers) = 11.2 and 20.7 min., t_R (minor diastereomers) = 10.0 and 13.6 min. **GP8** gave **22** in 95:5 d.r.

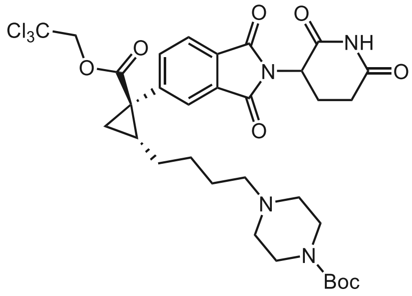

24

***tert*-butyl 4-(4-((1*R*,2*R*)-2-(2-(2,6-dioxopiperidin-3-yl)-1,3-dioxoisoindolin-5-yl)-2-((2,2,2-trichloroethoxy)carbonyl)cyclopropyl)butyl)piperazine-1-carboxylate (24).**

Compound **4** was prepared via **GP5** using **13** (30 mg, 1.1 equiv, 0.11 mmol), Rh_2(*S*-*tetra*-*p*-BrPhPTTL)$_4$ (3.7 mg, 1 mol%, 1.0 μmol), **6a** (47 mg, 1.0 equiv, 0.10 mmol), 1,1,1,3,3,3-hexafluoropropan-2-ol (0.20 mL, 20.0 equiv, 2.0 mmol), and 1.0 mL CH_2Cl_2 at room temperature with a 0.5 h slow addition of diazo and 0.25 h additional stirring. Purification via flash column chromatography (SiO_2, gradient of 50% to 100% EtOAc in hexanes) afforded tert-butyl 4-(4-((1*R*,2*R*)-2-(2-(2,6-dioxopiperidin-3-yl)-1,3-dioxoisoindolin-5-yl)-2-((2,2,2-trichloroethoxy)carbonyl)cyclopropyl)butyl)piperazine-1-carboxylate **(24)** as an amorphous white solid (35 mg, 49 μmol, 49% yield).

Compound **24** was prepared via **GP8** using **13** (30 mg, 1.1 equiv, 0.11 mmol), Rh_2(*S*-*tetra*-*p*-BrPhPTTL)$_4$ (3.7 mg, 1 mol%, 1.0 μmol), **6a** (47 mg, 1.0 equiv, 0.10 mmol), and 1.0 mL CH_2Cl_2 at room temperature with a 0.5 h slow addition of diazo and 0.25 h additional stirring. Purification via flash column chromatography (SiO_2, gradient of 50% to 100% EtOAc in hexanes) afforded tert-butyl 4-(4-((1*R*,2*R*)-2-(2-(2,6-dioxopiperidin-3-yl)-1,3-dioxoisoindolin-5-yl)-2-((2,2,2-trichloroethoxy)carbonyl)cyclopropyl)butyl)piperazine-1-carboxylate **(24)** as an amorphous white solid (22 mg, 30 μmol, 30% yield).

HRMS (ESI): *m/z* $[M+H]^+$ calcd for $C_{32}H_{40}O_8N_4{}^{35}Cl_3$ 713.1906; Found 713.1904.

^{1}H NMR (400 MHz, $CDCl_3$, reported as a mixture of diastereomers) δ 8.25 and 8.14 (s, 1H), 7.92 – 7.83 (m, 1H), 7.83 – 7.79 (m, 1H), 7.72 (dd, *J* = 7.7, 1.5 Hz, 1H), 4.990 and 4.987 (dd, *J* = 12.5, 5.3 Hz, 1H), 4.81 and 4.80 (d, *J* = 11.9 Hz, 1H), 4.58 and 4.57 (d, *J* = 11.9 Hz, 1H), 3.48 – 3.41 (m, 4H), 3.00 – 2.89 (m, 1H), 2.88 – 2.71 (m, 2H), 2.42 – 2.32 (m, 5H), 2.20 – 2.13 (m, 1H), 2.09 – 1.94 (m, 2H), 1.68 – 1.36 (m, 7H, overlapping with s at 1.44) 1.44 (s, 9H, overlapping with multiplet from 1.68-1.36), 1.32 (dd, *J* = 6.6, 4.3 Hz, 1H).

^{13}C{^{1}H} NMR (151 MHz, $CDCl_3$, reported as a mixture of diastereomers) δ 171.6, 170.92, 170.91, 168.2, 168.1, 167.2, 167.1, 154.8, 143.2, 137.9, 131.93, 131.92, 130.88, 130.86, 126.71, 126.68, 123.7, 123.6, 94.9, 74.5, 58.4, 53.0, 49.5, 33.9, 33.8, 31.6, 30.6, 30.5, 30.1, 28.6, 27.0, 22.8, 22.32, 22.27.

SFC analysis: **24** (CHIRALPAK® AD-3, 30% 1:1 MeOH:^{i}PrOH with 0.2% formic acid in CO_2, 1.5 mL/min, diode array) indicated 96:4 d.r. for asymmetric induction arising from formation of the major relative diastereomer using **GP5**: t_R (major diastereomer) = 4.5 min., t_R (minor diastereomer) = 2.1 min., t_R (inseparable major + minor) = 1.7 min. **GP8** gave **24** in 97:3 d.r.

Note: The other two diastereomers represented by the peak at 1.7 min were inseparable, and the d.r. was acquired from the diastereomers at 2.1 and 4.5 min. only.

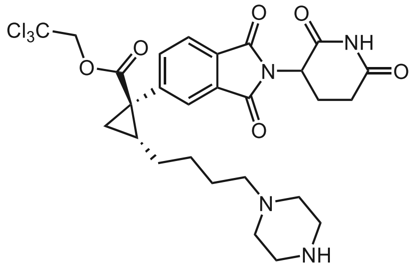

SI1

2,2,2-trichloroethyl (1*R*,2*R*)-1-(2-(2,6-dioxopiperidin-3-yl)-1,3-dioxoisoindolin-5-yl)-2-(4-(piperazin-1-yl)butyl)cyclopropane-1-carboxylate (SI1).

Compound **24** (27 mg, 38 µmol, 1.0 equiv) was added to a vial equipped with a PTFE magnetic stir bar under ambient conditions, and 0.5 mL CH_2Cl_2 were added. The vial was cooled to 0 °C in an ice bath, and 0.5 mL trifluoroacetic acid was added in one portion. The ice bath was removed and the reaction was allowed to come to room temperature, stirring for one hour. The reaction was concentrated in vacuuo and the residual trifluoroacetic acid was removed by concentration from toluene (3x). The obtained residue was dissolved in ethyl acetate (5 mL) and washed twice with a saturated aqueous solution of sodium bicarbonate (5 mL). The organic layer was dried over sodium sulfate, filtered, and concentrated under vacuum. The crude 2,2,2-trichloroethyl (1*R*,2*R*)-1-(2-(2,6-dioxopiperidin-3-yl)-1,3-dioxoisoindolin-5-yl)-2-(4-(piperazin-1-yl)butyl)cyclopropane-1-carboxylate **(SI1)** was carried forward to the next step without further purification.

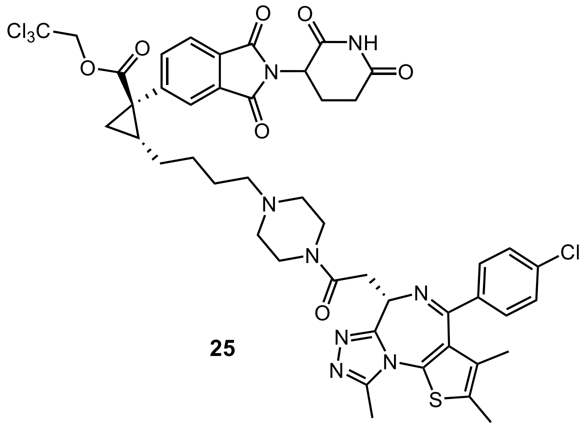

25

2,2,2-trichloroethyl (1*R*,2*R*)-2-(4-(4-(2-((*S*)-4-(4-chlorophenyl)-2,3,9-trimethyl-6H-thieno[3,2-f][1,2,4]triazolo[4,3-a][1,4]diazepin-6-yl)acetyl)piperazin-1-yl)butyl)-1-(2-(2,6-dioxopiperidin-3-yl)-1,3-dioxoisoindolin-5-yl)cyclopropane-1-carboxylate (25).

A flame-dried vial equipped with a PTFE magnetic stir bar and under inert atmosphere was charged with (*S*)-2-(4-(4-chlorophenyl)-2,3,9-trimethyl-6H-thieno[3,2-f][1,2,4]triazolo[4,3-a][1,4]diazepin-6-yl)acetic acid (9.0 mg, 22 µmol, 1.0 equiv) and HATU (17 mg, 45 µmol, 2.0 equiv). Dry DMF (0.5 mL) was charged by syringe along with DIPEA (12 µL, 67 µmol, 3.0 equiv). The reaction was stirred at room temperature for 0.5 hours, after which a solution of **SI1** (15 mg, 25 µmol, 1.1 equiv) in 0.5 mL dry DMF was charged by syringe. The reaction was allowed to stir for 4 hours at room temperature, after which it was partitioned between saturated aqueous sodium chloride solution (5 mL) and ethyl acetate (5 mL). The aqueous layer was extracted thrice with ethyl acetate (5 mL portions), after which the combined organic layers were washed with saturated aqueous sodium chloride solution (5 mL). The combined organic layers were dried

over sodium sulfate, filtered, and concentrated in vacuuo. Flash column chromatography (SiO_2, 5% to 10% MeOH in CH_2Cl_2) afforded 2,2,2-trichloroethyl (1*R*,2*R*)-2-(4-(4-(2-((*S*)-4-(4-chlorophenyl)-2,3,9-trimethyl-6H-thieno[3,2-f][1,2,4]triazolo[4,3-a][1,4]diazepin-6-yl)acetyl)piperazin-1-yl)butyl)-1-(2-(2,6-dioxopiperidin-3-yl)-1,3-dioxoisoindolin-5-yl)cyclopropane-1-carboxylate **(25)** as a tan amorphous solid (16 mg, 22 μmol, 72% yield).

HRMS (ESI) m/z: $[M+H]^+$ calcd for $C_{46}H_{47}O_7N_8{}^{35}Cl_4{}^{32}S$ 995.2037; Found 995.2065

^{1}H NMR (800 MHz, DMSO-*d*6, sample at 80 °C, reported as a mixture of diastereomers) δ 10.87 (s, 1H), 7.90 (t, *J* = 7.6 Hz, 1H), 7.88 (d, *J* = 1.4 Hz, 1H), 7.86 (dt, *J* = 7.7, 1.3 Hz, 1H), 7.46 (d, *J* = 9.0 Hz, 2H), 7.44 (d, *J* = 8.6 Hz, 2H), 5.13 and 5.12 (dd, *J* = 12.6, 5.6 Hz, 1H), 4.90 (d, *J* = 12.3 Hz, 1H), 4.791 and 4.790 (d, *J* = 12.3 Hz, 1H), 4.59 (t, *J* = 6.7 Hz, 1H), 3.62 – 3.55 (m, 5H), 3.398 and 3.396 (dd, *J* = 16.1, 6.5 Hz, 1H), 2.88 (ddt, *J* = 17.0, 13.4, 5.3 Hz, 1H), 2.66 – 2.54 (m, 3 H), 2.60 (s, 3H, overlapping with m from 2.66-2.54), 2.53 – 2.50 (m, 1H, overlapping with solvent signal at 2.50), 2.42 (s, 3H), 2.41 – 2.15 (m, 5H), 2.13 – 2.09 (m, 1H), 2.05 – 1.99 (m, 1H), 1.87 (dd, *J* = 9.1, 4.8 Hz, 1H), 1.65 (s, 3H), 1.58 (dd, *J* = 7.1, 4.9 Hz, 1H), 1.46 – 1.38 (m, 5H).

^{13}C{^{1}H} NMR (201 MHz, DMSO-*d*6, sample at 80 °C, reported as a mixture of diastereomers) δ 171.9, 170.7, 169.1, 166.5, 166.4, 155.0, 149.2, 142.4, 137.3, 136.6, 134.9, 131.8, 130.9, 130.3, 129.9, 129.7, 129.55, 129.46, 128.0, 125.7, 122.5, 95.0, 73.6, 73.4, 57.1, 54.0, 48.9, 34.4, 33.1, 30.6, 30.1, 29.2, 29.0, 25.9, 21.70, 21.68, 21.0, 13.4, 12.23, 12.01, 10.74, 10.72.

Purity (HPLC) 97%

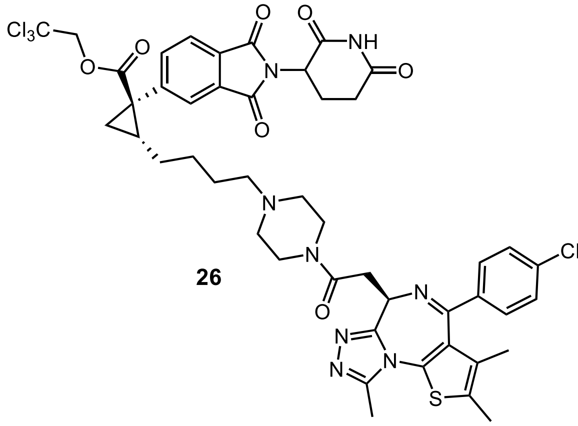

26

2,2,2-trichloroethyl (1*R*,2*R*)-2-(4-(4-(2-((*R*)-4-(4-chlorophenyl)-2,3,9-trimethyl-6H-thieno[3,2-f][1,2,4]triazolo[4,3-a][1,4]diazepin-6-yl)acetyl)piperazin-1-yl)butyl)-1-(2-(2,6-dioxopiperidin-3-yl)-1,3-dioxoisoindolin-5-yl)cyclopropane-1-carboxylate (26).

A flame-dried vial equipped with a PTFE magnetic stir bar and under inert atmosphere was charged with (*R*)-2-(4-(4-chlorophenyl)-2,3,9-trimethyl-6H-thieno[3,2-f][1,2,4]triazolo[4,3-a][1,4]diazepin-6-yl)acetic acid (12 mg, 30 μmol, 1.0 equiv) and HATU (23 mg, 60 μmol, 2.0 equiv). Dry DMF (0.5 mL) was charged by syringe along with DIPEA (16 μL, 90 μmol, 3.0 equiv). The reaction was stirred at room temperature for 0.5 hours, after which a solution of **SI1** (20 mg, 33 μmol, 1.1 equiv) in 0.5 mL dry DMF was charged by syringe. The reaction was allowed to stir for 4 hours at room temperature, after which it was partitioned between saturated aqueous sodium chloride solution (5 mL) and ethyl acetate (5 mL). The aqueous layer was extracted thrice with ethyl acetate (5 mL portions), after which the combined organic layers were washed with saturated aqueous sodium chloride solution (5 mL). The combined organic layers were dried over sodium sulfate, filtered, and concentrated in vacuuo. Flash column chromatography (SiO_2, 5% to 10% MeOH in CH_2Cl_2) afforded 2,2,2-trichloroethyl (1*R*,2*R*)-2-(4-(4-(2-((*R*)-4-(4-chlorophenyl)-2,3,9-

trimethyl-6H-thieno[3,2-f][1,2,4]triazolo[4,3-a][1,4]diazepin-6-yl)acetyl)piperazin-1-yl)butyl)-1-(2-(2,6-dioxopiperidin-3-yl)-1,3-dioxoisoindolin-5-yl)cyclopropane-1-carboxylate **(26)** as a tan amorphous solid (14 mg, 30 µmol, 47% yield).

HRMS (ESI) *m/z*: $[M+H]^+$ calcd for $C_{46}H_{47}O_7N_8{}^{35}Cl_4{}^{32}S$ 995.2037; Found 995.2049
^{1}H NMR (800 MHz, DMSO-*d*6, sample at 80 °C, reported as a mixture of diastereomers) δ 10.86 (s, 1H), 7.90 (d, *J* = 7.7 Hz, 2H), 7.88 (d, *J* = 1.5 Hz, 2H), 7.86 (dt, *J* = 7.7, 1.4 Hz, 2H), 7.46 (d, *J* = 8.8 Hz, 3H), 7.44 (d, *J* = 8.7 Hz, 4H), 5.13 and 5.12 (dd, *J* = 12.6, 5.5 Hz, 1H), 4.90 (d, *J* = 12.3 Hz, 1H), 4.792 and 4.790 (d, *J* = 12.2 Hz, 1H), 4.59 (t, *J* = 6.7 Hz, 1H), 3.68-3.43 (m, 4H, overlapped with dd at 3.57), 3.57 (dd, *J* = 16.0, 6.8 Hz, 1H), 3.40 and 3.39 (dd, *J* = 16.1, 6.5 Hz, 1H), 2.88 (ddt, *J* = 17.0, 13.4, 5.3 Hz, 1H), 2.65 – 2.55 (m, 3H, overlapped with s at 2.60), 2.60 (s, 3H, overlapped with m from 2.65-2.55), 2.53 – 2.49 (m, 1H, partially overlapped with solvent signal), 2.42 (s, 3H), 2.41 – 2.16 (m, 5H), 2.14 – 2.09 (m, 1H), 2.06 – 2.00 (m, 1H), 1.87 (dd, *J* = 9.1, 4.8 Hz, 1H), 1.66 (s, 3H), 1.58 (dd, *J* = 7.1, 4.9 Hz, 1H), 1.44 – 1.34 (m, 5H).
^{13}C{^{1}H} NMR (201 MHz, DMSO-*d*6, sample at 80 °C, reported as a mixture of diastereomers) δ 171.9, 170.7, 169.1, 167.8, 166.5, 166.4, 162.4, 155.0, 149.2, 142.4, 137.3, 136.6, 134.9, 131.8, 130.9, 130.3, 129.9, 129.7, 129.54, 129.46, 128.0, 125.8, 122.5, 95.0, 73.4, 57.1, 54.0, 48.9, 34.4, 33.1, 30.6, 30.1, 29.2, 29.04, 28.96, 25.9, 25.4, 21.70, 21.68, 21.0, 13.4, 12.2, 10.7.
Purity (HPLC) 95%

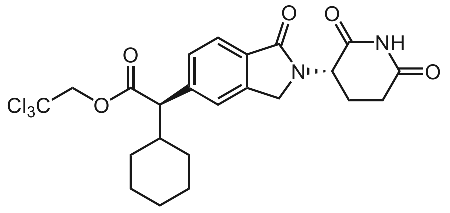

SI2

2,2,2-trichloroethyl (*R*)-2-cyclohexyl-2-(2-((*S*)-2,6-dioxopiperidin-3-yl)-1-oxoisoindolin-5-yl)acetate (SI2).

Compound **SI2** was produced following **GP 7**, using using **10a** (21 mg, 1.0 equiv, 34 µmol) and benzenesulfonic acid (11 mg, 2.0 equiv, 68 µmol) in acetonitrile (0.17 mL) at 70 °C for 3.5 h. Purification via flash column chromatography (SiO_2, 70% ethyl acetate in hexanes) gave 2,2,2-trichloroethyl (*R*)-2-cyclohexyl-2-(2-((*S*)-2,6-dioxopiperidin-3-yl)-1-oxoisoindolin-5-yl)acetate **(SI2)** as an amorphous white solid (13 mg, 73% yield, 34 µmol).

HRMS (APCI) *m/z*: $[M+H]^+$ calcd for $C_{23}H_{26}O_5N_2{}^{35}Cl_3$ 515.0902; Found 515.0902
^{1}H NMR (600 MHz, $CDCl_3$) δ 7.98 (s, 1H), 7.84 (d, *J* = 7.9 Hz, 1H), 7.53 (s, 1H), 7.47 (d, *J* = 7.9 Hz, 1H), 5.23 (dd, *J* = 13.4, 5.1 Hz, 1H), 4.80 (d, *J* = 12.0 Hz, 1H), 4.62 (d, *J* = 12.0 Hz, 1H), 4.50 (d, *J* = 15.9 Hz, 1H), 4.33 (d, *J* = 15.9 Hz, 1H), 3.51 (d, *J* = 10.6 Hz, 1H), 2.98 – 2.89 (m, 1H), 2.84 (ddd, *J* = 18.1, 13.4, 5.4 Hz, 1H), 2.36 (qd, *J* = 13.2, 4.6 Hz, 1H), 2.25 – 2.19 (m, 1H), 2.17 – 2.08 (m, 1H), 1.93 – 1.86 (m, 1H), 1.81 – 1.75 (m, 1H), 1.73 – 1.61 (m, 2H), 1.37 – 1.28 (m, 2H), 1.20 – 1.09 (m, 3H), 0.85 – 0.75 (m, 1H).
^{13}C{^{1}H} NMR (101 MHz, $CDCl_3$) δ 171.6, 171.1, 169.5, 169.1, 141.0, 138.7, 135.1, 132.5, 130.6, 128.3, 128.2, 127.2, 126.7, 123.6, 95.1, 74.5, 51.9, 46.9, 37.3, 34.4, 31.6, 23.5, 20.4.
SFC analysis: Using **GP1**, **9b** (CHIRALCEL® OZ-3, 20% 1:1 MeOH:^{i}PrOH with 0.2% formic acid in CO_2, 2.5 mL/min, 210 nm) indicated 99:1 d.r. for retained asymmetric induction arising from formation

of the major relative diastereomer and 99:1 d.r. for major relative configuration of the glutarimide stereogenic center: t_R (major diastereomer) = 6.7 min., t_R (minor diastereomers) = 7.2, 15.0, and 15.8min.
Specific Rotation: $[\alpha]_D^{24}$ -7.4 (*c* 1.0, $CHCl_3$)

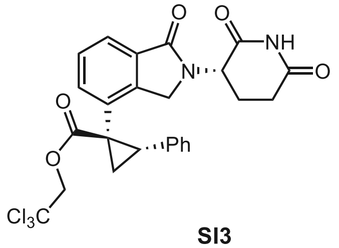

SI3

2,2,2-trichloroethyl (1*R*,2*S*)-1-(2-((*S*)-2,6-dioxopiperidin-3-yl)-1-oxoisoindolin-4-yl)-2-phenylcyclopropane-1-carboxylate (SI3).

Compound **SI3** was produced following **GP 7**, using using **12b** (15 mg, 1.0 equiv, 25 µmol) and benzenesulfonic acid (7.8 mg, 2.0 equiv, 50 µmol) in acetonitrile (0.12 mL) at 70 °C for 3.5 h. Purification via flash column chromatography (SiO_2, 70% ethyl acetate in hexanes) gave 2,2,2-trichloroethyl (1*R*,2*S*)-1-(2-((*S*)-2,6-dioxopiperidin-3-yl)-1-oxoisoindolin-4-yl)-2-phenylcyclopropane-1-carboxylate **(SI3)** as an amorphous white solid (7.5 mg, 57% yield, 25 µmol).

HRMS (APCI) *m/z*: $[M+H]^+$ calcd for $C_{25}H_{22}O_5N_2{}^{35}Cl_3$ 535.0589; Found 535.0591
^{1}H NMR (400 MHz, $CDCl_3$) δ 7.91 (s, 1H), 7.80 – 7.71 (m, 1H), 7.48 – 7.34 (m, 2H), 7.18 – 7.02 (m, 3H), 6.79 – 6.66 (m, 2H), 5.02 (dd, *J* = 13.2, 5.1 Hz, 1H), 4.87 (d, *J* = 11.9 Hz, 1H), 4.57 (d, *J* = 12.0 Hz, 1H), 4.29 (d, *J* = 16.0 Hz, 1H), 3.70 (d, *J* = 16.0 Hz, 1H), 3.25 (dd, *J* = 9.6, 7.4 Hz, 1H), 2.93 – 2.82 (m, 1H), 2.81 – 2.69 (m, 1H), 2.38 (dd, *J* = 9.6, 5.1 Hz, 1H), 2.23 (qd, *J* = 13.1, 4.8 Hz, 1H), 2.13 – 2.06 (m, 1H), 2.02 (dd, *J* = 7.5, 5.2 Hz, 1H).
^{13}C{^{1}H} NMR (101 MHz, $CDCl_3$) δ 171.1, 170.9, 169.0, 168.7, 143.7, 135.3, 134.7, 131.7, 129.5, 128.5, 128.3, 127.6, 127.4, 124.0, 94.9, 74.6, 51.8, 46.7, 35.1, 34.2, 31.6, 23.5, 21.4.
SFC analysis: **SI3** (CHIRALCEL® OZ-3, 20% 1:1 MeOH:^{i}PrOH with 0.2% formic acid in CO_2, 2.5 mL/min, 210 nm) indicated 93:7 d.r. for retained asymmetric induction arising from formation of the major relative diastereomer and 99:1 d.r. for major relative configuration of the glutarimide stereogenic center: t_R (major diastereomer) = 6.30 min., t_R (minor diastereomers) = 7.00, 12.94, and 20.28 min.
Specific Rotation: $[\alpha]_D^{24}$ -11.6 (*c* 0.5, $CHCl_3$)

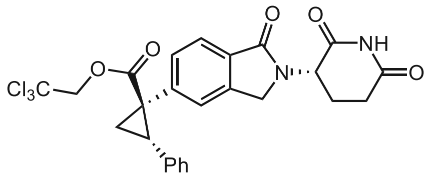

SI4

2,2,2-trichloroethyl (1*R*,2*S*)-1-(2-((*S*)-2,6-dioxopiperidin-3-yl)-1-oxoisoindolin-5-yl)-2-phenylcyclopropane-1-carboxylate (SI4).

Compound **SI4** was produced following **GP 7**, using using **12a** (15 mg, 1.0 equiv, 25 µmol) and benzenesulfonic acid (7.8 mg, 2.0 equiv, 50 µmol) in acetonitrile (0.12 mL) at 70 °C for 3.5 h. Purification via flash column chromatography (SiO_2, 65% ethyl acetate in hexanes) gave 2,2,2-

trichloroethyl (1*R*,2*S*)-1-(2-((*S*)-2,6-dioxopiperidin-3-yl)-1-oxoisoindolin-5-yl)-2-phenylcyclopropane-1-carboxylate **(SI4)** as an amorphous white solid (7.9 mg, 62% yield, 25 μmol).

HRMS (APCI): *m/z* $[M+H]^+$ calcd for $C_{25}H_{22}O_5N_2{}^{35}Cl_3$ 535.0589; Found 535.0592
^{1}H NMR (400 MHz, $CDCl_3$) δ 8.01 (s, 1H), 7.66 (d, *J* = 7.9 Hz, 1H), 7.24 (d, *J* = 8.1 Hz, 1H), 7.15 (s, 1H), 7.11 – 7.06 (m, 3H), 6.83 – 6.79 (m, 2H), 5.16 (dd, *J* = 13.3, 5.2 Hz, 1H), 4.86 (d, *J* = 11.9 Hz, 1H), 4.64 (d, *J* = 11.9 Hz, 1H), 4.29 (d, *J* = 15.9 Hz, 1H), 4.21 (d, *J* = 16.0 Hz, 1H), 3.29 (dd, *J* = 9.4, 7.5 Hz, 1H), 2.94 – 2.87 (m, 1H), 2.80 (m, 1H), 2.36 (dd, *J* = 9.4, 5.2 Hz, 1H), 2.29 (qd, *J* = 13.0, 4.9 Hz, 1H), 2.21 – 2.14 (m, 1H), 2.06 (dd, *J* = 7.5, 5.3 Hz, 1H).
^{13}C{^{1}H} NMR (101 MHz, $CDCl_3$) δ 171.6, 171.1, 169.5, 169.1, 141.0, 138.7, 135.1, 132.5, 130.6, 128.3, 128.2, 127.2, 126.7, 123.6, 95.1, 74.5, 51.9, 46.9, 37.3, 34.4, 31.6, 23.5, 20.4.
SFC analysis: **SI4** (CHIRALPAK® AS-3, 20% 1:1 MeOH:^{i}PrOH with 0.2% formic acid in CO_2, 2.5 mL/min, 210 nm) indicated 91:9 d.r. for retained asymmetric induction arising from formation of the major relative diastereomer and 97:3 d.r. for major relative configuration of the glutarimide stereogenic center: t_R (major diastereomer) = 2.50 min., t_R (minor diastereomers) = 1.92 and 2.84 min.
Specific Rotation: $[\alpha]_D^{24}$ 2.3 (*c* 0.5, $CHCl_3$)

Section 3: Computational Details

All calculations were performed using Gaussian-16 suite of programs.[8] Images of 3D structures were rendered using VMD[9] and Vesta.[10] Geometry and vibrational frequencies of the presented $Rh_2(OAc)_4$-carbene complexes were calculated at the B3LYP-D3(BJ)[11] level of theory in conjunction with Lanl2dz[12] basis set for rhodium and 6-31G(d,p)[13] basis set for other atoms. Geometry and vibrational frequencies of the Rh_2(*S-tetra-p-Br*PhPTTL)$_4$ systems with approximately 400 atoms and their carbene complexes were calculated by using the two-layer ONIOM[14] approach via partitioning of the complex Rh_2(*S-tetra-p*-BrPhPTTL)$_4$ into the two layers. (**Figure 1**) The highlighted catalyst structure and Rh-coordinated carbene fragment were treated at the B3LYP level of theory in conjunction with Lanl2dz basis set for rhodium and 6-31G(d,p) basis set for other atoms. The real system with all atoms from the catalyst and upcoming carbene fragment was calculated by using the molecular mechanics UFF[15] approach. The resulting approach is called the ONIOM(B3LYP:UFF) approach. The solvent effect (the CH_2Cl_2 was chosen as a solvent) was incorporated in all presented calculations by using the IFF-PCM method.[16] Frequency analyses and Gibbs free energy and zero-point energy corrections were calculated at a temperature and pressure corresponding to standard reaction conditions (i.e. at the 298.15K and 1 atm, respectively).

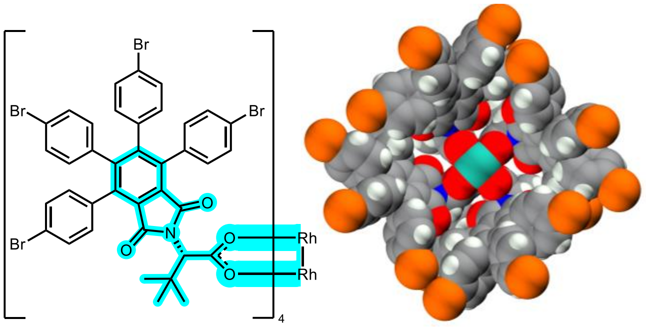

Figure S1. ONIOM Partitioning and the solid-state Structure of studied catalyst. The blue-highlighted atoms were modeled with QM layer (B3LYP), the rest was modeled with MM layer (UFF).

1. Analysis of the metal-carbene complex with $Rh_2(OAc)_4$

As the aryl fragment of the carbene moiety is unsymmetrical, there are four stereoisomers that will be formed upon the formation of the carbene complex due to the hinder rotation of the ester group and the aryl groups. As the studied catalyst is achiral and the stereocenter on the diazo is very far away from the ester group, it is expected that the diastereomer isomers resulted from the rotation of the carbene-ester bond will have comparable energy. In contrast, the rotation around the carbene-aryl bond would result in a significant energy difference as the configuration **II** (**SI-2**) is thermodynamically more stable than **I** (**SI-1**) by 2.6 kcal/mol.

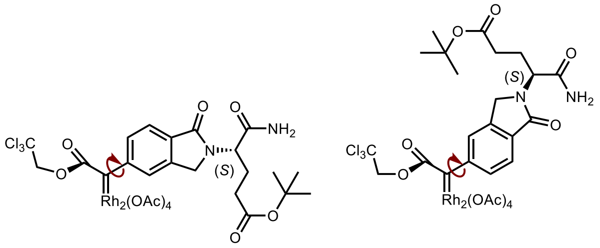

Rh2(OAc)4-Carbene-I
SI-1
$\Delta G = 0$ (kcal/mol)

$Rh_2(OAc)_4$-Carbene-II
SI-2
$\Delta G = -2.6$ (kcal/mol)

Figure S2. Study of $Rh_2(OAc)_4$-carbene complexes with isomers resulted from the rotation around the carbene-aryl bond.

2. Validation for ONIOM approach

The ONIOM approach can divide the study system into a QM level layer and an MM level layer. Increasing the size of the MM layer will help save computational cost but also imposed the loss of information especially weal interaction. Therefore, a comparison between structures obtaining from two layering of the ONIOM approach (**A** and **A-SI-1)** and the optimized structure from fully QM level (**A-SI-2**). The structure of model metal-carbene is displaced in **Figure S3** and its optimized structure is displaced in **Figure S4**. The significant difference between the two ONIOM layering methods and the full QM level is the rhodium-carbene length. As the ONIOM low level layer become bigger, the rhodium-carbene bond length becomes slightly longer by 0.01 Å and 0.04 Å, respectively. However, the optimized structures from ONIOM approach showed a good agreement with the optimized structure obtained from QM level with Root-Mean-Squares Deviation (RMSD) was only 0.65 Å and 0.94 Å. (**Figure S5**) This comparison showed that the ONIMO approach showed a fairly good correlation with the full QM level. As a result, the first layering approach to structure **A** was selected for the rest of this study.

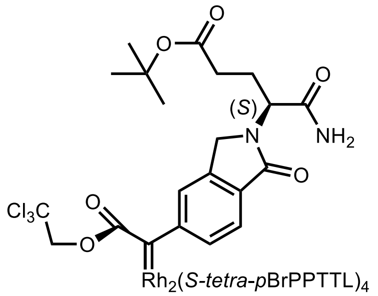

Figure S3. Structure of metal-carbene model study for Rh_2(*S*-*tetra*-p-*Br*PhPTTL)$_4$

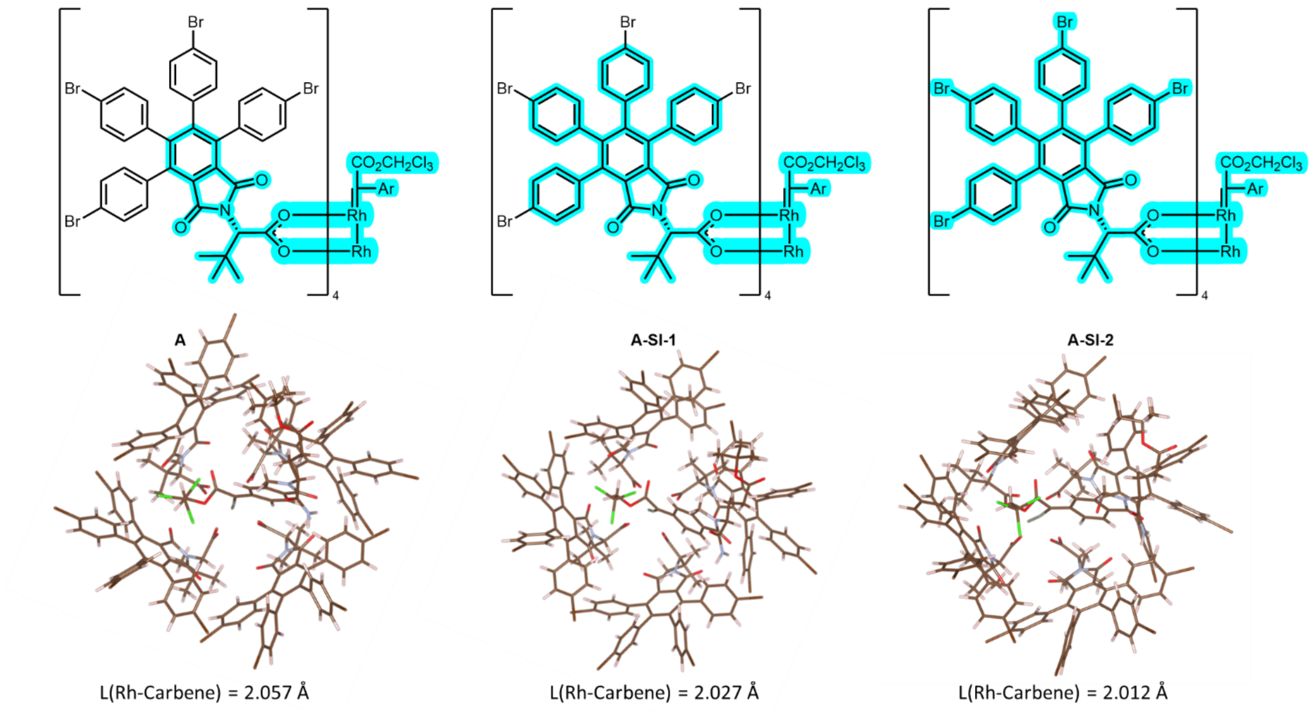

Figure S4. Optimized structure of metal-carbene complex. **A**. structure was optimized at ONIOM(B3LYP:UFF) with highlighted structure included in QM level while the rest in MM level. **B.** structure was optimized at ONIOM(B3LYP:UFF) with highlighted structure included in QM level while the rest in MM level. **C**. Structure was optimized at B3LYP-D3(BJ)/6-31G(d,p) (C,H,N,O,Br,Cl) – Lan2ldz (Rh). The reported bond length is Rhodium-carbene length

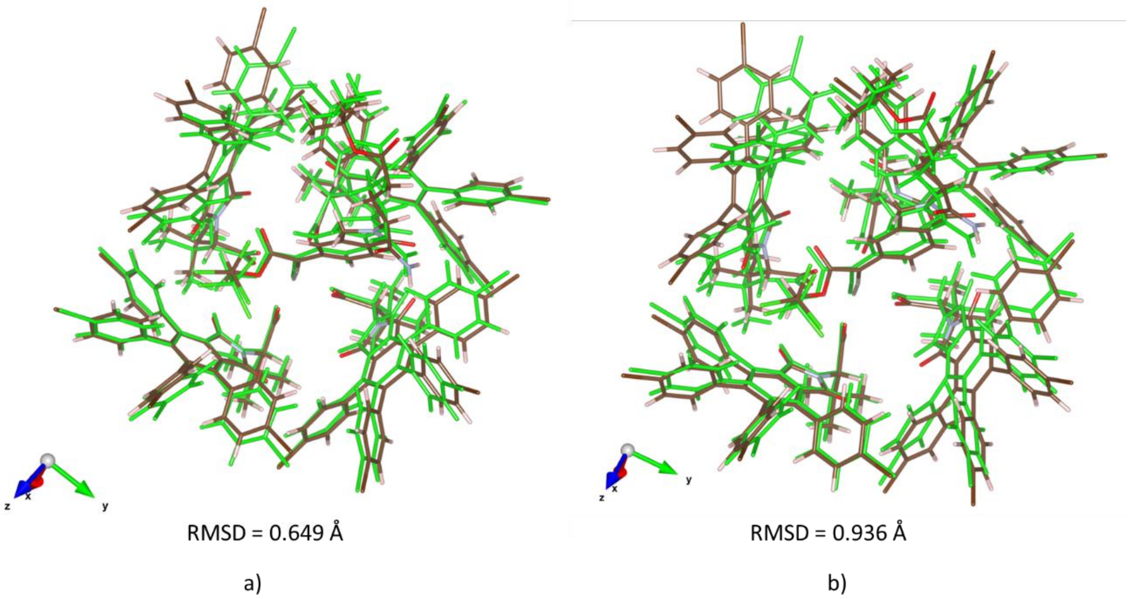

Figure S5. Comparison between structures obtained from ONOIM approach and normal QM approach. a) an overlay between structure **A** (colorful) and **A-SI-2** (green). b) an overlay between structure **A-SI-1** (colorful) and **A-SI-2** (green). Layering structures were generated by Vesta program. Root-Mean-Squares Deviation (RMSD) was calculated by Pymol using align function.

3. Analysis of the metal-carbene complexes with Rh_2(*S-tetra-p*-BrPhPTTL)$_4$ and Rh_2(*S-tetra-p*-BrPhPTTL)$_4$

Based on experimental results and previous computation models, the open face of the Rh_2(*S-tetra-p*-BrPhPTTL)$_4$ is *Re* – face and the open face of the Rh_2(*R-tetra-p*-BrPhPTTL)$_4$ is *Si* – face. Previously, we showed that the attack of the substrate in C-H insertion favored the approach from the trichloroethyl (TCE) side over the carbonyl side (**Figure S6**).[17] Because of the above reasoning and cost of calculation, we only consider the carbene diastereomer resulted from Rh_2(*S-tetra-p*-BrPhPTTL)$_4$ with the TCE group located on the *Re* – face and the one resulted from Rh_2(*R-tetra-p*-BrPhPTTL)$_4$ with the TCE group located on the *Si* – face. The calculated relative free energy of the metal-carbene complex showed that a carbene in a configuration **II** is more stable than configuration **I**, which is in good agreement with a model study with $Rh_2(OAc)_4$. (**Figure S7**) However, the energy difference between 2 configurations is now much more significant at 9.6 and 7.1 kcal/mol. This difference can be attributed to the effect of the bowl-shaped structure of the catalyst as the structure of the catalyst is significantly distorted in configuration **I** compared to configuration **II**. Additionally, the carbene complex resulted from Rh_2(*S-tetra-p*-BrPhPTTL)$_4$ is more energetically favored than the one from Rh_2(*R-tetra-p*-BrPhPTTL)$_4$. This could explain why the yield of the reaction with the *R* enantiomer of the catalyst is lower than the *S* enantiomer.

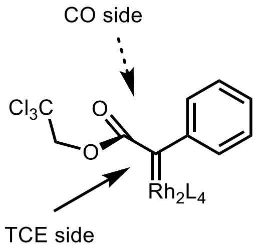

Figure S6. Illustrate of the side of approach during C-H insertion reaction

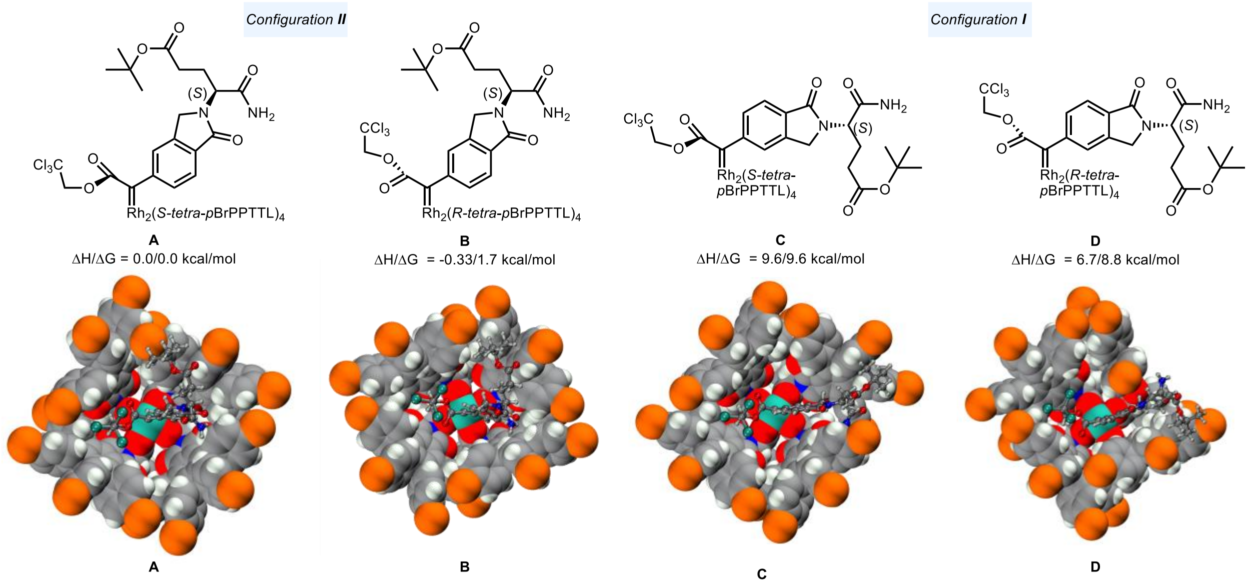

Figure S7. Structure of metal-carbene complex from Rh_2(*S-tetra*-p-*Br*PhPTTL)$_4$ and Rh_2(*R-tetra*-p-*Br*PhPTTL)$_4$ with I and II configuration. The reported relative enthalpies and free energy are in kcal/mol unit and relative to structure **A**.

4. Tables of energies

Table 1. zero-point correction (ZPE), thermal correction to enthalpy (TCH), thermal correction to Gibbs free energy (TCG), energies (E), enthalpies (H), and Gibbs free energies (G) (in Hartree) of the structures calculated at the B3LYP-D3(BJ) +CPCM(CH2Cl2) level of theory

Structure	ZPE	TCH	TCG	E	H	G	Imaginary Frequency
**SI-1**	0.633920	0.691141	0.529830	-3888.905165	-3888.214024	-3888.375335	-
**SI-2**	0.633718	0.690998	0.529273	-3888.908780	-3888.217782	-3888.379507	-

Table 2. zero-point correction (ZPE), thermal correction to enthalpy (TCH), thermal correction to Gibbs free energy (TCG), energies (E), enthalpies (H), and Gibbs free energies (G) (in Hartree) of the structures calculated at the ONIOM[B3LYP:UFF]+CPCM(CH_2Cl_2) level of theory followed **Figure 1**.

Structure	ZPE	TCH	TCG	E	H	G	Imaginary Frequency
**A**	2.710880	2.913795	2.430058	-6564.904862	-6561.991066	-6562.474804	-
**B**	2.711298	2.913860	2.433357	-6564.905450	-6561.991590	-6562.472093	-
**C**	2.711768	2.914275	2.430523	-6564.890041	-6561.975766	-6562.459518	-
**D**	2.712272	2.914810	2.434298	-6564.895144	-6561.90334	-6562.460846	-

5. Cartesian coordinates for calculated structure

Structure SI-1

Rh	6.44401000	12.42180000	-7.03264900
Rh	8.55091000	12.50559000	-5.77183300
O	7.63247900	13.89605300	-4.54137300
O	9.11967000	14.04434900	-7.02630400
O	9.29875900	11.13646300	-7.13402300
O	7.79839300	10.94897900	-4.62877300
O	5.68279800	13.81226100	-5.69917800
O	7.17658100	13.96625400	-8.19772000
O	7.34614400	11.03746800	-8.28494200
O	5.84787700	10.87588100	-5.78485200
C	6.42595800	14.25013400	-4.77268400
C	8.32384400	14.44133200	-7.94429700
C	8.54594600	10.69795700	-8.07270300
C	6.63587900	10.47632200	-4.87921100
C	10.23313900	12.55340600	-4.66258800
C	9.17733300	9.68858300	-8.99972800
H	10.01179500	10.15798700	-9.52865500
H	8.44741300	9.31862300	-9.71897800
H	9.58264500	8.85894200	-8.41446900
C	5.86286600	15.29619700	-3.84156300
H	6.42323800	16.22719700	-3.96927000
H	5.99056600	14.97143500	-2.80579400
H	4.80846800	15.47171200	-4.05277400
C	8.80599600	15.60401700	-8.77670100
H	8.69226000	16.52415500	-8.19433500
H	8.21933200	15.68724700	-9.69151100
H	9.86568700	15.48453200	-9.00970800
C	6.17959400	9.35027100	-3.98411000
H	5.95662500	9.75374700	-2.99144400
H	6.98232200	8.61797800	-3.87221100
H	5.28600700	8.87659800	-4.38979200
C	11.06195900	11.46785500	-4.26904700
C	12.19635200	11.68598100	-3.42358300
C	10.74540400	10.14474800	-4.69489000
C	12.99697200	10.63503300	-3.01110400
H	12.43093100	12.69431500	-3.10249300
C	11.54951400	9.10759400	-4.26825900
H	9.89018600	9.98288000	-5.33378000
C	12.65075900	9.35283500	-3.44047400
H	13.86001800	10.79011800	-2.37353300
C	11.45813000	7.63176500	-4.55174400
C	13.33453300	8.06299500	-3.16353800
N	12.56764500	7.09706800	-3.76096300
H	11.59226700	7.40417600	-5.61650700
H	10.50056200	7.20773700	-4.23284300
O	14.38903400	7.87372800	-2.55381600
C	12.99296200	5.69995500	-3.79626300
C	13.95049000	5.52678400	-5.00053800
C	11.80391800	4.74736300	-3.86163900
H	13.56030800	5.54115700	-2.87407200
N	15.19571200	6.00694300	-4.77337000
O	13.58463100	5.02226200	-6.05889200
C	12.24007700	3.28605900	-3.70257900
H	11.29456900	4.86025100	-4.82019200
H	11.09897500	5.01611900	-3.06815000
H	15.83896300	6.06119100	-5.55048200
H	15.38124700	6.55455600	-3.94003100
C	11.08540300	2.30280200	-3.71737900
H	12.78496900	3.12882800	-2.76852500
H	12.90720300	3.02020900	-4.53033400
O	10.98793300	1.36338500	-2.94987400
O	10.21891900	2.60292000	-4.69692100

C	9.00533800	1.79782200	-4.94366300
C	9.40106100	0.37308700	-5.33245300
C	8.35848200	2.52503100	-6.12106500
C	8.09932800	1.83962800	-3.71269900
H	8.50778100	-0.17975100	-5.63816400
H	9.86816700	-0.14712300	-4.49646900
H	10.09738200	0.39073500	-6.17609300
H	7.43313900	2.01857900	-6.40866400
H	8.12023700	3.55784400	-5.85136800
H	9.03157900	2.53649000	-6.98281500
H	7.89386100	2.87640100	-3.42962800
H	8.55752100	1.32467400	-2.86858000
H	7.14716700	1.35474000	-3.94778300
C	10.55072900	13.90424700	-4.16466400
O	11.17484400	14.73161700	-4.79452200
O	9.98889300	14.10524800	-2.94569200
C	9.95929500	15.43875300	-2.45135100
C	10.81334100	15.54413500	-1.18435400
H	10.34441700	16.13354100	-3.19824400
H	8.92480300	15.67559500	-2.19969600
Cl	12.53544900	15.18200300	-1.55493600
Cl	10.66781700	17.22892200	-0.57656300
Cl	10.22543800	14.39677500	0.06618700

<u>Structure SI-2</u>

Rh	6.31900300	13.05991200	-6.97477400
Rh	8.41675200	12.71143400	-5.74531000
O	7.71143500	14.08338100	-4.36323200
O	9.20008000	14.27415700	-6.84685000
O	8.96149900	11.39927400	-7.25064700
O	7.44879600	11.16966600	-4.75575700
O	5.76926400	14.40119400	-5.49192400
O	7.26466100	14.59802600	-7.98684900
O	7.01310500	11.69756700	-8.37386900
O	5.50751800	11.49484800	-5.88414900
C	6.56817800	14.62911800	-4.53636000
C	8.46917000	14.87478800	-7.70607200
C	8.15244400	11.16957300	-8.21589200
C	6.23045000	10.89554900	-5.03497100
C	10.09264900	12.41572400	-4.66700800
C	8.62843800	10.17687500	-9.24731600
H	9.54123200	10.55361800	-9.71733000
H	7.86320300	10.01348400	-10.00540300
H	8.87606100	9.23260700	-8.75504300
C	6.15987700	15.63468300	-3.48779600
H	6.89094900	16.44774100	-3.46446600
H	6.16600000	15.15647800	-2.50455400
H	5.16891000	16.03389500	-3.70146700
C	9.11251800	16.03593900	-8.42348000
H	9.13660100	16.89698000	-7.74767200
H	8.54093400	16.29850900	-9.31361400
H	10.14236500	15.78947500	-8.68850100
C	5.61791900	9.76025000	-4.25253300
H	5.47591100	10.07921800	-3.21528900
H	6.29875300	8.90608300	-4.24696200
H	4.65590700	9.47617100	-4.67818100
C	10.80052500	11.20777200	-4.42361200
C	10.31804500	9.97394800	-4.95863400
C	11.97950700	11.21446500	-3.61676100
C	10.97612300	8.78125800	-4.69907200
H	9.42296200	9.99041800	-5.56199700
C	12.62778100	10.02275700	-3.37897800
H	12.35370200	12.14348100	-3.20203700
C	12.12806800	8.82830300	-3.91572600
H	10.61463100	7.83675900	-5.09007400
C	13.86811400	9.73050400	-2.57765700

C	13.00207200	7.70987500	-3.47492700
N	14.02625900	8.28824200	-2.77305000
H	13.74073500	9.96946000	-1.51486100
H	14.73897600	10.28063500	-2.94728700
O	12.84581500	6.50097300	-3.66092700
C	15.03561400	7.48192700	-2.09117000
C	14.47279900	7.09058900	-0.70278800
C	16.37036700	8.21270100	-1.99029700
H	15.14186600	6.57337000	-2.69173100
N	13.55121600	6.10107500	-0.75357300
O	14.82683900	7.65526900	0.32949400
C	17.47854000	7.29765400	-1.45562900
H	16.27042600	9.07146800	-1.32439100
H	16.63945100	8.58367200	-2.98457600
H	13.03344200	5.88045800	0.08543800
H	13.19439700	5.78766500	-1.64966700
C	18.83544600	7.97090900	-1.37926400
H	17.59557800	6.40489300	-2.07478200
H	17.21656100	6.97223800	-0.44253400
O	19.87452500	7.43863000	-1.72483100
O	18.72512900	9.20402900	-0.86381100
C	19.89576400	10.08616300	-0.67753200
C	20.86909500	9.45027200	0.31515300
C	19.26835900	11.35042300	-0.09330800
C	20.54375600	10.37509100	-2.03182100
H	21.66711600	10.16244900	0.54531300
H	21.31372400	8.54407500	-0.09571800
H	20.35191900	9.20344700	1.24707400
H	20.04382600	12.09788200	0.09466700
H	18.53817900	11.77393600	-0.78870400
H	18.76281600	11.12821900	0.85057000
H	19.79934700	10.76770900	-2.73097100
H	20.98972700	9.47540900	-2.45536400
H	21.32503200	11.13028100	-1.90472500
C	10.56455700	13.64691500	-4.00404100
O	11.25346400	14.49198300	-4.53505500
O	10.06514400	13.71984300	-2.74383100
C	10.21354700	14.95160500	-2.04682200
C	11.14278000	14.76071400	-0.84329300
H	10.63137300	15.71679600	-2.70180800
H	9.22798100	15.24787100	-1.68561200
Cl	12.79862000	14.31276900	-1.38736800
Cl	11.20361500	16.32179100	0.04325500
Cl	10.51250500	13.47299700	0.23859800

Structure A

Rh	0	-0.39893618	0.06648936	0.00000000	H
Rh	0	1.85221582	-0.06469364	-1.00789600	H
Br	0	2.43003582	-7.93353664	4.44283300	L
Br	0	-1.02574818	-7.40566664	10.85677700	L
Br	0	-1.77997218	5.86830436	9.38045400	L
Br	0	-3.44282618	-0.51664964	13.50629800	L
O	0	2.60074482	0.53922036	0.82655100	H
O	0	0.55046782	0.50968236	1.79236400	H
O	0	0.86986382	2.36349036	5.28219000	H
O	0	2.04447482	-1.70759164	3.55351600	H
N	0	1.73761382	0.49086136	4.23502800	H
C	0	1.81312382	0.69096436	1.80159900	H
C	0	2.36313782	1.22729636	3.13591800	H
H	0	1.92160282	2.22904036	3.21399700	H
C	0	3.91018382	1.41989336	3.29107300	H
C	0	4.36827082	2.51933536	2.30719100	H
H	0	3.80414982	3.44647236	2.45940800	H
H	0	4.24437982	2.21020636	1.26839500	H
H	0	5.42725682	2.74129036	2.47536200	H
C	0	4.18329882	1.92766336	4.72389700	H

H	0	3.96110082	1.16627536	5.47760400	H
H	0	3.58829382	2.81641636	4.95238800	H
H	0	5.24078382	2.19169436	4.82246700	H
C	0	4.71644482	0.12849836	3.05723100	H
H	0	4.57530482	-0.25329964	2.04447100	H
H	0	4.43364382	-0.65775564	3.76227800	H
H	0	5.78250982	0.33668036	3.20031000	H
C	0	1.02017982	1.15824336	5.23618100	H
C	0	1.63778682	-0.89799264	4.36840700	H
C	0	0.92638282	-1.13635964	5.67174700	H
C	0	0.52476782	0.10436436	6.16989600	H
C	0	-0.17012118	0.23448536	7.36569100	H
C	0	-0.43657818	-0.94713064	8.10058900	H
C	0	-0.01877118	-2.21271964	7.60909100	H
C	0	0.65864782	-2.31627864	6.36214200	H
C	0	1.07637682	-3.62612964	5.80238800	L H 35
C	0	2.43907282	-3.92080764	5.63733000	L
H	0	3.19156882	-3.17544564	5.86376400	L
C	0	2.83785682	-5.19252564	5.21562300	L
H	0	3.89111882	-5.41548164	5.10492900	L
C	0	1.88026682	-6.17910964	4.96018700	L
C	0	0.52042982	-5.88472364	5.09960400	L
H	0	-0.22378518	-6.64396764	4.89789100	L
C	0	0.11820682	-4.61283064	5.51636800	L
H	0	-0.93712418	-4.40378364	5.63795200	L
C	0	-0.26225918	-3.44433564	8.41254700	L H 34
C	0	-1.57222318	-3.85936264	8.70306700	L
H	0	-2.41783618	-3.27462564	8.36989200	L
C	0	-1.79702818	-5.03221764	9.42820900	L
H	0	-2.81096818	-5.34498164	9.64185900	L
C	0	-0.71594818	-5.79853664	9.87369000	L
C	0	0.59183782	-5.38572664	9.60019200	L
H	0	1.43175582	-5.97375364	9.94686600	L
C	0	0.81931582	-4.21209564	8.87602000	L
H	0	1.83703882	-3.90244764	8.67312100	L
C	0	-1.15586818	-0.84508264	9.39733100	L H 33
C	0	-2.49657118	-0.42967564	9.42951100	L
H	0	-3.01630218	-0.18718064	8.51099400	L
C	0	-3.17307218	-0.33225664	10.64842500	L
H	0	-4.20696518	-0.01249864	10.66430800	L
C	0	-2.51616318	-0.64871064	11.84206100	L
C	0	-1.17990318	-1.06145164	11.81589700	L
H	0	-0.66770118	-1.30585564	12.73749300	L
C	0	-0.50008918	-1.15801864	10.59853900	L
H	0	0.53598782	-1.47340564	10.59010800	L
C	0	-0.56096018	1.58037836	7.85136800	L H 32
C	0	-1.51235118	2.33771436	7.14943000	L
H	0	-1.96528418	1.94690236	6.24741800	L
C	0	-1.87634118	3.60783936	7.60572200	L
H	0	-2.60645518	4.18781336	7.05575800	L
C	0	-1.29258418	4.12936736	8.76455400	L
C	0	-0.34519718	3.37886536	9.46836400	L
H	0	0.11037182	3.78030836	10.36429100	L
C	0	0.02029382	2.10845236	9.01453900	L
H	0	0.75959982	1.53893636	9.56414700	L
Br	0	-1.27224518	-5.49218664	-7.83898200	L
Br	0	-7.98391118	-8.78309964	-5.76247100	L
Br	0	-4.63546618	-6.85566064	6.97143800	L
Br	0	-9.93043318	-9.01478564	1.61188800	L
O	0	1.94968182	-2.04117064	-0.37268800	H
O	0	-0.17515818	-1.94747564	0.40622200	H
O	0	-1.12940718	-4.89875764	2.46789900	H
O	0	-0.23072118	-4.23944764	-1.95834300	H
N	0	-0.27917718	-4.64036764	0.32717300	H
C	0	0.93346482	-2.53897464	0.18288600	H

C	0	0.96304382	-3.97911164	0.72941900	H
H	0	0.84427082	-3.84853464	1.81193500	H
C	0	2.24996982	-4.84647364	0.53626100	H
C	0	3.38440382	-4.20308764	1.36303100	H
H	0	3.09804182	-4.09001464	2.41376200	H
H	0	3.65010982	-3.21668364	0.97747500	H
H	0	4.27530182	-4.83821364	1.32155300	H
C	0	1.95178982	-6.24448864	1.11880500	H
H	0	1.24862382	-6.80295764	0.49388600	H
H	0	1.52386482	-6.17359964	2.12276600	H
H	0	2.87701682	-6.82545364	1.18225300	H
C	0	2.69917682	-5.00399864	-0.92879700	H
H	0	2.94569182	-4.04001164	-1.37714900	H
H	0	1.92928682	-5.47925164	-1.54105000	H
H	0	3.59282282	-5.63724064	-0.96214900	H
C	0	-1.24779818	-5.02857664	1.26226800	H
C	0	-0.77703818	-4.71584464	-0.97843000	H
C	0	-2.09299118	-5.42409264	-0.88078900	H
C	0	-2.39206218	-5.58081064	0.47344100	H
C	0	-3.59188618	-6.13827964	0.90307200	H
C	0	-4.51319218	-6.54083964	-0.09395500	H
C	0	-4.19052018	-6.42451564	-1.46964700	H
C	0	-2.95769418	-5.85965064	-1.88166600	H
C	0	-2.55767118	-5.77129564	-3.30969400	L H 108
C	0	-1.38971318	-6.41156964	-3.75663000	L
H	0	-0.77355118	-6.97241164	-3.06450900	L
C	0	-1.01068718	-6.33023664	-5.09971200	L
H	0	-0.10372718	-6.81830664	-5.43222900	L
C	0	-1.80039918	-5.61981164	-6.00892500	L
C	0	-2.97185018	-4.99332464	-5.57394000	L
H	0	-3.58774318	-4.44354564	-6.27389500	L
C	0	-3.34466718	-5.06085364	-4.22959400	L
H	0	-4.24296718	-4.55392764	-3.90577800	L
C	0	-5.10852918	-6.99285664	-2.48718500	L H 107
C	0	-6.20322918	-6.24804464	-2.94824400	L
H	0	-6.38237118	-5.25206564	-2.56562100	L
C	0	-7.05424318	-6.77890364	-3.92127700	L
H	0	-7.89142118	-6.19437164	-4.27989900	L
C	0	-6.81976318	-8.05906564	-4.43360500	L
C	0	-5.72956618	-8.80639764	-3.97461800	L
H	0	-5.54330318	-9.79647264	-4.37018200	L
C	0	-4.87410918	-8.27471864	-3.00511400	L
H	0	-4.02989618	-8.85809064	-2.65861700	L
C	0	-5.81062618	-7.13614864	0.30914500	L H 106
C	0	-6.98233318	-6.36562464	0.26703800	L
H	0	-6.94591018	-5.33248464	-0.05577700	L
C	0	-8.20366918	-6.92422264	0.65293900	L
H	0	-9.10341218	-6.32348564	0.61913600	L
C	0	-8.26070718	-8.25360164	1.08451400	L
C	0	-7.09338618	-9.02378364	1.13205600	L
H	0	-7.13318618	-10.05199664	1.46762600	L
C	0	-5.87029218	-8.46714664	0.74653600	L
H	0	-4.97136218	-9.06991964	0.78722000	L
C	0	-3.85452118	-6.31822964	2.35213000	L H 105
C	0	-4.92515518	-5.65373864	2.97022500	L
H	0	-5.56378718	-4.99278564	2.39813200	L
C	0	-5.16771618	-5.82908564	4.33552700	L
H	0	-5.99363418	-5.30995564	4.80439200	L
C	0	-4.33186218	-6.65284564	5.09692300	L
C	0	-3.26374018	-7.31719264	4.48688500	L
H	0	-2.61461718	-7.95579964	5.07196600	L
C	0	-3.02915718	-7.15717564	3.11852100	L
H	0	-2.20085618	-7.68059664	2.65672600	L
Br	0	-0.92670518	7.74422236	-5.92705800	L
Br	0	-8.68832918	7.83856036	-6.85345500	L

Br	0	-9.33174018	-4.35306364	-2.10610800	L
Br	0	-12.73451018	1.56653936	-4.77804200	L
O	0	0.92354282	-0.66175164	-2.78561500	H
O	0	-1.12919918	-0.34287064	-1.88438200	H
O	0	-3.80240818	-1.96574864	-3.78599400	H
O	0	-1.32249718	1.82856336	-4.33641900	H
N	0	-2.27239318	-0.28862264	-4.27067600	H
C	0	-0.33636118	-0.65146764	-2.83595200	H
C	0	-1.07635418	-1.11735564	-4.10538400	H
H	0	-1.48904318	-2.09271464	-3.81626400	H
C	0	-0.25599418	-1.36698864	-5.41654800	H
C	0	0.63300282	-2.60951264	-5.18692300	H
H	0	0.03826582	-3.47360964	-4.87267800	H
H	0	1.38900682	-2.42325264	-4.42184100	H
H	0	1.14451782	-2.87357564	-6.11822300	H
C	0	-1.25663318	-1.68540864	-6.54776200	H
H	0	-1.84172618	-0.80647164	-6.83346800	H
H	0	-1.95633118	-2.47411364	-6.25789200	H
H	0	-0.71266618	-2.02685464	-7.43389700	H
C	0	0.62362782	-0.17917964	-5.85276000	H
H	0	1.35893282	0.07520336	-5.08698800	H
H	0	0.02813182	0.71126736	-6.06439500	H
H	0	1.16277582	-0.45098864	-6.76708000	H
C	0	-3.55922218	-0.80429264	-4.05174900	H
C	0	-2.30236718	1.10561536	-4.38815800	H
C	0	-3.75186518	1.47790636	-4.52738100	H
C	0	-4.50263918	0.33940736	-4.22924700	H
C	0	-5.89092118	0.36159236	-4.18067800	H
C	0	-6.53800218	1.56783236	-4.54878400	H
C	0	-5.77985618	2.71590936	-4.90354300	H
C	0	-4.35913618	2.68968936	-4.85207400	H
C	0	-3.53406718	3.89882036	-5.11117300	L H 181
C	0	-2.55396718	3.89093736	-6.11752300	L
H	0	-2.39085218	3.00348836	-6.71662300	L
C	0	-1.77921118	5.02998836	-6.35614100	L
H	0	-1.01896218	5.01036536	-7.12621800	L
C	0	-1.98914618	6.19097636	-5.60552900	L
C	0	-2.97698118	6.21325336	-4.61720700	L
H	0	-3.14310818	7.10997636	-4.03456500	L
C	0	-3.74176818	5.07099336	-4.36541100	L
H	0	-4.49035718	5.09696536	-3.58444300	L
C	0	-6.47275718	3.95045236	-5.36545900	L H 180
C	0	-7.34356618	4.64265236	-4.50869000	L
H	0	-7.51665418	4.28665236	-3.50325400	L
C	0	-7.99898518	5.79480536	-4.94986000	L
H	0	-8.67056418	6.31886336	-4.28215300	L
C	0	-7.78631118	6.26810236	-6.24840700	L
C	0	-6.91567818	5.58759636	-7.10543700	L
H	0	-6.74845618	5.95036236	-8.11133600	L
C	0	-6.26222718	4.43222036	-6.66778900	L
H	0	-5.59717718	3.90904836	-7.34382000	L
C	0	-8.02405118	1.60359736	-4.59897600	L H 179
C	0	-8.77725918	1.48437736	-3.41986900	L
H	0	-8.28233018	1.39003536	-2.46163200	L
C	0	-10.17336718	1.47383936	-3.47728600	L
H	0	-10.74834818	1.37028536	-2.56940000	L
C	0	-10.82684518	1.59014236	-4.70716900	L
C	0	-10.08227118	1.71710536	-5.88400300	L
H	0	-10.58429518	1.80510036	-6.83877900	L
C	0	-8.68558318	1.72047236	-5.83203200	L
H	0	-8.11876418	1.80357336	-6.75115100	L
C	0	-6.64233918	-0.82914964	-3.71302500	L H 178
C	0	-6.55409318	-1.22894364	-2.36961700	L
H	0	-5.89682018	-0.70609364	-1.68600900	L
C	0	-7.34126918	-2.28324464	-1.89595800	L

H	0	-7.27968318	-2.57412164	-0.85507100	L
C	0	-8.21916018	-2.94560264	-2.76090200	L
C	0	-8.29246918	-2.56726964	-4.10537200	L
H	0	-8.96827618	-3.07944464	-4.77792900	L
C	0	-7.50701118	-1.51331764	-4.58161800	L
H	0	-7.58730318	-1.21598064	-5.61987200	L
Br	0	3.86994982	5.30766836	6.79502600	L
Br	0	-1.96959318	9.80336436	8.71669500	L
Br	0	-6.47935818	8.94561936	-3.71086700	L
Br	0	-7.37735718	11.42918336	3.63794100	L
O	0	1.63612882	1.92391836	-1.55851800	H
O	0	-0.34057118	2.09780536	-0.47111300	H
O	0	-2.06145318	5.43180036	-1.78988300	H
O	0	1.19740982	4.26379736	1.20215000	H
N	0	-0.19271218	4.79156936	-0.58092800	H
C	0	0.61583382	2.56010736	-1.17961000	H
C	0	0.40384082	4.00666936	-1.66264600	H
H	0	-0.41137818	3.91702936	-2.39306600	H
C	0	1.57421782	4.73240836	-2.41077000	H
C	0	1.82792182	4.00611036	-3.75055900	H
H	0	0.91138882	3.95061736	-4.34919500	H
H	0	2.19984582	2.99224836	-3.59787000	H
H	0	2.57171282	4.56162736	-4.33117700	H
C	0	1.11325082	6.17023936	-2.73849400	H
H	0	0.98872782	6.77663536	-1.83637500	H
H	0	0.16533282	6.17359436	-3.28541000	H
H	0	1.86643182	6.65964436	-3.36373900	H
C	0	2.87310982	4.79732536	-1.58599000	H
H	0	3.24880382	3.79893236	-1.35311800	H
H	0	2.72794982	5.33622036	-0.64584900	H
H	0	3.64162982	5.32723736	-2.15926700	H
C	0	-1.40796318	5.46008036	-0.76309700	H
C	0	0.24370282	4.87132936	0.74956900	H
C	0	-0.70723218	5.81246336	1.44128100	H
C	0	-1.70303818	6.15152136	0.52260700	H
C	0	-2.73187118	7.03082636	0.82933400	H
C	0	-2.76845418	7.56238036	2.13789000	H
C	0	-1.76477218	7.22173336	3.08209000	H
C	0	-0.70188018	6.34224036	2.73243700	H
C	0	0.40029582	6.02246236	3.67834400	L H 254
C	0	1.72968082	6.33101936	3.34419900	L
H	0	1.96582782	6.76468436	2.38021300	L
C	0	2.75946282	6.10331836	4.26209000	L
H	0	3.78024782	6.34334636	3.99417800	L
C	0	2.46846682	5.58776136	5.52874400	L
C	0	1.14840682	5.28261236	5.87059800	L
H	0	0.92005582	4.88318236	6.84997000	L
C	0	0.12029682	5.48118236	4.94476700	L
H	0	-0.89550718	5.22835436	5.22025300	L
C	0	-1.80555418	7.82854536	4.43918300	L H 253
C	0	-2.83603318	7.49525136	5.33293400	L
H	0	-3.60002818	6.78318336	5.04607400	L
C	0	-2.88316518	8.08088736	6.60086800	L
H	0	-3.68260318	7.82094336	7.28258600	L
C	0	-1.90006218	8.99809636	6.98669200	L
C	0	-0.86775518	9.32860936	6.10284500	L
H	0	-0.10530318	10.03796136	6.39758600	L
C	0	-0.82051318	8.74847436	4.83229700	L
H	0	-0.02188818	9.01862336	4.15242300	L
C	0	-3.87007518	8.49085936	2.50399100	L H 252
C	0	-5.19232218	8.02303836	2.56739800	L
H	0	-5.41560918	6.98345736	2.36044500	L
C	0	-6.23116618	8.89530736	2.90330100	L
H	0	-7.24757918	8.52614636	2.95089100	L
C	0	-5.95683618	10.23921436	3.17757900	L

C	0	-4.64154618	10.71123536	3.11263200	L
H	0	-4.42563718	11.75103636	3.32151100	L
C	0	-3.60081218	9.84174136	2.77416300	L
H	0	-2.58733018	10.21945536	2.71862100	L
C	0	-3.65178718	7.49141936	-0.23539500	L H 251
C	0	-4.69377618	6.66793436	-0.68524600	L
H	0	-4.84882218	5.69402336	-0.23998200	L
C	0	-5.53600518	7.10337436	-1.71391400	L
H	0	-6.33273918	6.46388736	-2.06739700	L
C	0	-5.34281718	8.36102936	-2.29398700	L
C	0	-4.31143018	9.18857636	-1.83999500	L
H	0	-4.15883718	10.16281236	-2.28604100	L
C	0	-3.46862718	8.75665036	-0.81276300	L
H	0	-2.66761018	9.40187636	-0.47315400	L
C	0	-2.32647618	0.00878736	0.71560900	H
C	0	-3.45845818	0.80075536	0.35603100	H
C	0	-2.61275618	-1.18061664	1.55970300	H
C	0	-3.31821018	1.91255936	-0.53182200	H
C	0	-4.74715718	0.51452436	0.90753100	H
O	0	-2.98211218	-2.23417164	1.08449500	H
O	0	-2.38429018	-0.94981164	2.87496400	H
C	0	-4.39229118	2.73770836	-0.83160000	H
H	0	-2.34408918	2.11348136	-0.95252500	H
C	0	-5.80732418	1.33555636	0.59331000	H
H	0	-4.88888718	-0.33381664	1.56589400	H
C	0	-2.53140918	-2.04043164	3.79020100	H
C	0	-5.62509518	2.43739036	-0.25275700	H
H	0	-4.27421318	3.59148036	-1.48919900	H
C	0	-7.24841418	1.26315136	1.02538600	H
C	0	-3.79905918	-1.87919064	4.64624800	H
H	0	-2.56296218	-2.99591864	3.26462500	H
H	0	-1.66979418	-2.00699564	4.45775400	H
C	0	-6.92118618	3.15804236	-0.36827100	H
N	0	-7.83377618	2.40887436	0.32671000	H
H	0	-7.36226118	1.35991036	2.11205800	H
H	0	-7.71839518	0.32253836	0.71895700	H
Cl	0	-5.29458118	-2.06661164	3.65791900	H
Cl	0	-3.76189718	-3.17202564	5.89211900	H
Cl	0	-3.83071718	-0.27309164	5.45209000	H
O	0	-7.15728518	4.22359636	-0.93823800	H
C	0	-9.24096918	2.80625736	0.40301900	H
C	0	-9.44861018	3.76655236	1.60372300	H
C	0	-10.16059818	1.58686436	0.44546400	H
H	0	-9.42382518	3.37904936	-0.51310800	H
N	0	-8.90140818	4.99045236	1.41645500	H
O	0	-10.05192118	3.42678236	2.61876500	H
C	0	-11.63579318	1.94730336	0.21070800	H
H	0	-10.05795518	1.07466036	1.40507900	H
H	0	-9.82615418	0.89100636	-0.32880100	H
H	0	-8.90419618	5.65013836	2.18146500	H
H	0	-8.34081418	5.18294836	0.59328900	H
C	0	-12.48883918	0.79316736	-0.29582000	H
H	0	-11.74249418	2.74487136	-0.53162000	H
H	0	-12.08303818	2.32553836	1.13669300	H
O	0	-13.48239918	0.95299836	-0.98118100	H
O	0	-11.99770018	-0.38938764	0.10423500	H
C	0	-12.51894618	-1.68220464	-0.39503500	H
C	0	-13.98750918	-1.85785164	-0.00003000	H
C	0	-11.63475718	-2.69577864	0.33426600	H
C	0	-12.30177118	-1.75062764	-1.91006900	H
H	0	-14.30999918	-2.87276764	-0.25317700	H
H	0	-14.62627118	-1.14428264	-0.51996800	H
H	0	-14.10967718	-1.72284164	1.07914900	H
H	0	-11.89093818	-3.71152764	0.01996100	H
H	0	-10.57906518	-2.51728564	0.11065600	H

H	0	-11.77514318	-2.62302764	1.41665000	H	
H	0	-11.24887418	-1.57429064	-2.15259000	H	
H	0	-12.91247218	-1.01125664	-2.43040700	H	
H	0	-12.57298118	-2.74579864	-2.27527400	H	

Structure B

Rh	0	-2.27393623	0.41223404	0.00000000	H	
Rh	0	0.09611277	0.28628704	0.68644900	H	
Br	0	-0.99085923	-7.62470996	-4.90914100	L	
Br	0	-5.68014623	-6.44779296	-10.65385400	L	
Br	0	-3.64206523	6.57371804	-9.66849400	L	
Br	0	-7.44567423	0.64511904	-12.84089600	L	
O	0	0.58432577	0.83633504	-1.25363500	H	
O	0	-1.58369823	0.89970504	-1.90676600	H	
O	0	-1.51618923	2.79897704	-5.48533600	H	
O	0	-0.64886623	-1.39622396	-3.86857400	H	
N	0	-0.78931423	0.83033204	-4.51160600	H	
C	0	-0.32827523	1.01023504	-2.10758600	H	
C	0	0.05952977	1.49167204	-3.51896800	H	
H	0	-0.28652823	2.53323804	-3.54302800	H	
C	0	1.57991477	1.53276104	-3.90389900	H	
C	0	2.28235377	2.59374604	-3.02746400	H	
H	0	1.79328577	3.57032104	-3.12184900	H	
H	0	2.28586577	2.31233004	-1.97385600	H	
H	0	3.32033977	2.70950504	-3.35632600	H	
C	0	1.69455277	1.99418804	-5.37414300	H	
H	0	1.29134677	1.25189004	-6.06966600	H	
H	0	1.17004977	2.93913804	-5.54242300	H	
H	0	2.74948877	2.14146104	-5.62554900	H	
C	0	2.27978877	0.16861604	-3.75608000	H	
H	0	2.25414777	-0.18501796	-2.72380600	H	
H	0	1.81677577	-0.59217096	-4.39057900	H	
H	0	3.32791577	0.26327304	-4.06045700	H	
C	0	-1.51948223	1.58386504	-5.43759300	H	
C	0	-1.06791823	-0.53794196	-4.62495600	H	
C	0	-1.97028323	-0.67891596	-5.82448600	H	
C	0	-2.25379923	0.60725804	-6.29049200	H	
C	0	-3.05637923	0.84002304	-7.39943300	H	
C	0	-3.62046123	-0.28472896	-8.04617600	H	
C	0	-3.32944923	-1.59681796	-7.58845200	H	
C	0	-2.48239323	-1.80892596	-6.46389500	H	
C	0	-2.12079023	-3.17536196	-6.00012500	L	H 35
C	0	-0.77494123	-3.57734196	-5.96514100	L	
H	0	0.01232077	-2.87970196	-6.22312500	L	
C	0	-0.43939923	-4.89143196	-5.62467500	L	
H	0	0.60039177	-5.19077596	-5.59967300	L	
C	0	-1.44486223	-5.82096496	-5.34091600	L	
C	0	-2.78570123	-5.42969496	-5.37579500	L	
H	0	-3.56595723	-6.14552196	-5.15386400	L	
C	0	-3.12256623	-4.10859496	-5.68176800	L	
H	0	-4.16510623	-3.81825696	-5.69268100	L	
C	0	-3.89152023	-2.76520896	-8.31763400	L	H 34
C	0	-5.27506523	-3.00261996	-8.31321400	L	
H	0	-5.93995623	-2.34339696	-7.76964600	L	
C	0	-5.80283023	-4.09189196	-9.01085500	L	
H	0	-6.86977323	-4.26772196	-9.00520700	L	
C	0	-4.95418123	-4.95649496	-9.70845100	L	
C	0	-3.57443323	-4.72829496	-9.71221000	L	
H	0	-2.91403023	-5.39494596	-10.25145900	L	
C	0	-3.04353923	-3.63498896	-9.02215600	L	
H	0	-1.97440523	-3.46277796	-9.03814900	L	
C	0	-4.53565723	-0.06760896	-9.20043300	L	H 33
C	0	-5.74193023	0.62883204	-9.01932500	L	
H	0	-6.01601323	1.00288704	-8.04073500	L	
C	0	-6.59738323	0.84736004	-10.10184100	L	
H	0	-7.52040123	1.39032204	-9.95549500	L	

C	0	-6.26292323	0.36213704	-11.36936100	L
C	0	-5.06206023	-0.32939696	-11.55768600	L
H	0	-4.79668923	-0.70157696	-12.53870500	L
C	0	-4.19657723	-0.53721396	-10.47993800	L
H	0	-3.26266623	-1.06092696	-10.64143300	L
C	0	-3.20421623	2.21400304	-7.93640600	L H 32
C	0	-3.90820123	3.19015504	-7.21577700	L
H	0	-4.34861723	2.94676804	-6.25819400	L
C	0	-4.03812523	4.48424704	-7.72917600	L
H	0	-4.57888423	5.23345304	-7.16807400	L
C	0	-3.46870623	4.80863204	-8.96492000	L
C	0	-2.77127823	3.83723704	-9.68896900	L
H	0	-2.33032723	4.08380004	-10.64616200	L
C	0	-2.63926323	2.54391104	-9.17798500	L
H	0	-2.09311223	1.80049204	-9.74568400	L
Br	0	-2.84065423	-5.14927496	7.84827000	L
Br	0	-8.92623123	-9.56291196	5.95551800	L
Br	0	-7.15211123	-6.51282896	-6.73233300	L
Br	0	-11.36854723	-9.64868196	-1.23005000	L
O	0	0.08385677	-1.69115296	0.07293700	H
O	0	-2.11342423	-1.60250796	-0.47681200	H
O	0	-3.07137623	-4.74005896	-2.52185700	H
O	0	-1.97315923	-3.93263796	1.84005700	H
N	0	-2.15080023	-4.32910196	-0.43949400	H
C	0	-0.98413923	-2.18953296	-0.37680600	H
C	0	-0.97667323	-3.61871696	-0.94772900	H
H	0	-1.20706923	-3.47151796	-2.01024400	H
C	0	0.34974177	-4.44850096	-0.90094700	H
C	0	1.40092177	-3.73521396	-1.77969400	H
H	0	1.02579577	-3.57853496	-2.79715100	H
H	0	1.68264777	-2.76577296	-1.36574300	H
H	0	2.30170677	-4.35413696	-1.84651200	H
C	0	0.06204777	-5.82724296	-1.53417300	H
H	0	-0.62274423	-6.42371496	-0.92398200	H
H	0	-0.37391023	-5.72286196	-2.53186300	H
H	0	0.99631777	-6.38920796	-1.62936800	H
C	0	0.90411277	-4.65651896	0.52085100	H
H	0	1.14339377	-3.70530396	0.99948000	H
H	0	0.19590277	-5.19215896	1.15829000	H
H	0	1.82086777	-5.25401096	0.46632600	H
C	0	-3.12951023	-4.82217196	-1.31089100	H
C	0	-2.54661823	-4.44863396	0.89683700	H
C	0	-3.80111023	-5.27973196	0.88199700	H
C	0	-4.18377823	-5.43759696	-0.45214700	H
C	0	-5.34680623	-6.10447096	-0.81809300	H
C	0	-6.12008423	-6.68212696	0.21936700	H
C	0	-5.71010623	-6.57005096	1.57514000	H
C	0	-4.54421923	-5.83439796	1.92305000	H
C	0	-4.12328523	-5.64322996	3.33559900	L H 108
C	0	-2.85485523	-6.06870596	3.76350000	L
H	0	-2.16611123	-6.52713296	3.06452200	L
C	0	-2.47266423	-5.91352896	5.09931300	L
H	0	-1.49096023	-6.23923496	5.41798600	L
C	0	-3.35947023	-5.34958396	6.02175600	L
C	0	-4.62808223	-4.93535296	5.60611700	L
H	0	-5.31787223	-4.49843596	6.31677700	L
C	0	-5.00608823	-5.07270096	4.26751400	L
H	0	-5.98571123	-4.73369396	3.95593600	L
C	0	-6.47860223	-7.27053596	2.64051500	L H 107
C	0	-7.80589023	-6.90702196	2.92024000	L
H	0	-8.27919623	-6.10216696	2.37488800	L
C	0	-8.53104623	-7.58901396	3.90030500	L
H	0	-9.55640623	-7.30725696	4.10186100	L
C	0	-7.93304723	-8.62955996	4.61817500	L
C	0	-6.60778523	-8.98869496	4.35327200	L

H	0	-6.14121923	-9.79357296	4.90634100	L
C	0	-5.88304423	-8.31466096	3.36656000	L
H	0	-4.86140223	-8.61012096	3.16162900	L
C	0	-7.37709623	-7.39655096	-0.12689100	L H 106
C	0	-8.47038123	-6.68547996	-0.64643200	L
H	0	-8.40560323	-5.61501696	-0.79464900	L
C	0	-9.65283723	-7.35435096	-0.97329900	L
H	0	-10.49127823	-6.79811996	-1.37212200	L
C	0	-9.75134323	-8.73673096	-0.78435700	L
C	0	-8.66401523	-9.45085196	-0.27006700	L
H	0	-8.73563623	-10.52091796	-0.12434900	L
C	0	-7.47925823	-8.78481896	0.05582000	L
H	0	-6.64143323	-9.34820896	0.44766400	L
C	0	-5.73462123	-6.20093796	-2.24778200	L H 105
C	0	-6.04108123	-5.04012696	-2.97597300	L
H	0	-5.95957423	-4.06650496	-2.51064400	L
C	0	-6.45897323	-5.13249796	-4.30662700	L
H	0	-6.69965823	-4.23263696	-4.85836200	L
C	0	-6.56652123	-6.38401496	-4.92104900	L
C	0	-6.24673823	-7.54287396	-4.20644600	L
H	0	-6.32459323	-8.51290296	-4.68009600	L
C	0	-5.83119123	-7.45321796	-2.87501300	L
H	0	-5.59025223	-8.35817796	-2.33117500	L
Br	0	-3.47229323	8.34164604	4.73936700	L
Br	0	-10.42675723	7.50539504	7.46782300	L
Br	0	-9.04057323	-5.66796296	6.91590600	L
Br	0	-13.41947923	0.56579204	8.39799100	L
O	0	-0.56562323	-0.26047396	2.57872400	H
O	0	-2.73434623	-0.12832196	1.93987200	H
O	0	-4.84819023	-2.02242496	4.70470400	H
O	0	-2.89970023	2.07724704	4.15533300	H
N	0	-3.53258223	-0.12805996	4.50778400	H
C	0	-1.80725723	-0.35012396	2.78559000	H
C	0	-2.31546923	-0.85585396	4.14648500	H
H	0	-2.68056623	-1.86771596	3.92618700	H
C	0	-1.29143423	-1.01004696	5.32303200	H
C	0	-0.28918923	-2.12785796	4.95908700	H
H	0	-0.80928723	-3.06638096	4.73471200	H
H	0	0.32155577	-1.85856496	4.09634900	H
H	0	0.37702277	-2.31231896	5.80827400	H
C	0	-2.06883223	-1.46667796	6.57744500	H
H	0	-2.73617923	-0.68514696	6.95283400	H
H	0	-2.66944323	-2.35827796	6.37444200	H
H	0	-1.36123723	-1.70880796	7.37647300	H
C	0	-0.53926723	0.29399804	5.64770800	H
H	0	0.05131577	0.63709804	4.79596900	H
H	0	-1.22647823	1.09514804	5.93291100	H
H	0	0.14074477	0.12195204	6.48924900	H
C	0	-4.71793223	-0.81486196	4.78406700	H
C	0	-3.73312123	1.25961204	4.50229100	H
C	0	-5.14086123	1.47553104	4.99759400	H
C	0	-5.72129623	0.21654404	5.16652800	H
C	0	-7.00199523	0.04439204	5.67251600	H
C	0	-7.73204523	1.20591204	6.01149500	H
C	0	-7.16335923	2.49272004	5.82365200	H
C	0	-5.84445623	2.64031904	5.30804400	H
C	0	-5.22841023	3.97845104	5.10782400	L H 181
C	0	-4.04960423	4.32731304	5.78758900	L
H	0	-3.55310023	3.60864804	6.42788700	L
C	0	-3.51989423	5.61572204	5.66493800	L
H	0	-2.61002823	5.87450604	6.19087400	L
C	0	-4.17538623	6.57210804	4.88276500	L
C	0	-5.35101823	6.23357204	4.20755800	L
H	0	-5.85982823	6.97046304	3.60060900	L
C	0	-5.86641723	4.93863304	4.30381900	L

H	0	-6.77422023	4.68966804	3.76903800	L
C	0	-7.94290723	3.69927704	6.21374400	L H 180
C	0	-9.13463223	4.02044404	5.54362800	L
H	0	-9.48843323	3.40119904	4.72857100	L
C	0	-9.87438323	5.14319004	5.92386600	L
H	0	-10.79533523	5.37916804	5.40674300	L
C	0	-9.42246123	5.96204704	6.96326900	L
C	0	-8.23418223	5.65030304	7.63158900	L
H	0	-7.88222023	6.28013904	8.43832700	L
C	0	-7.49862323	4.52059704	7.26279300	L
H	0	-6.58527023	4.28334904	7.79426500	L
C	0	-9.09621323	1.05736604	6.58411100	L H 179
C	0	-10.13630623	0.53221204	5.80095900	L
H	0	-9.95410323	0.24094904	4.77368900	L
C	0	-11.41743423	0.38673704	6.34020600	L
H	0	-12.21413623	-0.01760496	5.72928900	L
C	0	-11.66805823	0.76378504	7.66366800	L
C	0	-10.63458123	1.28465304	8.44929800	L
H	0	-10.82389023	1.57536604	9.47452000	L
C	0	-9.35179723	1.42909304	7.91356500	L
H	0	-8.55724223	1.82658904	8.53295000	L
C	0	-7.50565123	-1.31821796	5.96141500	L H 178
C	0	-7.89363623	-2.17111096	4.91755000	L
H	0	-7.83836223	-1.83489096	3.89045500	L
C	0	-8.35118523	-3.46231996	5.20038600	L
H	0	-8.64428923	-4.11919196	4.39210600	L
C	0	-8.42375223	-3.90538396	6.52540700	L
C	0	-8.04276523	-3.05564796	7.56824700	L
H	0	-8.09771423	-3.39485896	8.59456600	L
C	0	-7.58590623	-1.76556996	7.28856100	L
H	0	-7.28813423	-1.11706996	8.10344500	L
Br	0	2.29365877	5.80998904	-6.57040000	L
Br	0	-3.80065923	10.39331304	-8.55470400	L
Br	0	-9.66905223	6.94737704	2.80446900	L
Br	0	-10.00189823	10.29722604	-4.12390100	L
O	0	-0.03821423	2.27532604	1.27237800	H
O	0	-2.17738623	2.41082004	0.53962100	H
O	0	-3.91333123	5.49353704	1.70541600	H
O	0	-0.50175223	4.69036004	-1.22217300	H
N	0	-1.94327623	5.10821604	0.54903100	H
C	0	-1.12542423	2.88461004	1.08990300	H
C	0	-1.31284623	4.32124804	1.61083200	H
H	0	-2.11111023	4.21899004	2.35648100	H
C	0	-0.12815623	5.03206904	2.34080500	H
C	0	0.18457377	4.24207104	3.63086100	H
H	0	-0.70806823	4.13694204	4.25763200	H
H	0	0.56703377	3.24405704	3.40986400	H
H	0	0.94142877	4.77740404	4.21341900	H
C	0	-0.61335723	6.43938804	2.74992100	H
H	0	-0.80110423	7.07593504	1.88015300	H
H	0	-1.53485323	6.38539004	3.33745900	H
H	0	0.15000877	6.92850104	3.36296000	H
C	0	1.14548177	5.16655404	1.48564400	H
H	0	1.52659577	4.18995004	1.18143500	H
H	0	0.96912477	5.76035004	0.58538500	H
H	0	1.92138077	5.67078904	2.07226800	H
C	0	-3.25229123	5.58974104	0.69033600	H
C	0	-1.49863123	5.22832904	-0.77309000	H
C	0	-2.52466423	6.06941904	-1.48051800	H
C	0	-3.61386723	6.20594604	-0.61934000	H
C	0	-4.78934123	6.83466204	-1.00645600	H
C	0	-4.82489723	7.41982104	-2.29410300	H
C	0	-3.69645623	7.34114204	-3.15457400	H
C	0	-2.53434923	6.62193504	-2.76088100	H
C	0	-1.37606123	6.42672404	-3.67150800	L H 254

C	0	-0.08872823	6.83335404	-3.28329600	L	
H	0	0.07182977	7.29520704	-2.31687800	L	
C	0	0.99855477	6.64667904	-4.14194000	L	
H	0	1.98817677	6.95387504	-3.82943500	L	
C	0	0.80699177	6.06951404	-5.40104600	L	
C	0	-0.47418523	5.68054404	-5.80233100	L	
H	0	-0.62784123	5.23636704	-6.77755800	L	
C	0	-1.56022923	5.84962604	-4.93904300	L	
H	0	-2.54336523	5.52458904	-5.25543400	L	
C	0	-3.70921223	8.05491004	-4.46078800	L	H 253
C	0	-4.63355623	7.70347504	-5.45747500	L	
H	0	-5.33476623	6.89732004	-5.29182400	L	
C	0	-4.66168523	8.39829804	-6.66902200	L	
H	0	-5.38579823	8.12644304	-7.42600800	L	
C	0	-3.75796023	9.43998304	-6.90082300	L	
C	0	-2.82598423	9.78700304	-5.91769900	L	
H	0	-2.12509323	10.59294304	-6.09268600	L	
C	0	-2.80236623	9.09996004	-4.70084800	L	
H	0	-2.08462723	9.38666504	-3.94209700	L	
C	0	-6.06326323	8.11158604	-2.73656800	L	H 252
C	0	-7.23508223	7.37514004	-2.97303900	L	
H	0	-7.24149523	6.29950704	-2.84357900	L	
C	0	-8.40245823	8.02560804	-3.38090100	L	
H	0	-9.30274823	7.45238504	-3.55639400	L	
C	0	-8.40639023	9.41251204	-3.56084800	L	
C	0	-7.24148423	10.15072404	-3.32699100	L	
H	0	-7.24105123	11.22442104	-3.46327900	L	
C	0	-6.07365923	9.50414304	-2.91282800	L	
H	0	-5.17890823	10.08568004	-2.72789600	L	
C	0	-5.94989423	6.86323904	-0.08578500	L	H 251
C	0	-6.61887823	5.67212904	0.23851700	L	
H	0	-6.28720823	4.72755204	-0.17616900	L	
C	0	-7.72197823	5.69790304	1.09626000	L	
H	0	-8.23102723	4.77547304	1.34453700	L	
C	0	-8.16427123	6.91190604	1.63215900	L	
C	0	-7.50185223	8.10110004	1.31029200	L	
H	0	-7.84066123	9.04189804	1.72435700	L	
C	0	-6.39733023	8.07833504	0.45435900	L	
H	0	-5.89001423	9.00437804	0.21347400	L	
C	0	-4.24799323	0.54591204	-0.50599200	H	
C	0	-4.83369323	1.32961604	-1.54976500	H	
C	0	-5.18446323	-0.17638696	0.39202300	H	
C	0	-4.14616923	2.46606404	-2.07618800	H	
C	0	-6.10273123	0.96507604	-2.09539800	H	
O	0	-5.52421923	0.32083204	1.44435100	H	
O	0	-5.57774423	-1.39452096	-0.06043600	H	
C	0	-4.71483423	3.24789504	-3.07322600	H	
H	0	-3.18572823	2.72463704	-1.65386300	H	
C	0	-6.60876223	1.69820204	-3.14765900	H	
H	0	-6.62636723	0.09157004	-1.72671400	H	
C	0	-6.39010923	-2.20111796	0.80283500	H	
C	0	-5.93834923	2.83937704	-3.60478000	H	
H	0	-4.21654423	4.13412504	-3.45069500	H	
C	0	-7.84629523	1.47436104	-3.97712300	H	
C	0	-7.88368123	-2.14048696	0.42381400	H	
H	0	-6.05042523	-3.22808396	0.66936400	H	
H	0	-6.27230823	-1.89291296	1.84252400	H	
C	0	-6.73594223	3.44152204	-4.70841500	H	
N	0	-7.76599223	2.56882404	-4.94683400	H	
H	0	-8.76723423	1.53546304	-3.38380500	H	
H	0	-7.82771423	0.49779704	-4.47155000	H	
Cl	0	-8.11897423	-2.52798096	-1.31811600	H	
Cl	0	-8.72903323	-3.37437396	1.42427100	H	
Cl	0	-8.59753423	-0.52461096	0.75813400	H	
O	0	-6.54336223	4.50338004	-5.30262400	H	

C	0	-8.69985323	2.77621004	-6.05583200	H
C	0	-9.89201923	3.64341104	-5.57588300	H
C	0	-9.13459323	1.44988104	-6.68333000	H
H	0	-8.13840423	3.36452204	-6.79038500	H
N	0	-9.56276023	4.93964504	-5.36652100	H
O	0	-11.01782223	3.18132304	-5.40500800	H
C	0	-9.90349923	1.65081104	-7.99377100	H
H	0	-9.75644123	0.88971204	-5.98037300	H
H	0	-8.23675723	0.85539204	-6.87199900	H
H	0	-10.25545223	5.55872404	-4.97050100	H
H	0	-8.59640623	5.24099104	-5.43107200	H
C	0	-10.08433123	0.41091304	-8.85878500	H
H	0	-9.41286723	2.39346604	-8.63314900	H
H	0	-10.90335823	2.04723904	-7.78617400	H
O	0	-10.48084423	0.47248704	-10.00887600	H
O	0	-9.76213223	-0.71407696	-8.20398100	H
C	0	-9.81354323	-2.05025496	-8.84344200	H
C	0	-11.24588223	-2.36775096	-9.28136300	H
C	0	-9.37509323	-2.97922396	-7.70907700	H
C	0	-8.82088223	-2.09607596	-10.00831300	H
H	0	-11.29567023	-3.40496896	-9.62715700	H
H	0	-11.56775423	-1.71179996	-10.09018100	H
H	0	-11.93499023	-2.25766596	-8.43834900	H
H	0	-9.34680423	-4.01360296	-8.06327900	H
H	0	-8.37922323	-2.70924496	-7.34744800	H
H	0	-10.07374423	-2.92108296	-6.86948100	H
H	0	-7.81773323	-1.82670396	-9.66537800	H
H	0	-9.11673423	-1.41435196	-10.80602200	H
H	0	-8.77990323	-3.11162396	-10.41355700	H

Structure C

Rh	0	0.75797874	0.23936170	0.00000000	H
Rh	0	3.00840674	0.35525470	-1.00785500	H
Br	0	4.34449374	-7.70164230	3.88086700	L
Br	0	1.37251274	-7.70626330	10.78525800	L
Br	0	0.19018174	5.51870770	10.20170300	L
Br	0	-1.29211926	-1.21443330	13.85186800	L
O	0	3.73356274	0.90137470	0.83605700	H
O	0	1.69805674	0.70117870	1.81064200	H
O	0	2.14257774	2.35422670	5.49359800	H
O	0	3.34845074	-1.54037530	3.41329300	H
N	0	2.97527074	0.59315870	4.24690400	H
C	0	2.95002974	0.94973470	1.82347200	H
C	0	3.51047574	1.42957170	3.17412100	H
H	0	3.02341674	2.39978470	3.33371800	H
C	0	5.05534674	1.69015270	3.28696000	H
C	0	5.41701374	2.88955070	2.38337000	H
H	0	4.81519874	3.76961470	2.63498000	H
H	0	5.26315674	2.66189670	1.32778500	H
H	0	6.47019174	3.15174270	2.52841600	H
C	0	5.36844674	2.08906170	4.74618700	H
H	0	5.24553474	1.24852270	5.43597600	H
H	0	4.72563574	2.90424970	5.08720800	H
H	0	6.40858274	2.42179270	4.81642900	H
C	0	5.91834274	0.46736970	2.91981700	H
H	0	5.77154674	0.16780170	1.88111500	H
H	0	5.69556274	-0.39106330	3.55924700	H
H	0	6.97553774	0.71913270	3.05893300	H
C	0	2.34052174	1.16301670	5.35685900	H
C	0	2.97171374	-0.80350330	4.30793700	H
C	0	2.40617674	-1.15376030	5.65816200	H
C	0	1.99858274	0.03406570	6.26988200	H
C	0	1.44196474	0.06225770	7.54245100	H
C	0	1.29708174	-1.17422830	8.21837300	H
C	0	1.72556374	-2.38538930	7.61207700	H
C	0	2.28748074	-2.38193830	6.30569900	H

C	0	2.76001974	-3.62623630	5.64778900	L	H 35
C	0	4.11645574	-3.77581730	5.31559700	L	
H	0	4.81674674	-2.96962130	5.49681300	L	
C	0	4.58112274	-4.97775430	4.77457000	L	
H	0	5.62964874	-5.08622930	4.52920000	L	
C	0	3.69822974	-6.04231130	4.57100400	L	
C	0	2.34483374	-5.89676930	4.88954100	L	
H	0	1.66025174	-6.71829030	4.73081800	L	
C	0	1.87392374	-4.69251630	5.42109800	L	
H	0	0.82359874	-4.59559230	5.66889500	L	
C	0	1.63739474	-3.66512030	8.36882900	L	H 34
C	0	0.38927974	-4.18375030	8.75054900	L	
H	0	-0.52255726	-3.66150930	8.49451500	L	
C	0	0.31377574	-5.37626530	9.47456200	L	
H	0	-0.65184826	-5.76139530	9.77467100	L	
C	0	1.48102974	-6.06887830	9.80938300	L	
C	0	2.72723974	-5.56018230	9.43073100	L	
H	0	3.63372074	-6.09094230	9.69148900	L	
C	0	2.80701974	-4.36028330	8.71862700	L	
H	0	3.77881474	-3.97058230	8.44176900	L	
C	0	0.67997274	-1.18635830	9.57054600	L	H 33
C	0	-0.66997326	-0.83532830	9.72962200	L	
H	0	-1.26881926	-0.56113930	8.86954500	L	
C	0	-1.25318226	-0.84388930	10.99956400	L	
H	0	-2.29510326	-0.57395730	11.11342600	L	
C	0	-0.49285526	-1.20159630	12.11784800	L	
C	0	0.85357174	-1.54885830	11.96530100	L	
H	0	1.44574674	-1.82412730	12.82841800	L	
C	0	1.44021374	-1.53948930	10.69679300	L	
H	0	2.48513374	-1.80369930	10.59073400	L	
C	0	1.12407974	1.36202070	8.18511700	L	H 32
C	0	0.12148574	2.19301070	7.65980800	L	
H	0	-0.43479826	1.89092670	6.78277500	L	
C	0	-0.15419826	3.42741370	8.25587800	L	
H	0	-0.92257326	4.06612870	7.83915400	L	
C	0	0.56493974	3.83627670	9.38363400	L	
C	0	1.56143374	3.01114970	9.91370600	L	
H	0	2.12147474	3.32520470	10.78501600	L	
C	0	1.84216974	1.77947770	9.31680200	L	
H	0	2.62479874	1.15503570	9.72981900	L	
Br	0	-1.81212626	-5.00824930	-7.65550400	L	
Br	0	-7.69971926	-8.31502930	-4.42475000	L	
Br	0	-2.05589926	-7.28353330	7.40695700	L	
Br	0	-7.88287526	-9.46717330	3.04883300	L	
O	0	3.20979574	-1.67039530	-0.49697300	H	
O	0	1.10808874	-1.74922930	0.34326400	H	
O	0	0.59201874	-4.75262230	2.51133000	H	
O	0	0.72229074	-3.90524730	-1.97447900	H	
N	0	1.06649474	-4.39205230	0.26946800	H	
C	0	2.23648374	-2.26311430	0.04544300	H	
C	0	2.35368574	-3.73025230	0.50205000	H	
H	0	2.39922274	-3.65128030	1.59617600	H	
C	0	3.60443174	-4.56710630	0.07752400	H	
C	0	4.84146274	-3.97159430	0.78544700	H	
H	0	4.70592374	-3.95369330	1.87264600	H	
H	0	5.04459074	-2.95305530	0.44961300	H	
H	0	5.72104974	-4.58639930	0.56845200	H	
C	0	3.39851774	-6.00687330	0.59462000	H	
H	0	2.60955674	-6.52803730	0.04468300	H	
H	0	3.12974074	-6.01304630	1.65522000	H	
H	0	4.32376274	-6.57905230	0.47507800	H	
C	0	3.84055674	-4.60658230	-1.44349200	H	
H	0	4.01628374	-3.60661230	-1.84543700	H	
H	0	2.99015574	-5.04287130	-1.97281700	H	
H	0	4.72233374	-5.22088230	-1.65689000	H	

C	0	0.27454574	-4.83237330	1.33849400	H
C	0	0.34601774	-4.41322930	-0.93284800	H
C	0	-0.94184726	-5.12797630	-0.63794900	H
C	0	-0.98265526	-5.36506330	0.73769900	H
C	0	-2.06348326	-5.98413330	1.35223200	H
C	0	-3.14412926	-6.37347430	0.52853200	H
C	0	-3.10484626	-6.15077630	-0.87028500	H
C	0	-1.98795126	-5.51506330	-1.47665900	H
C	0	-1.90200726	-5.31746830	-2.94814600	L H 108
C	0	-0.84477426	-5.88779530	-3.67676300	L
H	0	-0.05884526	-6.43176830	-3.16738400	L
C	0	-0.81096426	-5.78300230	-5.07063300	L
H	0	0.00770374	-6.22627130	-5.62258000	L
C	0	-1.84233026	-5.12717030	-5.74994000	L
C	0	-2.89680226	-4.55638630	-5.03265300	L
H	0	-3.69542426	-4.04536630	-5.55471500	L
C	0	-2.91869426	-4.63376430	-3.63748900	L
H	0	-3.73602126	-4.17360030	-3.09669100	L
C	0	-4.20874926	-6.66872230	-1.71964700	L H 107
C	0	-5.46086326	-6.03445530	-1.72095300	L
H	0	-5.63023826	-5.15626630	-1.11071900	L
C	0	-6.49605026	-6.52574130	-2.52067600	L
H	0	-7.45931426	-6.03240530	-2.51404400	L
C	0	-6.28528426	-7.64717930	-3.32968300	L
C	0	-5.03709326	-8.27861130	-3.33683000	L
H	0	-4.86946626	-9.14566730	-3.96254300	L
C	0	-4.00145126	-7.79270730	-2.53370300	L
H	0	-3.03921426	-8.28979330	-2.54448400	L
C	0	-4.28219326	-7.11137730	1.12935400	L H 106
C	0	-5.41997926	-6.42092630	1.57234500	L
H	0	-5.47699326	-5.34352630	1.47838100	L
C	0	-6.48760126	-7.12089430	2.14117200	L
H	0	-7.36251526	-6.58200930	2.48106400	L
C	0	-6.42370226	-8.51242430	2.27094000	L
C	0	-5.28962726	-9.20386030	1.83120500	L
H	0	-5.23599726	-10.28029330	1.93044200	L
C	0	-4.22056826	-8.50594130	1.26200300	L
H	0	-3.34606126	-9.04876430	0.92480200	L
C	0	-2.03899426	-6.26630230	2.80644100	L H 105
C	0	-2.93144126	-5.61477230	3.67058700	L
H	0	-3.62282826	-4.87533630	3.28757100	L
C	0	-2.93814826	-5.92038130	5.03410300	L
H	0	-3.63889026	-5.42306130	5.69197800	L
C	0	-2.04336926	-6.86547330	5.54532900	L
C	0	-1.14523526	-7.51077030	4.68988200	L
H	0	-0.45370526	-8.24562530	5.08132000	L
C	0	-1.14245326	-7.21395730	3.32404400	L
H	0	-0.44944626	-7.72704330	2.66851700	L
Br	0	1.55519174	7.78567170	-6.10761100	L
Br	0	-5.17459326	8.05146870	-9.95142200	L
Br	0	-7.62511726	-4.34211230	-5.34785400	L
Br	0	-10.33993826	2.32760670	-8.61253200	L
O	0	2.18583374	-0.22769430	-2.80897300	H
O	0	0.09702474	-0.21786430	-1.93620500	H
O	0	-2.41733326	-1.63458930	-4.63942600	H
O	0	0.34274074	2.01160270	-4.58894200	H
N	0	-0.73373626	-0.04445430	-4.63454200	H
C	0	0.94065374	-0.40161230	-2.87998500	H
C	0	0.32546874	-0.94913630	-4.18219600	H
H	0	-0.21957226	-1.84324530	-3.85854100	H
C	0	1.28921874	-1.41286030	-5.32969600	H
C	0	2.15477374	-2.57573730	-4.79861400	H
H	0	1.53396174	-3.38623030	-4.40443600	H
H	0	2.82903274	-2.24785330	-4.00560600	H
H	0	2.76086774	-2.97929830	-5.61642300	H

C	0	0.41799174	-1.96067130	-6.48151900	H
H	0	-0.16666026	-1.17263830	-6.96596700	H
H	0	-0.27405826	-2.72945430	-6.12777200	H
H	0	1.06142474	-2.40853130	-7.24501100	H
C	0	2.19432274	-0.29611030	-5.88459300	H
H	0	2.84204274	0.11759770	-5.11053400	H
H	0	1.61470474	0.52137170	-6.32122000	H
H	0	2.82608174	-0.71114930	-6.67773100	H
C	0	-2.03569826	-0.50496330	-4.87095600	H
C	0	-0.62126426	1.32789770	-4.88261400	H
C	0	-1.91695926	1.73687670	-5.52605300	H
C	0	-2.78493226	0.64821870	-5.45532800	H
C	0	-4.09168226	0.71338670	-5.92068500	H
C	0	-4.50586426	1.91240770	-6.55414000	H
C	0	-3.60718526	3.00894570	-6.68225000	H
C	0	-2.30053126	2.93368870	-6.12341200	H
C	0	-1.37359026	4.09177570	-6.11986800	L H 181
C	0	-0.14731326	4.02428070	-6.80002500	L
H	0	0.13376974	3.12461470	-7.33363700	L
C	0	0.71920574	5.12134270	-6.79727800	L
H	0	1.66447774	5.06010670	-7.32072800	L
C	0	0.36671074	6.29179470	-6.11716900	L
C	0	-0.85572626	6.36566670	-5.44190300	L
H	0	-1.13291426	7.26882770	-4.91362000	L
C	0	-1.72335226	5.27074770	-5.44374800	L
H	0	-2.66575426	5.33940270	-4.91848100	L
C	0	-3.99390126	4.21925270	-7.46392800	L H 180
C	0	-5.09459426	5.00527570	-7.08308800	L
H	0	-5.68851126	4.73875470	-6.22306900	L
C	0	-5.44396026	6.13879070	-7.82113400	L
H	0	-6.29565126	6.73440570	-7.51848400	L
C	0	-4.69705626	6.50022070	-8.94587900	L
C	0	-3.60293126	5.72215970	-9.33584300	L
H	0	-3.02424826	5.99578370	-10.20860300	L
C	0	-3.25417026	4.58522470	-8.60154000	L
H	0	-2.40982326	3.98703270	-8.92117000	L
C	0	-5.90121626	2.01349170	-7.05526300	L H 179
C	0	-6.97347026	1.99311570	-6.14919000	L
H	0	-6.78876426	1.90687370	-5.08563400	L
C	0	-8.28773026	2.08679270	-6.61226900	L
H	0	-9.10658026	2.06799270	-5.90602100	L
C	0	-8.54158626	2.20187270	-7.98260400	L
C	0	-7.47753626	2.21996070	-8.89084300	L
H	0	-7.66990026	2.30640070	-9.95238800	L
C	0	-6.16094626	2.12245370	-8.43058000	L
H	0	-5.34499126	2.13069070	-9.14262600	L
C	0	-4.97304626	-0.47129530	-5.78640500	L H 178
C	0	-5.35324726	-0.92710430	-4.51367400	L
H	0	-5.02898426	-0.39978530	-3.62450300	L
C	0	-6.14071426	-2.07482030	-4.38449600	L
H	0	-6.41939526	-2.42731330	-3.39971000	L
C	0	-6.56030426	-2.76817230	-5.52446600	L
C	0	-6.19402426	-2.31126030	-6.79478100	L
H	0	-6.51521926	-2.84655830	-7.67890800	L
C	0	-5.40204626	-1.16716130	-6.92688900	L
H	0	-5.11082026	-0.83182930	-7.91468100	L
Br	0	5.69150974	5.53365470	6.48549400	L
Br	0	-0.12073826	9.41677770	9.54201800	L
Br	0	-6.92879726	8.96416770	-1.17622200	L
Br	0	-6.32177626	10.72907370	5.57476100	L
O	0	2.58919474	2.37342670	-1.42136200	H
O	0	0.57929474	2.26532070	-0.38540200	H
O	0	-1.49622426	5.54402370	-1.08317400	H
O	0	2.19116974	4.39932070	1.37476400	H
N	0	0.56293174	4.97077870	-0.17668900	H

C	0	1.50147974	2.87308770	-1.02403900	H
C	0	1.13033974	4.33416870	-1.37177900	H
H	0	0.27138074	4.23077670	-2.04539300	H
C	0	2.17461174	5.22131870	-2.12984600	H
C	0	2.44472174	4.58388670	-3.51043900	H
H	0	1.51283374	4.36013170	-4.03890100	H
H	0	3.00782674	3.65421270	-3.41874700	H
H	0	3.02519674	5.27874270	-4.12616300	H
C	0	1.53870874	6.61035770	-2.35705100	H
H	0	1.38093874	7.14666370	-1.41648900	H
H	0	0.57552074	6.53589770	-2.87139400	H
H	0	2.20590874	7.21954070	-2.97418200	H
C	0	3.50260474	5.39975270	-1.37020300	H
H	0	3.98331074	4.43914870	-1.17622100	H
H	0	3.36200874	5.91290870	-0.41535800	H
H	0	4.18327374	6.00806570	-1.97588000	H
C	0	-0.71496826	5.53666670	-0.14309800	H
C	0	1.15872374	4.98069970	1.09594200	H
C	0	0.25613674	5.79387170	1.97418700	H
C	0	-0.89093526	6.09524770	1.23221300	H
C	0	-1.93440426	6.83552470	1.77311900	H
C	0	-1.80201126	7.27805570	3.11005000	H
C	0	-0.63205326	6.99634770	3.85628000	H
C	0	0.42740774	6.24240870	3.28395700	H
C	0	1.69790074	5.99462970	4.01559500	L H 254
C	0	2.91690874	6.45258070	3.48802600	L
H	0	2.94646874	6.94512370	2.52384700	L
C	0	4.10138574	6.30272370	4.21508900	L
H	0	5.03473474	6.66051670	3.80010300	L
C	0	4.07703874	5.71039870	5.48123000	L
C	0	2.86861374	5.25187070	6.01347900	L
H	0	2.84484974	4.79014870	6.99247300	L
C	0	1.68557474	5.38288770	5.28062700	L
H	0	0.75802974	5.01967670	5.70473800	L
C	0	-0.49169326	7.55617670	5.22676100	L H 253
C	0	-1.31248226	7.09482970	6.26828700	L
H	0	-2.03781726	6.31122270	6.08731700	L
C	0	-1.20160326	7.64704370	7.54705500	L
H	0	-1.84362826	7.29112870	8.34230000	L
C	0	-0.26534826	8.65597370	7.79660700	L
C	0	0.56131274	9.11231170	6.76487600	L
H	0	1.28644674	9.89329770	6.95364800	L
C	0	0.44759074	8.56745170	5.48301300	L
H	0	1.08408274	8.93688670	4.68838300	L
C	0	-2.88368826	8.10419370	3.70508200	L H 252
C	0	-4.08302126	7.50526670	4.11868900	L
H	0	-4.22472526	6.43662170	4.01494900	L
C	0	-5.10224726	8.28501670	4.67228700	L
H	0	-6.02504826	7.81623170	4.98865500	L
C	0	-4.92844926	9.66548670	4.81768700	L
C	0	-3.73317426	10.26620270	4.40824200	L
H	0	-3.59479626	11.33390870	4.51899800	L
C	0	-2.71311726	9.48897770	3.85240300	L
H	0	-1.79256026	9.96313970	3.53487500	L
C	0	-3.08178726	7.26026170	0.94244500	L H 251
C	0	-4.26734226	6.51122270	0.93290500	L
H	0	-4.31882626	5.56152670	1.45101900	L
C	0	-5.40718626	7.01415170	0.29668400	L
H	0	-6.32849026	6.44892470	0.31798300	L
C	0	-5.36356526	8.25797670	-0.34252600	L
C	0	-4.17439826	8.99334570	-0.35795100	L
H	0	-4.13637626	9.95638170	-0.85029300	L
C	0	-3.03695826	8.49804570	0.28469200	L
H	0	-2.13209126	9.09314570	0.30120200	L
C	0	-1.11166826	0.11711170	0.80754500	H

C	0	-2.22755226	0.92485670	0.44130900	H
C	0	-1.32940926	-0.97342030	1.78456400	H
C	0	-3.34787926	1.09099070	1.31897000	H
C	0	-2.21593326	1.59731870	-0.81380200	H
O	0	-1.66636826	-2.07421830	1.39819000	H
O	0	-1.07205126	-0.63813030	3.07043900	H
C	0	-4.39581826	1.93614270	0.99055000	H
H	0	-3.35581526	0.57492070	2.27224700	H
C	0	-3.29645426	2.38713470	-1.14619700	H
H	0	-1.39124426	1.44480770	-1.49409100	H
C	0	-1.11283726	-1.68147730	4.05245700	H
C	0	-4.35090826	2.57263170	-0.25253300	H
H	0	-5.23305526	2.09191570	1.66224700	H
C	0	-3.57495126	3.15366770	-2.40244500	H
C	0	-2.35492826	-1.55473030	4.94998500	H
H	0	-1.10630926	-2.66329930	3.57681200	H
H	0	-0.22923826	-1.55957130	4.67880600	H
C	0	-5.35668126	3.48968170	-0.86685200	H
N	0	-4.83932026	3.83912770	-2.09894900	H
H	0	-3.70162026	2.48687970	-3.26426200	H
H	0	-2.77580326	3.86472070	-2.63830400	H
Cl	0	-3.86702726	-1.86410130	4.02435000	H
Cl	0	-2.18870726	-2.78325430	6.25237400	H
Cl	0	-2.44899426	0.08274970	5.68659700	H
O	0	-6.41992026	3.83782970	-0.36647000	H
C	0	-5.49058126	4.58240970	-3.18066900	H
C	0	-5.01138226	6.04427670	-3.34906300	H
C	0	-7.02984426	4.45420670	-3.29015600	H
H	0	-5.10601426	4.09622070	-4.08434500	H
N	0	-3.93735926	6.44285370	-2.63496500	H
O	0	-5.56805726	6.76540670	-4.17649000	H
C	0	-7.88710026	5.46696370	-2.51511200	H
H	0	-7.30522226	3.43225970	-3.01375900	H
H	0	-7.26373126	4.57378970	-4.34850200	H
H	0	-3.57932926	7.36843670	-2.82212300	H
H	0	-3.45128726	5.88407970	-1.94821600	H
C	0	-9.35195026	5.42545270	-2.91287400	H
H	0	-7.53208326	6.47421170	-2.75303500	H
H	0	-7.81813626	5.30527370	-1.44159800	H
O	0	-10.27486826	5.35649470	-2.12074800	H
O	0	-9.48198926	5.51148470	-4.24972500	H
C	0	-10.79269726	5.54760270	-4.93105600	H
C	0	-11.56085726	4.25073570	-4.66116500	H
C	0	-10.39346226	5.65085670	-6.40497800	H
C	0	-11.57193526	6.78968070	-4.48874400	H
H	0	-12.47316326	4.23605470	-5.26558100	H
H	0	-11.83563626	4.16691070	-3.60938200	H
H	0	-10.95686926	3.38238270	-4.93924200	H
H	0	-11.28563526	5.65906670	-7.03750500	H
H	0	-9.82792126	6.56885970	-6.58933500	H
H	0	-9.77050026	4.80023270	-6.69440500	H
H	0	-10.97098926	7.69103370	-4.64419600	H
H	0	-11.85035626	6.72704070	-3.43629500	H
H	0	-12.48307326	6.88077970	-5.08833300	H

Structure D

Rh	0	-1.15691492	-0.18617021	0.00000000	H
Rh	0	1.17783408	-0.03636821	0.79760900	H
Br	0	2.16678308	-7.89540721	-4.76350300	L
Br	0	-1.56075392	-7.67144521	-11.07926600	L
Br	0	-3.47681592	5.30241579	-9.86863500	L
Br	0	-4.48187092	-1.00187721	-13.70247800	L
O	0	1.69211608	0.65668579	-1.07271900	H
O	0	-0.40795992	0.39227379	-1.87799000	H
O	0	-0.33481592	2.21443279	-5.52449500	H
O	0	1.01769308	-1.78442321	-3.73237100	H

N	0	0.59584708	0.39005479	-4.42838200	H
C	0	0.83090108	0.67079779	-1.99612900	H
C	0	1.29050808	1.14848479	-3.38941200	H
H	0	0.87294908	2.15873179	-3.48462800	H
C	0	2.83953208	1.28611779	-3.64419100	H
C	0	3.39990508	2.41155579	-2.74442500	H
H	0	2.77041508	3.30576579	-2.76143200	H
H	0	3.47693308	2.08806879	-1.70625300	H
H	0	4.39880008	2.69436879	-3.09254500	H
C	0	3.04310008	1.68940479	-5.12226700	H
H	0	2.79714908	0.87007879	-5.80439300	H
H	0	2.43318108	2.55080479	-5.40389400	H
H	0	4.09302808	1.94903679	-5.28743000	H
C	0	3.63608608	-0.00873821	-3.38718000	H
H	0	3.51710208	-0.35591221	-2.35960700	H
H	0	3.33735108	-0.81549221	-4.06023000	H
H	0	4.69986008	0.19010779	-3.55904900	H
C	0	-0.13758592	1.01581079	-5.44844200	H
C	0	0.57875608	-1.00269321	-4.55795600	H
C	0	-0.09262092	-1.28769021	-5.86926300	H
C	0	-0.56942692	-0.07565521	-6.37408400	H
C	0	-1.25643492	-0.00375121	-7.57815500	H
C	0	-1.44617092	-1.20591121	-8.30188600	H
C	0	-0.93766992	-2.43520421	-7.80915500	H
C	0	-0.25583292	-2.48501721	-6.56255700	H
C	0	0.31025408	-3.74846021	-6.02628200	L H 35
C	0	1.69878308	-3.88323521	-5.86429800	L
H	0	2.35776508	-3.04883121	-6.07105100	L
C	0	2.24652208	-5.10769621	-5.47147200	L
H	0	3.31878908	-5.20580821	-5.36206900	L
C	0	1.41354508	-6.20714621	-5.24354900	L
C	0	0.02880608	-6.07322121	-5.37953900	L
H	0	-0.61730092	-6.92056421	-5.19741700	L
C	0	-0.52337092	-4.84874821	-5.76602800	L
H	0	-1.59647492	-4.76428821	-5.88382800	L
C	0	-1.09019892	-3.67932321	-8.61327600	L H 34
C	0	-2.36513492	-4.20992321	-8.86891400	L
H	0	-3.25031892	-3.71004821	-8.50038000	L
C	0	-2.50302992	-5.39131821	-9.60200100	L
H	0	-3.48992392	-5.79342621	-9.79106300	L
C	0	-1.36996992	-6.05180921	-10.08648600	L
C	0	-0.09754892	-5.52466121	-9.84391000	L
H	0	0.78199108	-6.03110021	-10.21986100	L
C	0	0.04315508	-4.34139121	-9.11345200	L
H	0	1.03373908	-3.94206421	-8.93407400	L
C	0	-2.17227192	-1.15988321	-9.59704700	L H 33
C	0	-3.54900092	-0.88750321	-9.62324900	L
H	0	-4.09067192	-0.71782621	-8.70064200	L
C	0	-4.23234092	-0.84019421	-10.84133900	L
H	0	-5.29403092	-0.63021421	-10.85328800	L
C	0	-3.54613392	-1.06549821	-12.03930600	L
C	0	-2.17403892	-1.33740421	-12.01814300	L
H	0	-1.63944992	-1.51158321	-12.94296600	L
C	0	-1.48750692	-1.38326421	-10.80153200	L
H	0	-0.42435192	-1.58990321	-10.79607400	L
C	0	-1.74175592	1.29979579	-8.08459300	L H 32
C	0	-2.88219792	1.88914879	-7.52223200	L
H	0	-3.38562992	1.41995479	-6.68829900	L
C	0	-3.39845492	3.06999279	-8.05942500	L
H	0	-4.30090192	3.49368379	-7.64532700	L
C	0	-2.76308792	3.68733379	-9.14069400	L
C	0	-1.61189292	3.11444079	-9.69221600	L
H	0	-1.11749392	3.58706079	-10.53098400	L
C	0	-1.10585092	1.91907679	-9.17136500	L
H	0	-0.22728592	1.47067079	-9.61867200	L

Br	0	-1.01026892	-6.33796821	7.59356500	L
Br	0	-7.61163192	-9.83642621	5.64188100	L
Br	0	-4.98731492	-7.22275221	-7.09233100	L
Br	0	-9.70416692	-10.14605721	-1.64060600	L
O	0	1.45483208	-1.97527221	0.09518100	H
O	0	-0.72753492	-2.15037421	-0.47941000	H
O	0	-1.42522492	-5.08644421	-2.66594900	H
O	0	-0.40336092	-4.52762521	1.74890200	H
N	0	-0.51386192	-4.82164021	-0.55008700	H
C	0	0.46222108	-2.59637221	-0.37578100	H
C	0	0.63095608	-4.00971721	-0.96759300	H
H	0	0.45942608	-3.85836521	-2.04061100	H
C	0	2.01524108	-4.73000121	-0.84571100	H
C	0	3.03342508	-3.95660721	-1.71179300	H
H	0	2.68991108	-3.86835121	-2.74761100	H
H	0	3.20441808	-2.95031421	-1.32461400	H
H	0	3.99030908	-4.48881521	-1.71682100	H
C	0	1.85022608	-6.14606821	-1.43687200	H
H	0	1.24699508	-6.78959321	-0.78988800	H
H	0	1.36848208	-6.11167221	-2.41796300	H
H	0	2.83145808	-6.61602021	-1.55502200	H
C	0	2.54737008	-4.85025221	0.59499000	H
H	0	2.69794908	-3.86887621	1.04869500	H
H	0	1.86860408	-5.42089721	1.23253200	H
H	0	3.51115708	-5.37141021	0.57751700	H
C	0	-1.47625792	-5.27450421	-1.46353500	H
C	0	-0.94050692	-5.01296521	0.76799200	H
C	0	-2.17804492	-5.85174621	0.68928500	H
C	0	-2.52162692	-5.97678421	-0.65730900	H
C	0	-3.67894892	-6.63310621	-1.06277200	H
C	0	-4.51321792	-7.16282621	-0.04918200	H
C	0	-4.14504092	-7.06975321	1.31660300	H
C	0	-2.95065792	-6.41234221	1.70350400	H
C	0	-2.48226792	-6.37333921	3.11269100	L H 108
C	0	-1.24198292	-6.93623721	3.45738400	L
H	0	-0.61607492	-7.38919821	2.69824100	L
C	0	-0.80621192	-6.92377121	4.78533700	L
H	0	0.15324508	-7.35515621	5.03971100	L
C	0	-1.60894992	-6.35901221	5.78082200	L
C	0	-2.84744892	-5.80316921	5.44613200	L
H	0	-3.47080892	-5.36102421	6.21262600	L
C	0	-3.27889992	-5.80204321	4.11750600	L
H	0	-4.23167292	-5.35287421	3.87276500	L
C	0	-4.98041192	-7.73606321	2.34579100	L H 107
C	0	-6.12174992	-7.09678921	2.85083400	L
H	0	-6.39380592	-6.11108021	2.49718500	L
C	0	-6.90147592	-7.72002721	3.82848500	L
H	0	-7.77752492	-7.21744821	4.21762000	L
C	0	-6.54609692	-8.98623121	4.30503300	L
C	0	-5.40747492	-9.62722621	3.80417400	L
H	0	-5.12799992	-10.60603321	4.17192200	L
C	0	-4.62489592	-9.00399421	2.82754800	L
H	0	-3.74375092	-9.50585921	2.44706600	L
C	0	-5.75639192	-7.87789821	-0.42835900	L H 106
C	0	-6.99910692	-7.23520921	-0.32531000	L
H	0	-7.05787892	-6.21206921	0.02465900	L
C	0	-8.16945992	-7.90944321	-0.68346600	L
H	0	-9.12478392	-7.40749521	-0.60048300	L
C	0	-8.10423992	-9.22662421	-1.15021200	L
C	0	-6.86587092	-9.86878721	-1.25979100	L
H	0	-6.81112392	-10.88707121	-1.62244800	L
C	0	-5.69384092	-9.19680221	-0.90060700	L
H	0	-4.73943792	-9.70131921	-0.98814000	L
C	0	-3.99476292	-6.77337321	-2.50506800	L H 105
C	0	-5.14749792	-6.17886321	-3.04079800	L

H	0	-5.80662692	-5.59620621	-2.40999700	L
C	0	-5.44949692	-6.32613521	-4.39731900	L
H	0	-6.33914092	-5.86138221	-4.80225700	L
C	0	-4.59538792	-7.05499721	-5.23112200	L
C	0	-3.44759792	-7.65270521	-4.70247900	L
H	0	-2.78816392	-8.22328821	-5.34341300	L
C	0	-3.14799792	-7.51538721	-3.34401700	L
H	0	-2.25760892	-7.98602821	-2.94530400	L
Br	0	-1.07894492	6.74059579	6.88809400	L
Br	0	-8.75867992	6.77169979	7.91867800	L
Br	0	-9.30114992	-5.12327121	2.14523900	L
Br	0	-12.86812292	0.41197279	5.26303000	L
O	0	0.56083708	-0.82646621	2.61116600	H
O	0	-1.60480192	-0.76108021	1.95029000	H
O	0	-3.86450992	-2.72233821	3.94976100	H
O	0	-1.51410992	1.09826579	4.83444300	H
N	0	-2.37692292	-1.03089021	4.50661600	H
C	0	-0.67144692	-1.03006321	2.78131000	H
C	0	-1.16905192	-1.72663121	4.06512800	H
H	0	-1.55256892	-2.68797421	3.70120600	H
C	0	-0.14541492	-2.07689521	5.20011700	H
C	0	0.76172908	-3.21167121	4.67601100	H
H	0	0.17580408	-4.07841221	4.35539200	H
H	0	1.36263108	-2.88074721	3.82611800	H
H	0	1.44219508	-3.53489021	5.47066500	H
C	0	-0.95157192	-2.60947821	6.40373800	H
H	0	-1.51906692	-1.81469421	6.89652700	H
H	0	-1.65848992	-3.38723221	6.10312800	H
H	0	-0.26863392	-3.04021521	7.14252700	H
C	0	0.73362608	-0.90463821	5.67924400	H
H	0	1.34182908	-0.50299521	4.86709400	H
H	0	0.13668008	-0.09073921	6.09387200	H
H	0	1.40697108	-1.26732021	6.46414500	H
C	0	-3.65381192	-1.58728421	4.33130100	H
C	0	-2.45570292	0.32524579	4.83221300	H
C	0	-3.90041192	0.60543779	5.09907000	H
C	0	-4.62824092	-0.51815321	4.70686500	H
C	0	-6.01841092	-0.52786121	4.69918800	H
C	0	-6.67860692	0.62581979	5.19310300	H
C	0	-5.93542992	1.74309679	5.65459900	H
C	0	-4.52119192	1.75686479	5.56934800	H
C	0	-3.71385792	2.95624579	5.90508500	L H 181
C	0	-2.75913292	2.90529379	6.93308000	L
H	0	-2.61419992	1.99412879	7.50042600	L
C	0	-1.98130192	4.02946279	7.22693900	L
H	0	-1.24032692	3.97859379	8.01414800	L
C	0	-2.15740092	5.21330279	6.50285700	L
C	0	-3.11572992	5.27382279	5.48677700	L
H	0	-3.25532292	6.18742979	4.92364300	L
C	0	-3.88731892	4.14909479	5.18458100	L
H	0	-4.61162092	4.20478679	4.38365700	L
C	0	-6.63638192	2.92687879	6.21030700	L H 180
C	0	-7.38497392	3.75410479	5.36498100	L
H	0	-7.45194592	3.51894679	4.31264600	L
C	0	-8.01687692	4.89278779	5.87174200	L
H	0	-8.58688192	5.53200779	5.21051800	L
C	0	-7.90389192	5.20954879	7.22919100	L
C	0	-7.15692092	4.38584079	8.07860100	L
H	0	-7.06397392	4.62908579	9.12909600	L
C	0	-6.52141092	3.24841779	7.57106200	L
H	0	-5.93880392	2.62060879	8.23391500	L
C	0	-8.16303592	0.65811479	5.21301000	L H 179
C	0	-8.88474492	0.72844679	4.01077000	L
H	0	-8.36386092	0.80612379	3.06452200	L
C	0	-10.28144792	0.67458879	4.02651900	L

H	0	-10.83078892	0.71675279	3.09490400	L
C	0	-10.96379192	0.54650579	5.24142900	L
C	0	-10.24919292	0.50422779	6.44312300	L
H	0	-10.77407192	0.41250579	7.38514600	L
C	0	-8.85309492	0.56241379	6.43070100	L
H	0	-8.30827492	0.50593579	7.36502300	L
C	0	-6.75261192	-1.67749921	4.11373700	L H 178
C	0	-6.60371592	-1.97950221	2.75055300	L
H	0	-5.92454792	-1.40270021	2.13655100	L
C	0	-7.34550192	-3.01272021	2.17133200	L
H	0	-7.23210292	-3.22916621	1.11665200	L
C	0	-8.24338292	-3.75079421	2.94933000	L
C	0	-8.38020992	-3.46955321	4.31304000	L
H	0	-9.07061892	-4.04154221	4.91930700	L
C	0	-7.63703892	-2.43766721	4.89513800	L
H	0	-7.75952292	-2.22267321	5.94933500	L
Br	0	5.92059808	6.21932579	-4.28247900	L
Br	0	0.89086408	8.37104679	-9.71322000	L
Br	0	-9.02261892	4.23876879	-2.61258100	L
Br	0	-6.66569892	7.90266679	-8.27368400	L
O	0	0.74541208	1.92125879	1.41854800	H
O	0	-1.32345192	1.81766479	0.50351000	H
O	0	-3.22616092	4.67839279	0.25025500	H
O	0	1.21794708	4.42738879	-0.83604100	H
N	0	-0.91197092	4.56809979	0.09637700	H
C	0	-0.36504392	2.42153879	1.09712000	H
C	0	-0.71475192	3.89995179	1.39496300	H
H	0	-1.73398792	3.84594479	1.78943300	H
C	0	0.09836008	4.68727279	2.46957800	H
C	0	-0.01577792	3.89799279	3.79156400	H
H	0	-1.05493292	3.66659879	4.04348700	H
H	0	0.52859508	2.95287379	3.74302500	H
H	0	0.40424908	4.49060679	4.61066100	H
C	0	-0.59075692	6.05708579	2.64951100	H
H	0	-0.51470992	6.66981179	1.74586800	H
H	0	-1.65262392	5.94495579	2.89369600	H
H	0	-0.11417192	6.60654479	3.46740800	H
C	0	1.58763608	4.91066979	2.14737500	H
H	0	2.10145108	3.96528479	1.96548700	H
H	0	1.72759308	5.54857879	1.27289600	H
H	0	2.06273508	5.40302679	3.00341000	H
C	0	-2.20420592	4.81284079	-0.39888000	H
C	0	0.04971708	4.75726479	-0.91014600	H
C	0	-0.67608692	5.35800079	-2.07618400	H
C	0	-2.04250892	5.27296379	-1.80977200	H
C	0	-2.99962492	5.60122579	-2.76414300	H
C	0	-2.53624792	6.10718379	-4.00655700	H
C	0	-1.14572692	6.23812279	-4.25992900	H
C	0	-0.19044292	5.83820579	-3.28985500	H
C	0	1.27369708	5.92732279	-3.52827900	L H 254
C	0	2.08601308	6.68396779	-2.66749800	L
H	0	1.65207508	7.20750379	-1.82441700	L
C	0	3.46322208	6.76927479	-2.89144100	L
H	0	4.08157108	7.34891779	-2.21833100	L
C	0	4.03849008	6.11008279	-3.98222600	L
C	0	3.23458308	5.36716879	-4.85128100	L
H	0	3.67596808	4.85541679	-5.69661900	L
C	0	1.85915408	5.27080179	-4.62317500	L
H	0	1.25205508	4.67731879	-5.29386600	L
C	0	-0.67913292	6.76208879	-5.57061400	L H 253
C	0	-0.87464592	6.01164679	-6.74149300	L
H	0	-1.37517492	5.05219479	-6.69929900	L
C	0	-0.40500892	6.48928279	-7.96779600	L
H	0	-0.54677992	5.90047779	-8.86402400	L
C	0	0.25322008	7.72111879	-8.03509900	L

C	0	0.44742008	8.47410979	-6.87237700	L
H	0	0.95883508	9.42676979	-6.92012700	L
C	0	-0.01441592	7.99614879	-5.64279900	L
H	0	0.14549908	8.58414579	-4.74742800	L
C	0	-3.52004392	6.53375379	-5.03901800	L H 252
C	0	-4.36629192	5.59335279	-5.64716700	L
H	0	-4.30096892	4.54628279	-5.37913900	L
C	0	-5.30535392	6.00292379	-6.59807900	L
H	0	-5.96098892	5.27339779	-7.05528000	L
C	0	-5.39398492	7.34991979	-6.96139700	L
C	0	-4.55062992	8.29110679	-6.36303800	L
H	0	-4.61825392	9.33548879	-6.63886000	L
C	0	-3.62084192	7.88694879	-5.40109900	L
H	0	-2.98207692	8.62702579	-4.93510900	L
C	0	-4.43140892	5.29523279	-2.52863000	L H 251
C	0	-4.85032192	3.95637179	-2.45854600	L
H	0	-4.12314492	3.15327779	-2.46521700	L
C	0	-6.21261592	3.64558079	-2.45302500	L
H	0	-6.52616792	2.60990779	-2.44150300	L
C	0	-7.16479292	4.66804179	-2.50554600	L
C	0	-6.75330892	6.00469479	-2.52448800	L
H	0	-7.48741792	6.79874279	-2.56561300	L
C	0	-5.39126192	6.31943979	-2.53284500	L
H	0	-5.08453492	7.35633979	-2.59263700	L
C	0	-3.13642392	-0.47497121	-0.57337300	H
C	0	-4.36538892	0.15315079	-0.19397000	H
C	0	-3.29724392	-1.68048121	-1.43194300	H
C	0	-5.62259992	-0.44583721	-0.54990400	H
C	0	-4.36947492	1.41929679	0.46849100	H
O	0	-3.48939092	-2.79653721	-0.99809100	H
O	0	-3.18481192	-1.35406021	-2.74309700	H
C	0	-6.82611092	0.18631279	-0.30091100	H
H	0	-5.63162492	-1.42218021	-1.01632000	H
C	0	-5.58376692	2.05443579	0.68205200	H
H	0	-3.42638292	1.88177879	0.73063600	H
C	0	-3.25945592	-2.39345621	-3.72195700	H
C	0	-6.78115592	1.44659079	0.29679900	H
H	0	-7.77355792	-0.26132421	-0.58059200	H
C	0	-5.89008192	3.41918279	1.23829100	H
C	0	-4.56358092	-2.28693321	-4.53103600	H
H	0	-2.42577492	-2.24333821	-4.40827100	H
H	0	-3.19015892	-3.37934221	-3.26018100	H
C	0	-7.90998492	2.39269279	0.52545000	H
N	0	-7.35423292	3.51543879	1.09305900	H
H	0	-5.37890992	4.20222679	0.67151900	H
H	0	-5.60020692	3.52628679	2.28624000	H
Cl	0	-4.77977892	-0.62819221	-5.18789500	H
Cl	0	-4.44015592	-3.45353021	-5.89259800	H
Cl	0	-5.99796092	-2.70446121	-3.52514600	H
O	0	-9.08657292	2.21720579	0.21570100	H
C	0	-8.09694692	4.78264679	1.18573600	H
C	0	-7.19169392	5.88536979	1.76715100	H
C	0	-9.37607992	4.65490379	2.04195800	H
H	0	-8.40182292	5.06365679	0.17045400	H
N	0	-7.09709392	7.00599179	1.01226100	H
O	0	-6.62774692	5.75614679	2.84962200	H
C	0	-10.18139792	5.96331179	2.09818800	H
H	0	-9.10469992	4.35647479	3.05176100	H
H	0	-9.98350392	3.85808579	1.61011800	H
H	0	-6.58278192	7.79449579	1.37924000	H
H	0	-7.60002792	7.12646279	0.14756000	H
C	0	-11.47535392	5.83681979	2.88570500	H
H	0	-10.45181492	6.31364779	1.09833500	H
H	0	-9.58987192	6.75423879	2.57602400	H
O	0	-12.54988292	6.24925579	2.48814500	H

O	0	-11.25731292	5.23624879	4.06554800	H
C	0	-12.35130292	4.92922379	5.01411900	H
C	0	-12.97866292	6.22970479	5.52201400	H
C	0	-11.61747392	4.20166279	6.14227800	H
C	0	-13.36980392	4.00695379	4.33842800	H
H	0	-13.69818292	6.00089579	6.31452200	H
H	0	-13.49641692	6.75811279	4.72091300	H
H	0	-12.20842992	6.88439479	5.94142200	H
H	0	-12.33391992	3.83242679	6.88115800	H
H	0	-11.05355292	3.35341079	5.74733200	H
H	0	-10.91830992	4.87324079	6.64622100	H
H	0	-12.86098092	3.14767579	3.89144500	H
H	0	-13.92294192	4.52967579	3.55804700	H
H	0	-14.07894292	3.63607579	5.08466600	H

Structure A-SI-1

ONIOM total E = -10262.097654 Hartree

ZPE = 2.604137 Hartree

TCH = 2.816067 Hartree

TCG = 2.302779 Hartree

H = -10259.281587 Hartree

G = -10259.794875 Hartree

No Imaginary frequency

Rh	0	14.88064600	10.34251700	20.96964100	H
Rh	0	17.12325600	10.19437600	19.96527300	H
Br	0	18.20396200	2.33354700	25.41544100	L H 41
Br	0	14.53449800	2.78683000	32.05078500	L H 51
Br	0	14.32492700	16.13021700	30.73105300	L H 71
Br	0	12.64486000	9.68903600	34.92528900	L H 61
O	0	17.88913600	10.68203900	21.81433900	H
O	0	15.84508500	10.66259900	22.79043300	H
O	0	16.33786400	12.53067600	26.35261400	H
O	0	17.38417100	8.43384700	24.58664100	H
N	0	17.10116400	10.64003900	25.25170800	H
C	0	17.11087400	10.82891200	22.79682900	H
C	0	17.69847300	11.35540200	24.12191300	H
H	0	17.28339100	12.36791000	24.20737100	H
C	0	19.25963500	11.51036000	24.22066000	H
C	0	19.71144100	12.60531900	23.22787400	H
H	0	19.17661100	13.54520400	23.39951600	H
H	0	19.54769000	12.30446400	22.19292600	H
H	0	20.78169400	12.79693100	23.36416200	H
C	0	19.60450800	12.00147900	25.64425300	H
H	0	19.38147500	11.24633700	26.40508100	H
H	0	19.06208200	12.91655600	25.89859400	H
H	0	20.67686900	12.21532500	25.70216400	H
C	0	20.02833100	10.20270800	23.94821300	H
H	0	19.83374000	9.82510100	22.94360800	H
H	0	19.76488300	9.41717300	24.66119300	H
H	0	21.10384200	10.39235500	24.04247100	H
C	0	16.47316300	11.32432100	26.30513400	H
C	0	17.02948800	9.25093000	25.41521200	H
C	0	16.42484000	9.03148000	26.77427100	H
C	0	16.05495000	10.27805900	27.28901900	H
C	0	15.47476400	10.42400300	28.54866300	H
C	0	15.25102700	9.23263900	29.29251100	H
C	0	15.62384100	7.96619900	28.77269500	H
C	0	16.23883600	7.85022800	27.49323900	H
C	0	16.69762700	6.53102000	26.95896900	H
C	0	18.06991900	6.26680100	26.83821600	H
H	0	18.78866800	7.03558600	27.10734700	H
C	0	18.51714100	5.02666900	26.38563100	H
H	0	19.58452500	4.84042500	26.30785300	H
C	0	17.59529700	4.03702300	26.03373600	H
C	0	16.22790500	4.30023900	26.12994200	H
H	0	15.49443700	3.55537600	25.83935500	H

C	0	15.78174100	5.53698300	26.59530300	H
H	0	14.71607200	5.72609500	26.66968400	H
C	0	15.36617300	6.72594500	29.57565100	H
C	0	14.05394900	6.32280900	29.86274300	H
H	0	13.22300800	6.92673100	29.51374200	H
C	0	13.80792200	5.16030900	30.59266200	H
H	0	12.78176400	4.86867400	30.79839500	H
C	0	14.87175300	4.38343900	31.05510500	H
C	0	16.18278100	4.77945100	30.78309600	H
H	0	17.02234500	4.18796900	31.13720100	H
C	0	16.42688900	5.93884600	30.04694400	H
H	0	17.44897500	6.23225800	29.82939900	H
C	0	14.62496700	9.33239500	30.65050900	H
C	0	13.30613300	9.78737200	30.79249300	H
H	0	12.73775500	10.06027400	29.90860000	H
C	0	12.71928500	9.88815000	32.05384300	H
H	0	11.69415800	10.23788900	32.13721000	H
C	0	13.44627000	9.54536400	33.19560000	H
C	0	14.76240700	9.09686200	33.06622700	H
H	0	15.34198300	8.82733700	33.94464800	H
C	0	15.34472400	8.98808300	31.80364900	H
H	0	16.36539100	8.63012700	31.70961500	H
C	0	15.16309700	11.78017800	29.09673100	H
C	0	14.21143500	12.60370300	28.48032000	H
H	0	13.68462400	12.24476100	27.60198800	H
C	0	13.95382500	13.88302400	28.97078900	H
H	0	13.21415100	14.50545800	28.47517100	H
C	0	14.65441500	14.36403100	30.07930500	H
C	0	15.60464200	13.55157700	30.70087100	H
H	0	16.15905100	13.91118100	31.56306900	H
C	0	15.85295400	12.26763800	30.21552100	H
H	0	16.59318400	11.64087400	30.70282100	H
Br	0	13.26825600	4.43154200	13.19614100	L H 114
Br	0	7.42680900	0.39408500	15.77006300	L H 124
Br	0	11.39487100	2.92165900	28.30459300	L H 144
Br	0	6.06329700	-0.05691700	23.34224400	L H 134
O	0	17.15387200	8.19548000	20.49817000	H
O	0	15.03282800	8.30238600	21.27940100	H
O	0	14.24798600	5.30678500	23.51594000	H
O	0	14.83994600	5.85450800	19.01172500	H
N	0	14.93603400	5.57835000	21.31664900	H
C	0	16.13713800	7.70160300	21.05433400	H
C	0	16.18981400	6.27083600	21.62251500	H
H	0	16.13738300	6.42608500	22.70786600	H
C	0	17.48130800	5.41795500	21.36488800	H
C	0	18.65793800	6.08692400	22.11086600	H
H	0	18.43987400	6.20889600	23.17679900	H
H	0	18.88886000	7.06910900	21.69624200	H
H	0	19.55048300	5.45815300	22.01788700	H
C	0	17.25547500	4.02288400	21.98695300	H
H	0	16.48608800	3.45660400	21.45234700	H
H	0	16.96221600	4.09528000	23.03897000	H
H	0	18.18435100	3.44533200	21.93428800	H
C	0	17.83913100	5.24974300	19.87545200	H
H	0	18.01857200	6.21371200	19.39718400	H
H	0	17.05081200	4.73871200	19.31852800	H
H	0	18.75310800	4.64993900	19.79362900	H
C	0	14.07416100	5.12288800	22.32430100	H
C	0	14.37636600	5.40185900	20.03944300	H
C	0	13.13740600	4.58255700	20.24967600	H
C	0	12.96189800	4.40762100	21.62483800	H
C	0	11.90479800	3.66732000	22.15247100	H
C	0	10.99402400	3.09635700	21.22244100	H
C	0	11.18976700	3.24753300	19.82528000	H
C	0	12.28121700	4.00337800	19.31558800	H

C	0	12.53216900	4.11859700	17.84519900	H
C	0	13.08780100	3.04177500	17.14114600	H
H	0	13.34448200	2.12927800	17.67165200	H
C	0	13.31274600	3.13375700	15.76739500	H
H	0	13.75204300	2.29054400	15.24161800	H
C	0	12.96872600	4.29927300	15.07948400	H
C	0	12.40946500	5.37300400	15.77500500	H
H	0	12.12002600	6.28540700	15.26489400	H
C	0	12.20395800	5.28883300	17.15188200	H
H	0	11.78198500	6.13948000	17.67593500	H
C	0	10.26619500	2.56690700	18.86034000	H
C	0	9.45974700	3.30844300	17.98472600	H
H	0	9.49403300	4.39306400	18.00921400	H
C	0	8.62038100	2.66585800	17.07455100	H
H	0	8.01171100	3.26220300	16.40116400	H
C	0	8.57423200	1.27168900	17.02222200	H
C	0	9.37479700	0.52326500	17.88727100	H
H	0	9.35452200	-0.56253500	17.85760600	H
C	0	10.21204300	1.16671100	18.79806200	H
H	0	10.83169400	0.57862100	19.46800900	H
C	0	9.81118300	2.32856800	21.73019700	H
C	0	8.51030900	2.80664200	21.51530400	H
H	0	8.36754300	3.73227400	20.96603600	H
C	0	7.40365000	2.10547800	21.99350200	H
H	0	6.40648500	2.49841100	21.81557100	H
C	0	7.57931000	0.90850700	22.69076900	H
C	0	8.86955600	0.42155900	22.90919500	H
H	0	9.02371900	-0.50816600	23.44958400	H
C	0	9.97523700	1.12846000	22.43665400	H
H	0	10.97550000	0.74767100	22.61822500	H
C	0	11.75952600	3.47746500	23.62889800	H
C	0	10.69322600	4.05109500	24.33305300	H
H	0	9.95291200	4.63786500	23.79849900	H
C	0	10.58010000	3.88026000	25.71350700	H
H	0	9.75037800	4.34330300	26.23947400	H
C	0	11.52857900	3.12772500	26.40862000	H
C	0	12.58697100	2.53874800	25.71262100	H
H	0	13.32804900	1.94291800	26.23831000	H
C	0	12.70262100	2.71688500	24.33470600	H
H	0	13.53328600	2.26688900	23.79881100	H
Br	0	14.33341300	18.35988400	15.42224700	L H 187
Br	0	6.76447200	18.41795500	13.98663400	L H 197
Br	0	6.08256200	5.62474300	17.31552900	L H 217
Br	0	2.76909800	11.82733700	14.36010600	L H 207
O	0	16.20700200	9.72985800	18.16444200	H
O	0	14.14718000	10.06210200	19.04264000	H
O	0	11.54565600	8.40795900	16.61645400	H
O	0	13.97598300	12.29063800	16.67478300	H
N	0	13.06714600	10.15774900	16.56200600	H
C	0	14.94962900	9.76789300	18.09332500	H
C	0	14.25918200	9.33461100	16.78421800	H
H	0	13.84337400	8.34842700	17.02796300	H
C	0	15.15899200	9.12549700	15.51356700	H
C	0	16.06773900	7.89948400	15.76050800	H
H	0	15.47941800	7.01027000	16.01241600	H
H	0	16.77603100	8.08040800	16.56953200	H
H	0	16.63550200	7.67833600	14.85001000	H
C	0	14.23811900	8.79592200	14.31877500	H
H	0	13.59784100	9.64080500	14.04611000	H
H	0	13.59799700	7.93382600	14.52880400	H
H	0	14.85106400	8.55222100	13.44496100	H
C	0	16.02191900	10.35036300	15.15245800	H
H	0	16.70118700	10.61331300	15.96461900	H
H	0	15.41225100	11.22767700	14.92466800	H
H	0	16.62229300	10.11670500	14.26570300	H

C	0	11.78262900	9.59714600	16.51635700	H
C	0	13.02180600	11.55947300	16.49088000	H
C	0	11.59189500	11.90672800	16.18331500	H
C	0	10.83868800	10.73302700	16.28907100	H
C	0	9.45517100	10.70935000	16.11706700	H
C	0	8.83667800	11.93618200	15.74440000	H
C	0	9.59982200	13.12614700	15.62307700	H
C	0	11.00430200	13.13239800	15.86404500	H
C	0	11.80911100	14.38664500	15.76187100	H
C	0	12.82998500	14.50234700	14.80729500	H
H	0	13.04124500	13.66891200	14.14388100	H
C	0	13.57103900	15.67808300	14.70072300	H
H	0	14.35296300	15.74856500	13.94985600	H
C	0	13.30966500	16.75113100	15.55746100	H
C	0	12.30270300	16.64145200	16.51789400	H
H	0	12.09614400	17.45507400	17.20574700	H
C	0	11.55308200	15.46989000	16.61297000	H
H	0	10.76538900	15.39975000	17.35575500	H
C	0	8.91784600	14.40342700	15.23317100	H
C	0	7.98101400	15.00470600	16.08583800	H
H	0	7.76251000	14.55455700	17.04879200	H
C	0	7.34534600	16.18948800	15.71422500	H
H	0	6.62344900	16.63838000	16.39093000	H
C	0	7.63460800	16.79134000	14.48814200	H
C	0	8.56889400	16.20137500	13.63440700	H
H	0	8.80864400	16.65793100	12.67823200	H
C	0	9.20702500	15.01835200	14.00647200	H
H	0	9.93968300	14.56940200	13.34249400	H
C	0	7.37007600	11.94924600	15.43038700	H
C	0	6.41112200	11.65390700	16.40992600	H
H	0	6.73972900	11.43275100	17.42086700	H
C	0	5.05156700	11.61952300	16.09694800	H
H	0	4.32144500	11.36414900	16.85975000	H
C	0	4.63031300	11.88431600	14.79201000	H
C	0	5.57380400	12.18249800	13.80707400	H
H	0	5.26135800	12.38869900	12.78718000	H
C	0	6.93176100	12.21343600	14.12420800	H
H	0	7.66004300	12.44420500	13.35310700	H
C	0	8.67293300	9.45886700	16.36251300	H
C	0	8.66618300	8.89923800	17.64927500	H
H	0	9.26220200	9.35913500	18.43233000	H
C	0	7.90690800	7.76433800	17.93036500	H
H	0	7.91546800	7.35180600	18.93581000	H
C	0	7.14861100	7.16248000	16.92314800	H
C	0	7.16743500	7.69737200	15.63336900	H
H	0	6.58982500	7.23643300	14.83737300	H
C	0	7.92125800	8.83811000	15.35615200	H
H	0	7.91666100	9.25653200	14.35503700	H
Br	0	20.23088900	15.69336400	27.27820400	L H 260
Br	0	14.53906200	20.18073500	29.96431800	L H 270
Br	0	8.43912100	18.86801000	18.10471800	L H 290
Br	0	8.72674900	22.02156200	25.40073100	L H 280
O	0	16.97776800	12.20543300	19.50061100	H
O	0	15.01170400	12.39936100	20.60308900	H
O	0	13.28915000	15.70497400	19.52778000	H
O	0	16.85489600	14.63313700	22.18636500	H
N	0	15.28568000	15.11899900	20.54560700	H
C	0	15.97394200	12.85527400	19.89306100	H
C	0	15.78626400	14.31250400	19.42986500	H
H	0	14.92621700	14.25722000	18.74943500	H
C	0	16.94043500	14.98793000	18.61009300	H
C	0	17.08038200	14.24453500	17.26233600	H
H	0	16.12899600	14.21796800	16.72053500	H
H	0	17.42204100	13.21839100	17.40231000	H
H	0	17.81144000	14.76641900	16.63495500	H

C	0	16.51474000	16.44094400	18.30444200	H
H	0	16.46512400	17.05287700	19.21081600	H
H	0	15.53852400	16.47953400	17.81082200	H
H	0	17.24975800	16.90264300	17.63696300	H
C	0	18.29602300	15.00374500	19.34197200	H
H	0	18.63904100	13.99309100	19.56864700	H
H	0	18.24849600	15.56161700	20.28022800	H
H	0	19.04420100	15.48541300	18.70185200	H
C	0	14.04742800	15.77472000	20.47718300	H
C	0	15.86521900	15.23587100	21.81971800	H
C	0	14.99982500	16.20694200	22.57158800	H
C	0	13.89407000	16.50861100	21.77056700	H
C	0	12.90074100	17.40040700	22.17334500	H
C	0	13.04222000	17.97551900	23.46447300	H
C	0	14.15379000	17.65717500	24.28712100	H
C	0	15.17323400	16.77191200	23.83523200	H
C	0	16.38637900	16.48554900	24.66011900	H
C	0	17.64100700	16.95440000	24.24255000	H
H	0	17.72441800	17.50198500	23.30796100	H
C	0	18.77674800	16.72726300	25.01781500	H
H	0	19.73810400	17.10230400	24.67813100	H
C	0	18.67379200	16.01804900	26.21786800	H
C	0	17.43180000	15.53466800	26.63245600	H
H	0	17.33291200	14.95895900	27.54677000	H
C	0	16.29494600	15.77019000	25.85949700	H
H	0	15.33816400	15.37993800	26.18859300	H
C	0	14.25612400	18.26235900	25.65486900	H
C	0	13.31265900	17.94648300	26.64324500	H
H	0	12.50525200	17.25928000	26.40964900	H
C	0	13.39591600	18.51059800	27.91594200	H
H	0	12.65043500	18.25179300	28.66272400	H
C	0	14.42546100	19.40290500	28.22170200	H
C	0	15.37162600	19.72330900	27.24622600	H
H	0	16.17997000	20.41424700	27.46810600	H
C	0	15.28884000	19.15517100	25.97512100	H
H	0	16.03388500	19.40221200	25.22547400	H
C	0	12.00674000	18.94952400	23.93922500	H
C	0	10.68627200	18.53479700	24.16408600	H
H	0	10.41781300	17.49609600	23.99668400	H
C	0	9.71843000	19.43962700	24.60013900	H
H	0	8.70376100	19.09216000	24.77352000	H
C	0	10.05322100	20.77904500	24.80805100	H
C	0	11.36320900	21.20523800	24.57958900	H
H	0	11.63974300	22.24430500	24.73440300	H
C	0	12.33134100	20.29703000	24.15213800	H
H	0	13.34979800	20.63367300	23.98492600	H
C	0	11.78599900	17.77081900	21.24733000	H
C	0	10.80679100	16.83570100	20.89006100	H
H	0	10.83998000	15.83902400	21.31984100	H
C	0	9.80813300	17.16041700	19.97223800	H
H	0	9.07785300	16.40521900	19.69786300	H
C	0	9.77917100	18.43328800	19.39636200	H
C	0	10.74240400	19.37892000	19.75525400	H
H	0	10.73028000	20.37319700	19.31750000	H
C	0	11.73715700	19.05011500	20.67693800	H
H	0	12.48847400	19.78593700	20.94706500	H
C	0	12.97457800	10.26872300	21.65591200	H
C	0	11.83070400	11.01295100	21.20236600	H
C	0	12.68854900	9.09944700	22.52962300	H
C	0	12.01554100	12.20370200	20.44140500	H
C	0	10.50375900	10.58633000	21.51221800	H
O	0	12.26516700	8.04776800	22.10033000	H
O	0	12.98565200	9.35473600	23.82767700	H
C	0	10.93502900	12.97218200	20.02202500	H
H	0	13.02569900	12.51708800	20.21546400	H

C	0	9.43833200	11.34163200	21.06635000	H
H	0	10.34444800	9.67041400	22.06939700	H
C	0	12.87258200	8.27823600	24.75724300	H
C	0	9.65475300	12.52012700	20.33754700	H
H	0	11.09188100	13.89012600	19.46559100	H
C	0	7.95453600	11.11766500	21.22838700	H
C	0	11.68168900	8.49592000	25.70494700	H
H	0	12.76127200	7.32071700	24.24536300	H
H	0	13.78498400	8.27626000	25.35592200	H
C	0	8.33481800	13.12923600	20.02772800	H
N	0	7.38842400	12.24756500	20.48845300	H
H	0	7.63548800	11.14008100	22.27887300	H
H	0	7.63230900	10.16237000	20.79807500	H
Cl	0	10.11332200	8.42478300	24.81544200	H
Cl	0	11.72358800	7.17641400	26.92406800	H
Cl	0	11.80135200	10.08723300	26.53630300	H
O	0	8.09186600	14.21522900	19.49366800	H
C	0	5.95933200	12.56314700	20.42726900	H
C	0	5.57730100	13.37898800	21.68984400	H
C	0	5.09951300	11.30949100	20.26374800	H
H	0	5.84720500	13.20734600	19.54894100	H
N	0	5.95840500	14.68253200	21.62662100	H
O	0	5.00583800	12.87263000	22.64645700	H
C	0	3.62771200	11.65953300	19.96760200	H
H	0	5.14019300	10.70439000	21.17287500	H
H	0	5.51379300	10.71727900	19.43956600	H
H	0	5.85480900	15.24643800	22.45779200	H
H	0	6.56378100	15.00098200	20.87856200	H
C	0	2.86647400	10.45235200	19.45089100	H
H	0	3.55676900	12.42975100	19.19488900	H
H	0	3.15769700	12.03071300	20.88278700	H
O	0	2.45666100	10.35060000	18.30975300	H
O	0	2.74774200	9.51899100	20.41071300	H
C	0	2.10833200	8.21049000	20.15355600	H
C	0	0.63792100	8.41629300	19.77673900	H
C	0	2.23222400	7.50860900	21.50765100	H
C	0	2.88780000	7.45523300	19.07205900	H
H	0	0.14002600	7.44381600	19.70573600	H
H	0	0.54451500	8.92872300	18.81897600	H
H	0	0.12653900	9.00325200	20.54601100	H
H	0	1.78451300	6.51170600	21.45583400	H
H	0	3.28260800	7.40225100	21.79404200	H
H	0	1.71978000	8.07949700	22.28710400	H
H	0	3.94533400	7.38211700	19.34521900	H
H	0	2.80716800	7.95276400	18.10536200	H
H	0	2.49127500	6.43903200	18.97900500	H

Structure A-SI-2 (Frequency calculation was limited due to the physical limitation of our facility)

E = -51396.217145 Hartree

Rh	15.09725200	10.03737200	20.90350600
Rh	17.31015400	9.97665200	19.82163600
Br	17.23291500	2.07032200	25.55601600
Br	14.11498100	3.00626400	31.85702400
Br	14.62035400	16.26513100	30.41712200
Br	12.42185100	10.16452800	34.53223900
O	18.06578600	10.63761600	21.62788500
O	16.05071800	10.51565600	22.65898600
O	16.56749500	12.52352500	26.11334000
O	17.33453100	8.35804600	24.38498400
N	17.22586800	10.57698300	25.05730600
C	17.30413100	10.74012900	22.62945800
C	17.87932600	11.26208200	23.94715500
H	17.50078000	12.29000600	24.01410400
C	19.42988700	11.34635800	24.08848200
C	19.95230000	12.42122500	23.11420900
H	19.46009100	13.38319000	23.29670500

H	19.78392900	12.13950800	22.07475600
H	21.02713300	12.56064600	23.26720400
C	19.75716000	11.81033900	25.52127800
H	19.46653400	11.06372800	26.26621300
H	19.25749400	12.75130200	25.76535600
H	20.83603000	11.96692100	25.61315600
C	20.11892700	9.99713800	23.82726800
H	19.91871700	9.63644300	22.81717100
H	19.78326600	9.23476600	24.53485100
H	21.20134500	10.11190400	23.94600000
C	16.61926400	11.30999700	26.08478600
C	17.02753400	9.19989700	25.21013200
C	16.36596000	9.03386600	26.55091200
C	16.09851400	10.31180000	27.05392300
C	15.49161500	10.52396400	28.28466200
C	15.15571500	9.37210500	29.03883200
C	15.43453000	8.07594900	28.55074300
C	16.04057100	7.88594700	27.27361600
C	16.31729900	6.51482400	26.76577500
C	17.62582000	6.12226500	26.45419100
H	18.43468100	6.84106500	26.52027700
C	17.90807900	4.80710500	26.09119400
H	18.92361300	4.50368500	25.86745100
C	16.86279800	3.88622000	26.02800700
C	15.55027800	4.25875800	26.29835400
H	14.74502700	3.54153300	26.21859000
C	15.28558800	5.57217500	26.67052700
H	14.26633200	5.85581100	26.90246700
C	15.11569000	6.88339800	29.39086000
C	13.80881900	6.62997300	29.82750700
H	13.01836300	7.33199600	29.59721300
C	13.50367100	5.48378000	30.55708700
H	12.48559500	5.28845300	30.87102400
C	14.52649800	4.59255200	30.87167800
C	15.84265500	4.83440200	30.48298200
H	16.63100200	4.13941600	30.74610800
C	16.12749700	5.97873400	29.74228000
H	17.14718500	6.16130500	29.42258000
C	14.50388800	9.55353200	30.36649700
C	13.23993900	10.15167400	30.44752000
H	12.73791000	10.47142700	29.54086500
C	12.61376100	10.33232800	31.67889200
H	11.63282200	10.78863600	31.73649200
C	13.26985700	9.91673100	32.83571200
C	14.53017800	9.32430600	32.78393200
H	15.02750500	9.00957900	33.69353000
C	15.13948100	9.14278600	31.54439900
H	16.11712100	8.67589600	31.49350600
C	15.25918200	11.89957600	28.80632800
C	14.38685100	12.78072100	28.15663700
H	13.88094700	12.47158900	27.25006600
C	14.17147900	14.06599300	28.64712000
H	13.48705400	14.74210200	28.14830000
C	14.86527200	14.48017500	29.78281600
C	15.74595500	13.62724800	30.44512700
H	16.28057800	13.96426100	31.32509200
C	15.92932400	12.33505500	29.95643900
H	16.60730900	11.66260800	30.47049000
Br	14.94602200	3.52779200	13.47026100
Br	7.27679600	3.12475600	15.10547800
Br	10.78170100	4.20969400	28.00639800
Br	5.07317600	3.54628700	22.39556600
O	17.53033000	8.05869800	20.55013800
O	15.40102500	8.04503900	21.32439000
O	14.54829500	5.43082600	23.54604300

O	15.67504200	5.65119400	19.13170000
N	15.51184900	5.39791000	21.43689100
C	16.56084000	7.53317000	21.15662000
C	16.70161000	6.15733700	21.81612400
H	16.55785200	6.36083300	22.88300500
C	18.04564700	5.38950800	21.69075400
C	19.11818100	6.19314800	22.45295900
H	18.82183300	6.35777900	23.49286500
H	19.28871700	7.16709700	21.99041700
H	20.06249800	5.63944400	22.44826900
C	17.85314500	4.03465400	22.39422400
H	17.14868800	3.39789400	21.85154900
H	17.47532700	4.16670400	23.40989300
H	18.80957300	3.50657300	22.45567500
C	18.50746500	5.14481800	20.24562300
H	18.65005200	6.08379500	19.70903300
H	17.78825800	4.54041900	19.69005600
H	19.46172200	4.60714200	20.26467400
C	14.46340700	5.20146900	22.35453800
C	15.05222900	5.29369000	20.11498700
C	13.65730300	4.78498100	20.20519400
C	13.29281900	4.74583300	21.55066300
C	11.99340800	4.45150900	21.95118200
C	11.03912700	4.26045600	20.92154500
C	11.41499400	4.28455300	19.55727400
C	12.76092700	4.50863400	19.17824200
C	13.25979600	4.31967300	17.78635700
C	14.06497600	3.20079100	17.53018900
H	14.29868800	2.51404400	18.33736100
C	14.56820500	2.95311300	16.25452900
H	15.18977700	2.08607600	16.06542600
C	14.25775400	3.84112300	15.22766000
C	13.46207500	4.96051400	15.45576600
H	13.23361200	5.64148100	14.64543600
C	12.97033200	5.19997300	16.73852400
H	12.38360800	6.09065100	16.91604400
C	10.39786900	4.01238300	18.50140500
C	9.97505900	5.03114700	17.63899800
H	10.34594200	6.04221100	17.76097700
C	9.06216200	4.76672700	16.62058100
H	8.74687100	5.55752500	15.95354600
C	8.55224000	3.47930100	16.48626000
C	8.94073200	2.45072300	17.34194200
H	8.53124500	1.45420400	17.22690400
C	9.86821200	2.72536300	18.34575300
H	10.18334800	1.93074300	19.01386600
C	9.60759700	4.05525700	21.27849900
C	8.66768100	5.02747700	20.91747600
H	8.99841300	5.90215400	20.37032800
C	7.32511000	4.88674400	21.25515500
H	6.59879800	5.63868100	20.97591300
C	6.92297400	3.74976900	21.95148300
C	7.83557300	2.76456200	22.32345500
H	7.50576100	1.88583400	22.86471300
C	9.17968800	2.92767400	21.98874600
H	9.89905200	2.17065500	22.28208300
C	11.64767900	4.37178000	23.39334000
C	10.68999500	5.22014100	23.96339500
H	10.17475300	5.94468400	23.34430100
C	10.40739100	5.15970300	25.32649700
H	9.68046100	5.83090200	25.76550700
C	11.09403500	4.24253800	26.11945700
C	12.03115900	3.36892300	25.57348400
H	12.53956700	2.64720600	26.20175500
C	12.30658200	3.44397800	24.21016500

H	13.04702600	2.77906800	23.77857100
Br	13.96226200	18.21085800	15.75402100
Br	6.71175800	18.03308800	14.35970200
Br	5.79225900	5.60040200	17.98489400
Br	2.64200000	11.39833500	15.70900400
O	16.31155100	9.35307600	18.08051900
O	14.29526800	9.62052700	19.07301400
O	11.51752100	8.11152700	17.00296900
O	13.97512100	11.96220700	16.98833500
N	13.04525200	9.84747200	16.78036200
C	15.05241700	9.37920200	18.07715500
C	14.25582600	9.02634200	16.81457500
H	13.87747700	8.01695900	17.01344200
C	15.01917700	8.95542300	15.45771400
C	15.97714000	7.74788300	15.51522900
H	15.43975900	6.82468500	15.75309900
H	16.75563400	7.89621600	16.26463300
H	16.45559700	7.61422600	14.54015200
C	13.97978700	8.71175800	14.34697100
H	13.33913600	9.58502600	14.19533000
H	13.33463700	7.85904300	14.57842300
H	14.49134100	8.50261800	13.40313400
C	15.81420700	10.23128900	15.13131700
H	16.53881600	10.45846100	15.91501800
H	15.16064700	11.09685100	15.00941800
H	16.35686300	10.08335600	14.19187800
C	11.75999900	9.29928800	16.87780700
C	13.00364400	11.25154700	16.80319200
C	11.56175700	11.61842400	16.61156400
C	10.81455500	10.44337600	16.76352900
C	9.42692200	10.43421500	16.71857700
C	8.78592000	11.65968500	16.40060400
C	9.53429300	12.83596600	16.18373200
C	10.95206900	12.84368100	16.33878400
C	11.71343900	14.11274100	16.21082500
C	12.81614600	14.22291300	15.35307800
H	13.14470900	13.36465800	14.77849400
C	13.48883000	15.43488700	15.21200400
H	14.33706500	15.51906300	14.54342900
C	13.04769200	16.54048200	15.93926100
C	11.96088300	16.45750900	16.80358000
H	11.64188100	17.31514500	17.38156200
C	11.30122800	15.24080300	16.93206800
H	10.46344200	15.16803100	17.61336500
C	8.83154500	14.07596100	15.74312600
C	7.81576900	14.64921200	16.51882500
H	7.54765500	14.20268700	17.46702000
C	7.17630700	15.81423000	16.10566600
H	6.40772100	16.26549000	16.72047300
C	7.55538600	16.40471800	14.90279800
C	8.54815700	15.84652000	14.10105300
H	8.82831300	16.31418300	13.16477600
C	9.18192300	14.68168900	14.52892500
H	9.96522600	14.24774600	13.91692300
C	7.30171600	11.66025000	16.24369000
C	6.47267500	11.37300300	17.33490800
H	6.91637800	11.18382900	18.30458300
C	5.09007500	11.30481700	17.18409900
H	4.44651000	11.07889800	18.02318300
C	4.53636700	11.52232700	15.92445800
C	5.33657500	11.81987200	14.82350700
H	4.89170700	11.98742600	13.84997900
C	6.71829500	11.88960000	14.99185700
H	7.34841500	12.11763900	14.13915400
C	8.61749000	9.21608100	16.99464500

C	8.52496200	8.72994500	18.30385000
H	9.11660000	9.18823700	19.08915400
C	7.66170100	7.68052500	18.61329300
H	7.57257600	7.33204900	19.63310900
C	6.91283000	7.09375900	17.59470100
C	6.99862200	7.55250100	16.28101800
H	6.41542900	7.08222000	15.49839500
C	7.84846200	8.61726800	15.98957400
H	7.90152400	8.99464300	14.97443200
Br	19.69520400	15.37813600	27.37518800
Br	13.70269400	19.72631400	29.65865700
Br	8.42381700	18.68637100	17.78246500
Br	7.94964600	20.89846600	25.07528200
O	16.99257000	11.92289500	19.21393000
O	15.10280200	12.04896800	20.45153000
O	13.10630700	15.14543500	19.28965100
O	16.67083600	14.19829700	21.97523300
N	15.12532900	14.69336000	20.31969300
C	15.98376200	12.52827400	19.66059500
C	15.67793200	13.94911100	19.19515700
H	14.82489700	13.82339600	18.51626700
C	16.76884200	14.72242200	18.39850000
C	16.95699200	14.02490800	17.03634900
H	16.00734000	13.96893000	16.49411000
H	17.34516300	13.01252700	17.15391800
H	17.66202400	14.59859600	16.42644300
C	16.23623400	16.14424500	18.13870600
H	16.15046900	16.71990000	19.06493700
H	15.25490000	16.12183900	17.65838900
H	16.92321700	16.67826900	17.47574400
C	18.10829700	14.81033300	19.14699200
H	18.52123500	13.81885900	19.34121400
H	17.99898000	15.32777000	20.10310800
H	18.82670100	15.37131800	18.54011400
C	13.84502000	15.25060800	20.25087200
C	15.65782500	14.77032900	21.61472500
C	14.71295300	15.64708000	22.38707400
C	13.61725400	15.91893900	21.55781600
C	12.56819200	16.74233900	21.94363800
C	12.62247000	17.26896300	23.25697100
C	13.71767000	16.99350900	24.10616100
C	14.80540000	16.18268200	23.67307200
C	15.98547000	15.95618500	24.54907800
C	17.27997400	16.23619600	24.08799100
H	17.42779400	16.59430100	23.07577700
C	18.38502500	16.06991600	24.91965900
H	19.38320100	16.28769000	24.55906100
C	18.18469800	15.62469300	26.22603000
C	16.91305100	15.34198700	26.71150500
H	16.77060600	14.97111600	27.71791400
C	15.82238700	15.50471600	25.86434700
H	14.83467400	15.26333000	26.23321300
C	13.73879000	17.61709200	25.46145000
C	12.75815900	17.29697400	26.40942100
H	11.99203500	16.56880600	26.16539000
C	12.74660000	17.90925200	27.66086000
H	11.98956800	17.65473000	28.39234700
C	13.72367900	18.85801700	27.95546400
C	14.71595600	19.18855200	27.03566400
H	15.47113300	19.92512300	27.28246000
C	14.71955500	18.56005000	25.79221700
H	15.48989200	18.80963300	25.07055200
C	11.50826100	18.14915800	23.71137700
C	10.20519700	17.64084700	23.78917000
H	10.01687500	16.60347400	23.53397700

C	9.14495700	18.44790400	24.19649100
H	8.14035700	18.04713600	24.26136500
C	9.39765600	19.77945300	24.51884100
C	10.68262600	20.31368900	24.44342100
H	10.86133200	21.35267800	24.69317400
C	11.73265500	19.49180500	24.04060900
H	12.73561700	19.89972000	23.98328700
C	11.52286200	17.18112100	20.97478500
C	10.52167300	16.32612200	20.50406200
H	10.47190400	15.30098500	20.85277900
C	9.58210000	16.77324000	19.57523800
H	8.82114200	16.09583900	19.21165500
C	9.66282100	18.08381400	19.10909100
C	10.64680700	18.95858200	19.56537300
H	10.69751500	19.97471000	19.19290700
C	11.56948400	18.49974500	20.50289500
H	12.34109900	19.17138100	20.86405800
C	13.20975500	9.92016300	21.59163200
C	12.06405000	10.61774000	21.10587000
C	12.97133900	8.78322500	22.50949200
C	12.22805900	11.72192900	20.21633000
C	10.75260100	10.26815400	21.55193400
O	12.50222200	7.72421900	22.14445100
O	13.38741000	9.06983300	23.76090100
C	11.15051700	12.50074300	19.82402000
H	13.22014000	11.96525500	19.87373400
C	9.68766400	11.04044000	21.14301800
H	10.60528300	9.41618600	22.20251900
C	13.15519200	8.10305900	24.78346100
C	9.89286200	12.14339400	20.30039500
H	11.29193600	13.36513900	19.18745100
C	8.22471300	10.93772300	21.48285400
C	12.09445300	8.64477600	25.74757000
H	12.83137800	7.14963700	24.36536200
H	14.09102300	7.97307400	25.32483100
C	8.59287600	12.81839700	20.08835100
N	7.65554700	12.05503800	20.72371000
H	8.03890200	11.06251600	22.55594800
H	7.78730800	9.98449400	21.17079200
Cl	10.49854900	8.78348400	24.92201700
Cl	11.96309100	7.50305000	27.11679200
Cl	12.56478400	10.26432800	26.36673200
O	8.37272000	13.87842300	19.49301900
C	6.26361700	12.48383300	20.83229800
C	6.12680600	13.40811400	22.06628200
C	5.32522600	11.28629500	20.88636000
H	6.06790800	13.07561400	19.93280400
N	6.66529000	14.63572100	21.88621100
O	5.59530100	13.02911800	23.10630700
C	3.85331000	11.69827400	20.75487400
H	5.46807300	10.74055300	21.82082300
H	5.59568800	10.60529800	20.07513400
H	6.71431500	15.26884200	22.67159700
H	7.17531100	14.85706700	21.03966500
C	2.96374300	10.57876400	20.24559900
H	3.72644000	12.53180200	20.05792700
H	3.47419000	12.03867900	21.72405300
O	1.98733400	10.76756600	19.54303100
O	3.41503300	9.38579700	20.65275600
C	2.87760000	8.11508400	20.11758500
C	1.41459300	7.95199400	20.52632200
C	3.75972600	7.07522600	20.80564500
C	3.07290100	8.09362800	18.60092000
H	1.06908900	6.95463900	20.23815900
H	0.78637100	8.69829700	20.04063700

H	1.31090300	8.04921800	21.61106600
H	3.48926900	6.06959100	20.47575300
H	4.81013000	7.24535100	20.55685500
H	3.64336600	7.12894500	21.89158200
H	4.11919100	8.28944100	18.35034800
H	2.44439200	8.83658100	18.10918100
H	2.81748200	7.10313400	18.21568700

Section 4: Spectroscopic Data

^{1}H NMR Spectra

^{1}H NMR Spectrum for Compound 4 (500 MHz).

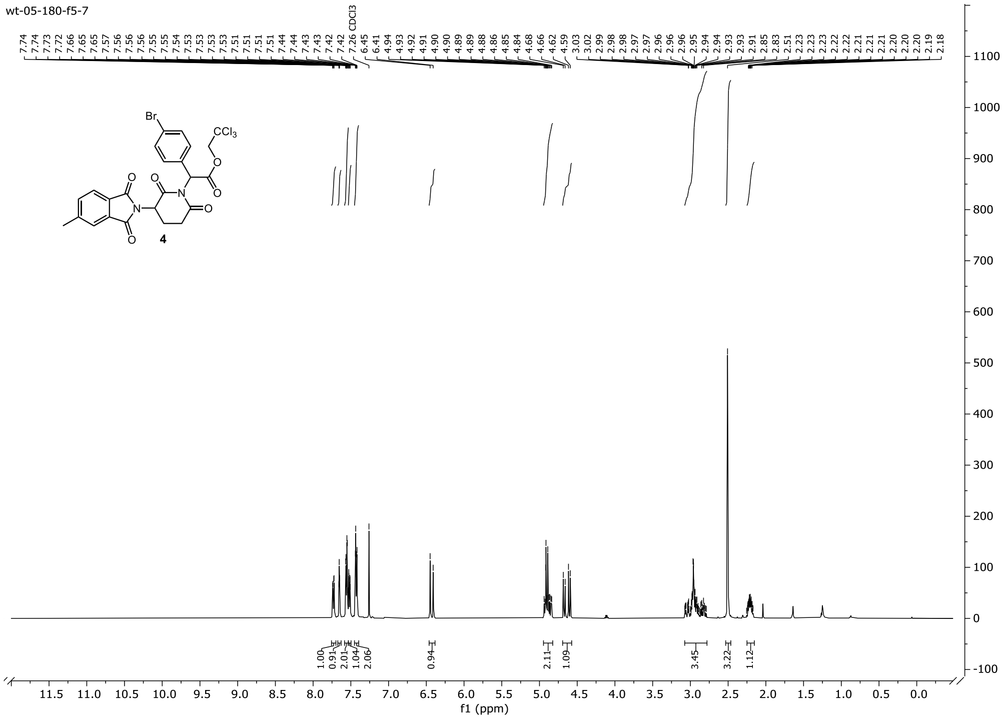

^{1}H NMR Spectrum for Compound 6a (400 MHz).

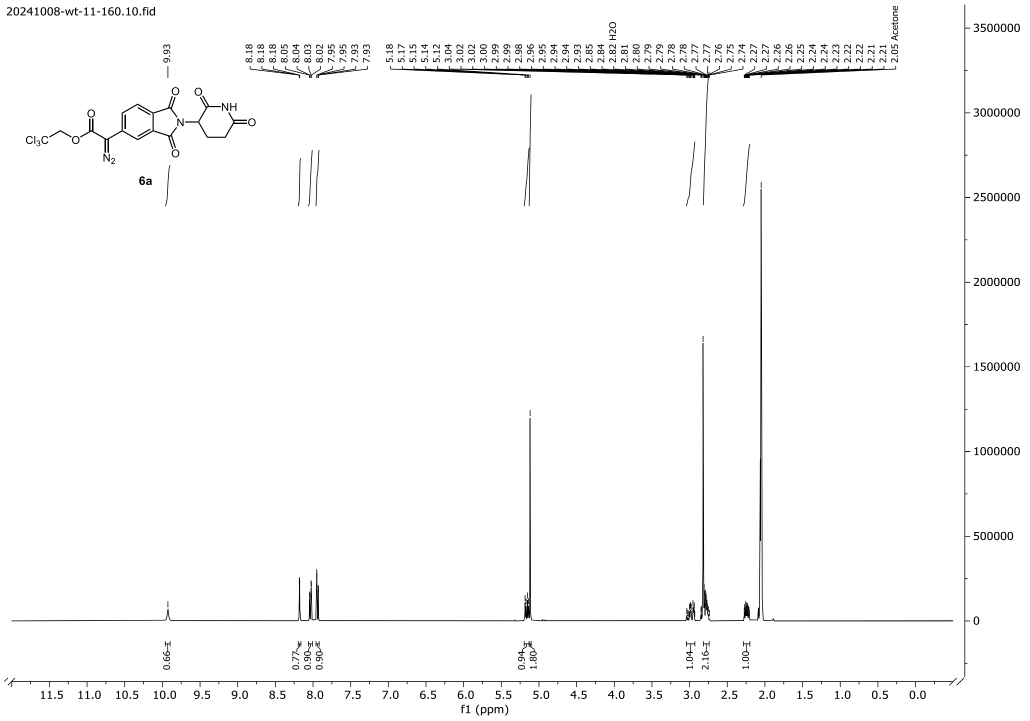

^{1}H NMR Spectrum for Compound 6b (600 MHz).

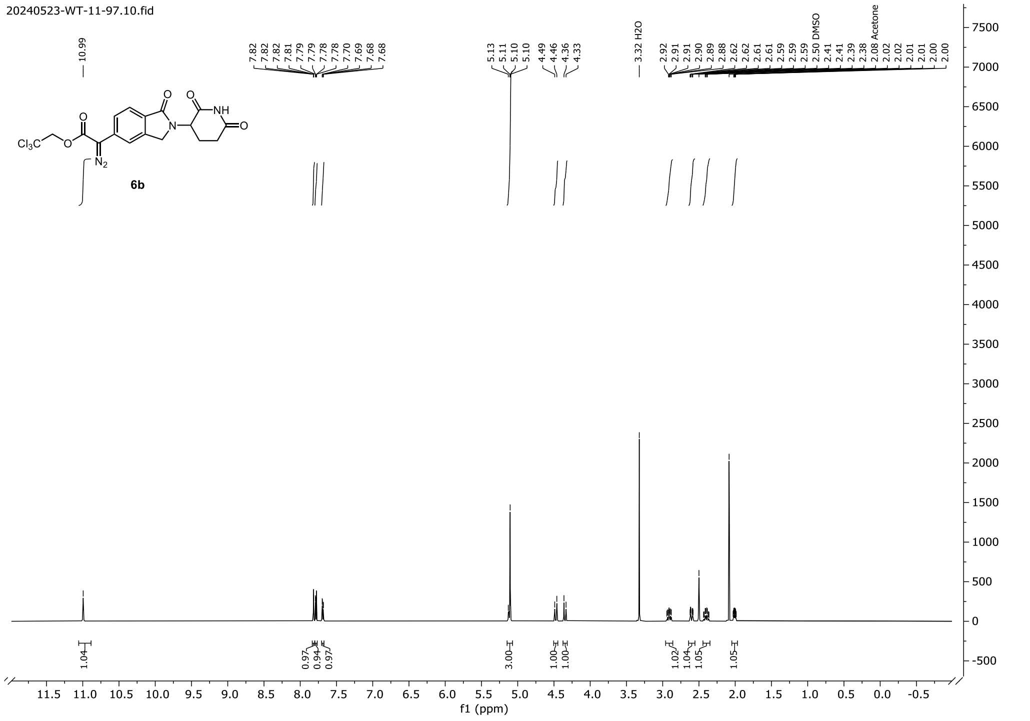

^{1}H NMR Spectrum for Compound 6c (400 MHz).

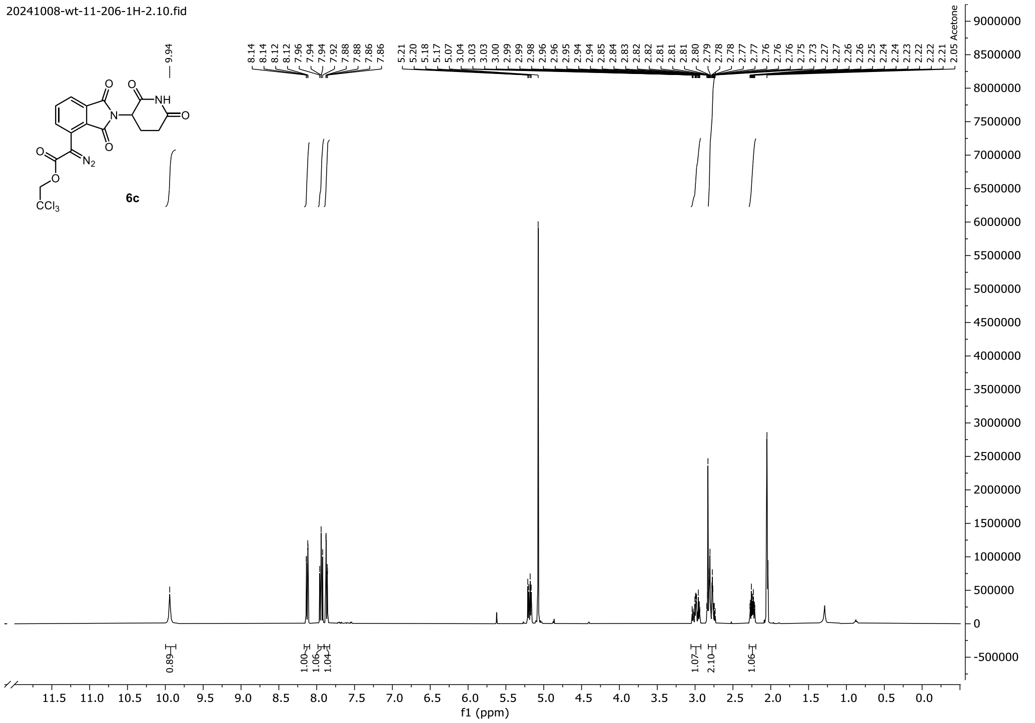

^{1}H NMR Spectrum for Compound 8a (400 MHz).

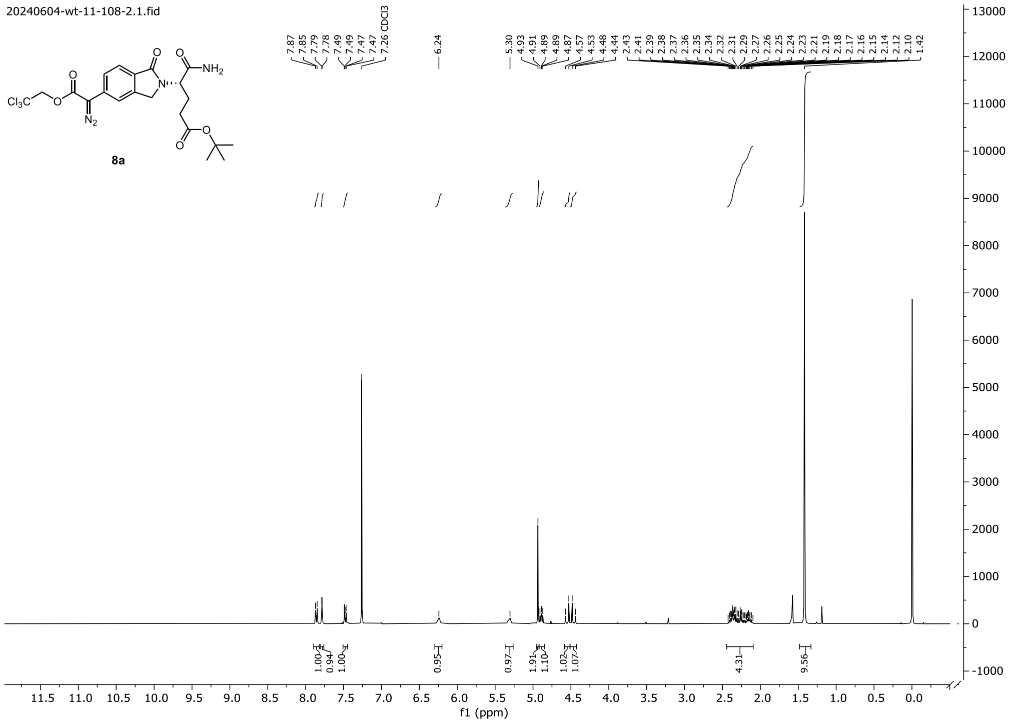

^{1}H NMR Spectrum for Compound 8b (400 MHz).

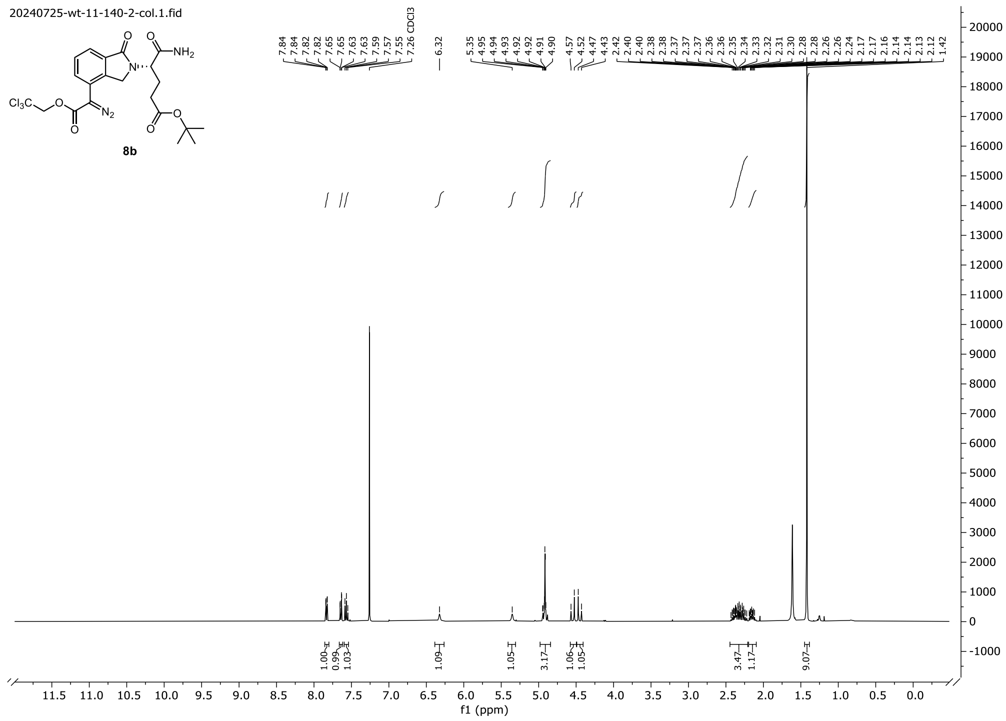

^{1}H NMR Spectrum for Compound 9a (400 MHz).

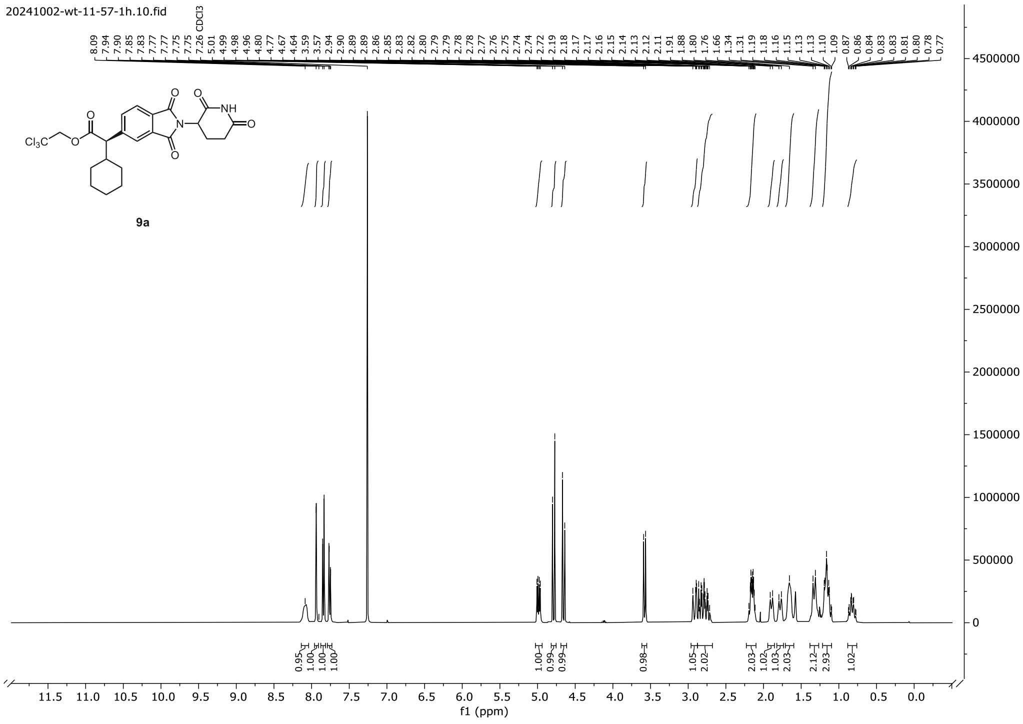

^{1}H NMR Spectrum for Compound 9b (400 MHz).

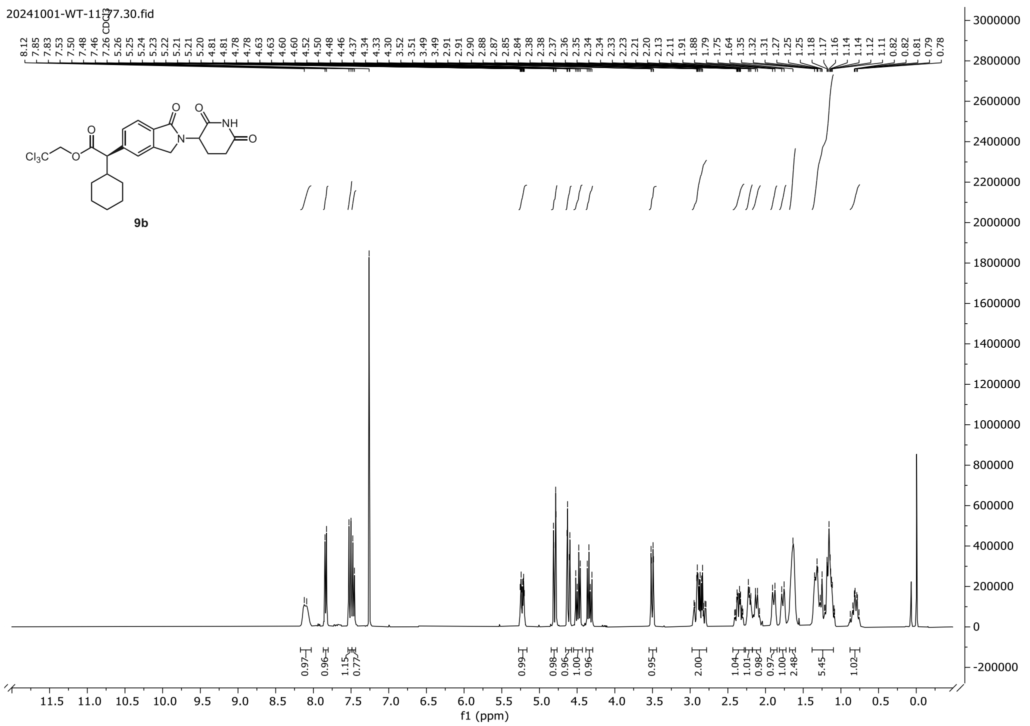

^{1}H NMR Spectrum for Compound 9c (600 MHz).

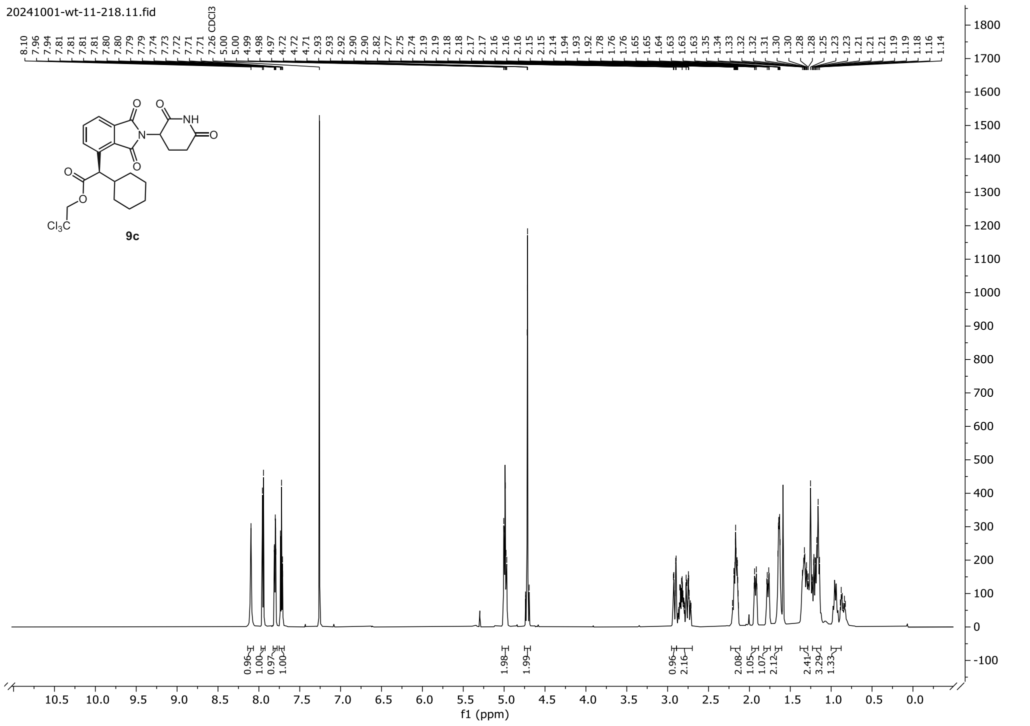

^{1}H NMR Spectrum for Compound (S,R)-10a (600 MHz).

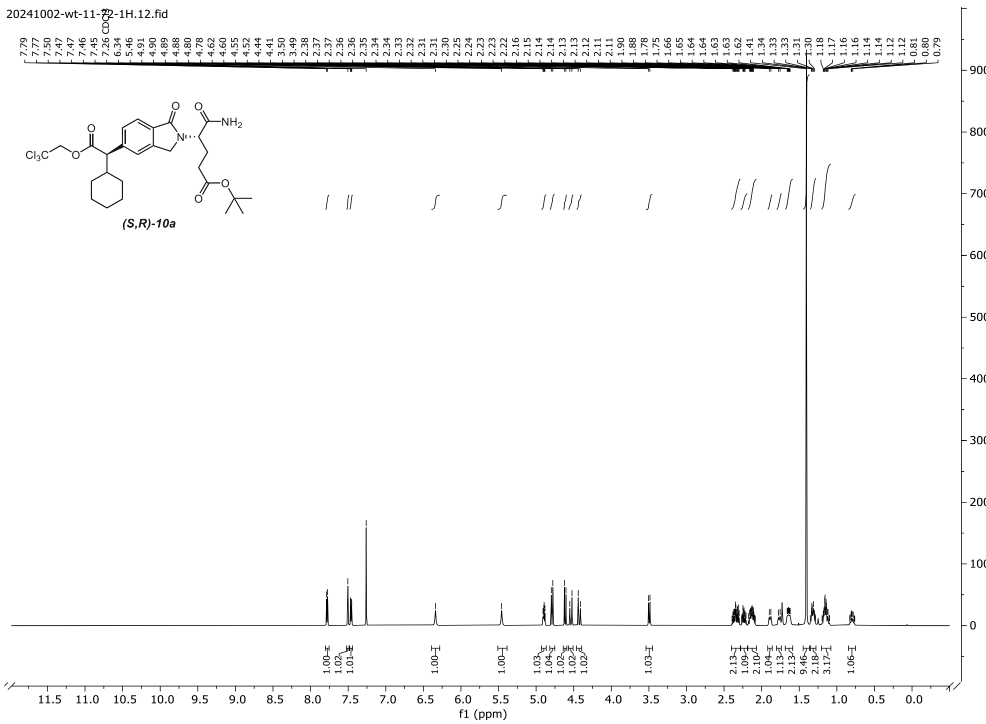

^{1}H NMR Spectrum for Compound (S,S)-10b (400 MHz).

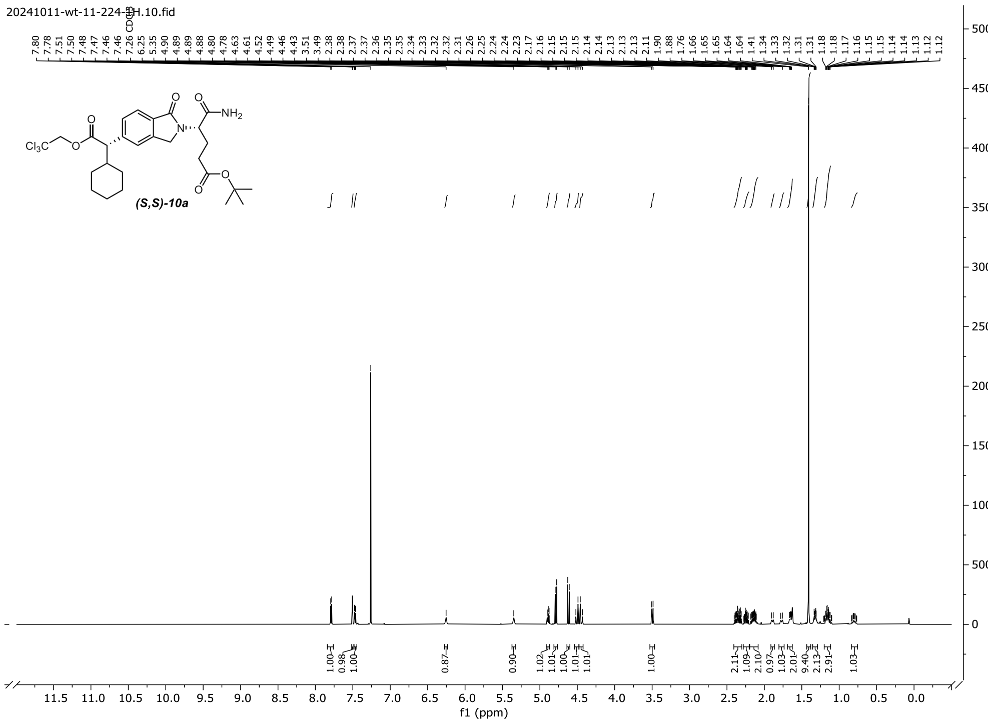

^{1}H NMR Spectrum for Compound 10b (400 MHz).

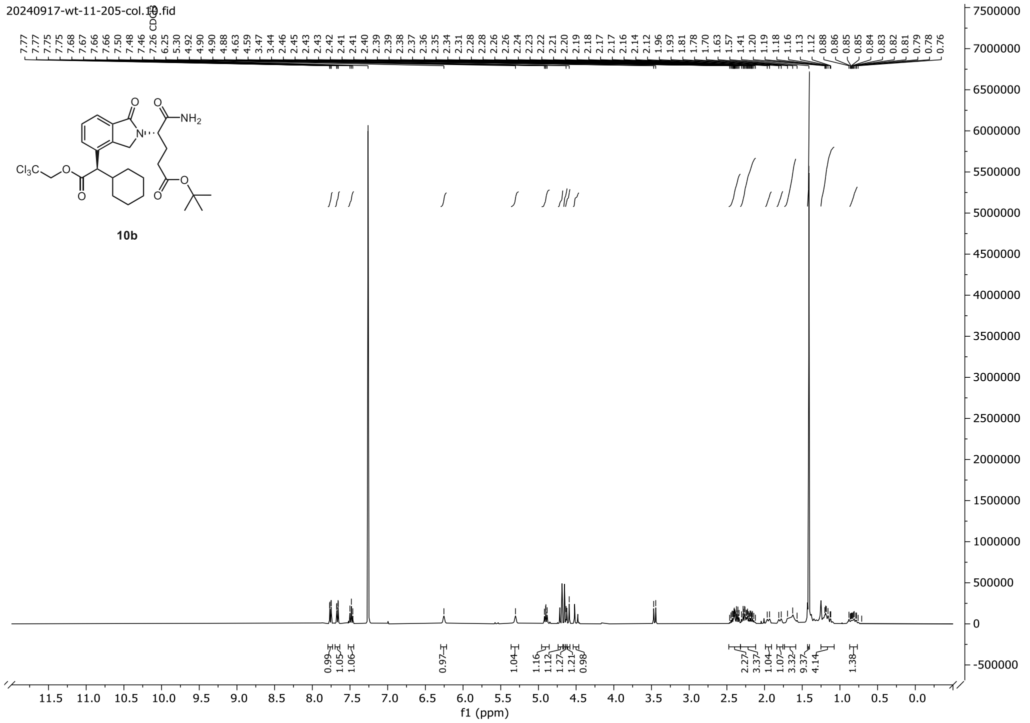

^{1}H NMR Spectrum for Compound 11a (400 MHz).

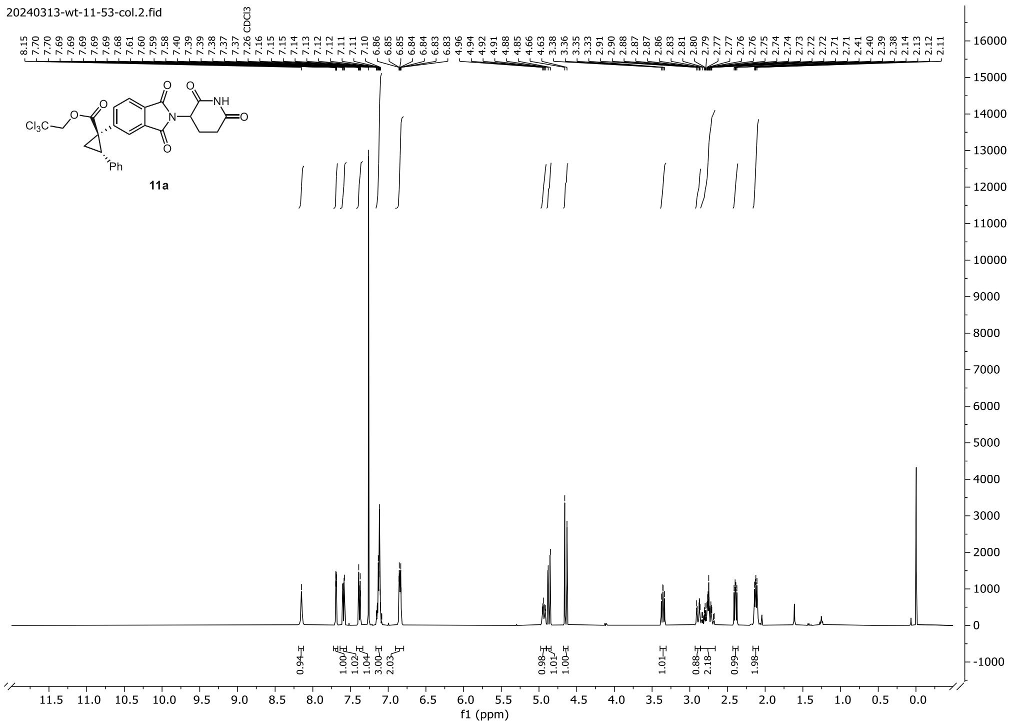

^{1}H NMR Spectrum for Compound 11b (600 MHz).

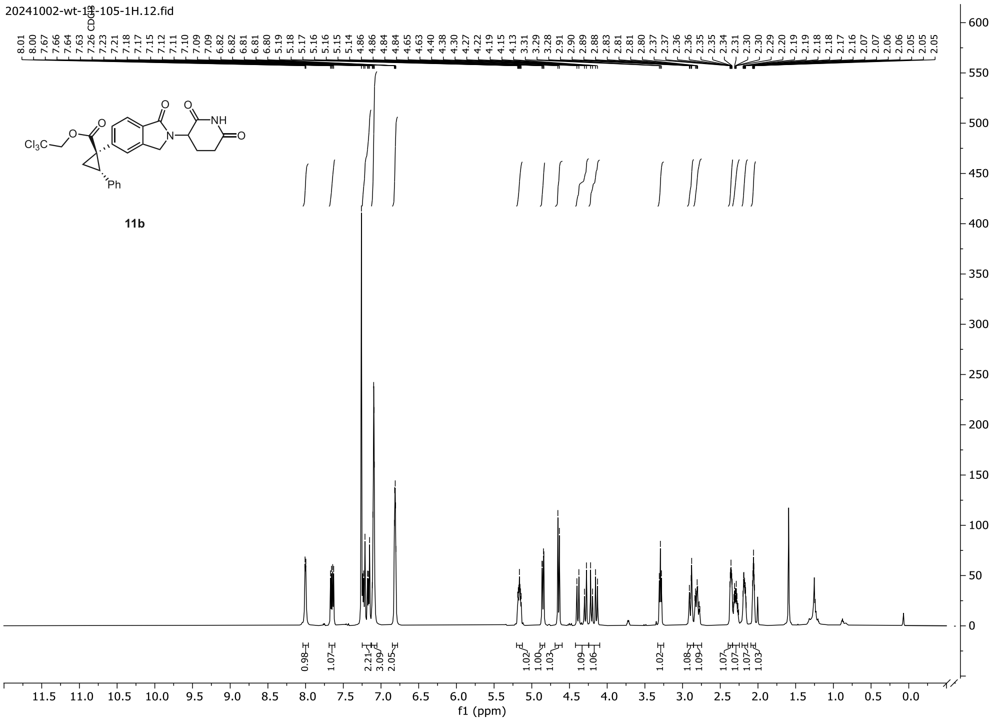

^{1}H NMR Spectrum for Compound 11c (400 MHz).

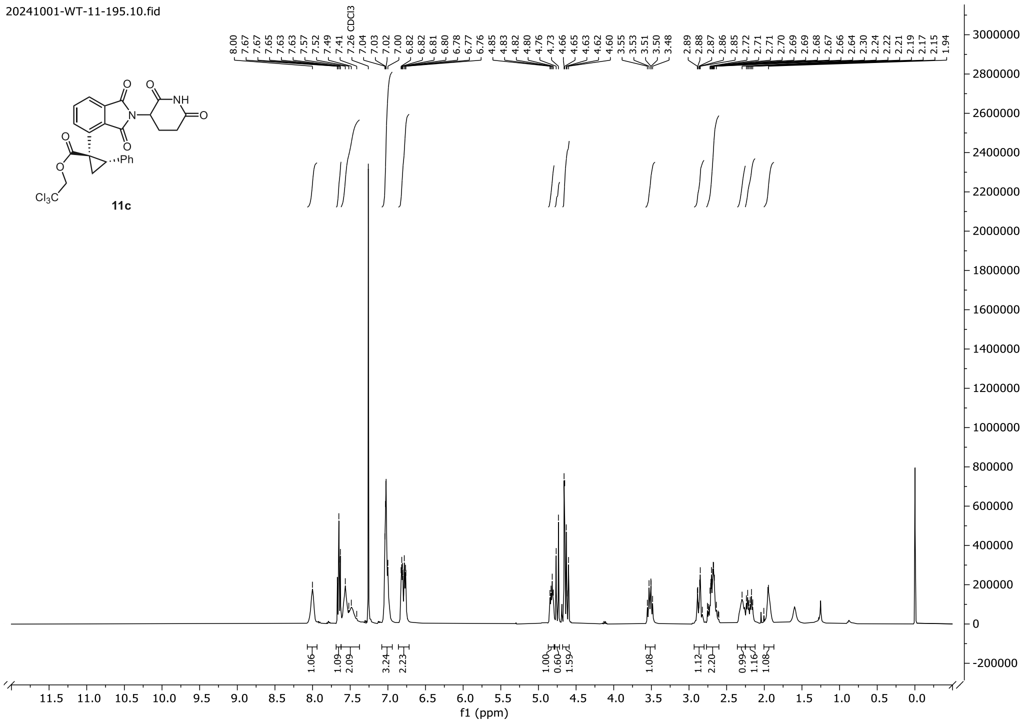

^{1}H NMR Spectrum for Compound 12a (400 MHz).

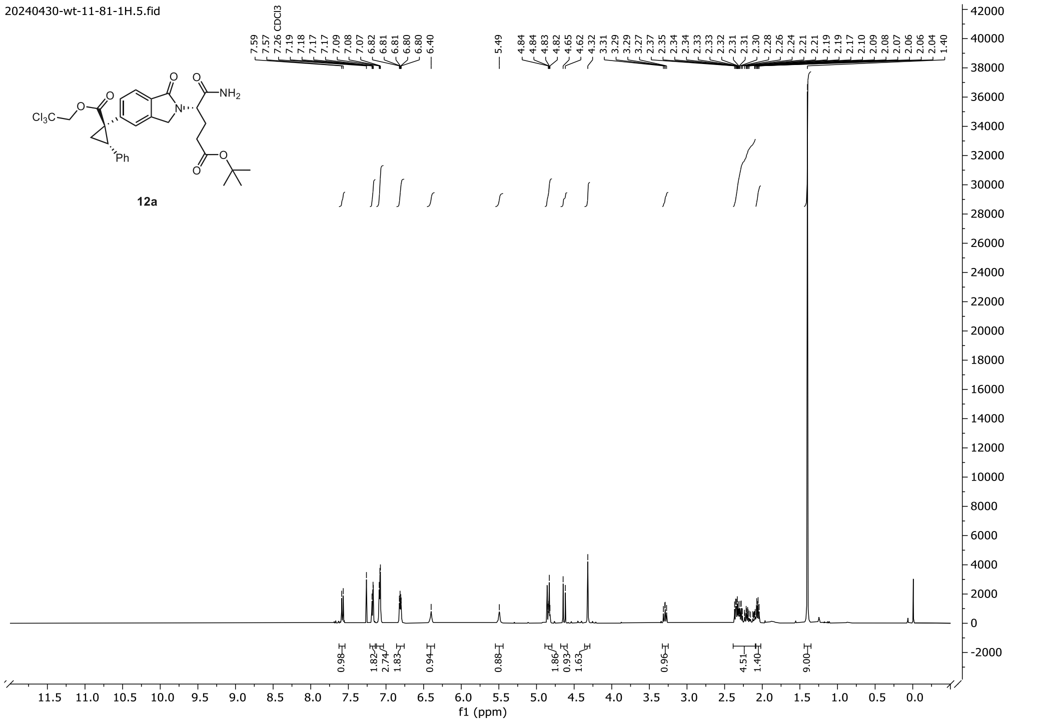

^{1}H NMR Spectrum for Compound 12b (400 MHz).

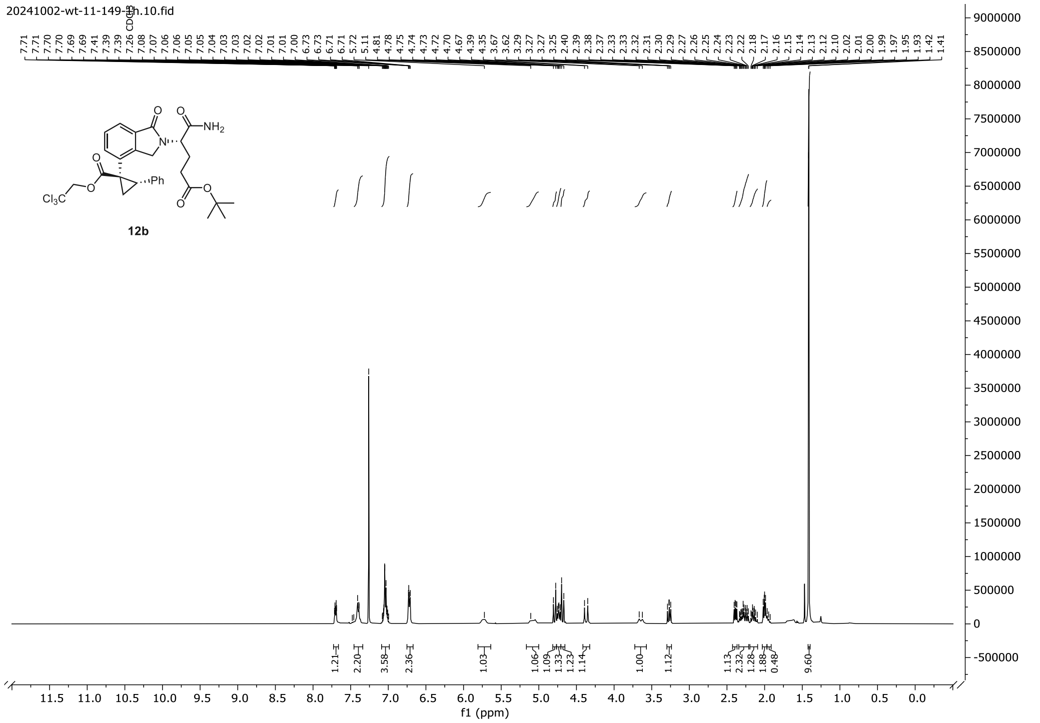

^1H NMR Spectrum for Compound 13 (400 MHz).

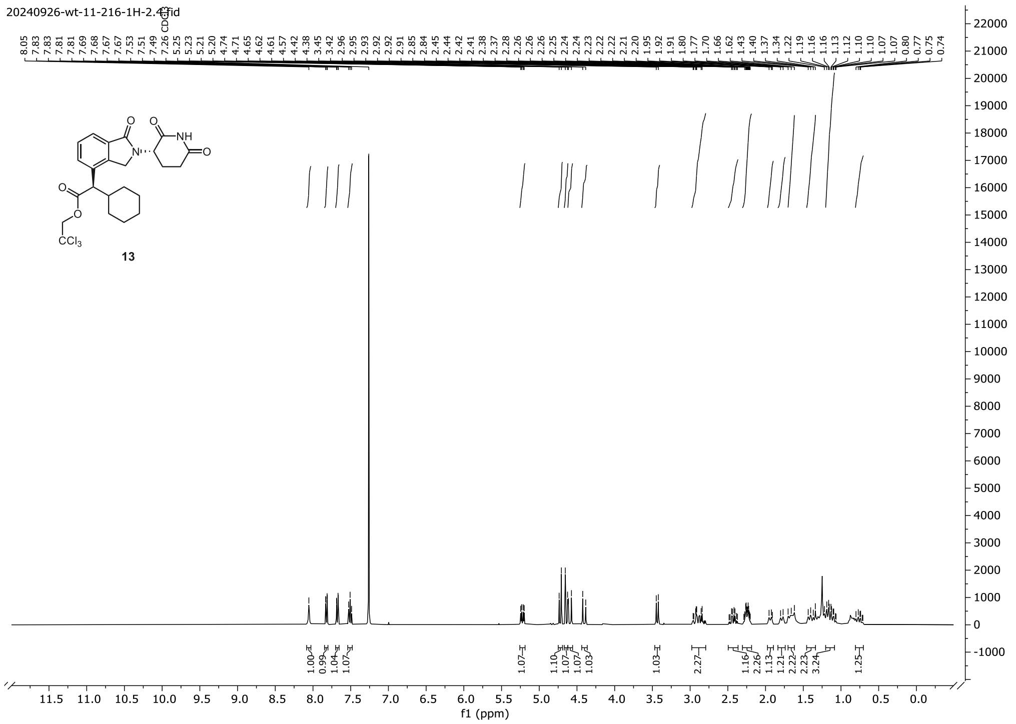

^1H NMR Spectrum for Compound 14 (600 MHz).

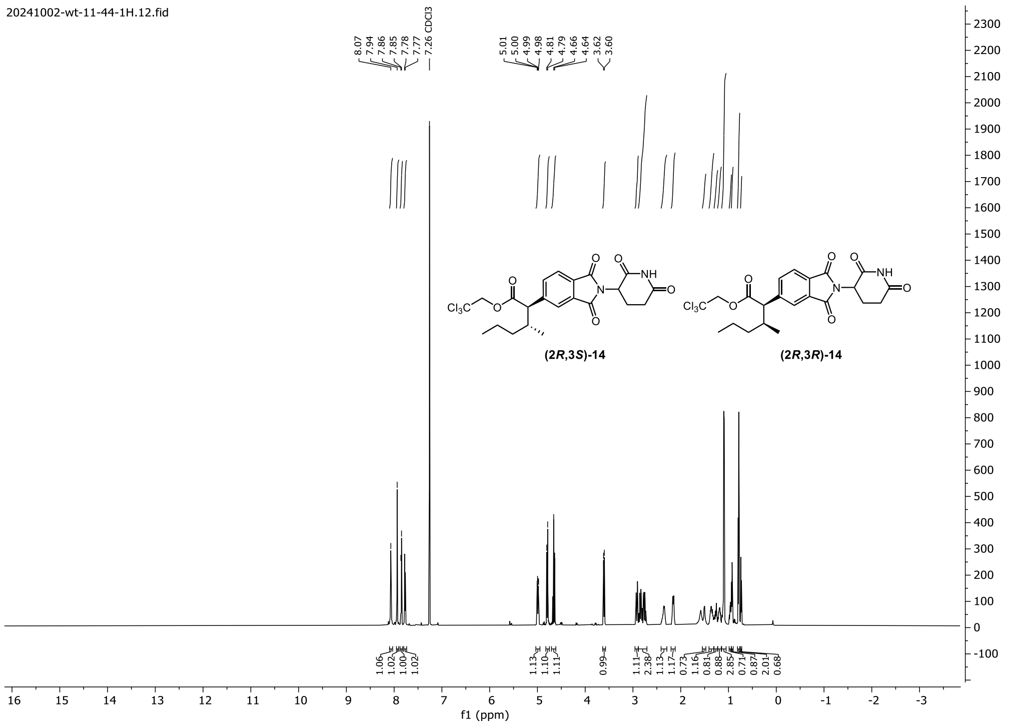

^{1}H NMR Spectrum for Compound 15 (400 MHz).

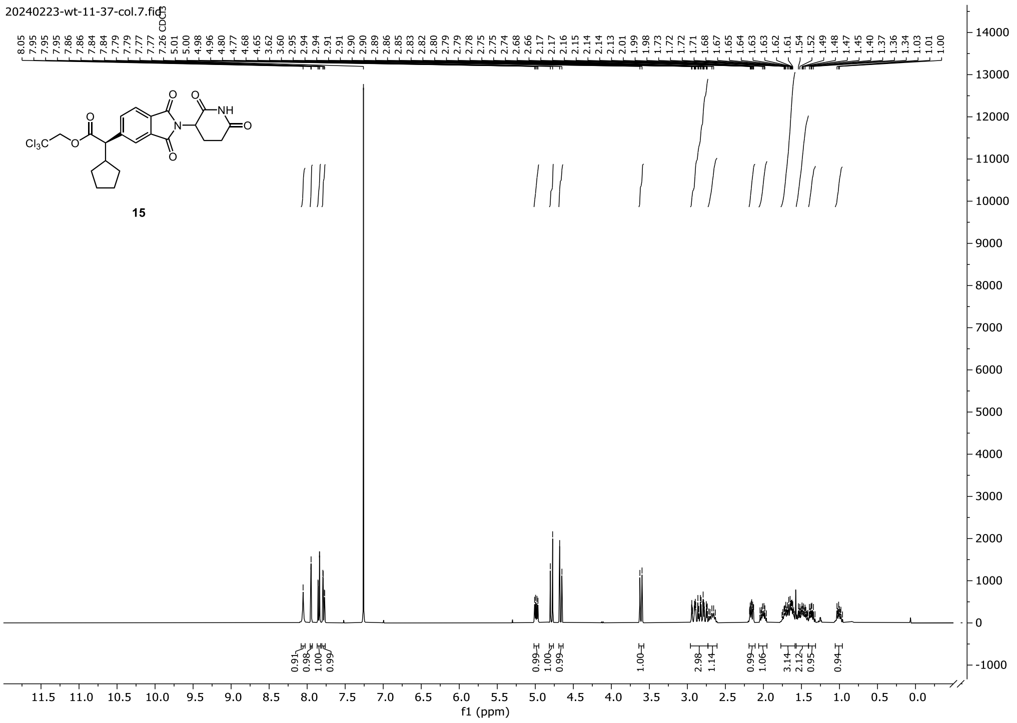

^{1}H NMR Spectrum for Compound 16 (400 MHz).

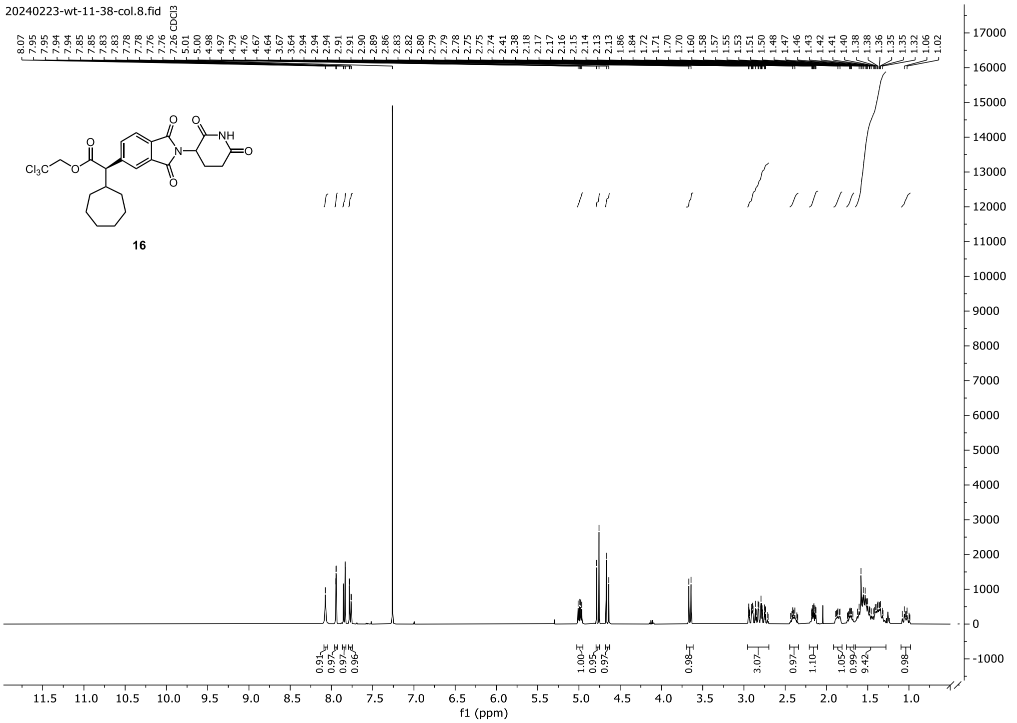

^{1}H NMR Spectrum for Compound 17 (600 MHz).

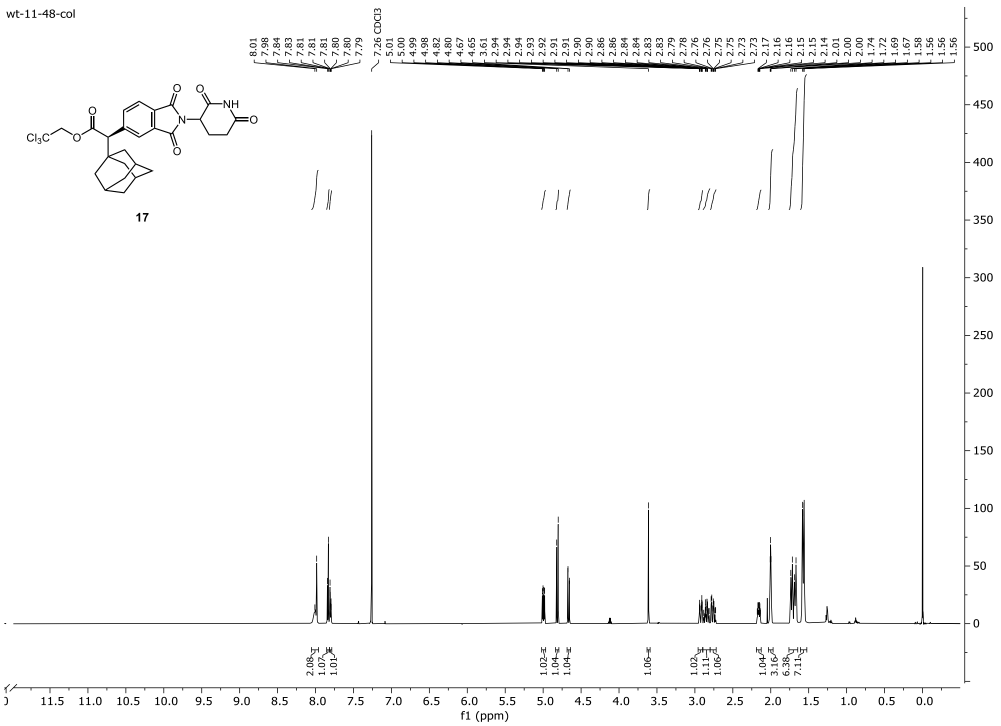

^{1}H NMR Spectrum for Compound 18 (400 MHz).

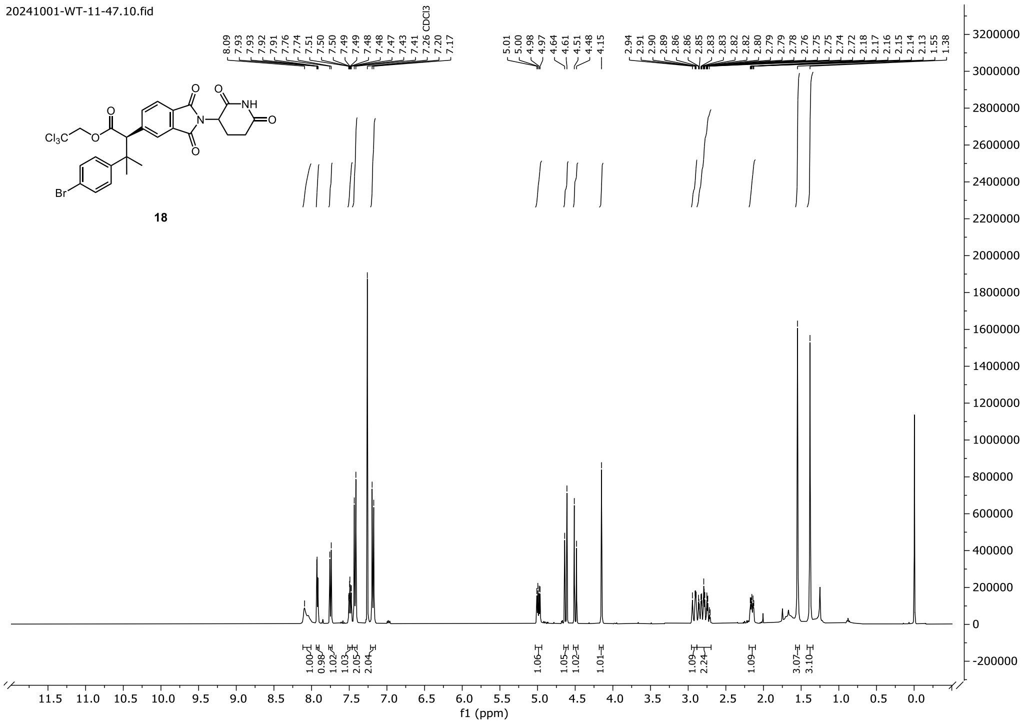

^{1}H NMR Spectrum for Compound 19 (400 MHz).

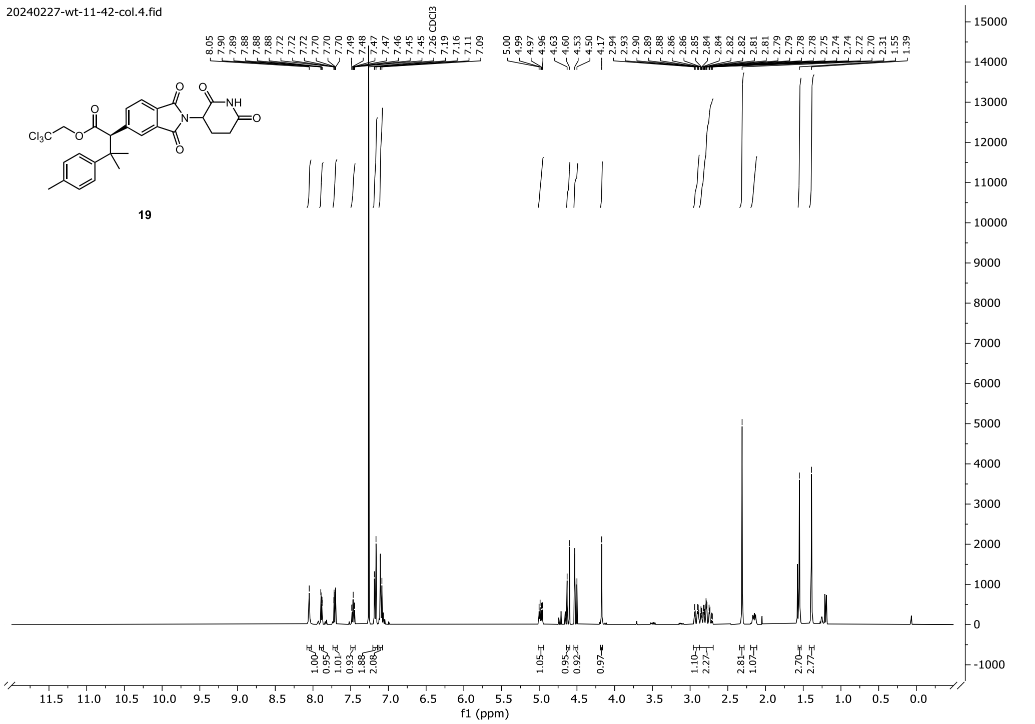

^{1}H NMR Spectrum for Compound 20 (400 MHz).

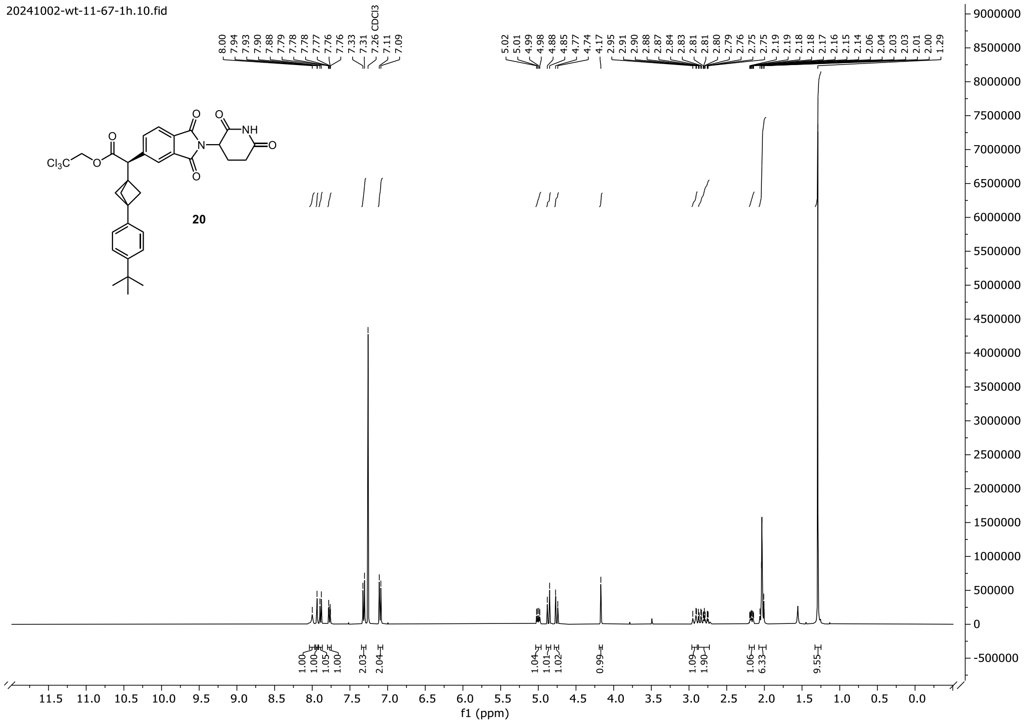

^{1}H NMR Spectrum for Compound 21 (600 MHz).

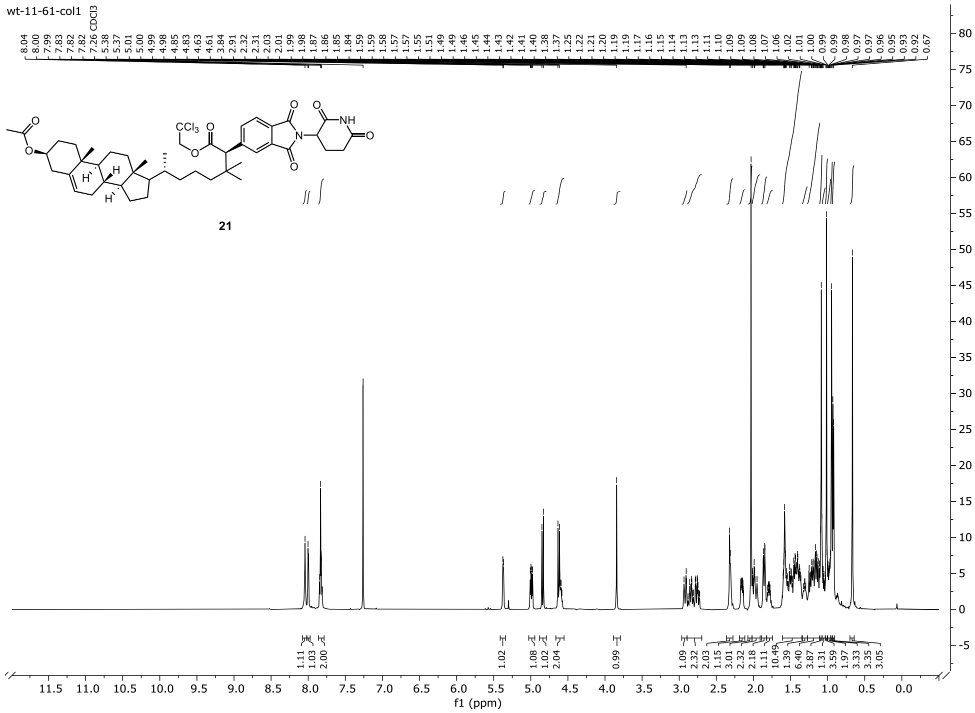

^{1}H NMR Spectrum for Compound 22 (400 MHz).

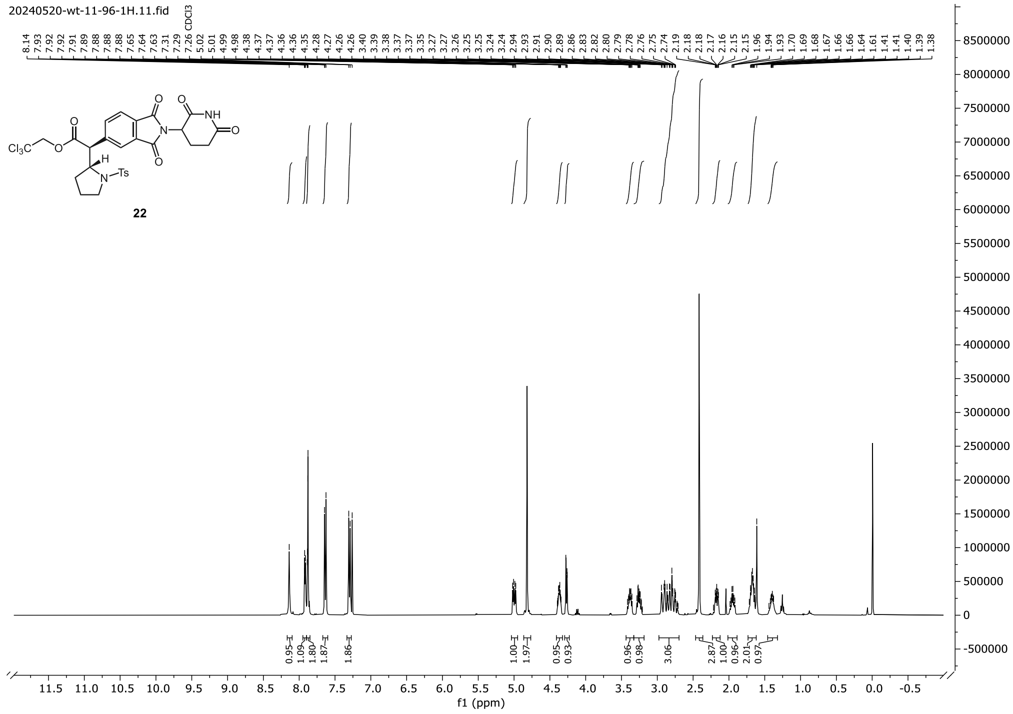

^{1}H NMR Spectrum for Compound 23 (400 MHz).

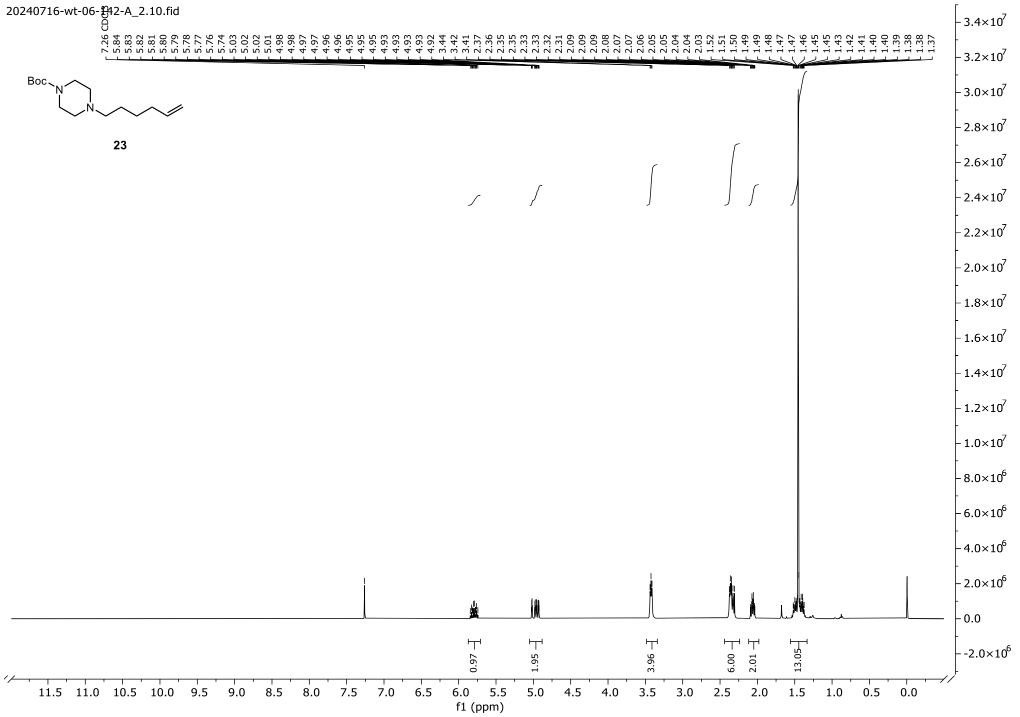

^{1}H NMR Spectrum for Compound 24 (400 MHz).

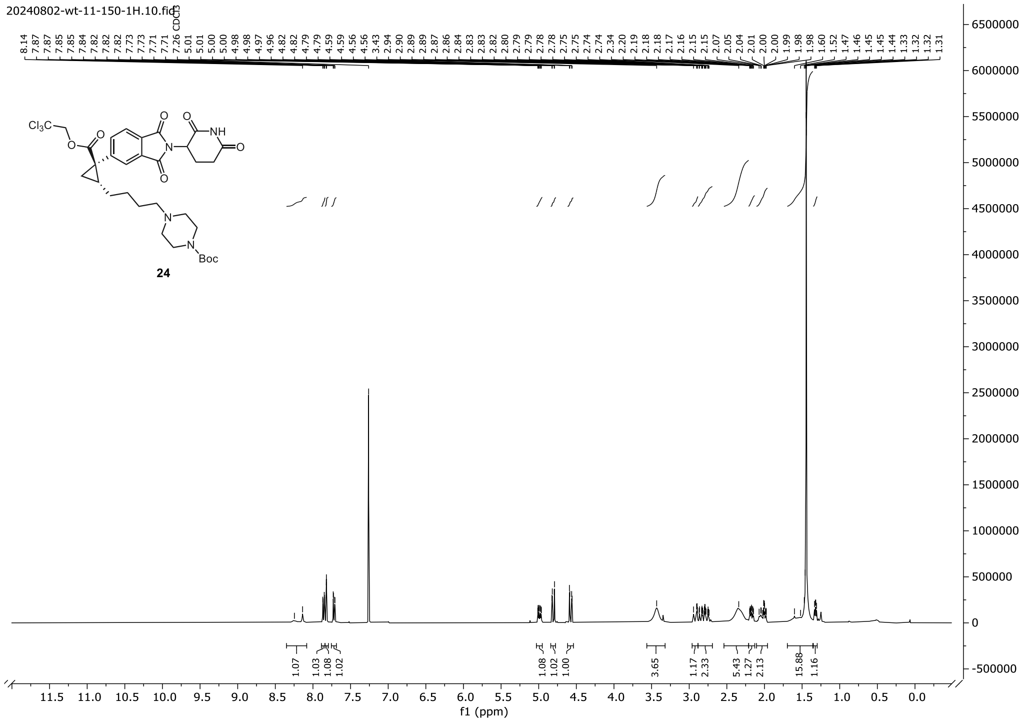

^{1}H NMR Spectrum for Compound 25 at 23 °C (800 MHz).

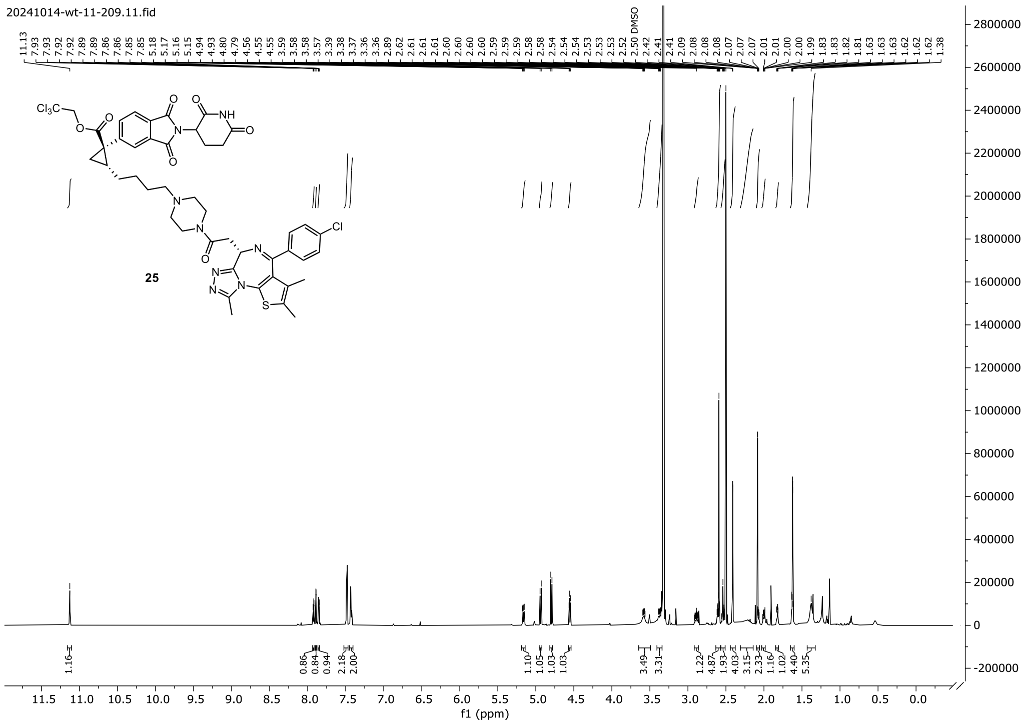

^{1}H NMR Spectrum for Compound 25 at 80 °C (800 MHz).

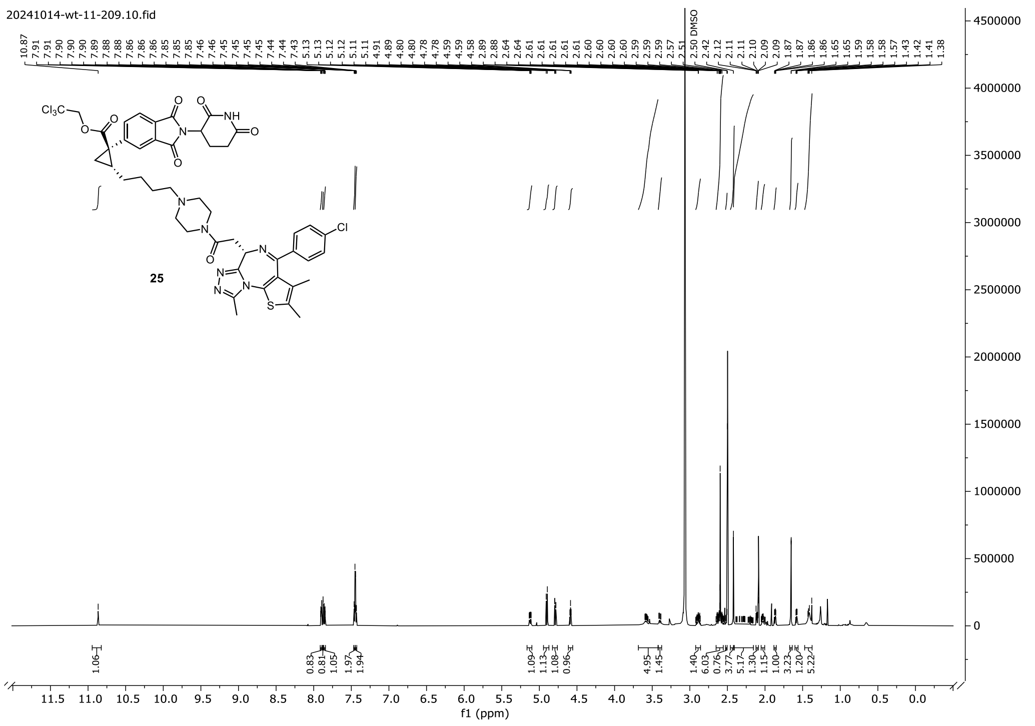

^{1}H NMR Spectrum for Compound 26 at 23 °C (800 MHz).

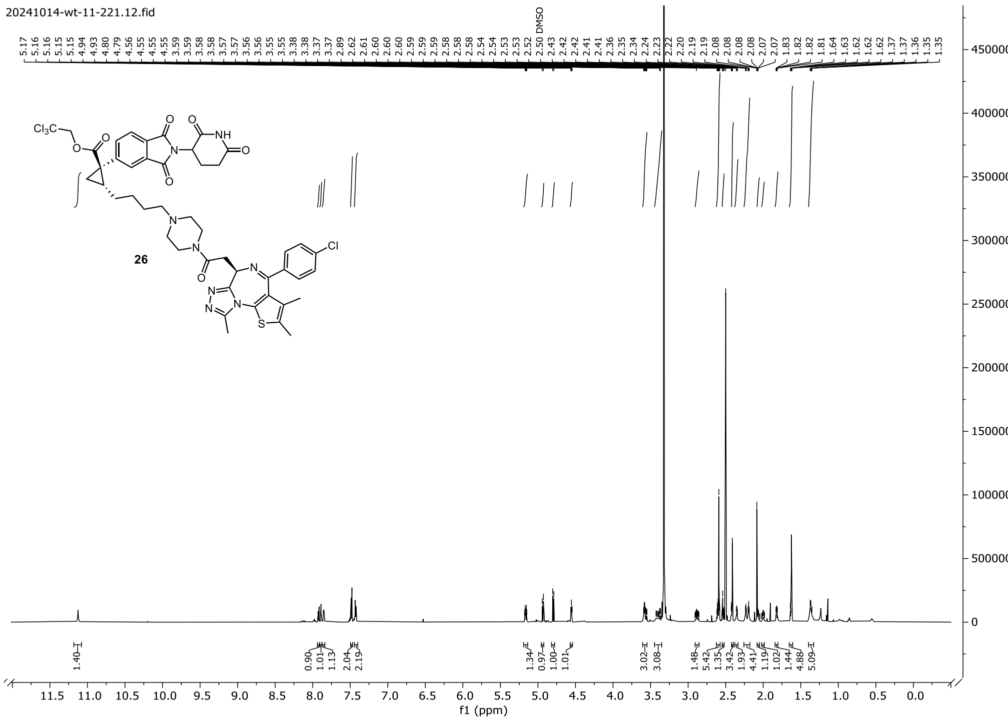

^{1}H NMR Spectrum for Compound 26 at 80 °C (800 MHz).

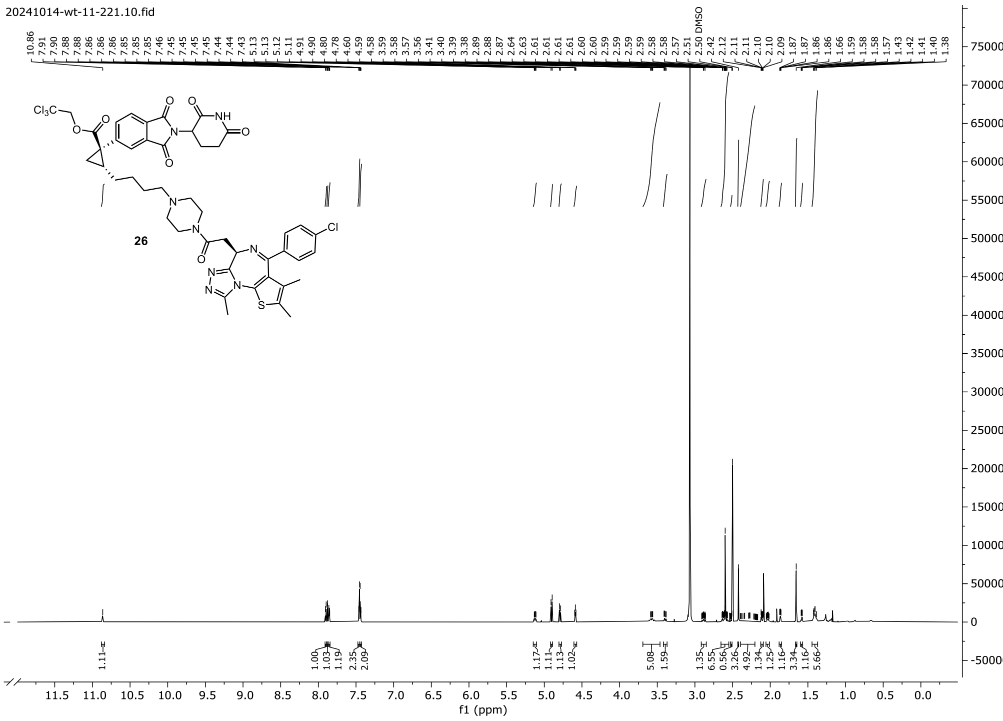

^{1}H NMR Spectrum for Compound SI2 (600 MHz).

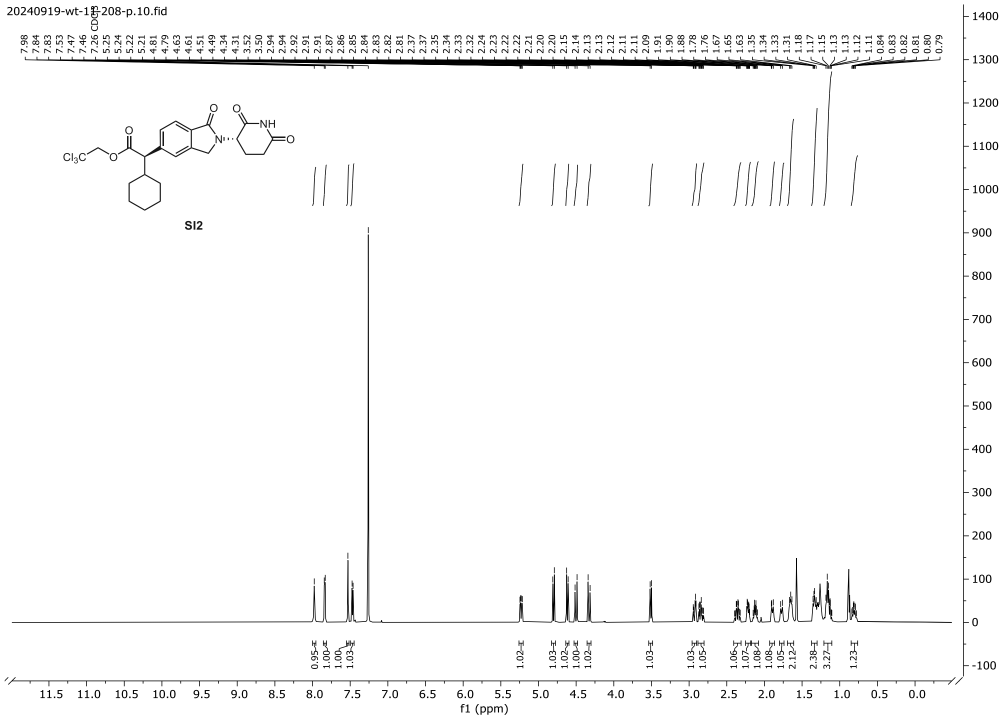

^{1}H NMR Spectrum for Compound SI3 (400 MHz).

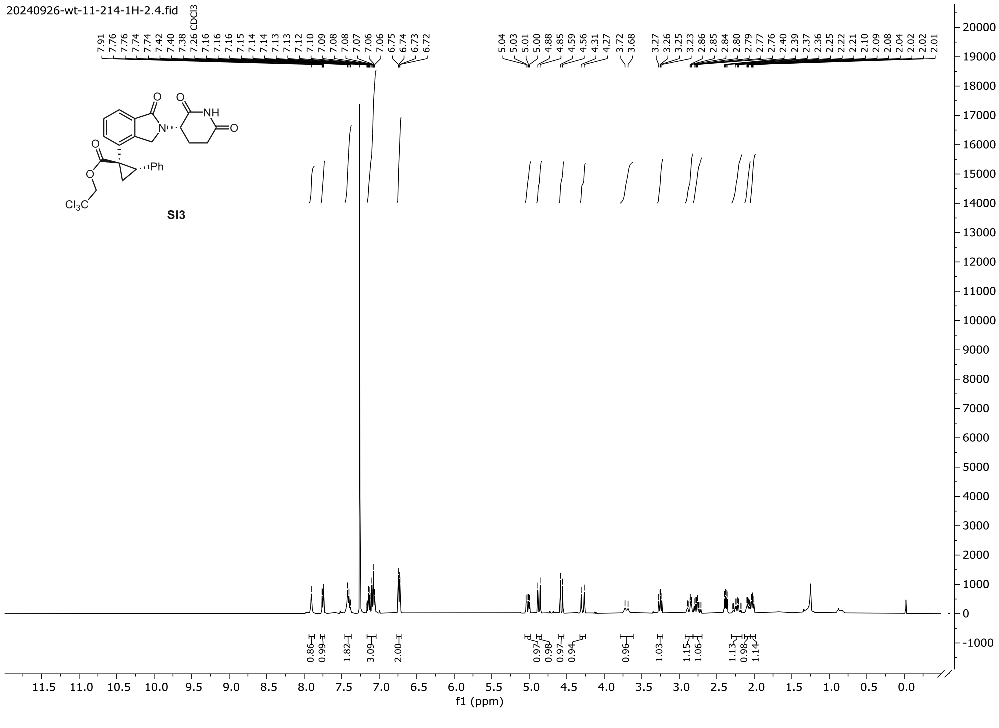

^{1}H NMR Spectrum for Compound SI4 (400 MHz).

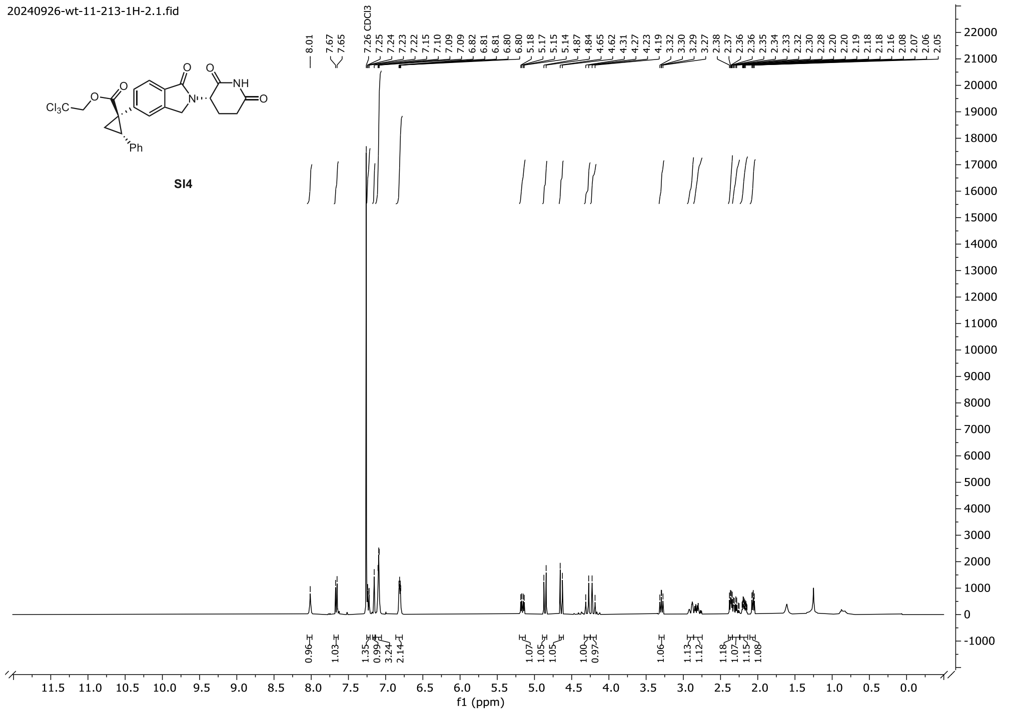

$^{13}C\{^{1}H\}$ NMR Spectra

$^{13}C\{^{1}H\}$ Spectrum for Compound 4 (151 MHz).

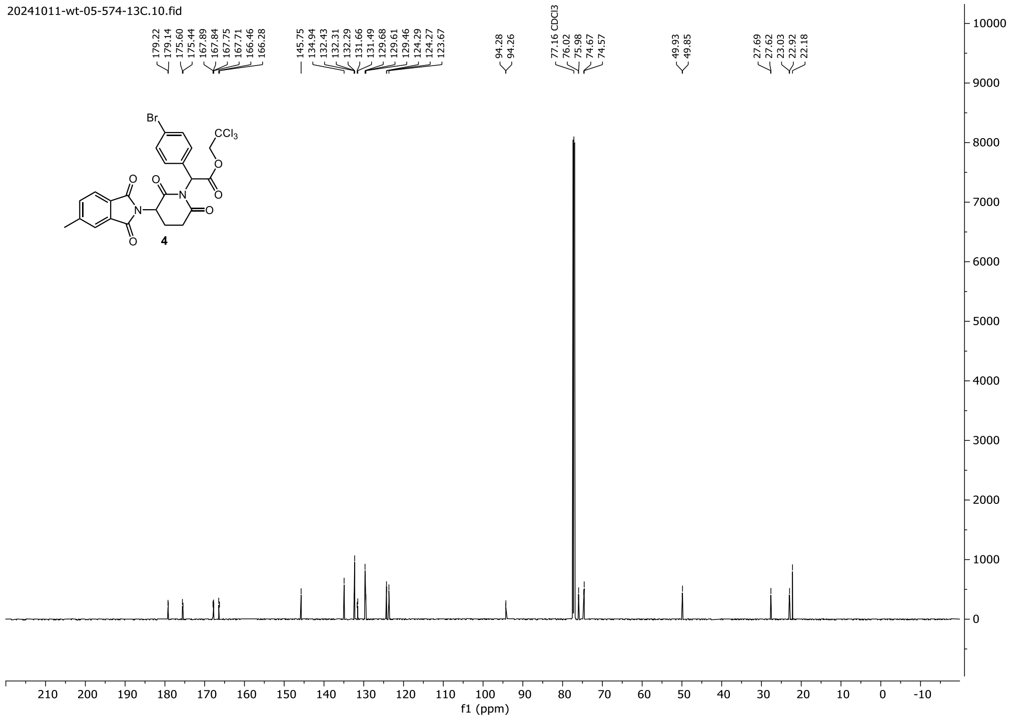

$^{13}C\{^1H\}$ Spectrum for Compound 6a (151 MHz).

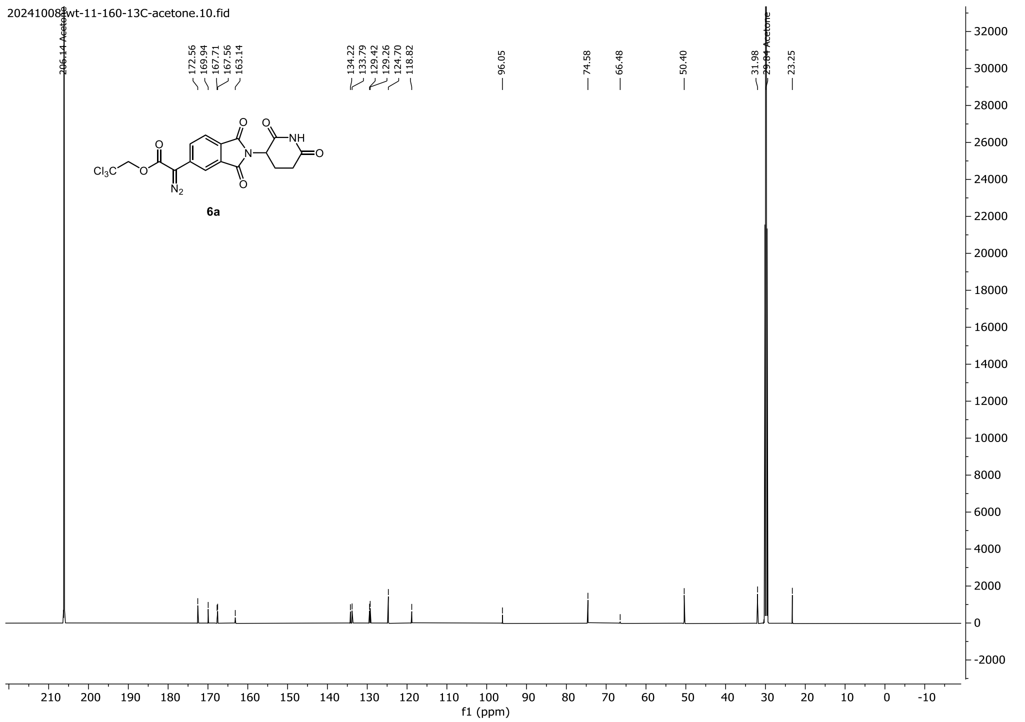

$^{13}C\{^1H\}$ Spectrum for Compound 6b (151 MHz).

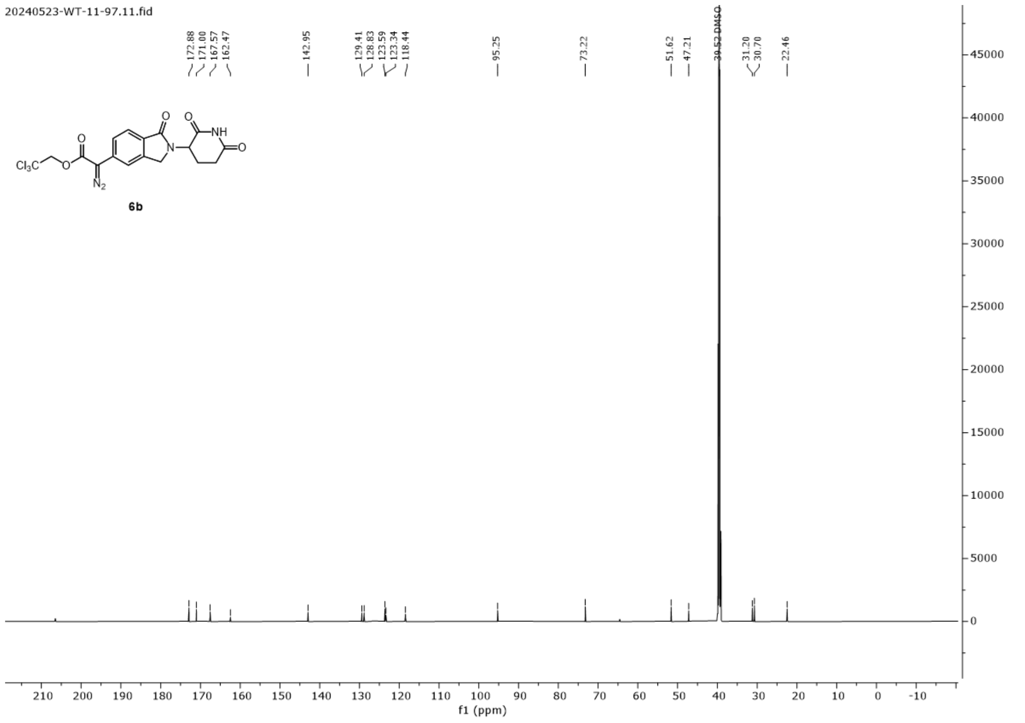

$^{13}C\{^{1}H\}$ Spectrum for Compound 6c (151 MHz).

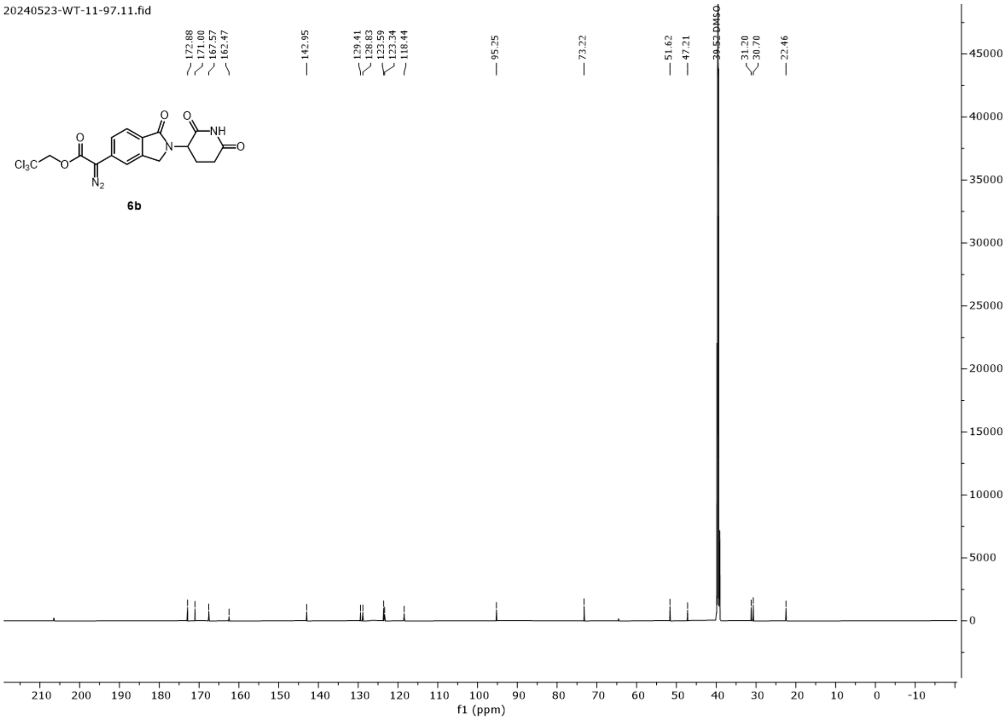

$^{13}C\{^{1}H\}$ Spectrum for Compound 8a (101 MHz).

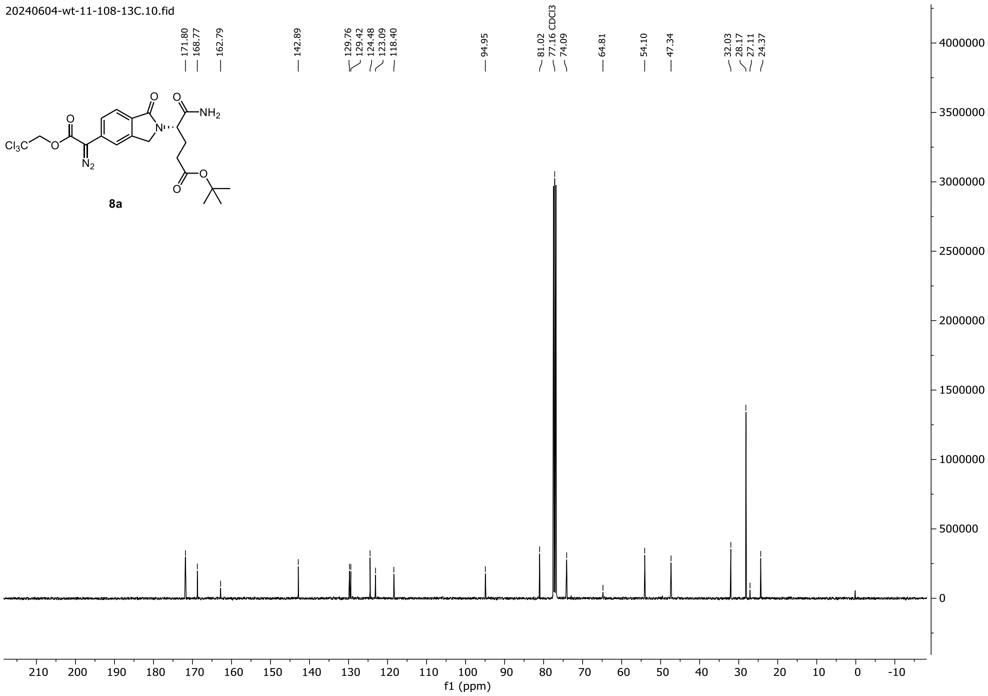

$^{13}C\{^1H\}$ Spectrum for Compound 8b (151 MHz).

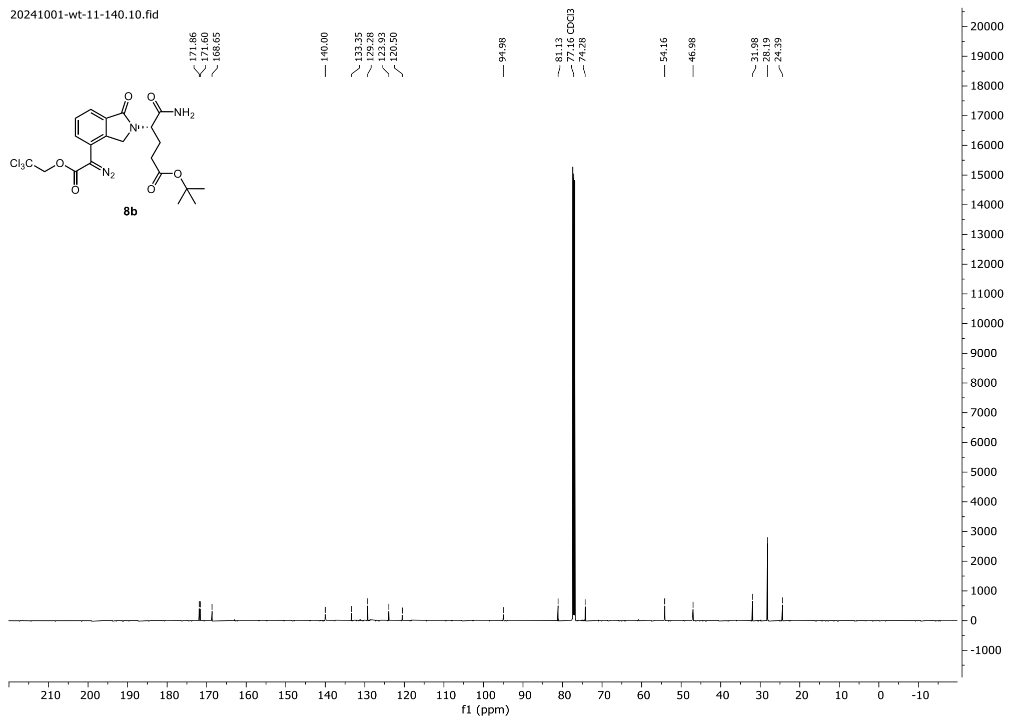

$^{13}C\{^1H\}$ Spectrum for Compound 9a (151 MHz).

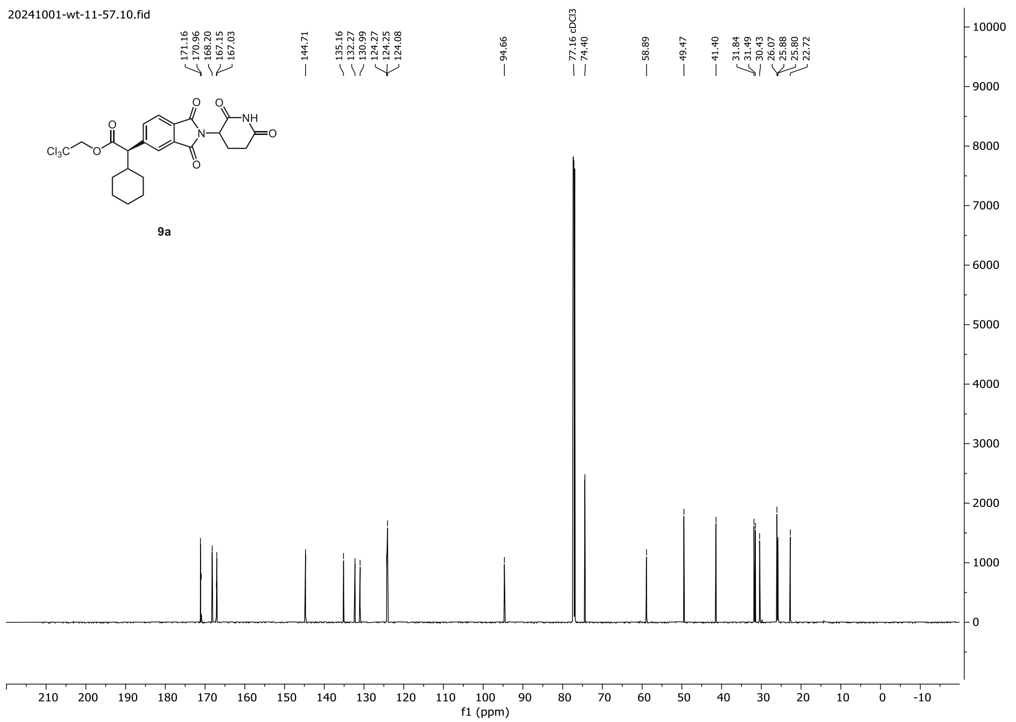

$^{13}C\{^{1}H\}$ Spectrum for Compound 9b (151 MHz).

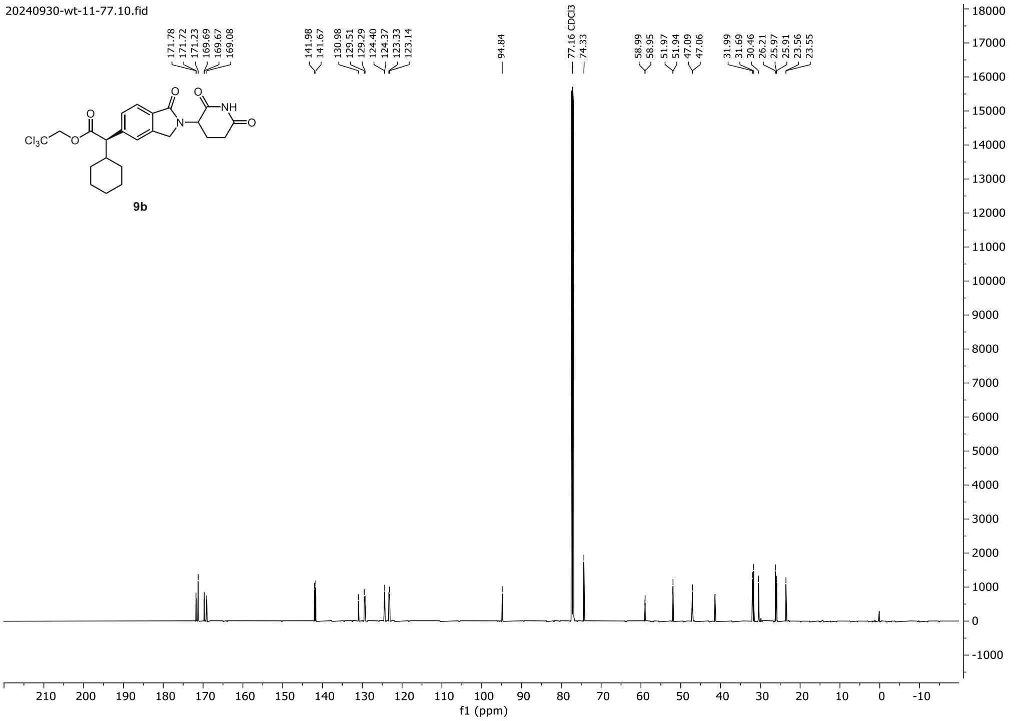

$^{13}C\{^{1}H\}$ Spectrum for Compound 9c (151 MHz).

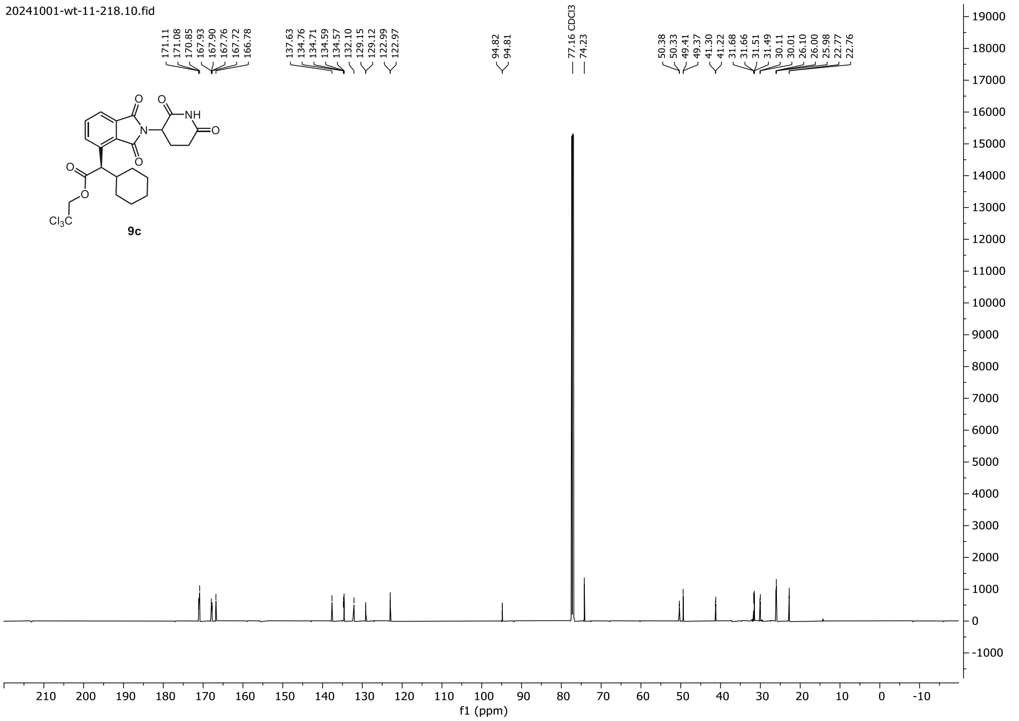

$^{13}C\{^1H\}$ Spectrum for Compound (S,R)-10a (151 MHz).

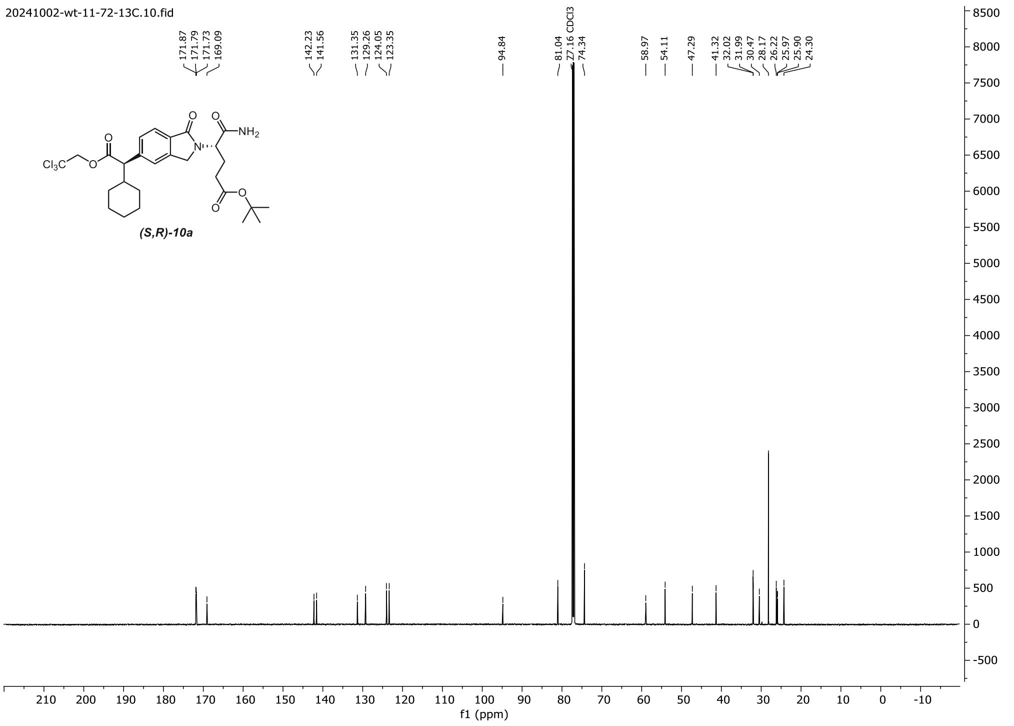

$^{13}C\{^1H\}$ Spectrum for Compound (S,S)-10a (151 MHz).

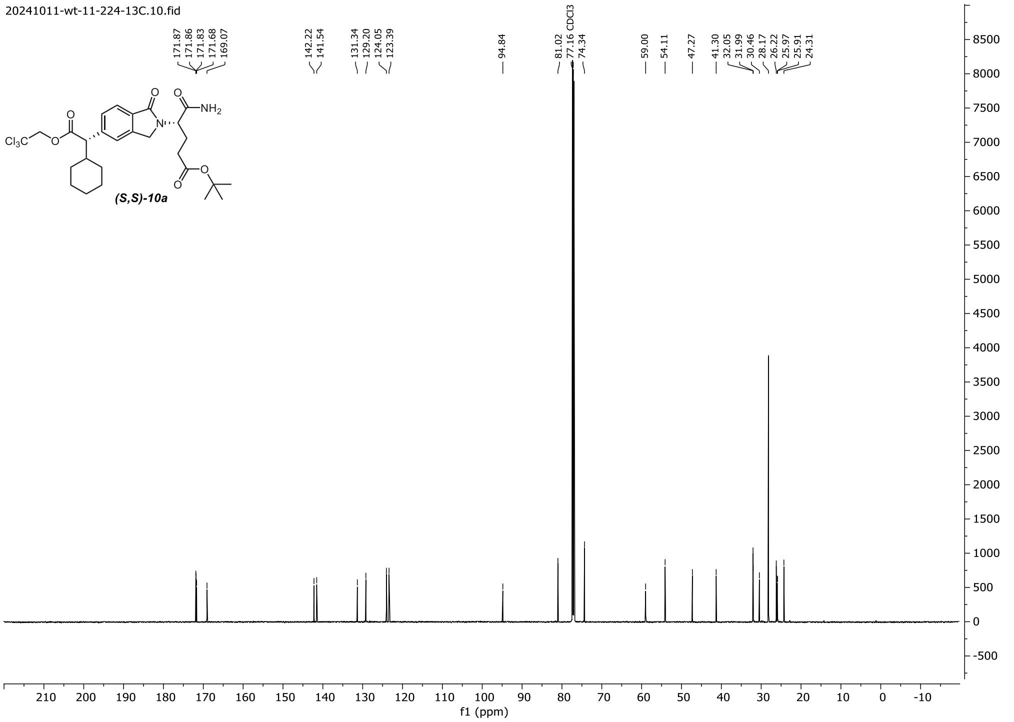

$^{13}C\{^1H\}$ Spectrum for Compound 10b (151 MHz).

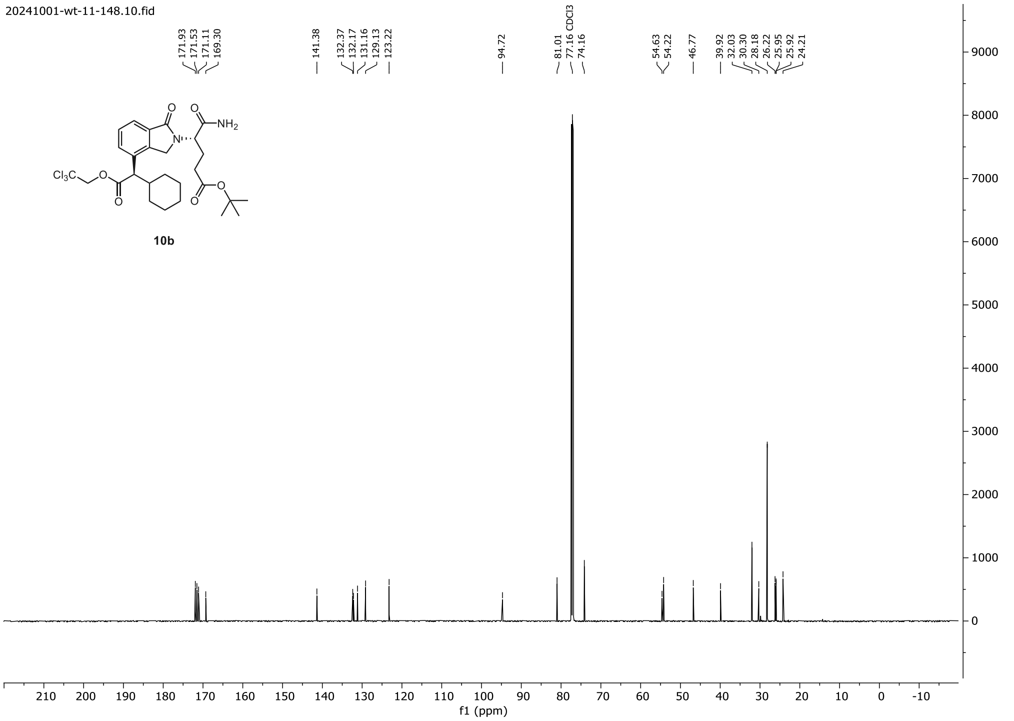

$^{13}C\{^1H\}$ Spectrum for Compound 11a (151 MHz).

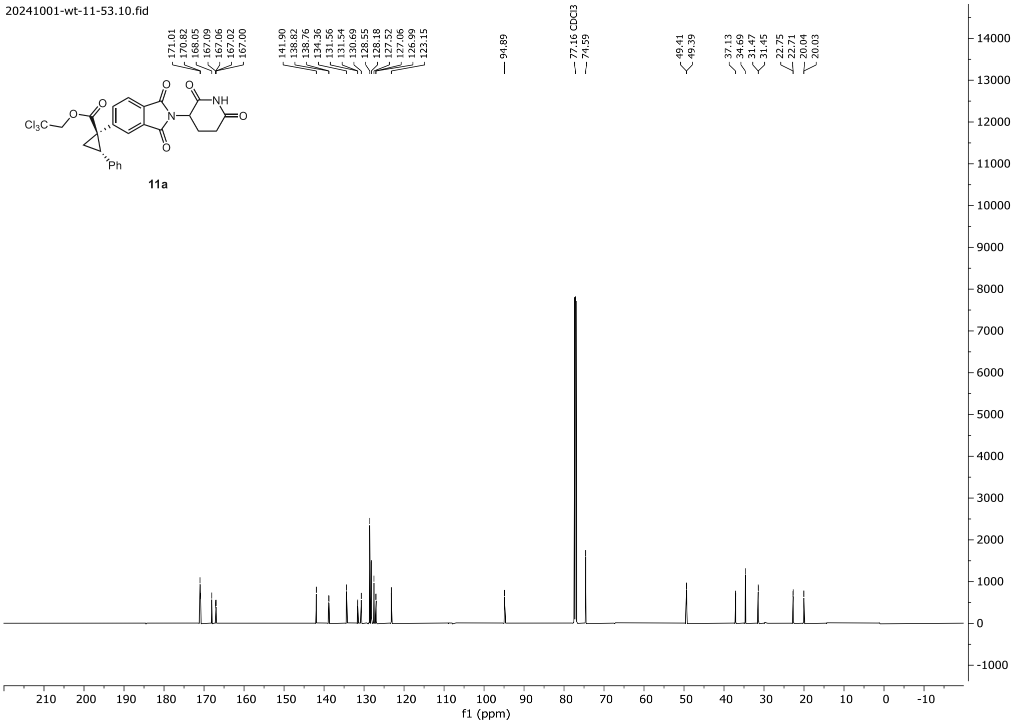

$^{13}C\{^1H\}$ Spectrum for Compound 11b (151 MHz).

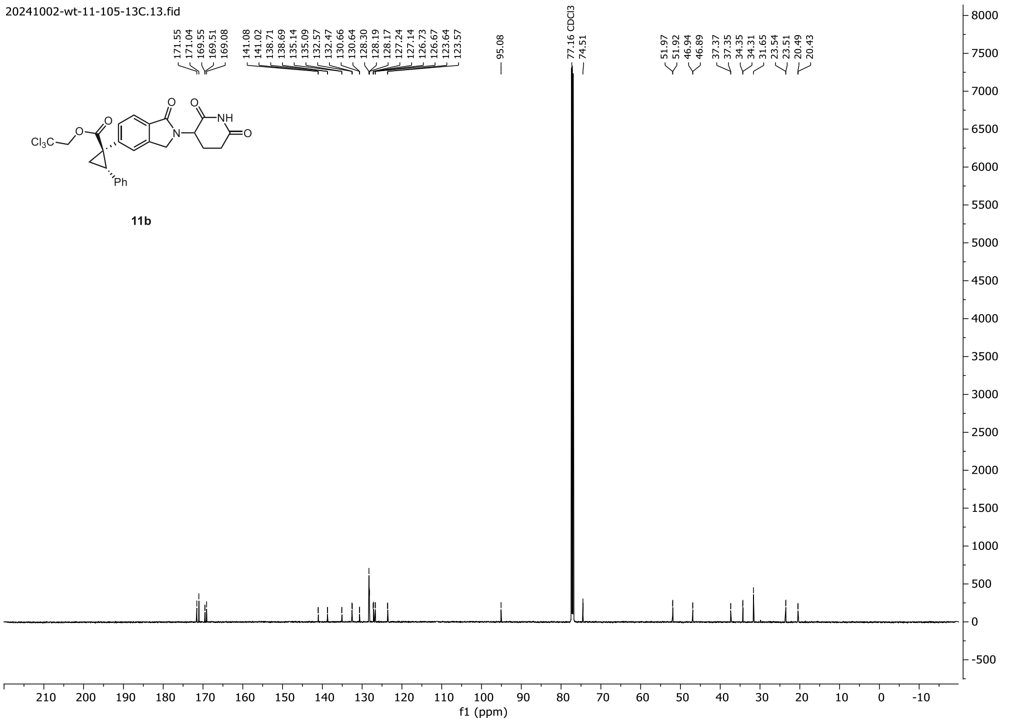

$^{13}C\{^1H\}$ Spectrum for Compound 11c (151 MHz).

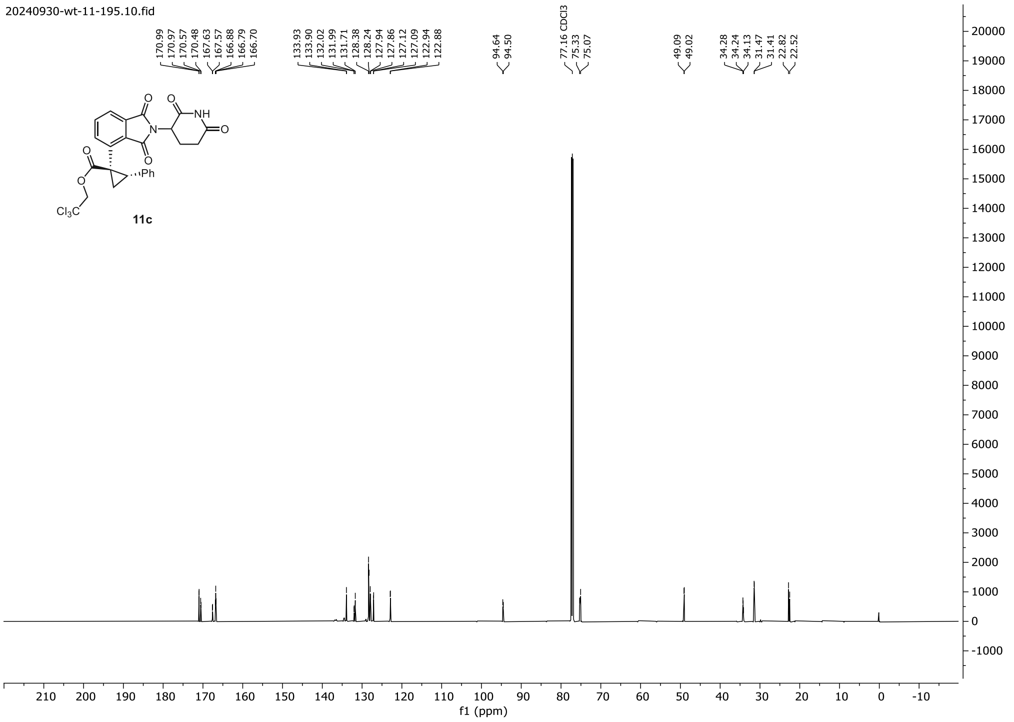

$^{13}C\{^{1}H\}$ Spectrum for Compound 12a (101 MHz).

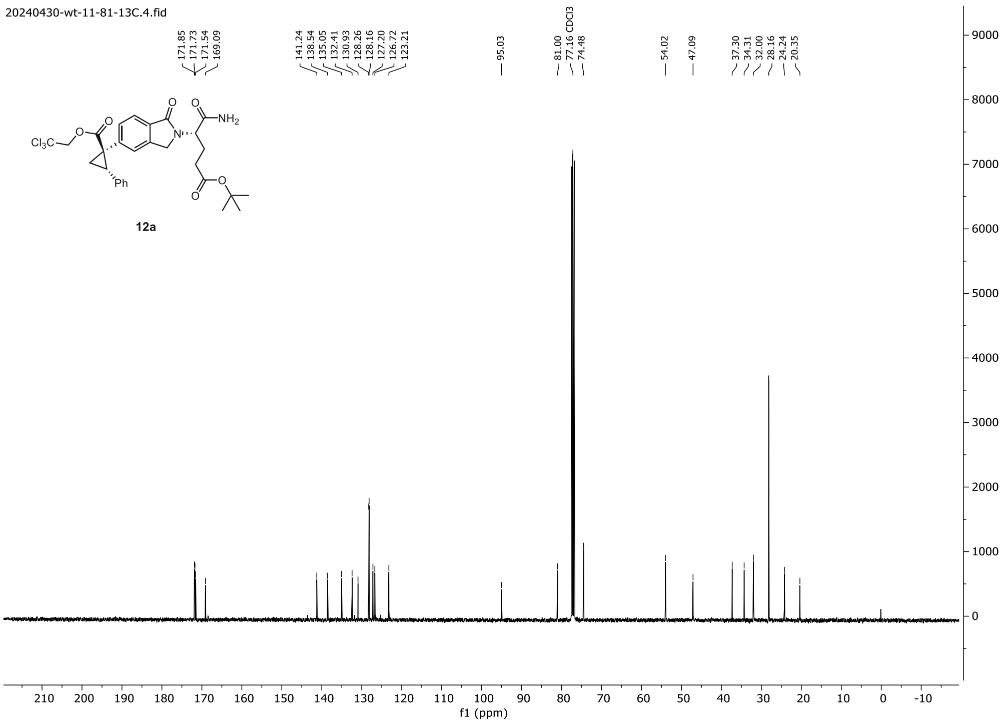

$^{13}C\{^{1}H\}$ Spectrum for Compound 12b (151 MHz).

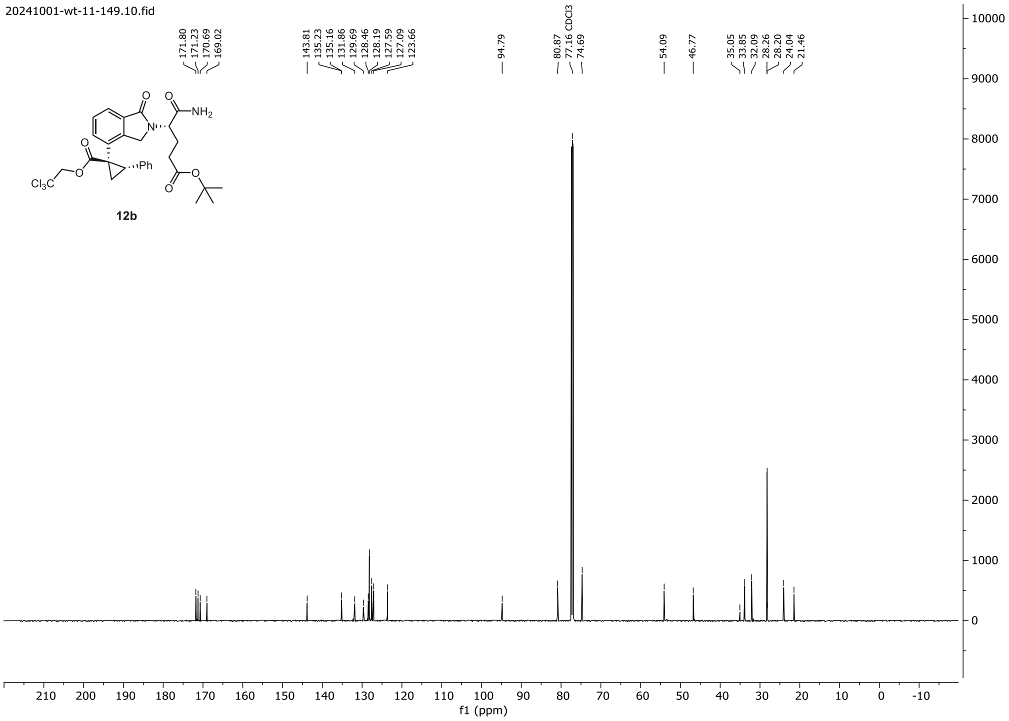

$^{13}C\{^1H\}$ Spectrum for Compound 13 (101 MHz).

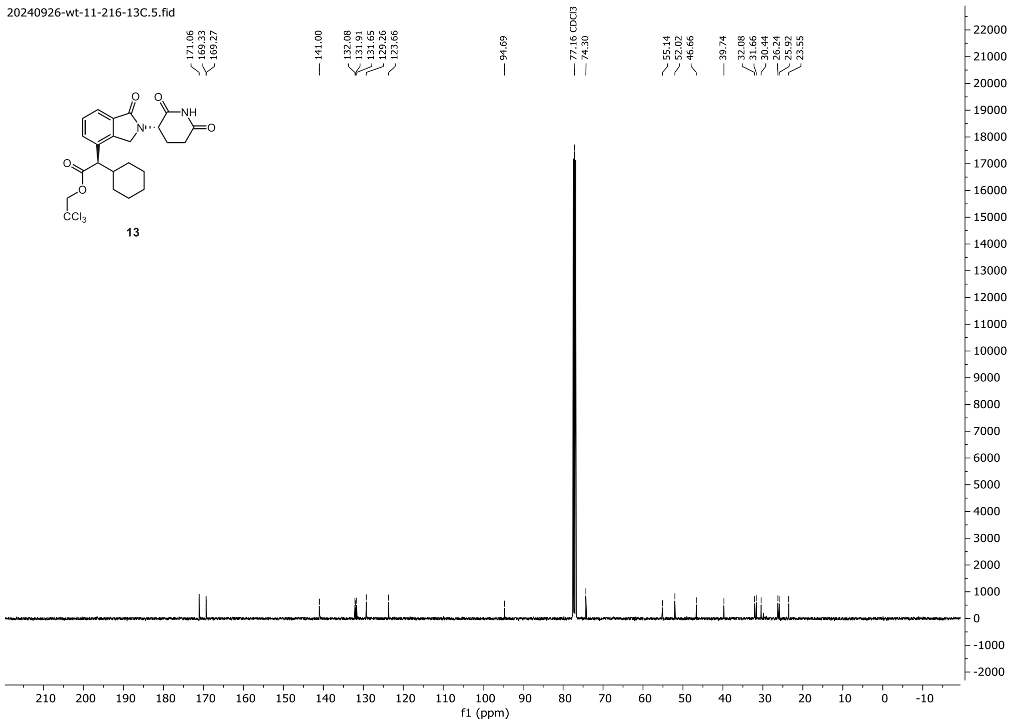

$^{13}C\{^1H\}$ Spectrum for Compound 14 (151 MHz).

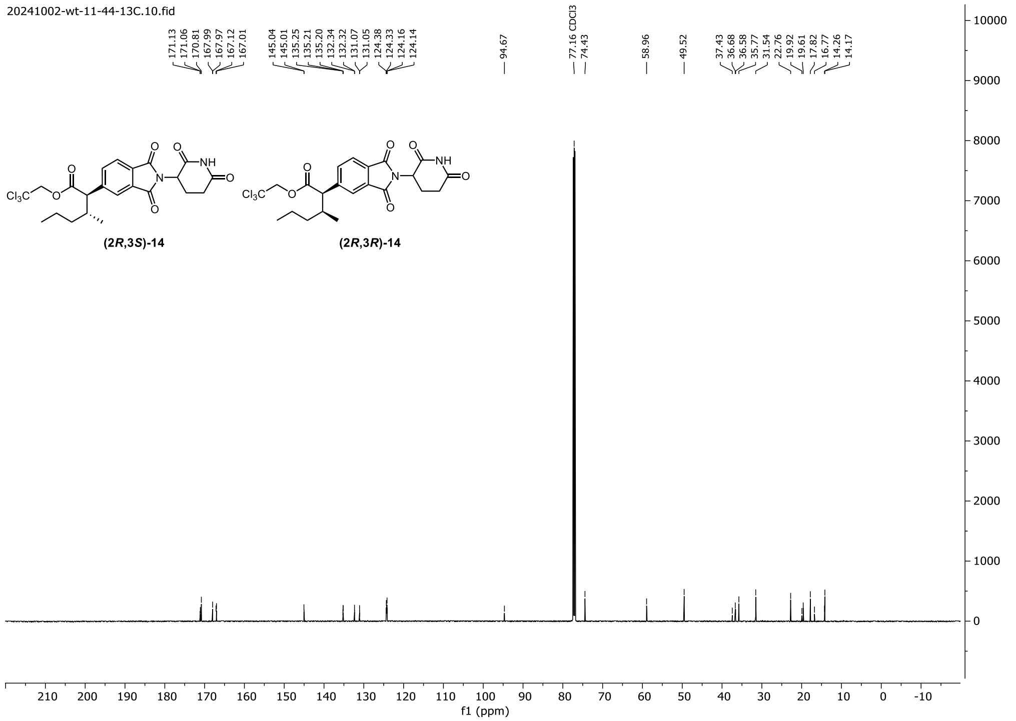

$^{13}C\{^1H\}$ Spectrum for Compound 15 (151 MHz).

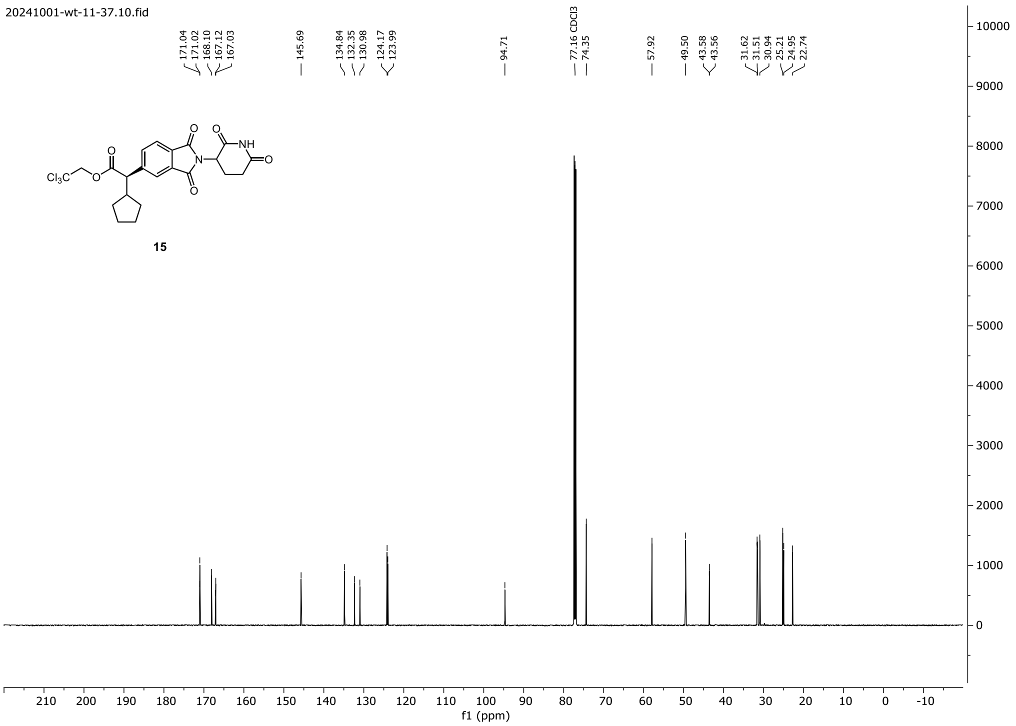

$^{13}C\{^1H\}$ Spectrum for Compound 16 (151 MHz).

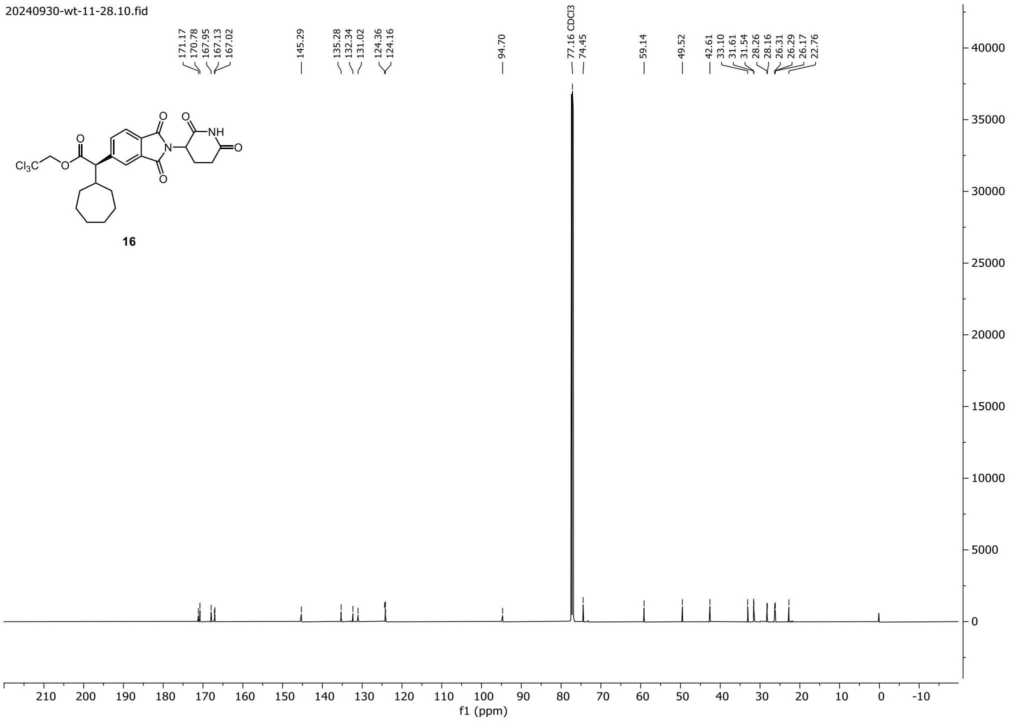

$^{13}C\{^1H\}$ Spectrum for Compound 17 (151 MHz).

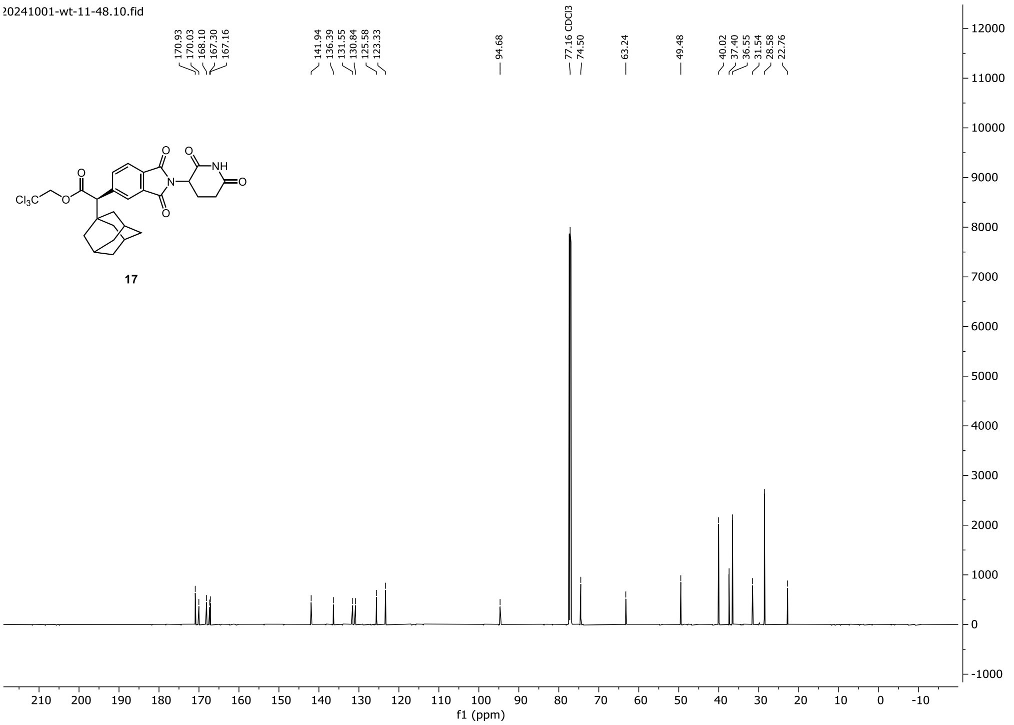

$^{13}C\{^1H\}$ Spectrum for Compound 18 (151 MHz).

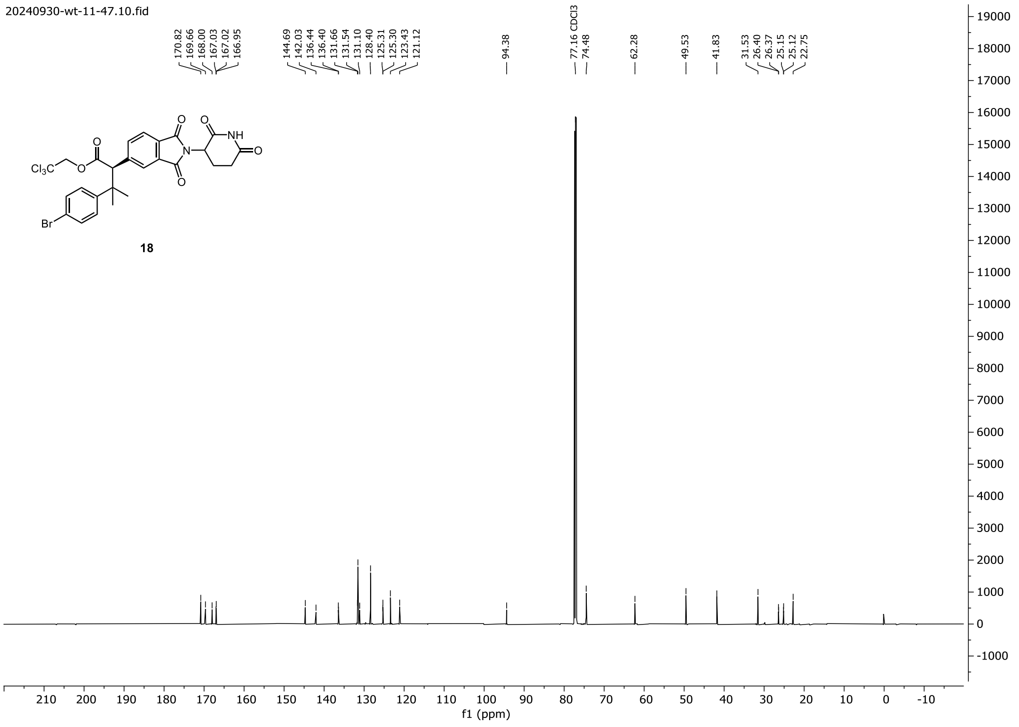

$^{13}C\{^1H\}$ Spectrum for Compound 19 (151 MHz).

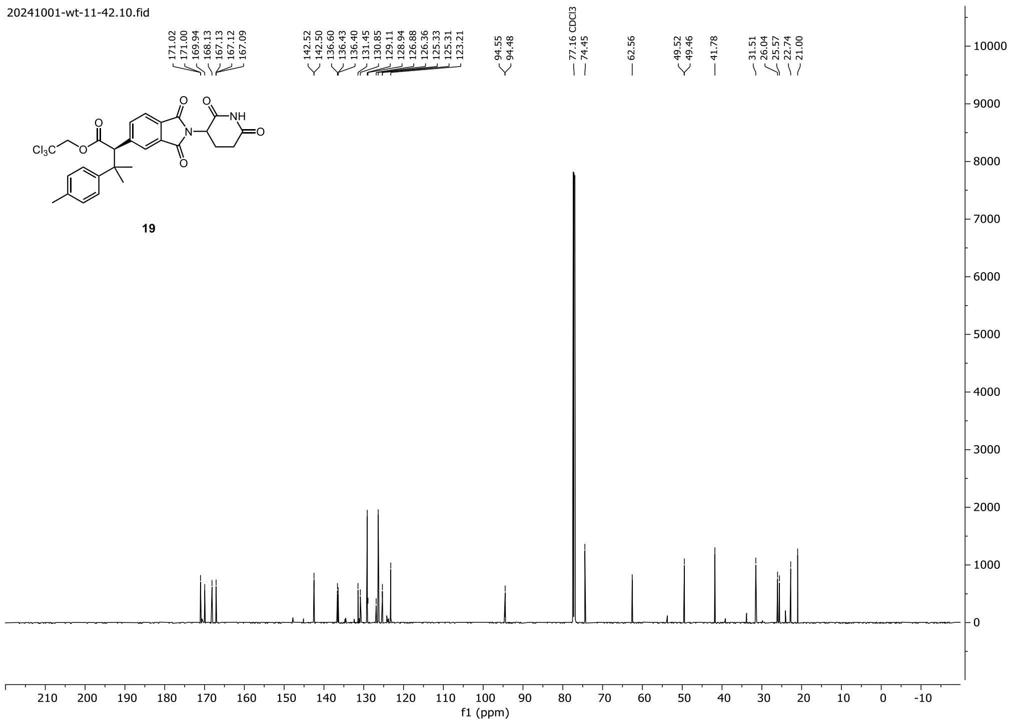

$^{13}C\{^1H\}$ Spectrum for Compound 20 (151 MHz).

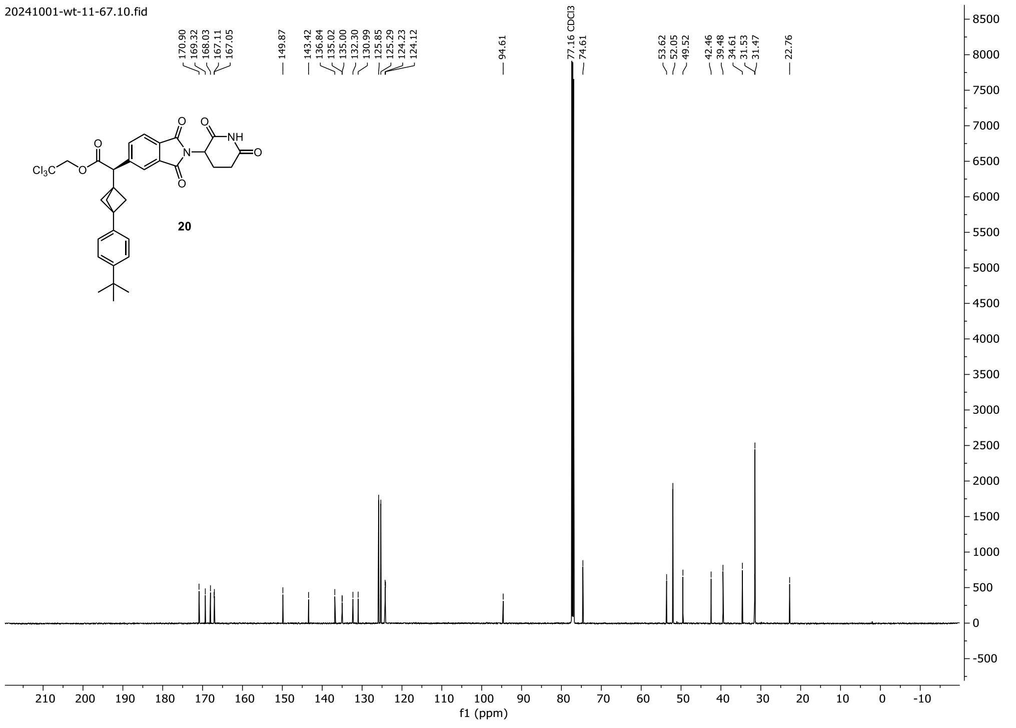

$^{13}C\{^{1}H\}$ Spectrum for Compound 21 (151 MHz).

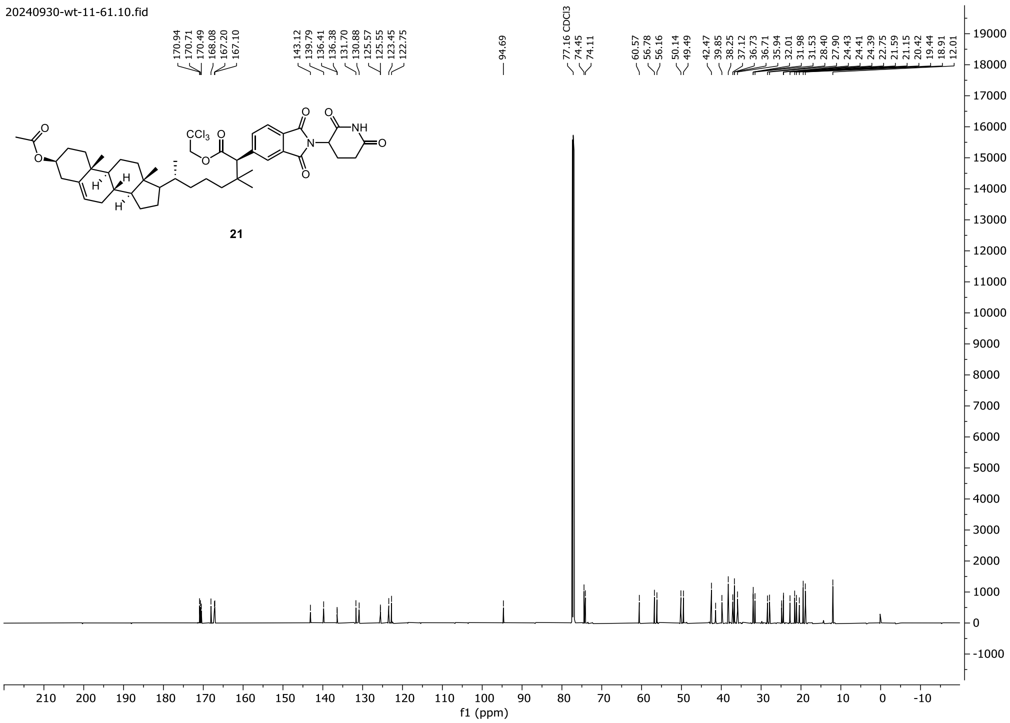

$^{13}C\{^{1}H\}$ Spectrum for Compound 22 (101 MHz).

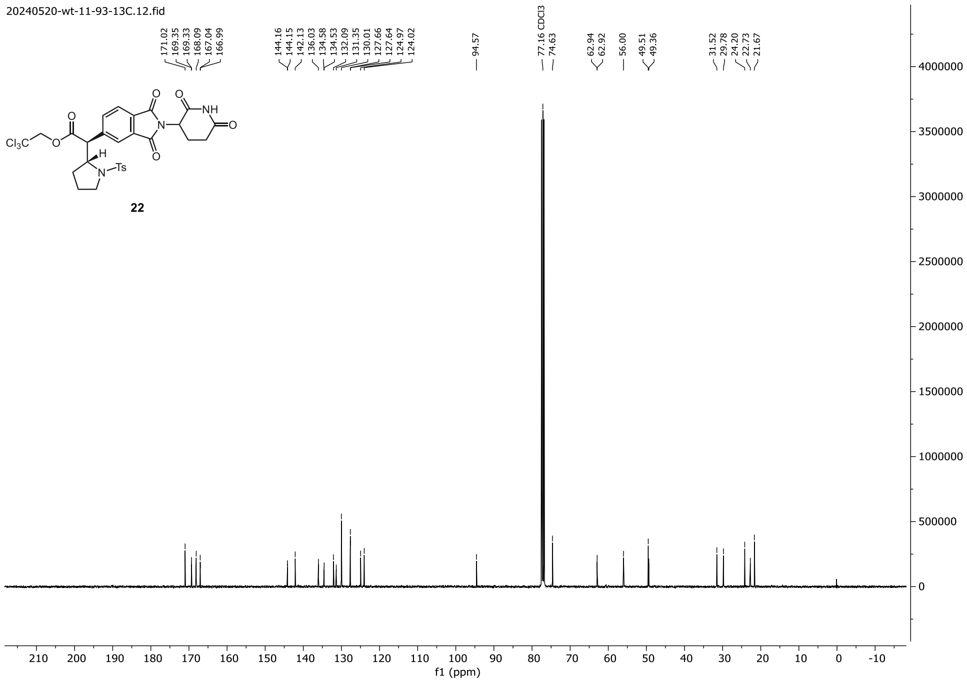

$^{13}C\{^{1}H\}$ Spectrum for Compound 23 (151 MHz).

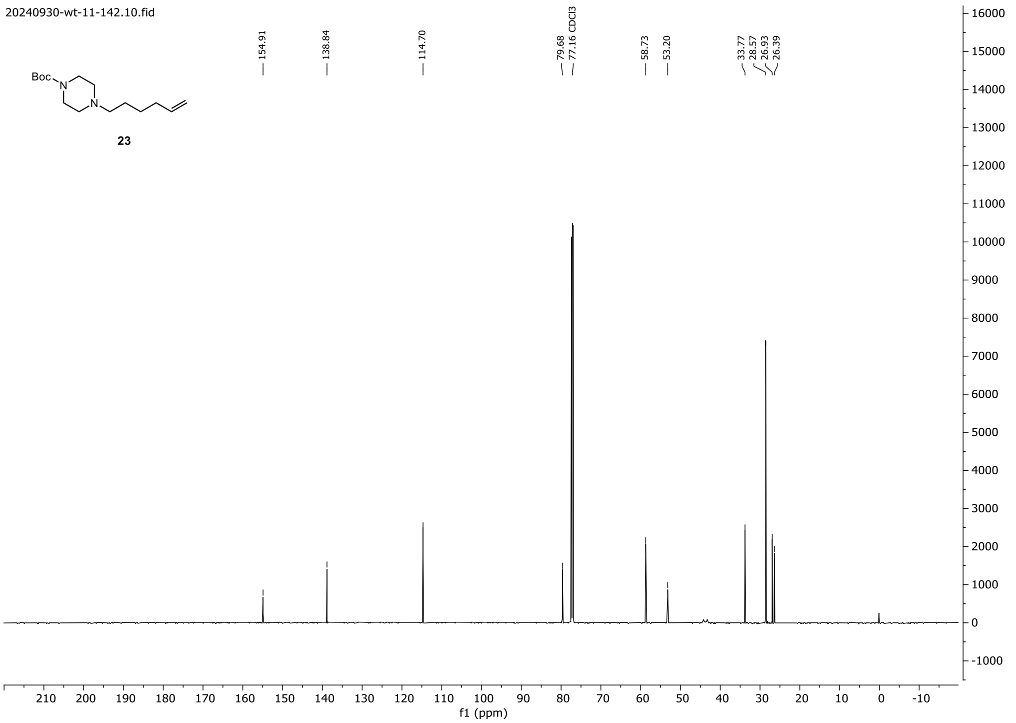

$^{13}C\{^{1}H\}$ Spectrum for Compound 24 (151 MHz).

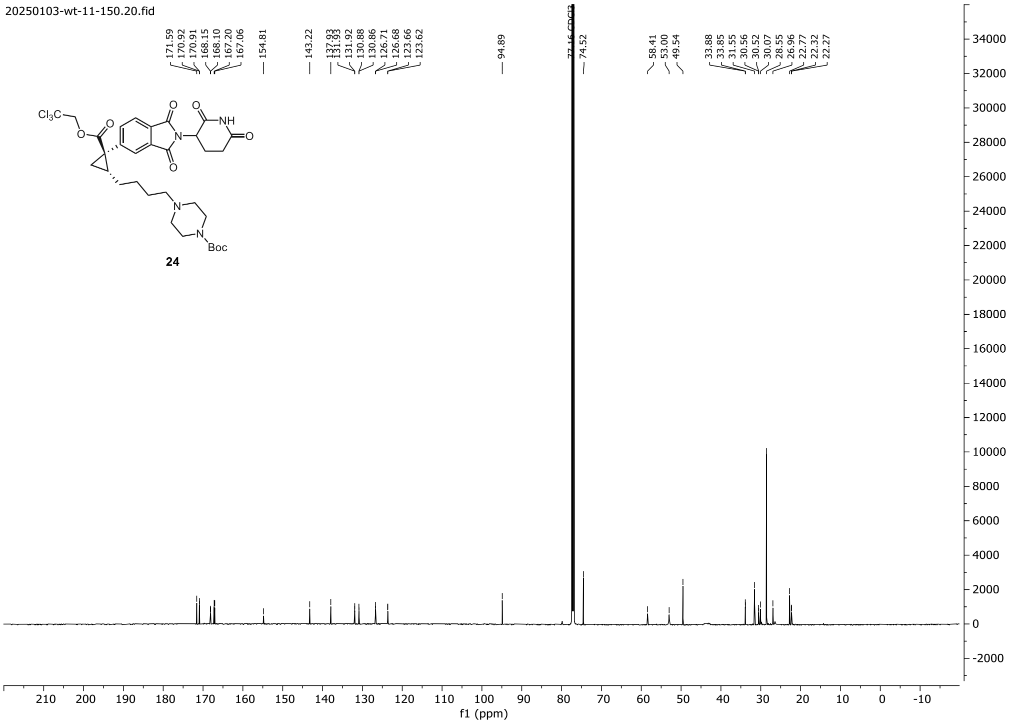

$^{13}C\{^1H\}$ Spectrum for Compound 25 at 80 °C (201 MHz).

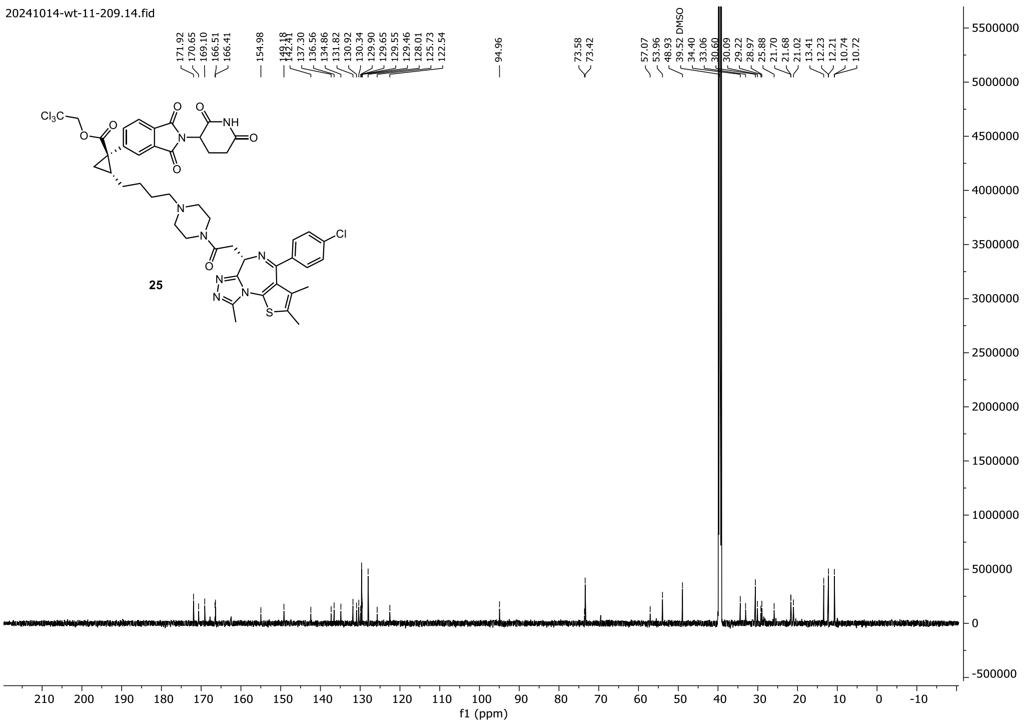

$^{13}C\{^1H\}$ Spectrum for Compound 26 at 80 °C (201 MHz).

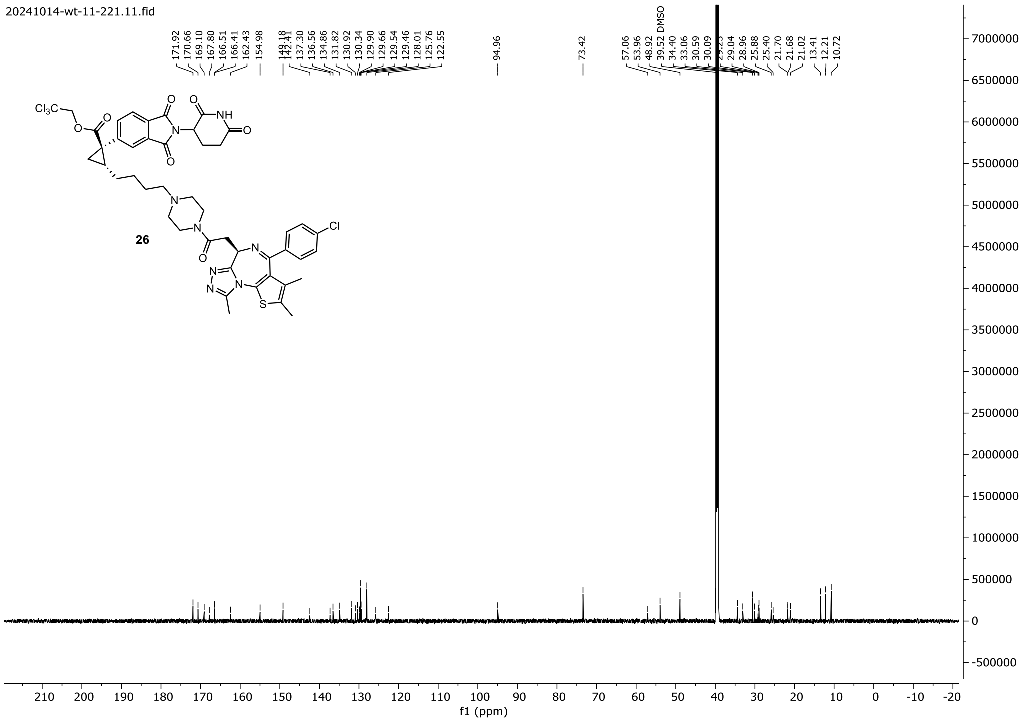

$^{13}C\{^1H\}$ Spectrum for Compound SI2 (101 MHz).

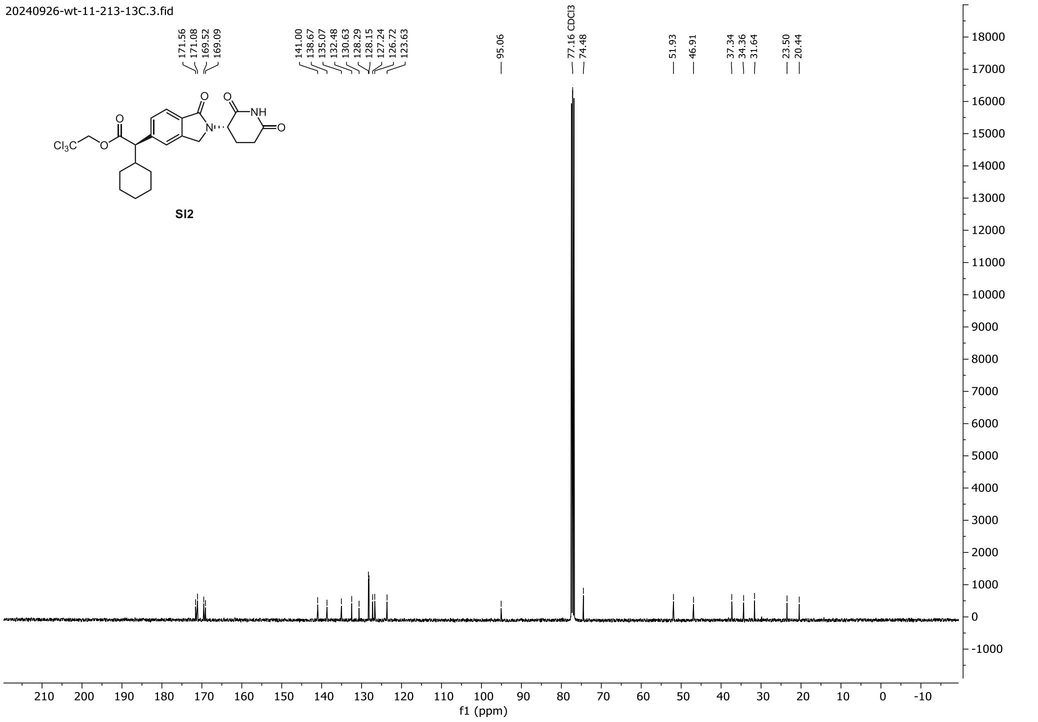

$^{13}C\{^1H\}$ Spectrum for Compound SI3 (101 MHz).

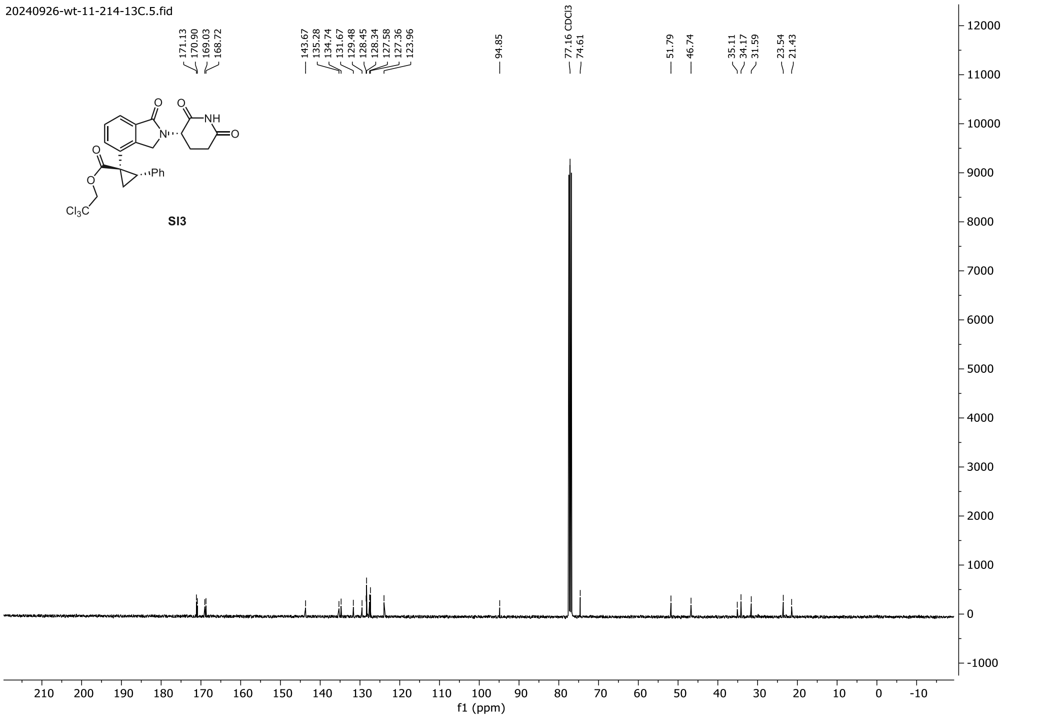

$^{13}C\{^1H\}$ *Spectrum for Compound SI4 (101 MHz).*

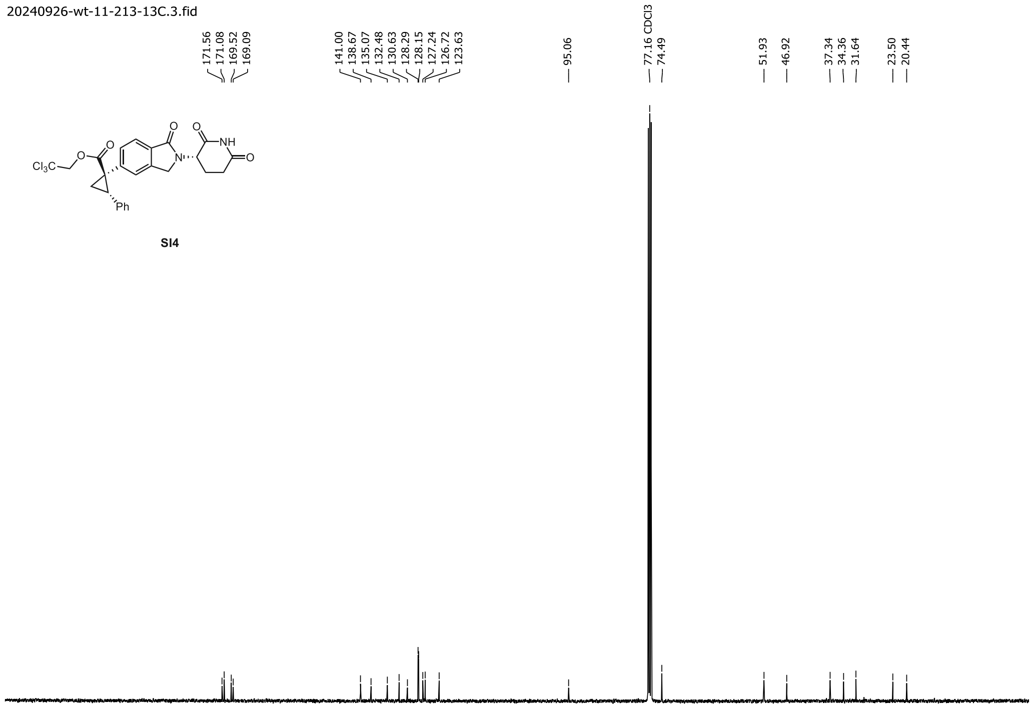

Supplementary NMR Spectra

^1H *NMR spectrum for 10b showing support for d.r. determination*

10b *epimerized with DBU showing appearance of diastereomeric peaks*

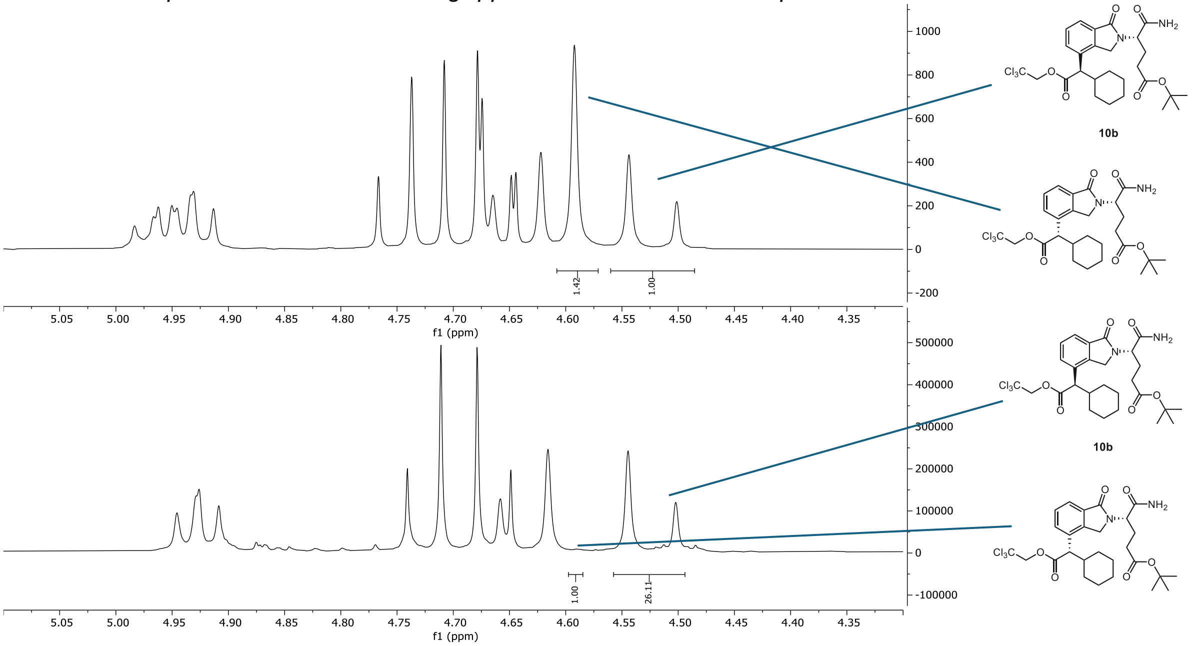

Reaction of ***8b*** *with S catalyst showing absence of diastereomeric peaks in* ***10b***

^{1}H NMR spectrum for (S,R)-10a and (S,R)-10a showing support for d.r. determination

*Reaction of **8a** with R/S catalyst showing appearance of diastereomeric peaks for **(S,S)-10a***

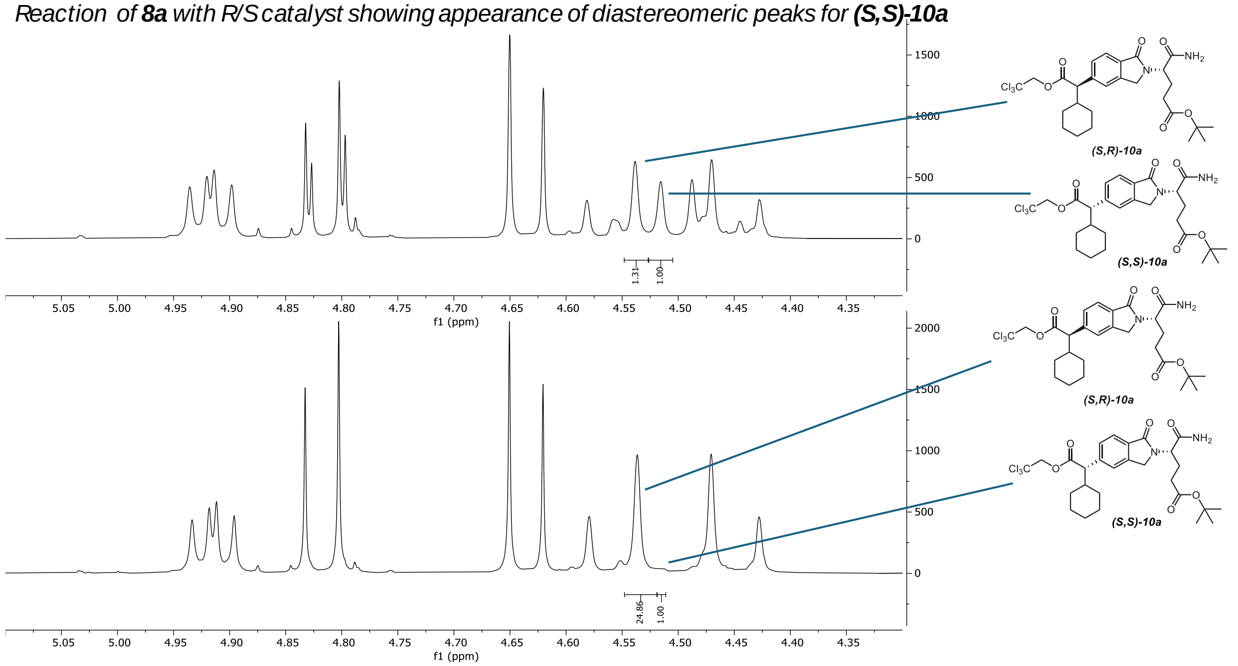

*Reaction of **8a** with S catalyst showing disappearance of diastereomeric peaks for **(S,S)-10a***

^{1}H NMR spectrum for 12b showing support for d.r. determination

*Reaction of **xx** with $Rh_2(esp)_2$ showing appearance of diastereomeric peaks for **12b***

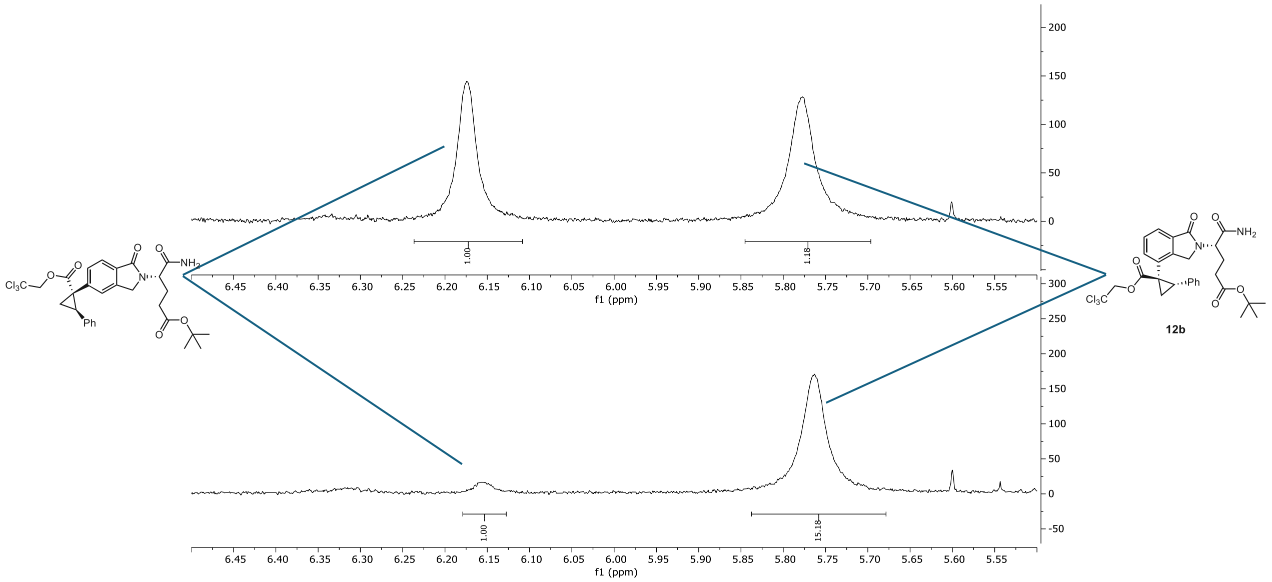

*Reaction of **8b** with S catalyst showing disappearance of diastereomeric peaks for **12b***

^{1}H NMR spectrum for 14 showing support for r.r. determination

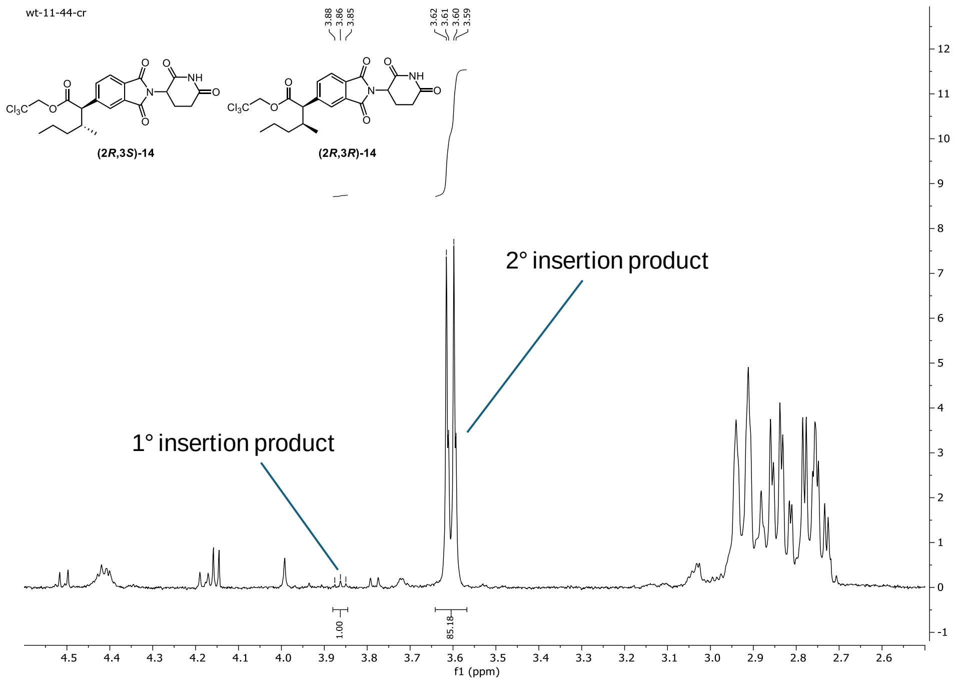

^{1}H NMR spectrum for 14 showing support for d.r. (relative configuration of the two new stereocenters) determination

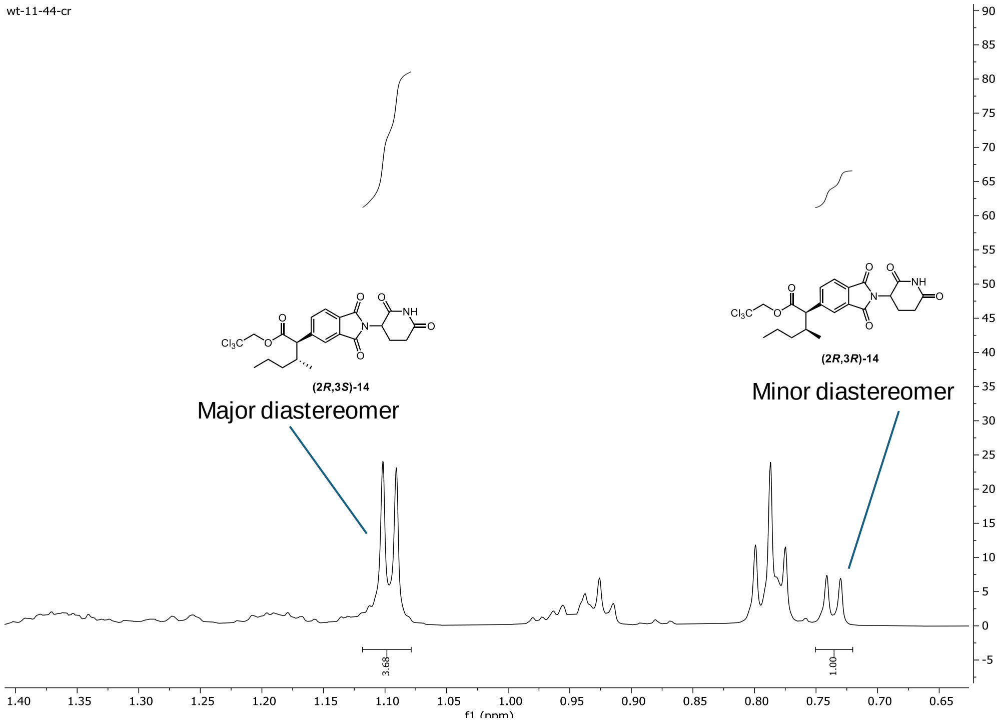

^{1}H NMR spectrum for 19 showing support for r.r. determination, Method A.

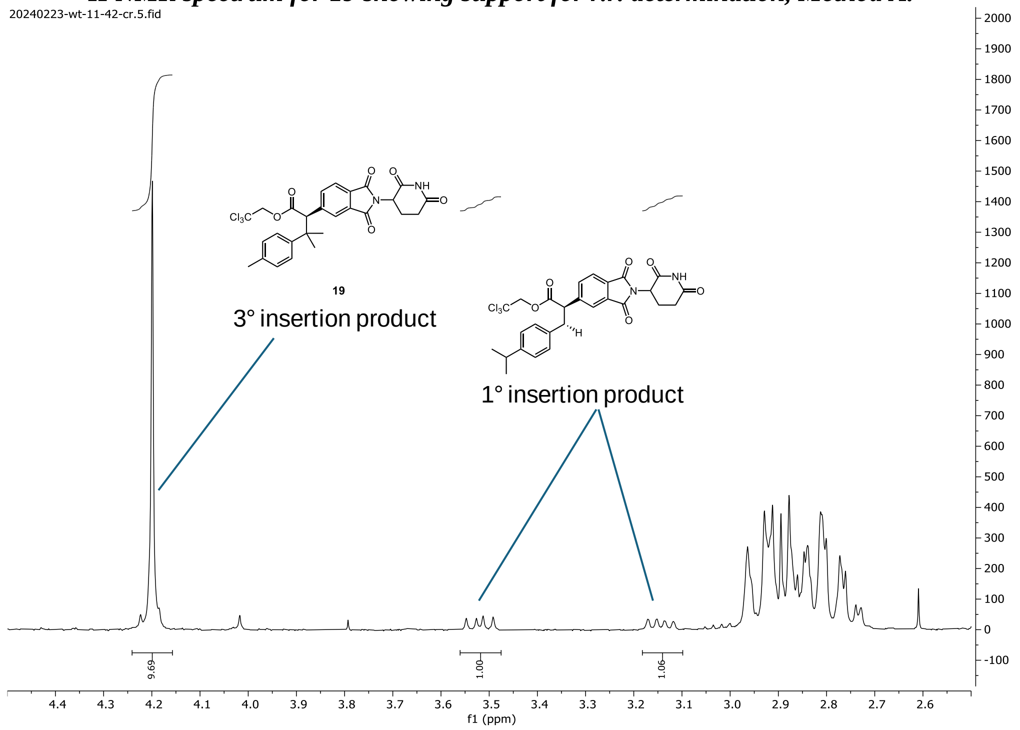

^{1}H NMR spectrum for 19 showing support for r.r. determination, Method B.

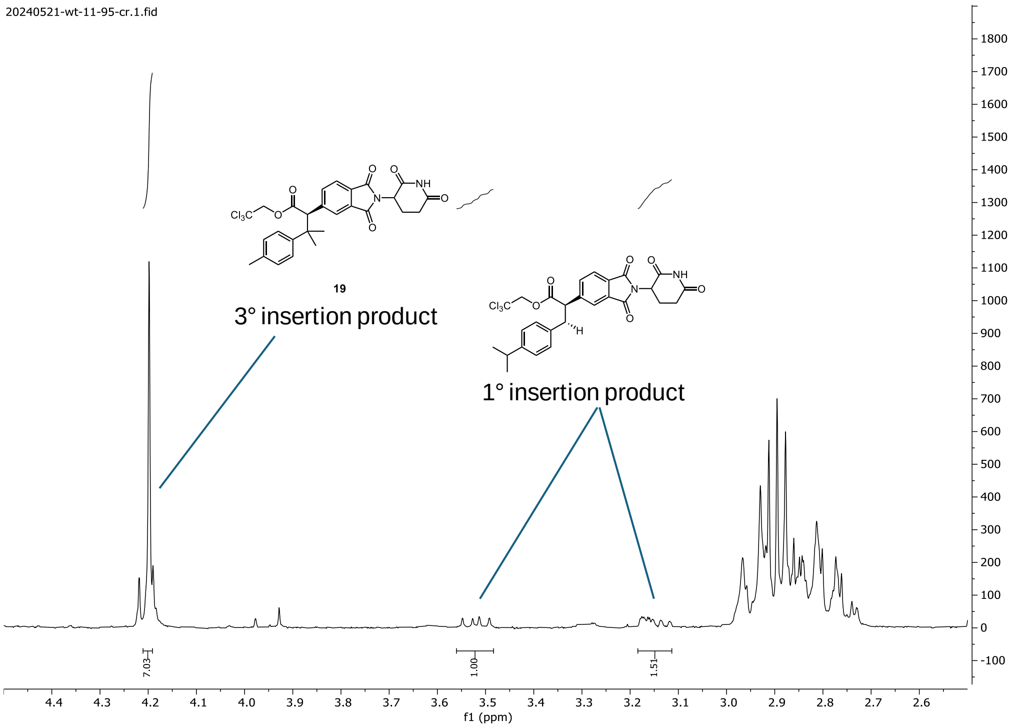

Section 5: Chromatographic Data

Chiral SFC Chromatograms

Racemic Chromatogram for Compound 9a

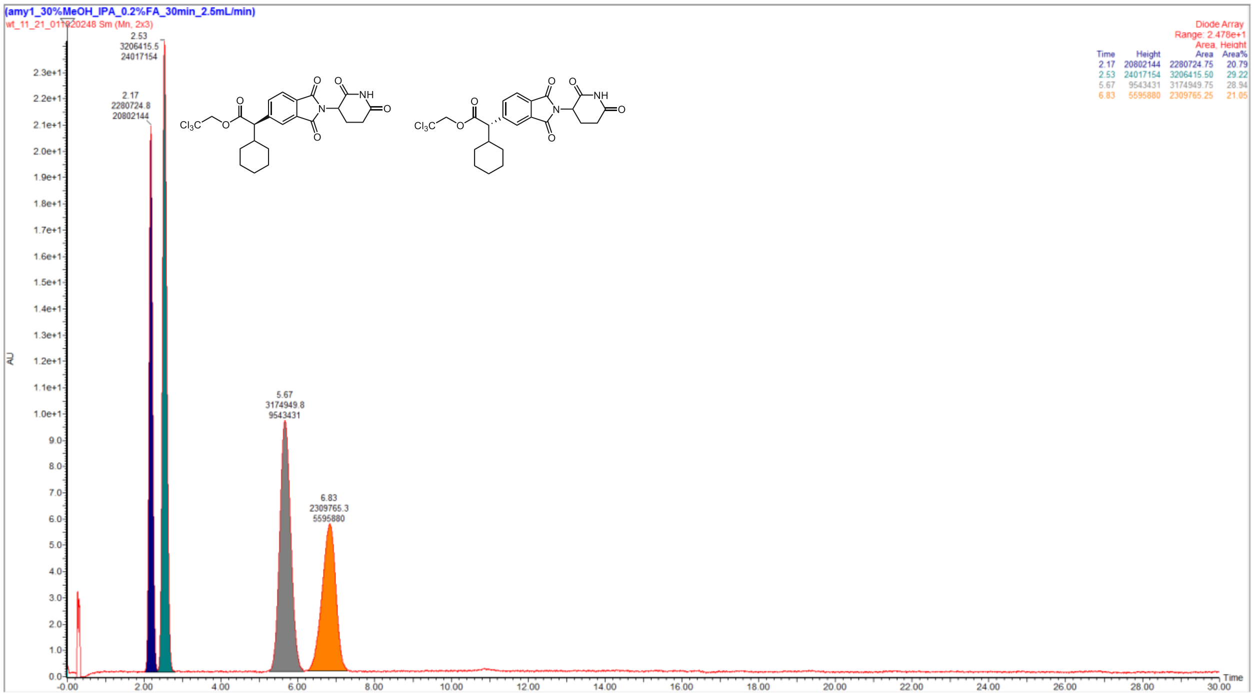

Chromatogram for Compound 9a Using GP1

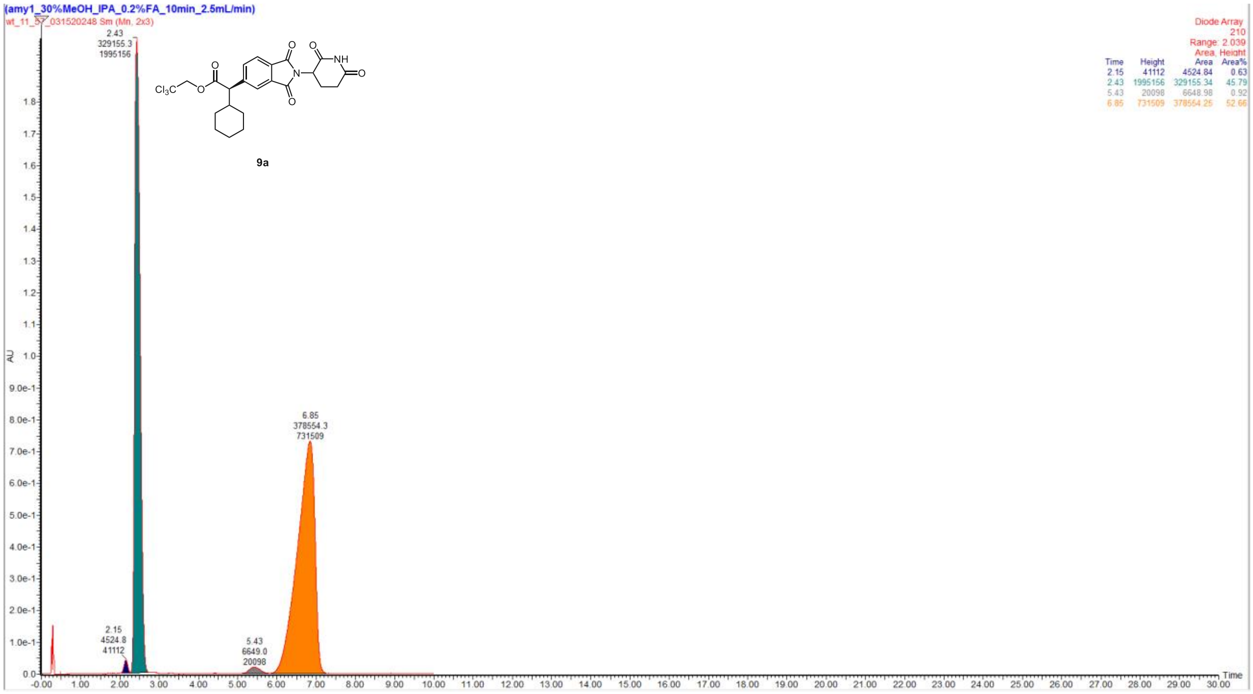

Chromatogram for Compound 9a Using GP2

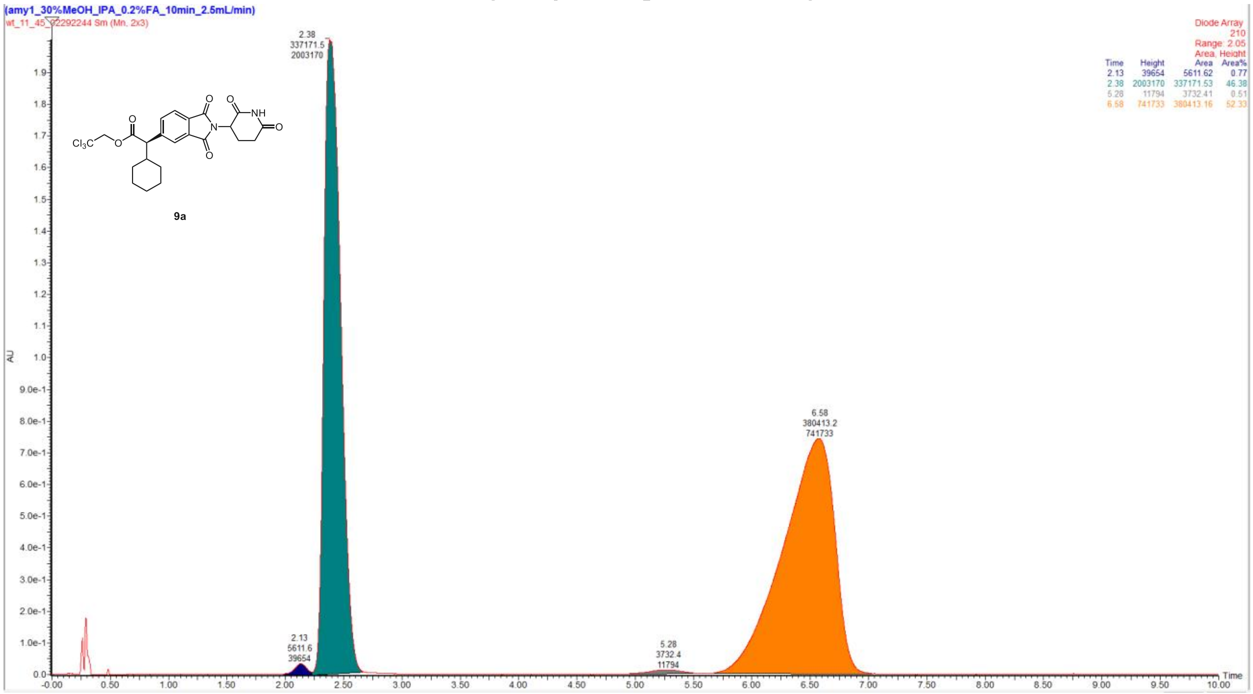

Chromatogram for Compound 9a Using GP3

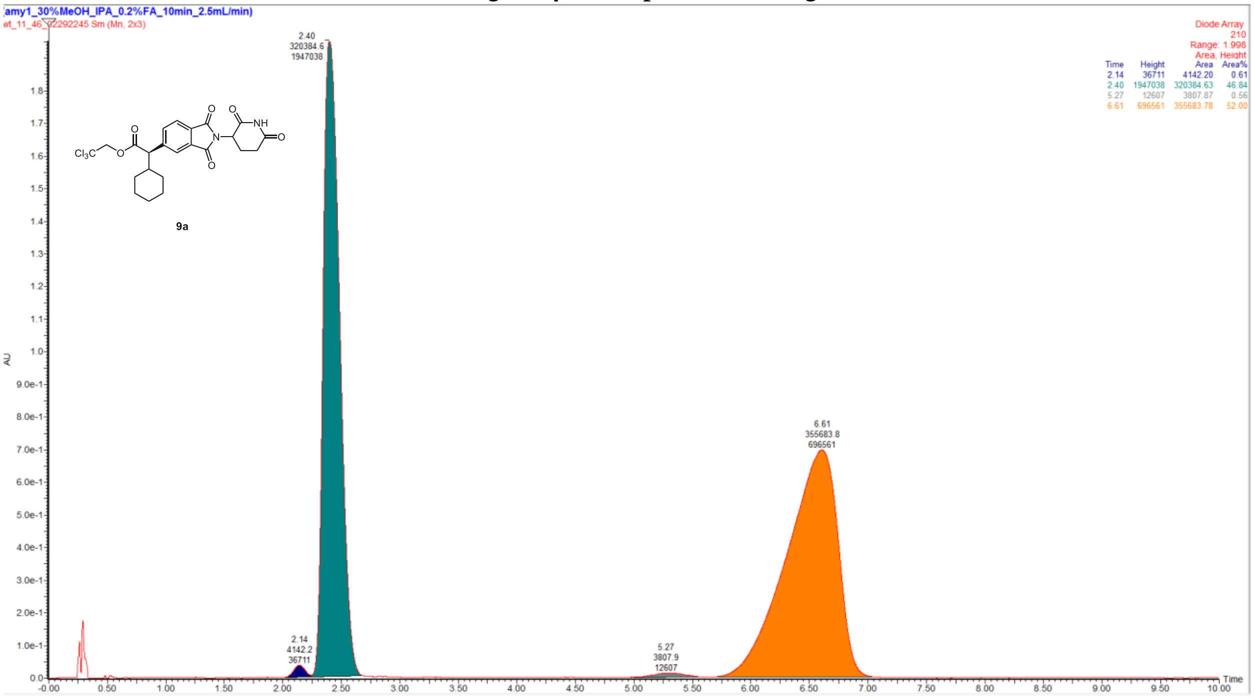

Racemic Chromatogram for Compound 9b

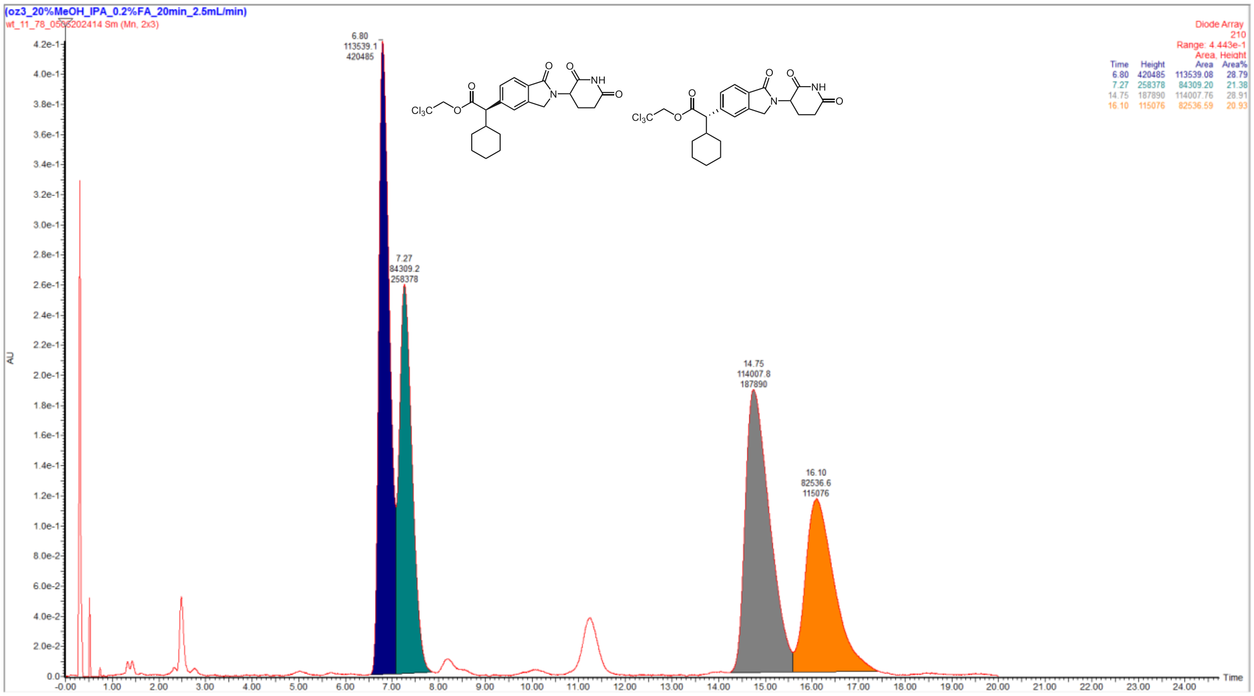

Chromatogram for Compound 9b Using GP1

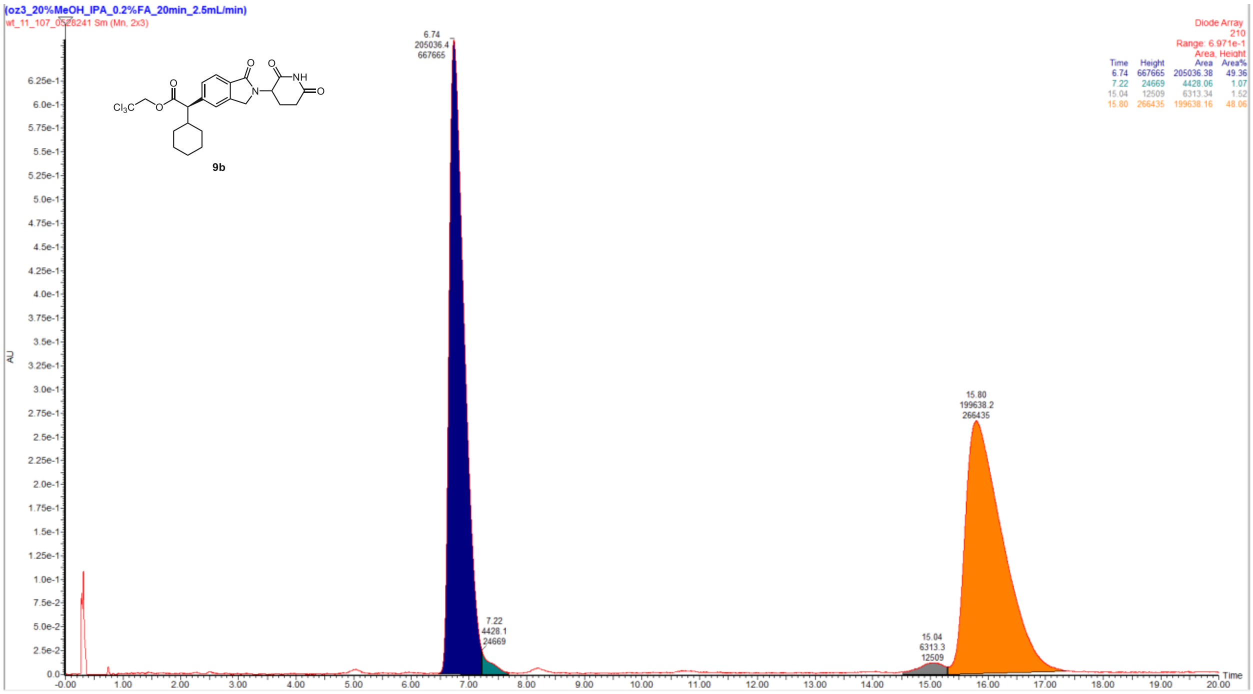

Chromatogram for Compound 9b Using GP3

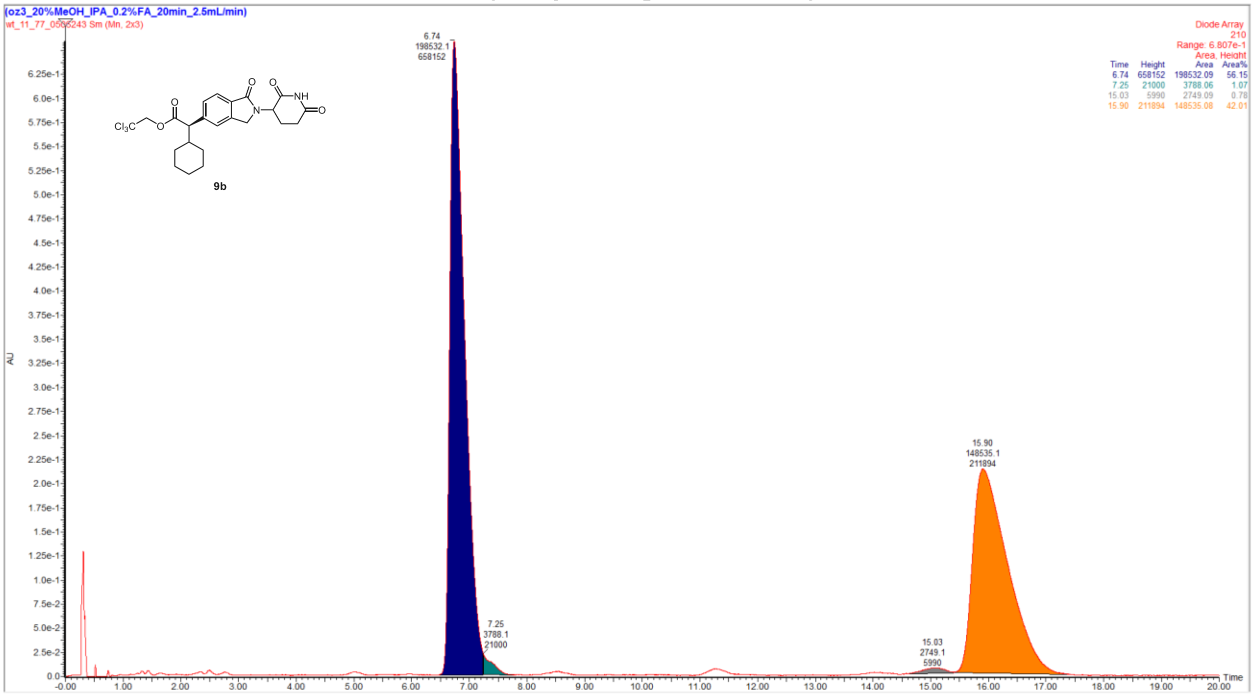

Racemic Chromatogram for Compound 9c

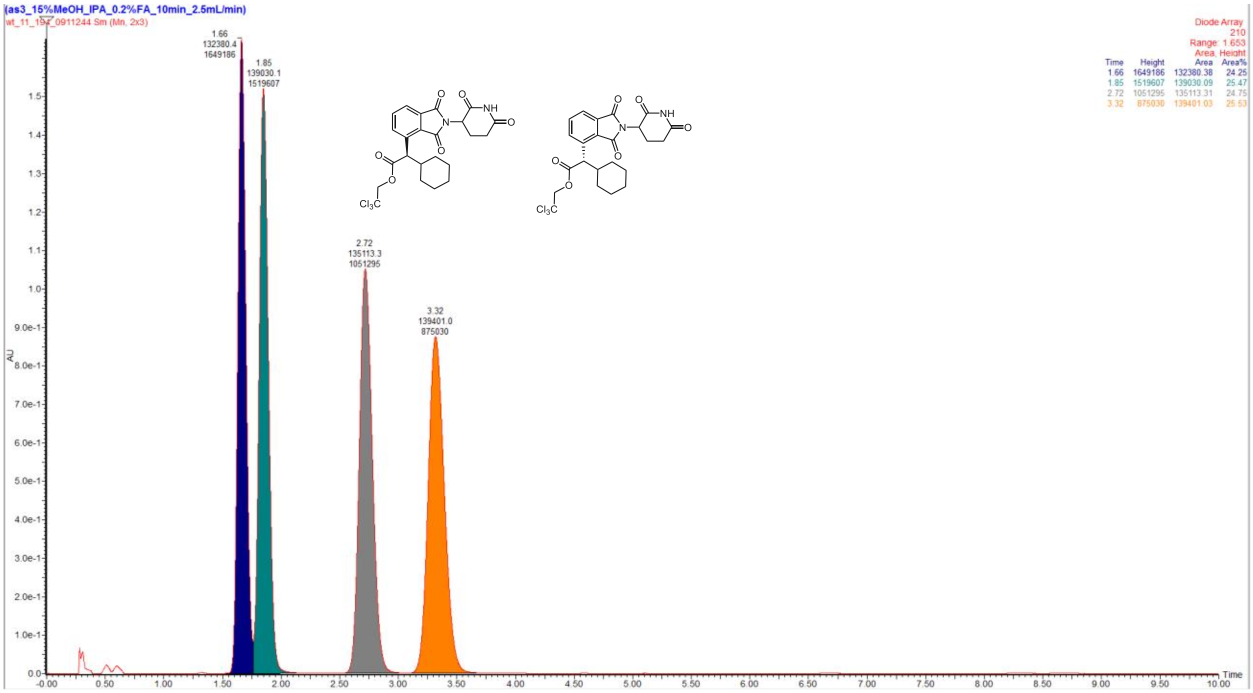

Chromatogram for Compound 9c Using GP1

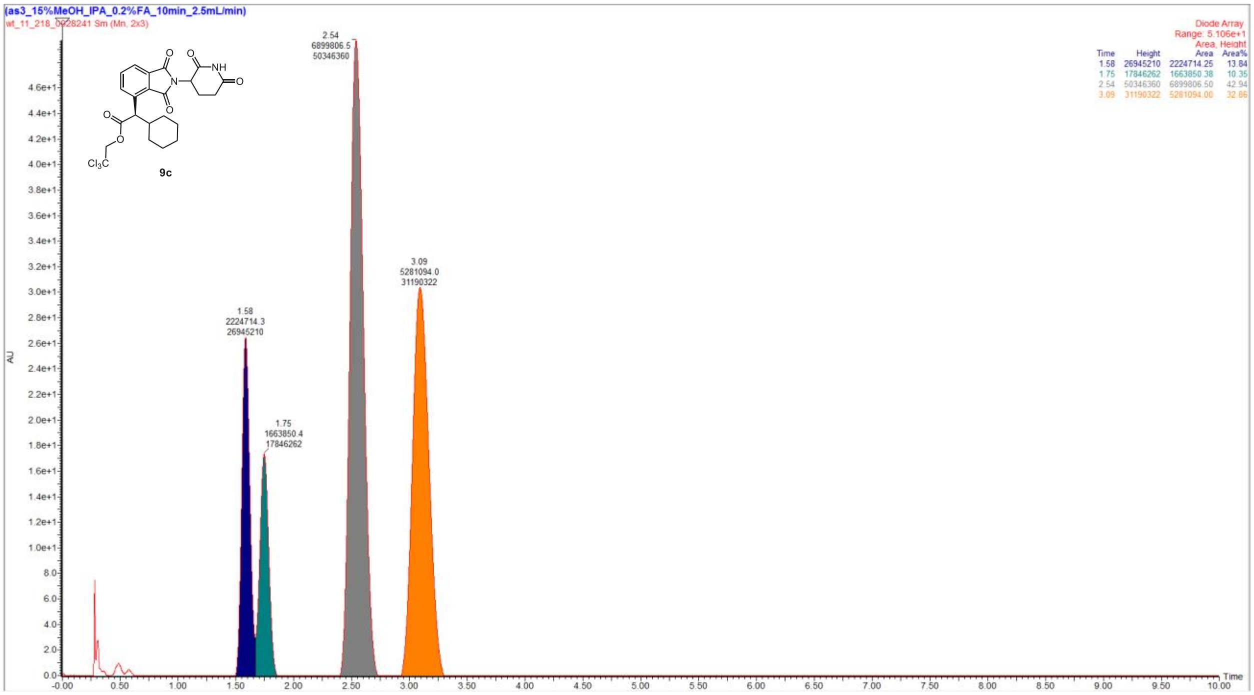

Chromatogram for Compound 9c Using GP3

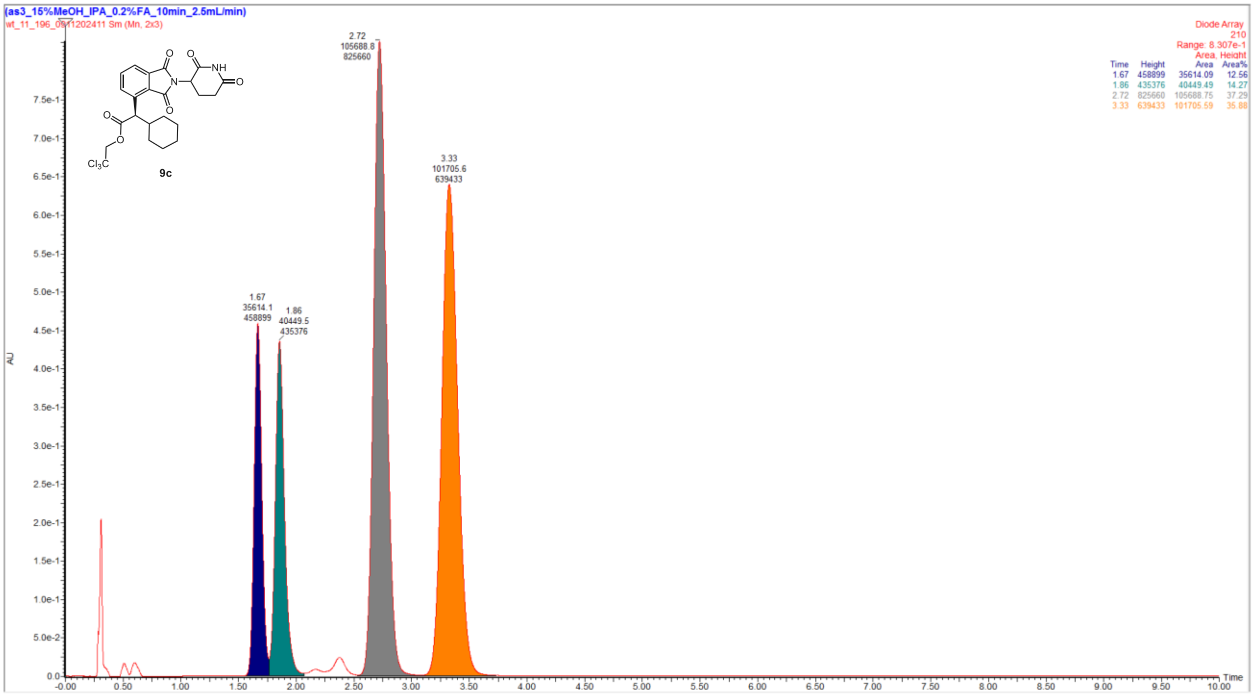

Racemic Chromatogram for Compound 10a

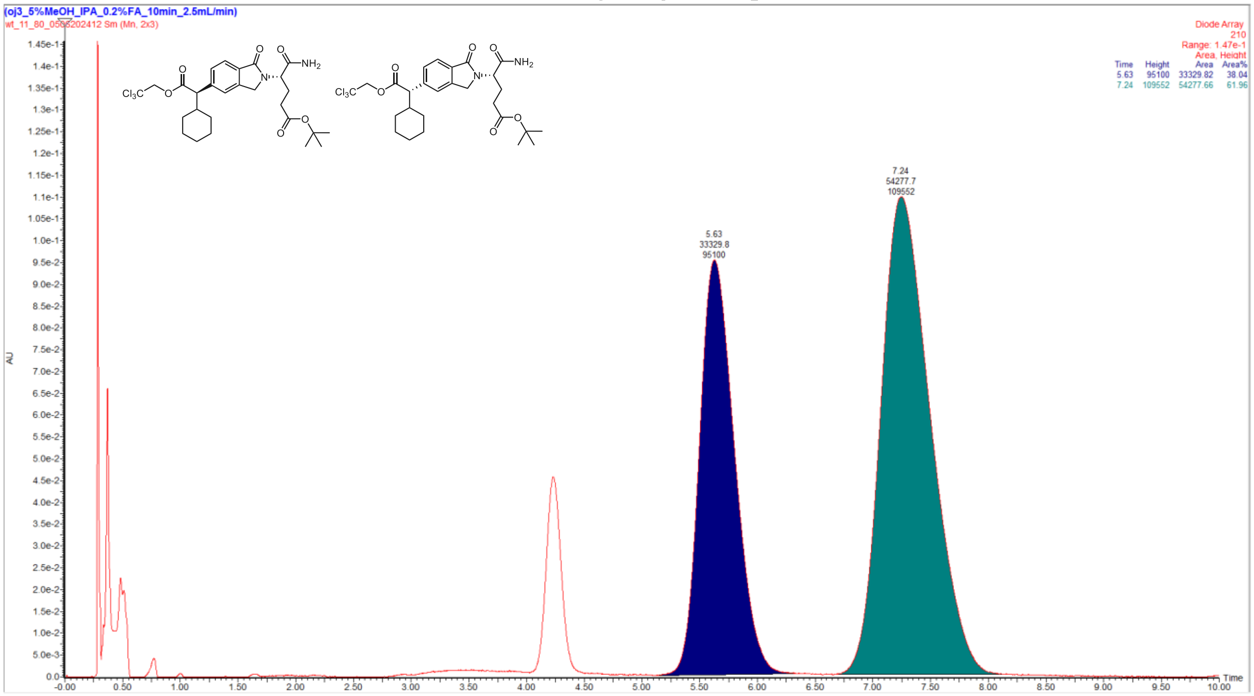

Chromatogram for Compound (S,R)-10a Using GP1

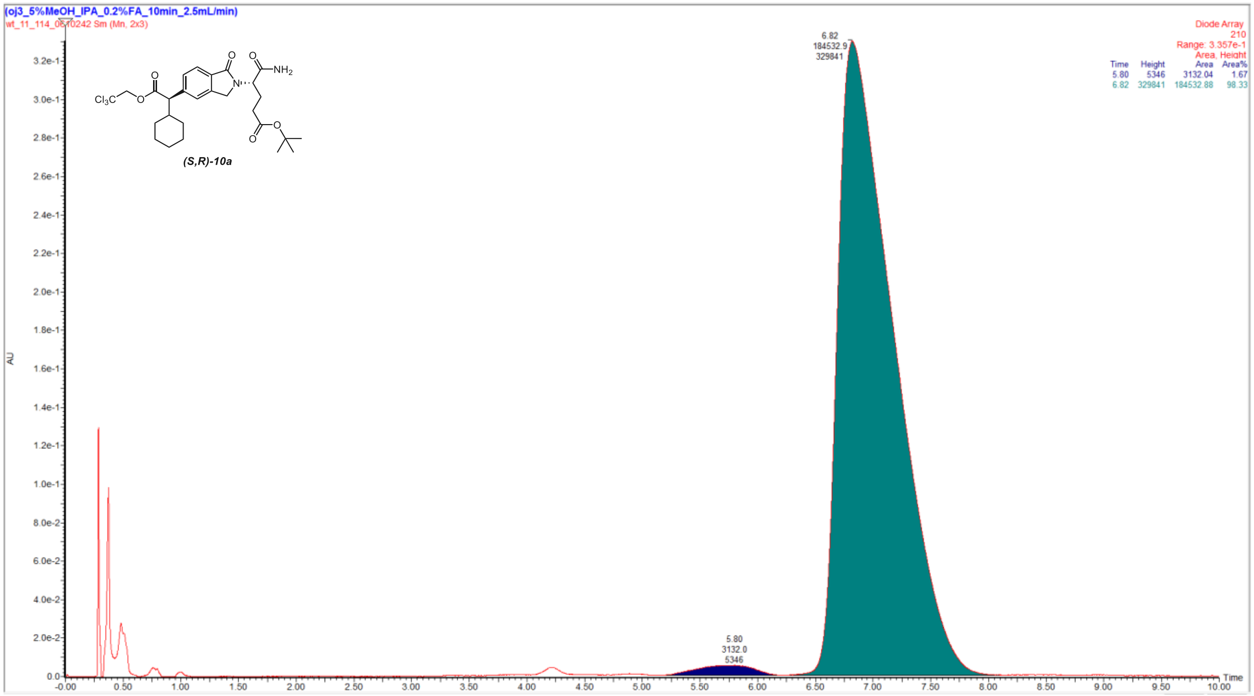

Chromatogram for Compound (S,R)-10a Using GP4

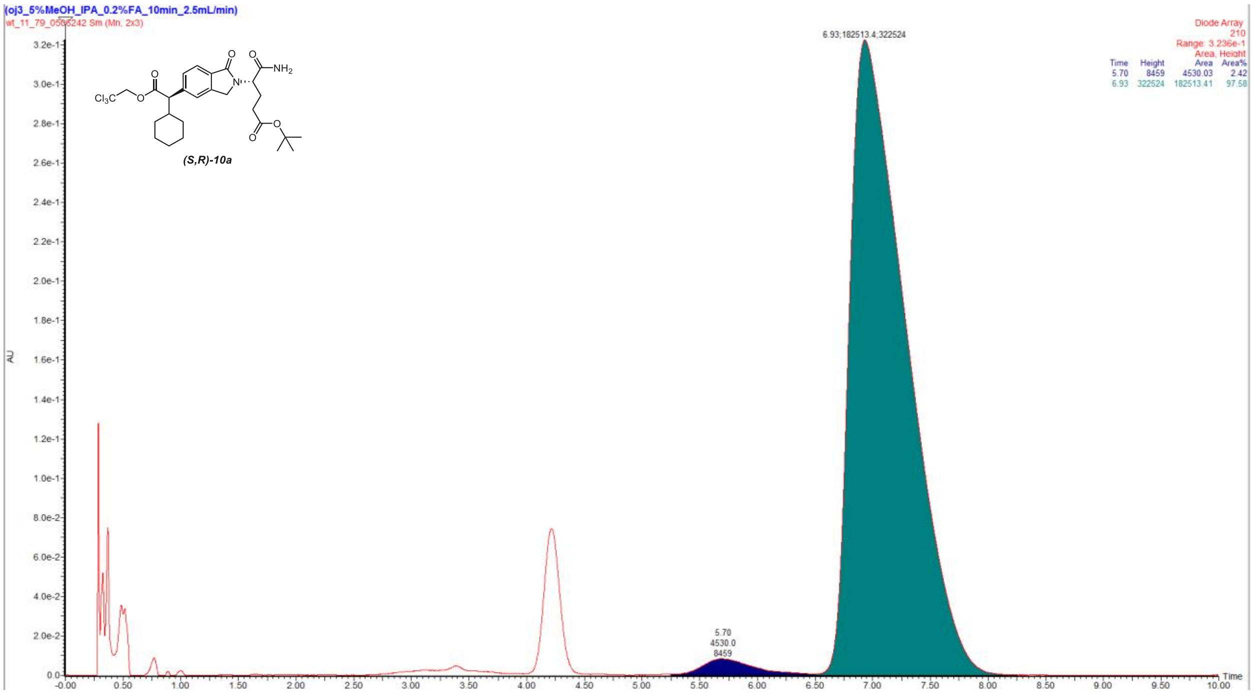

Chromatogram for Compound (S,S)-10a Using GP4

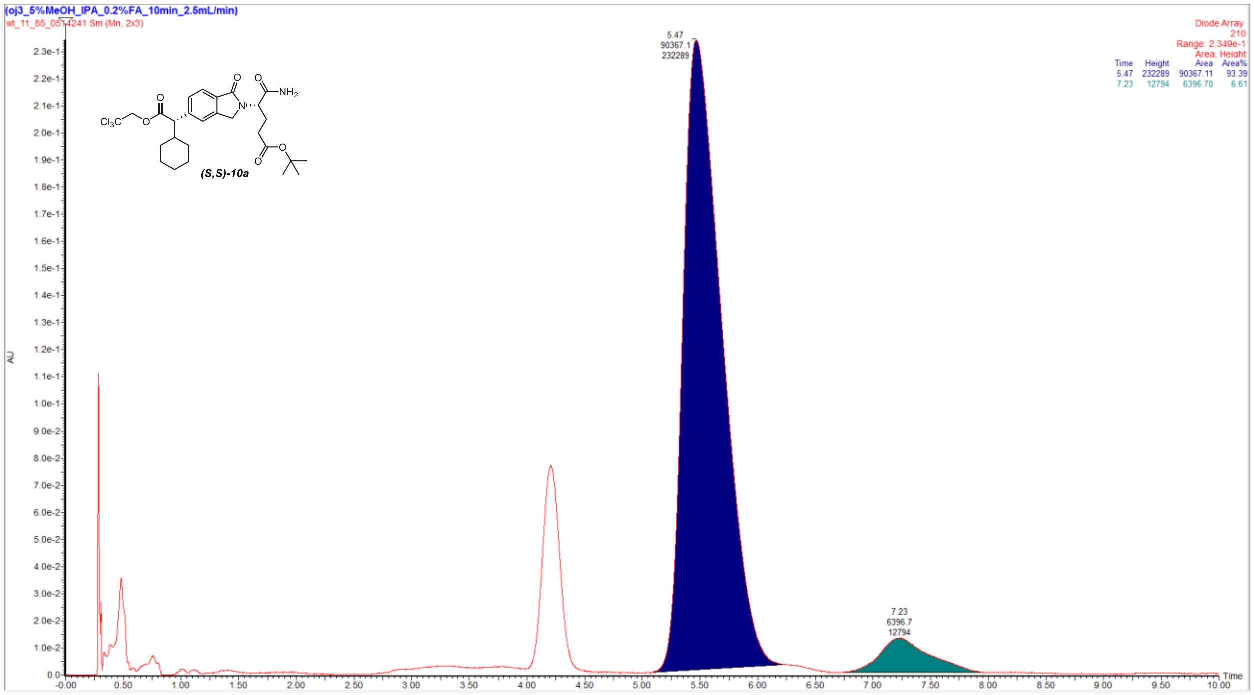

Racemic Chromatogram for Compound 10b

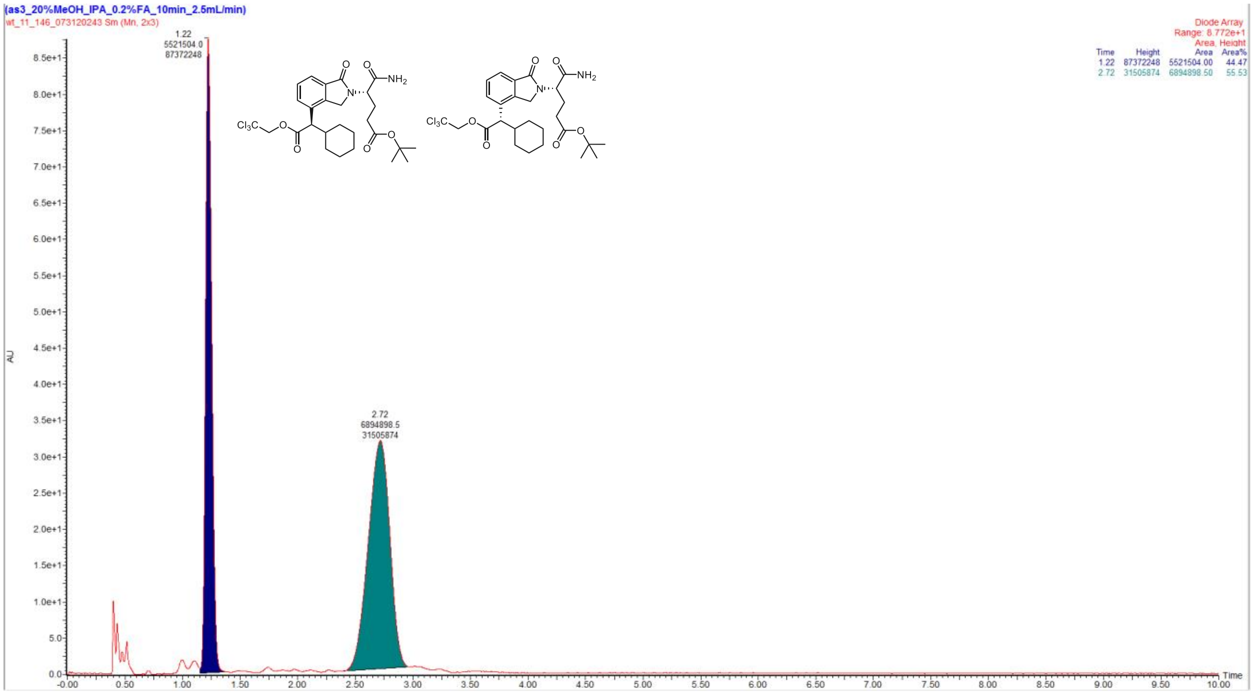

Chromatogram for Compound 10b Using GP1

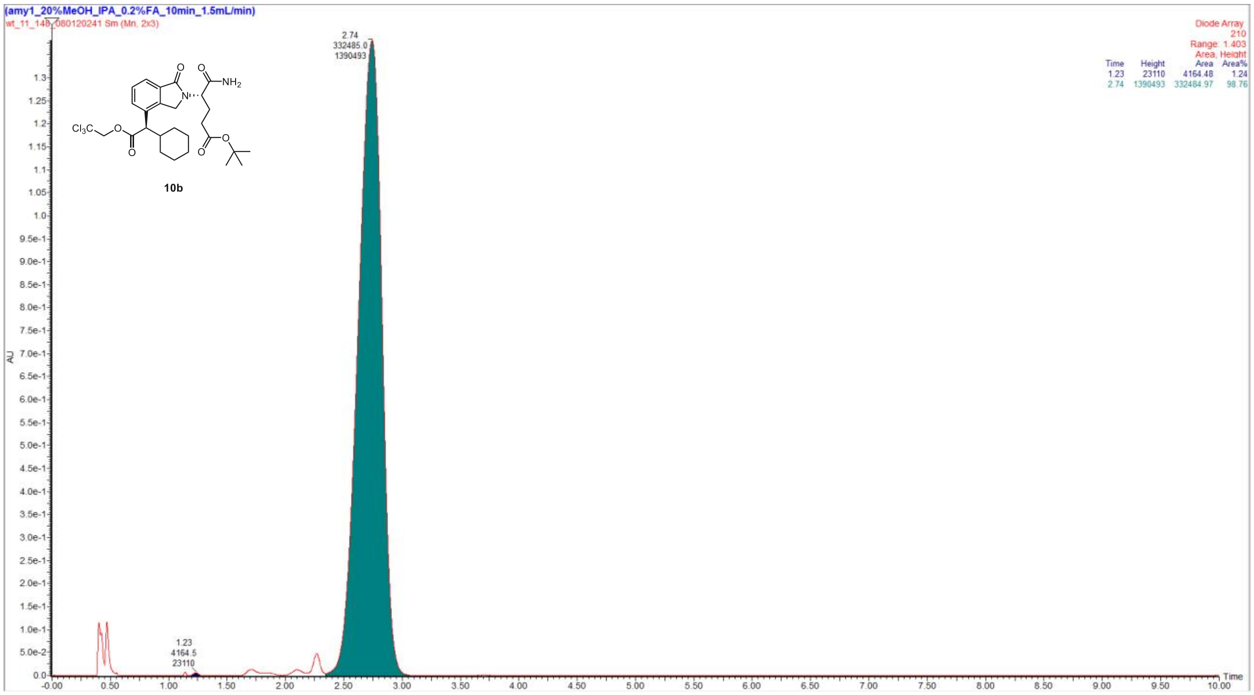

Chromatogram for Compound 10b Using GP4

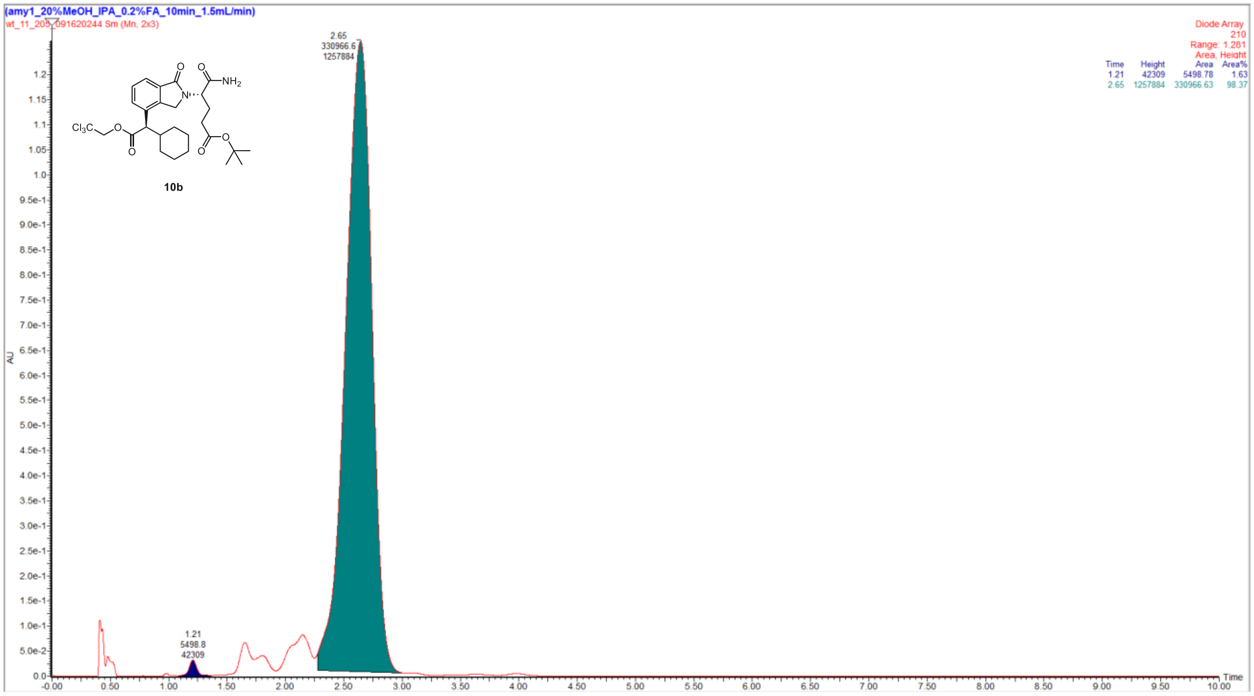

Racemic Chromatogram for Compound 11a

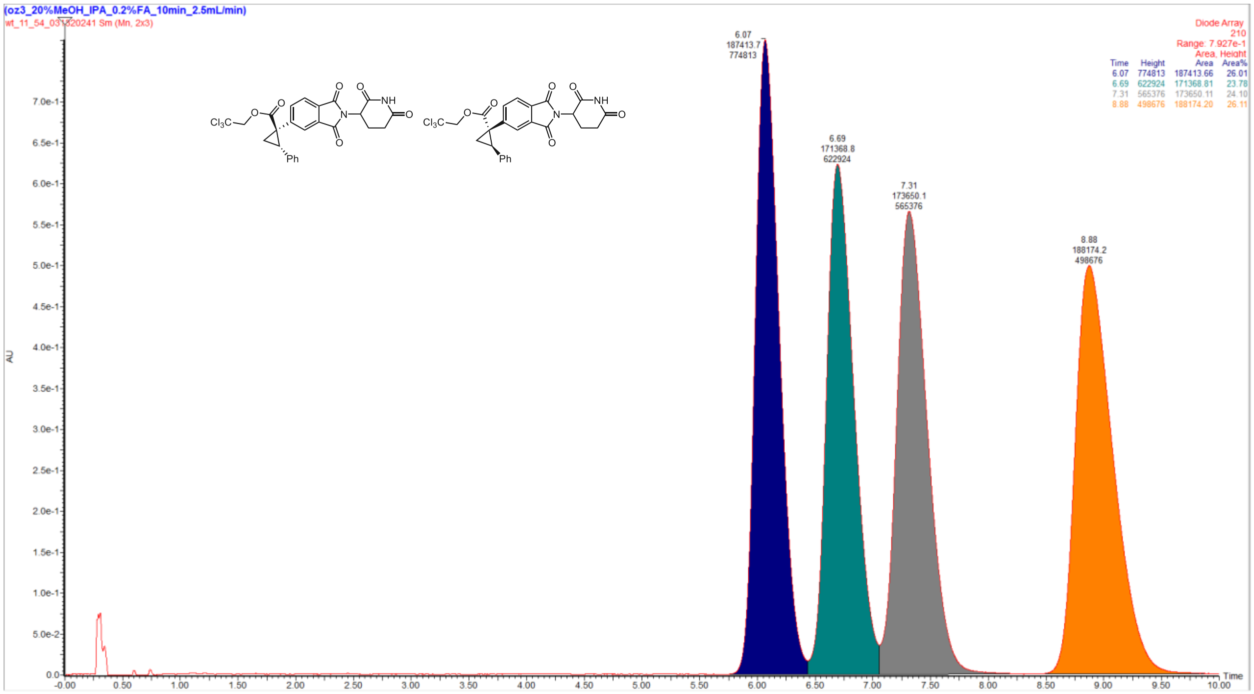

Chromatogram for Compound 11a Using GP5

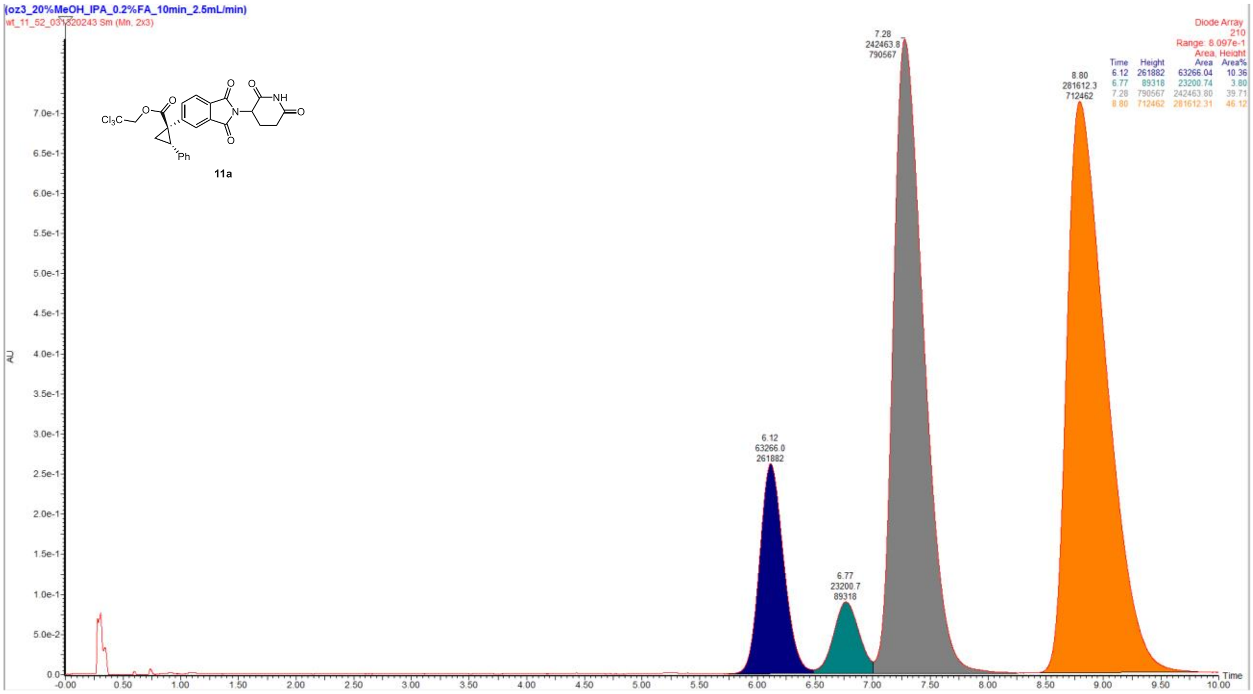

Racemic Chromatogram for Compound 11b

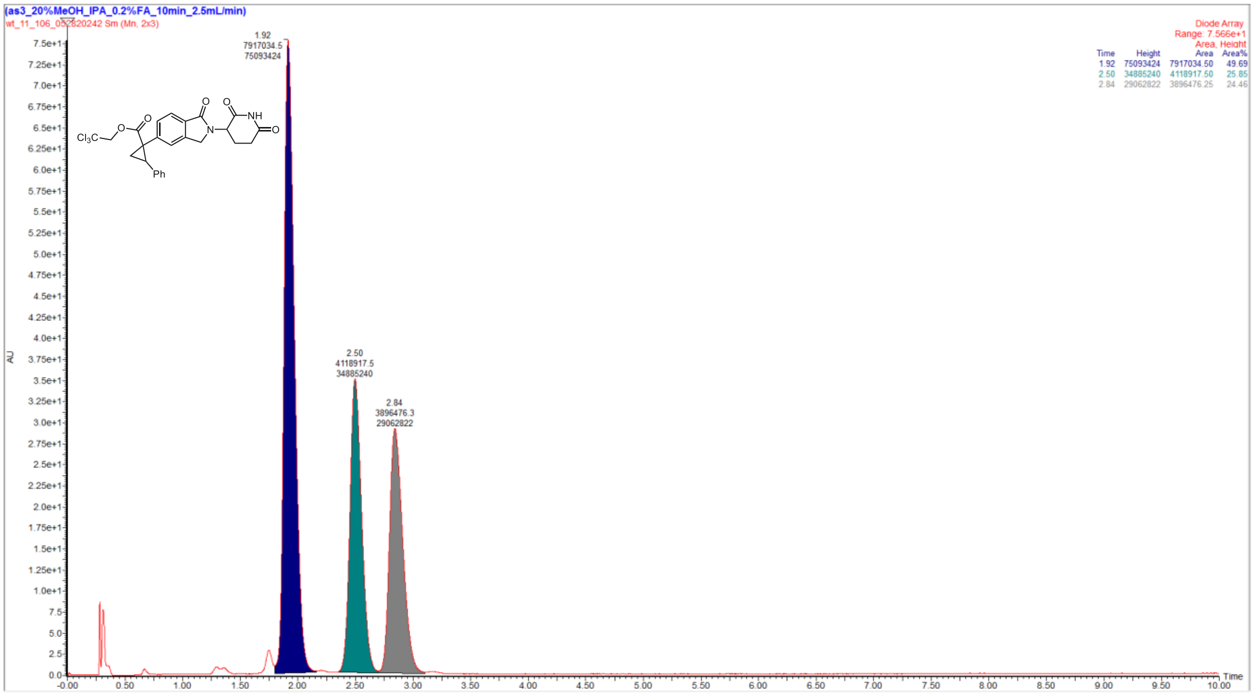

Chromatogram for Compound 11b Using GP5

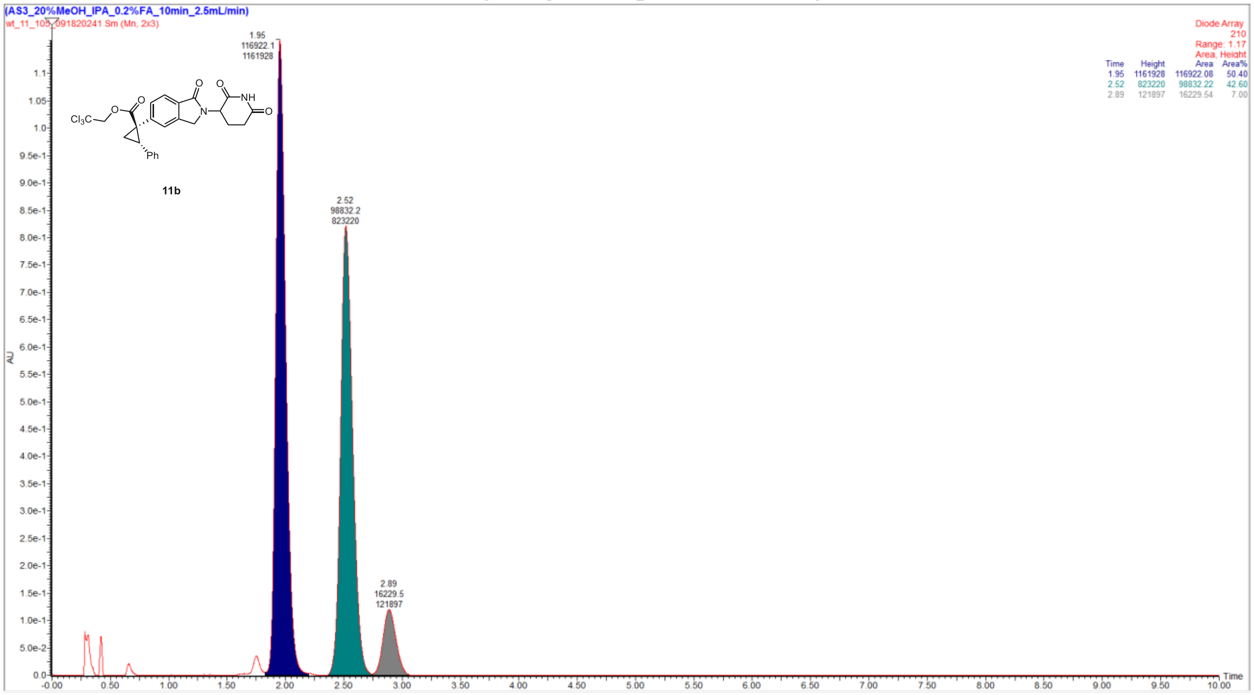

Racemic Chromatogram for Compound 11c

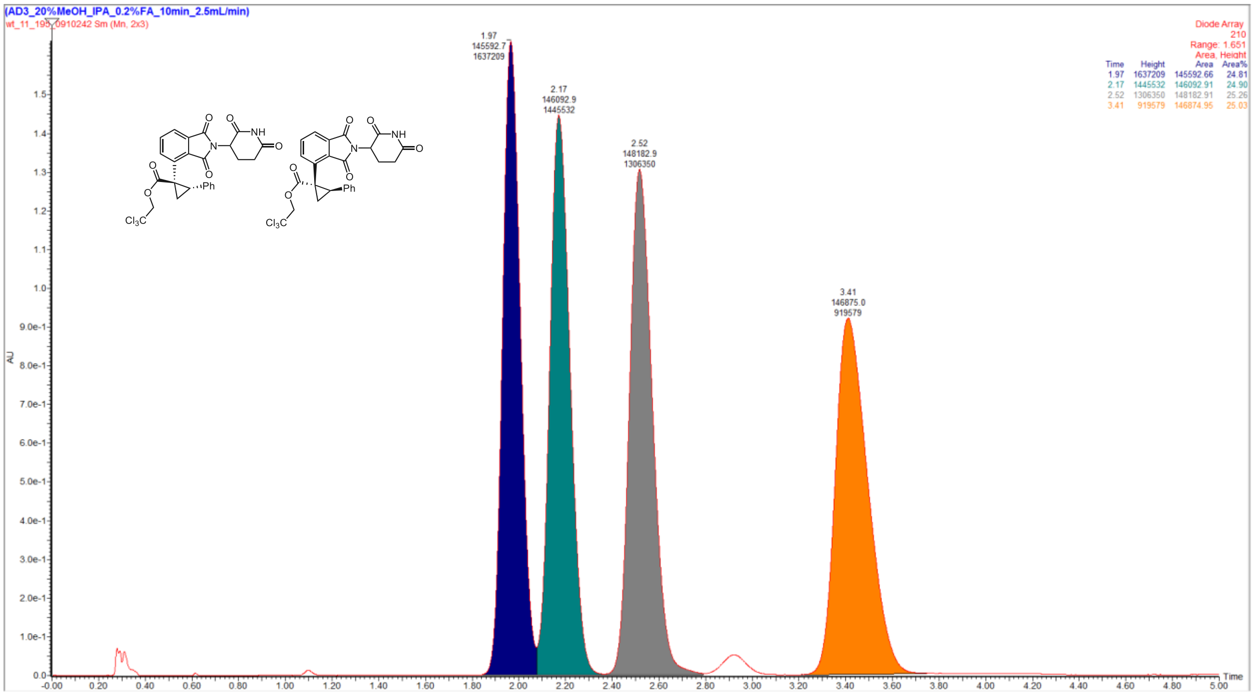

Chromatogram for Compound 11c Using GP5

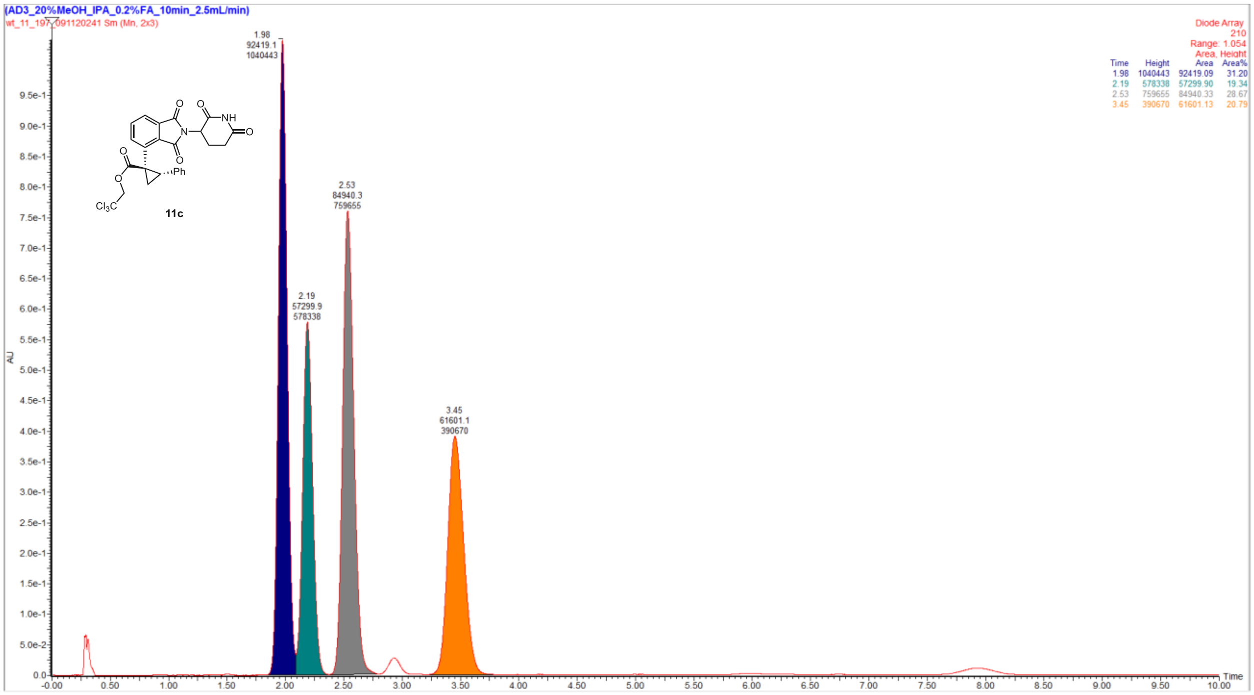

Racemic Chromatogram for Compound 12a

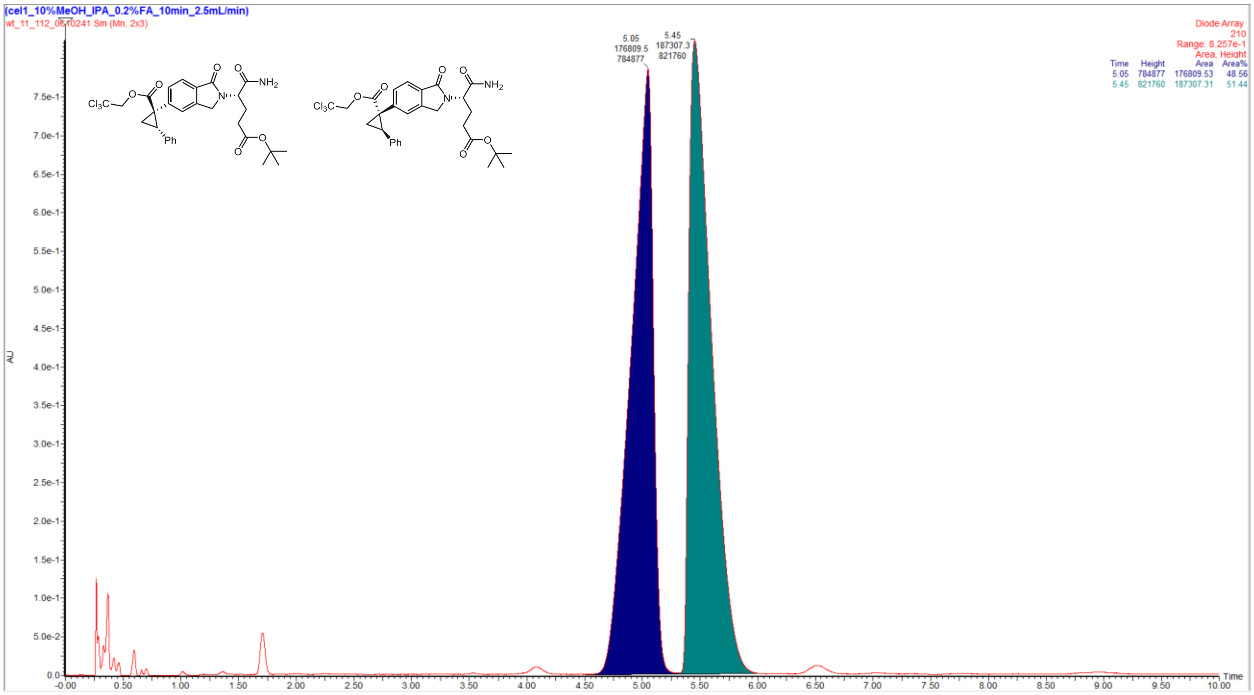

Chromatogram for Compound 12a Using GP6

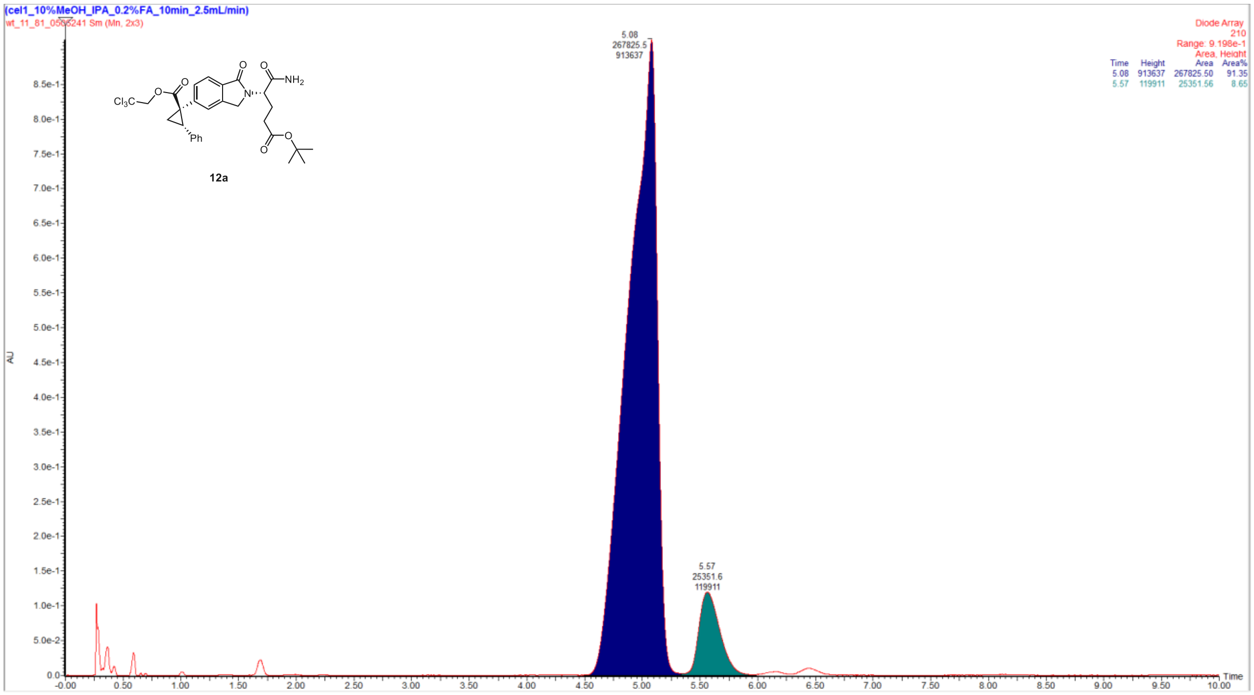

Racemic Chromatogram for Compound 12b

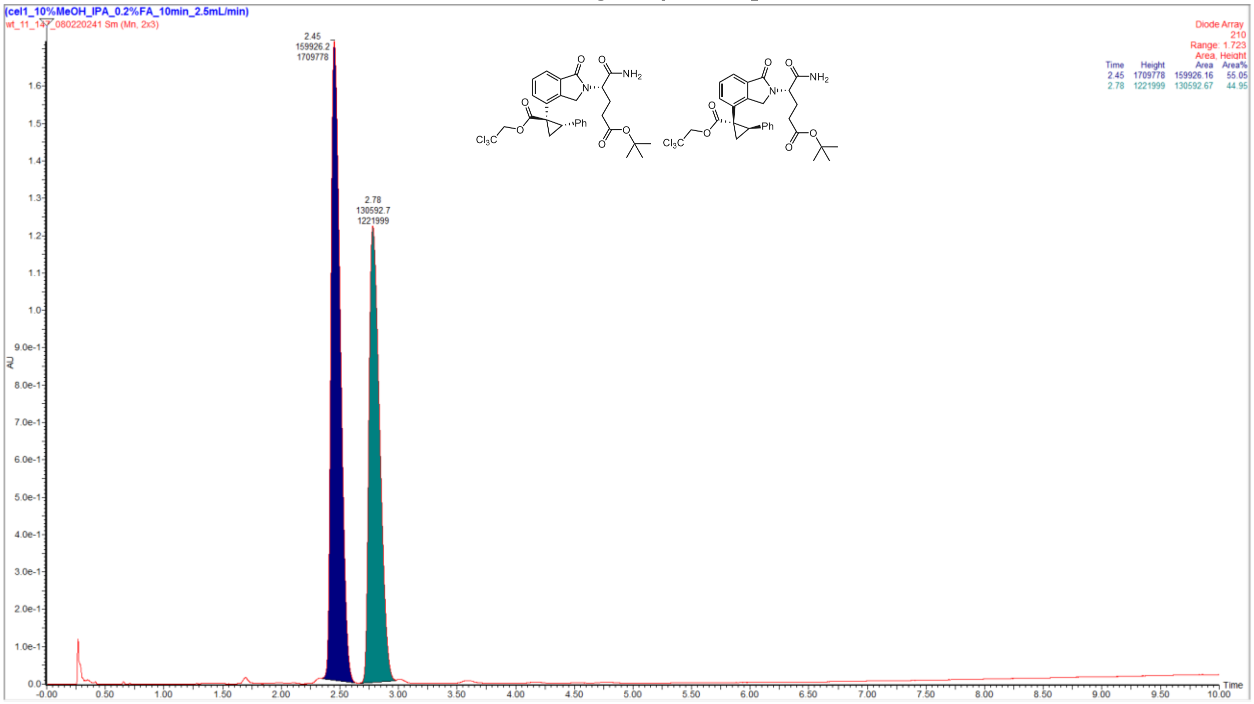

Chromatogram for Compound 12b Using GP6

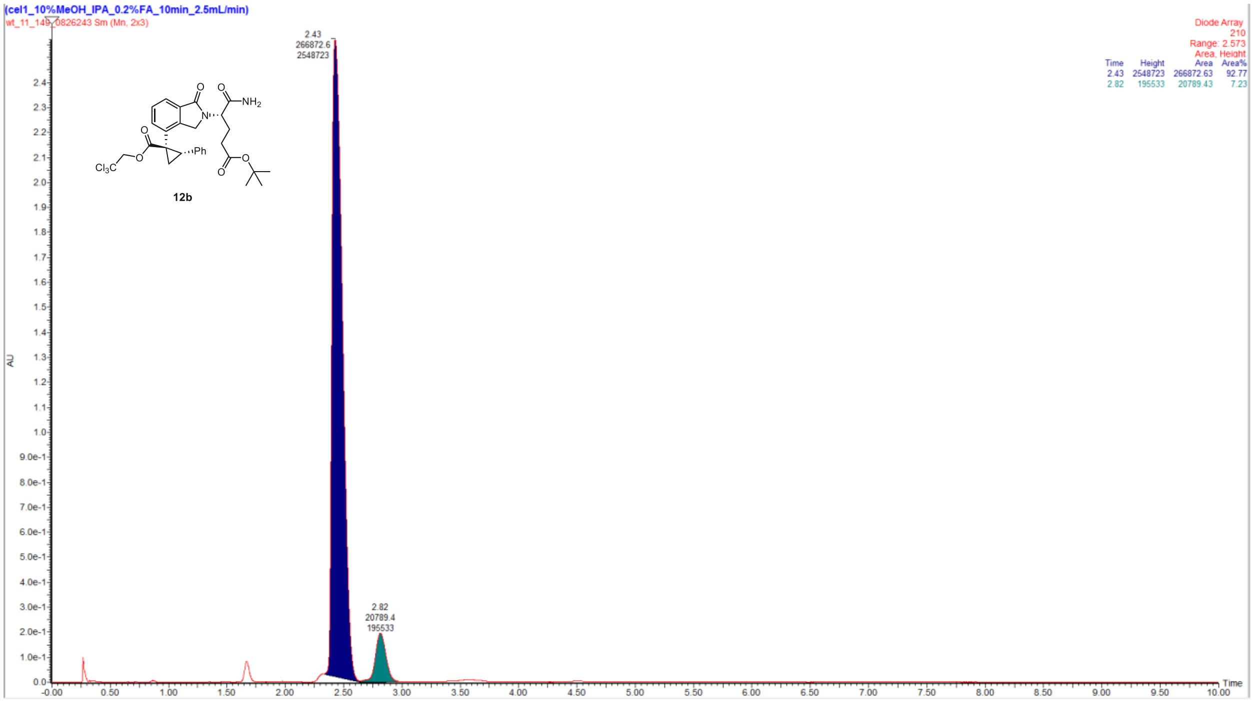

Racemic Chromatogram for Compound 13

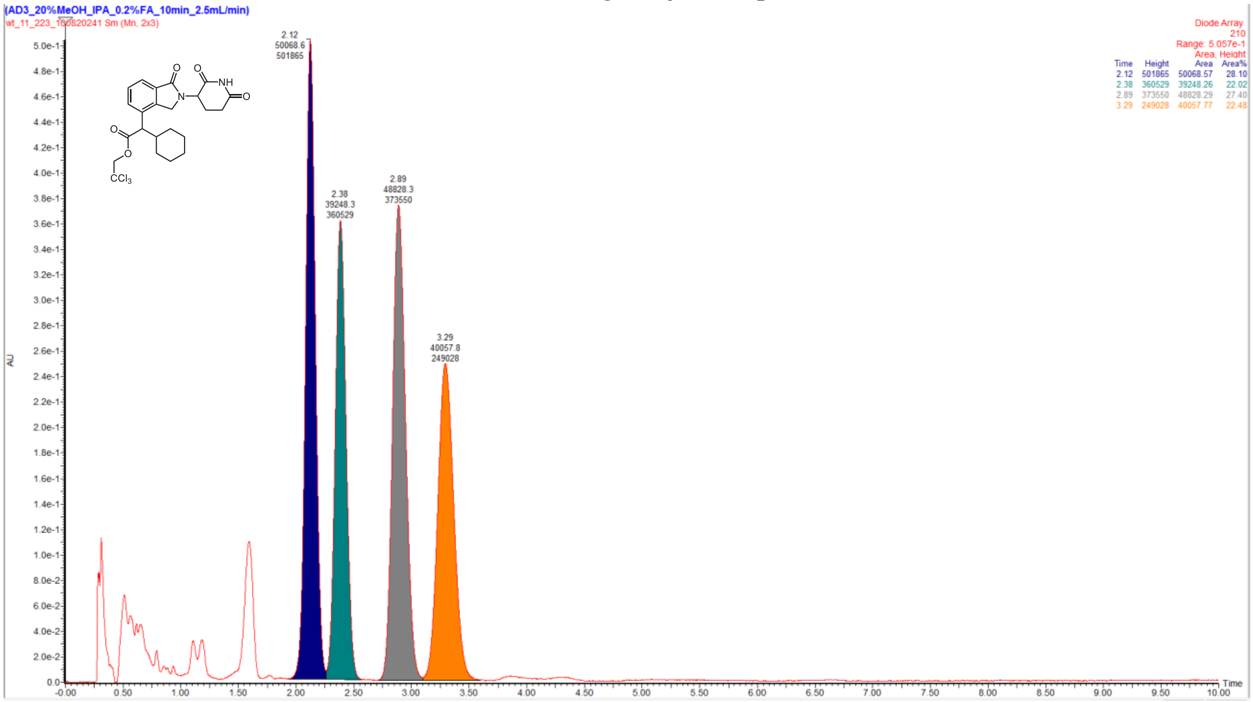

Chromatogram for Compound 13

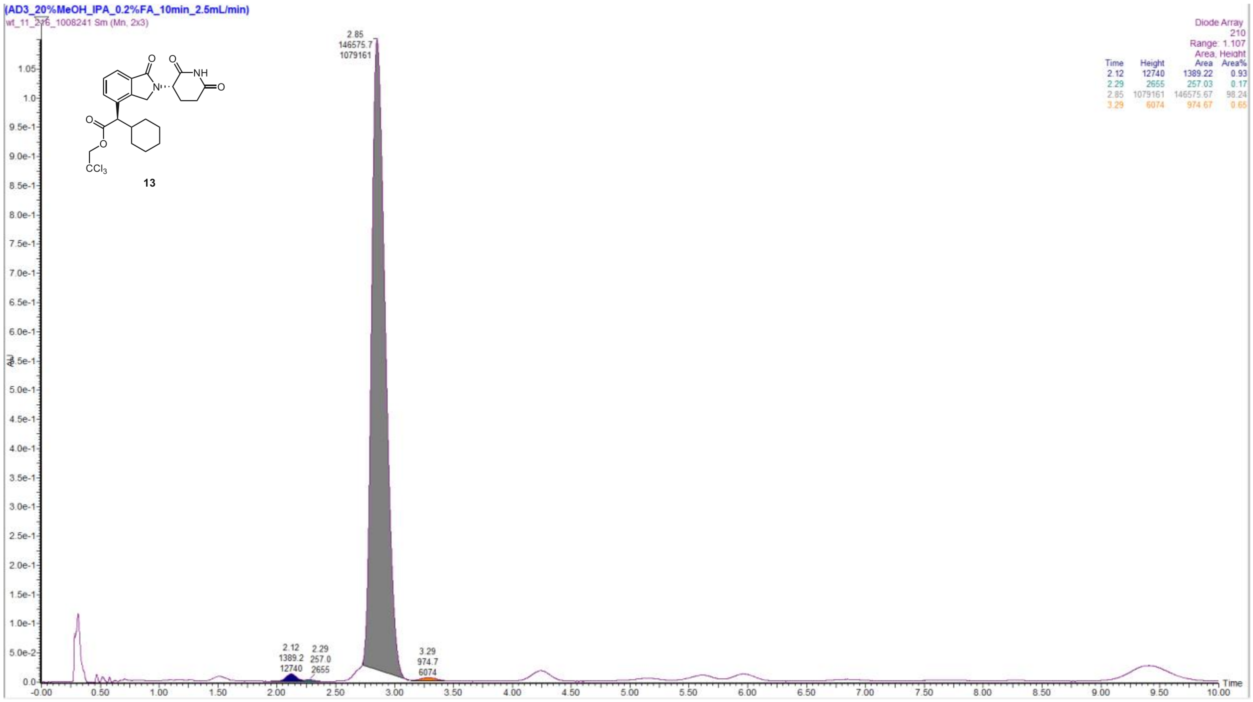

Racemic Chromatogram for Compound 14 and Chromatogram for Compound 14 Using GP1, Major Relative Diastereomer (2R,3S)-14

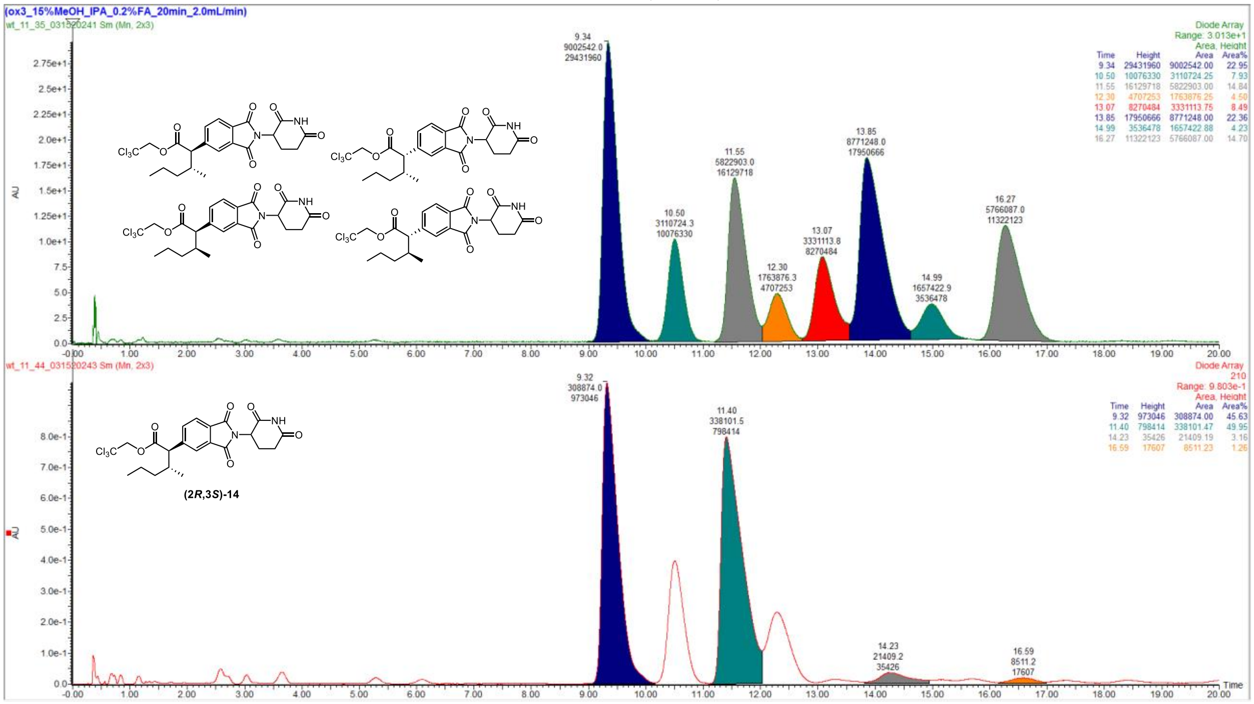

Racemic Chromatogram for Compound 14 and Chromatogram for Compound 14 Using GP1, Minor Relative Diastereomer (2R,3R)-14

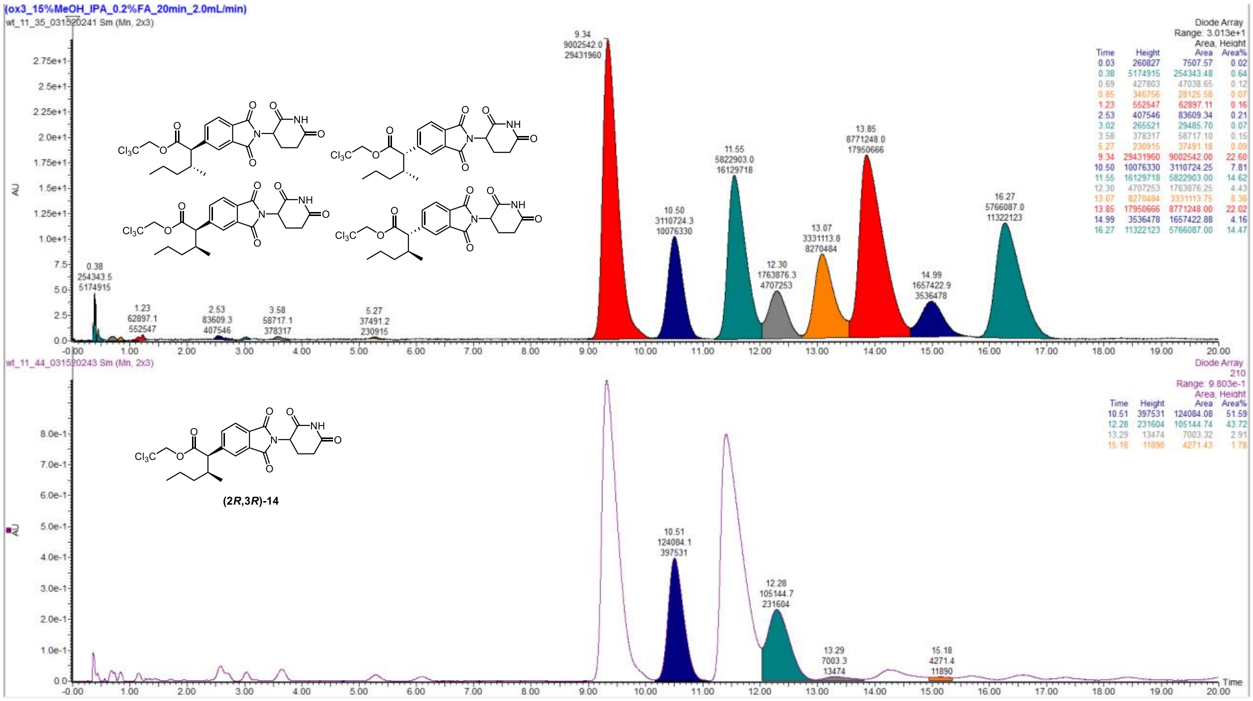

Racemic Chromatogram for Compound 15

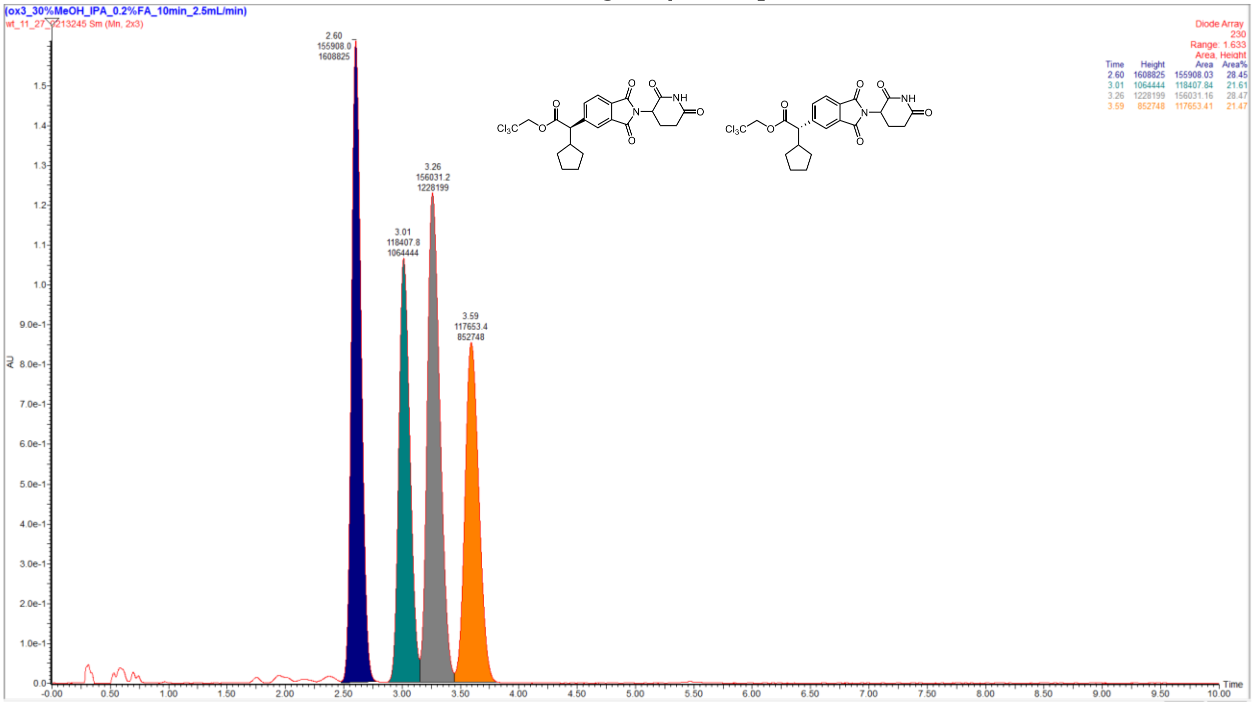

Chromatogram for Compound 15 Using GP1

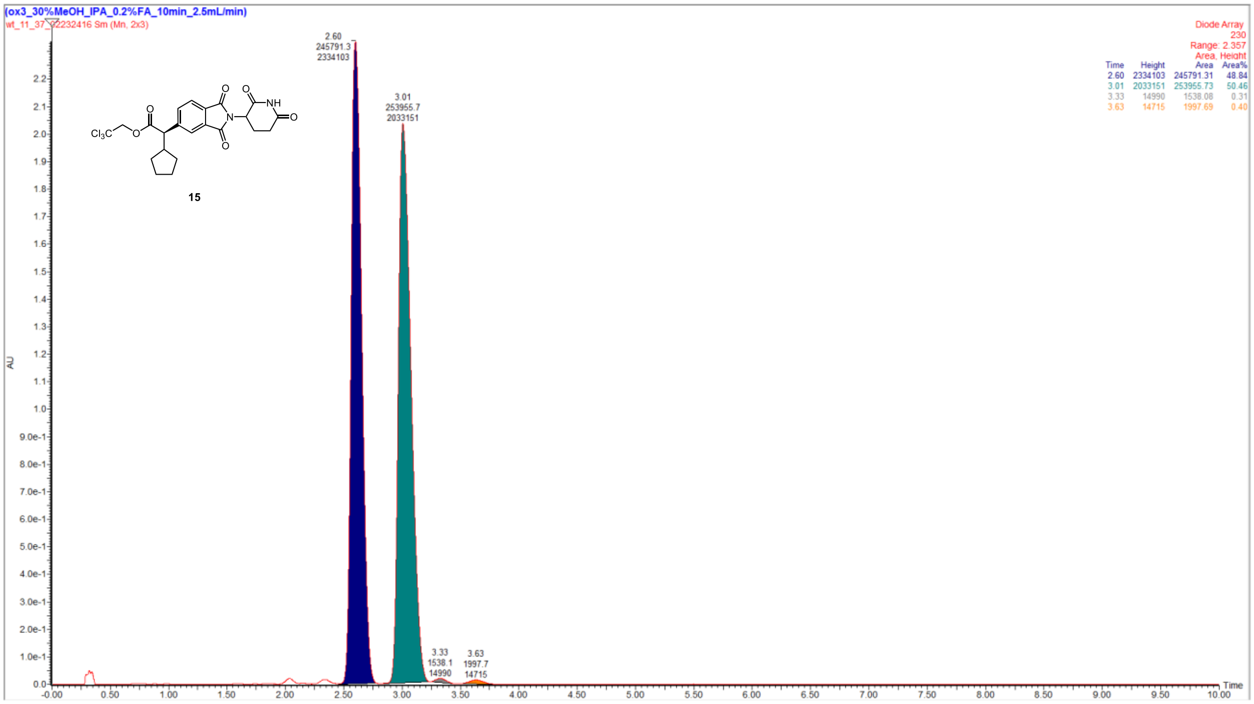

Racemic Chromatogram for Compound 16

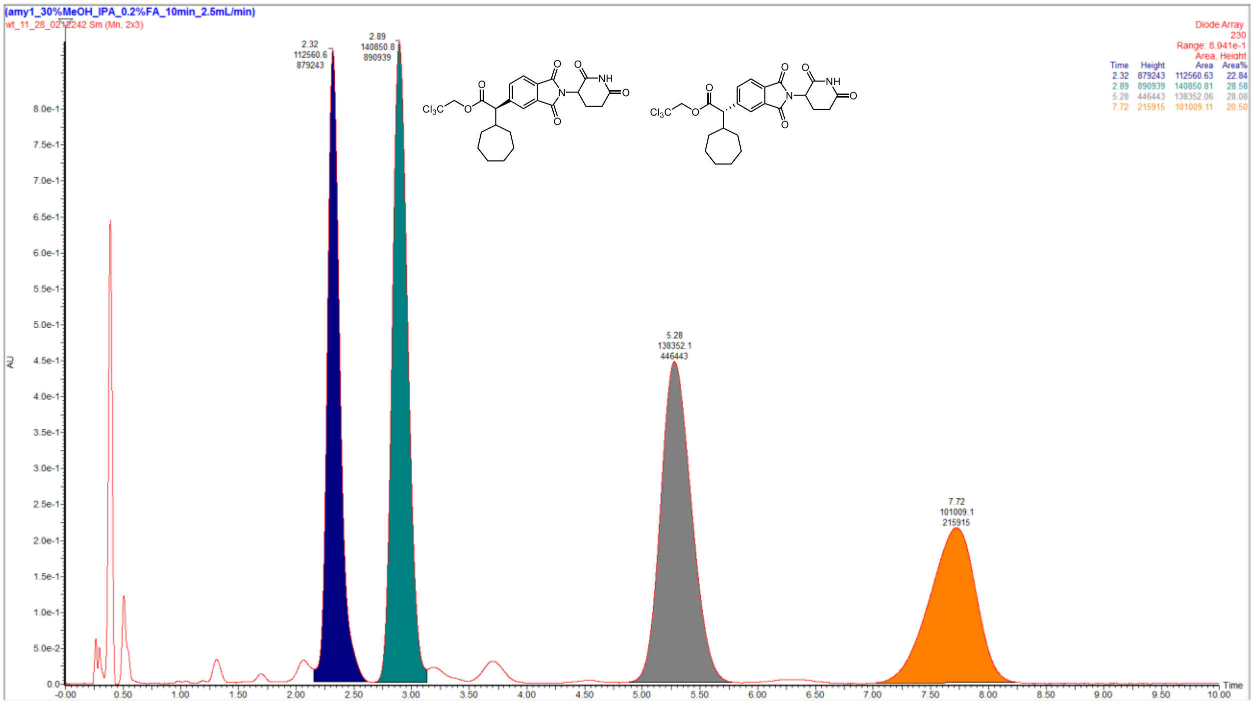

Chromatogram for Compound 16 Using GP1

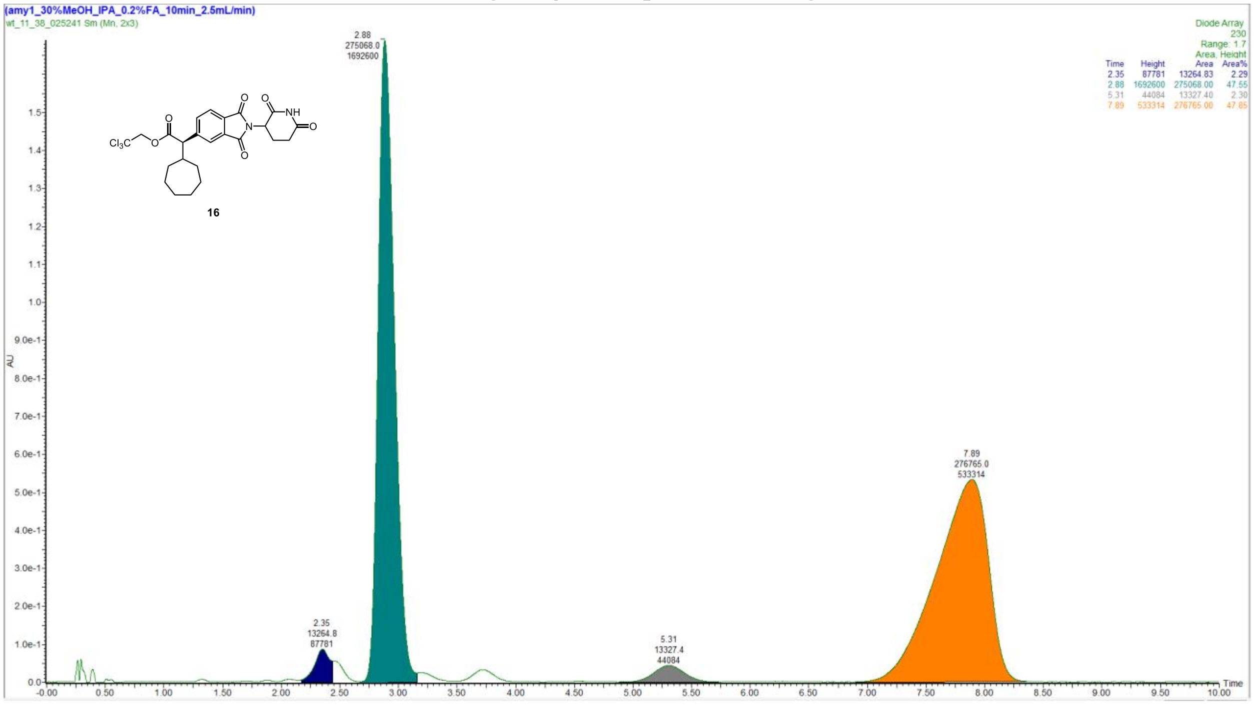

Racemic Chromatogram for Compound 17

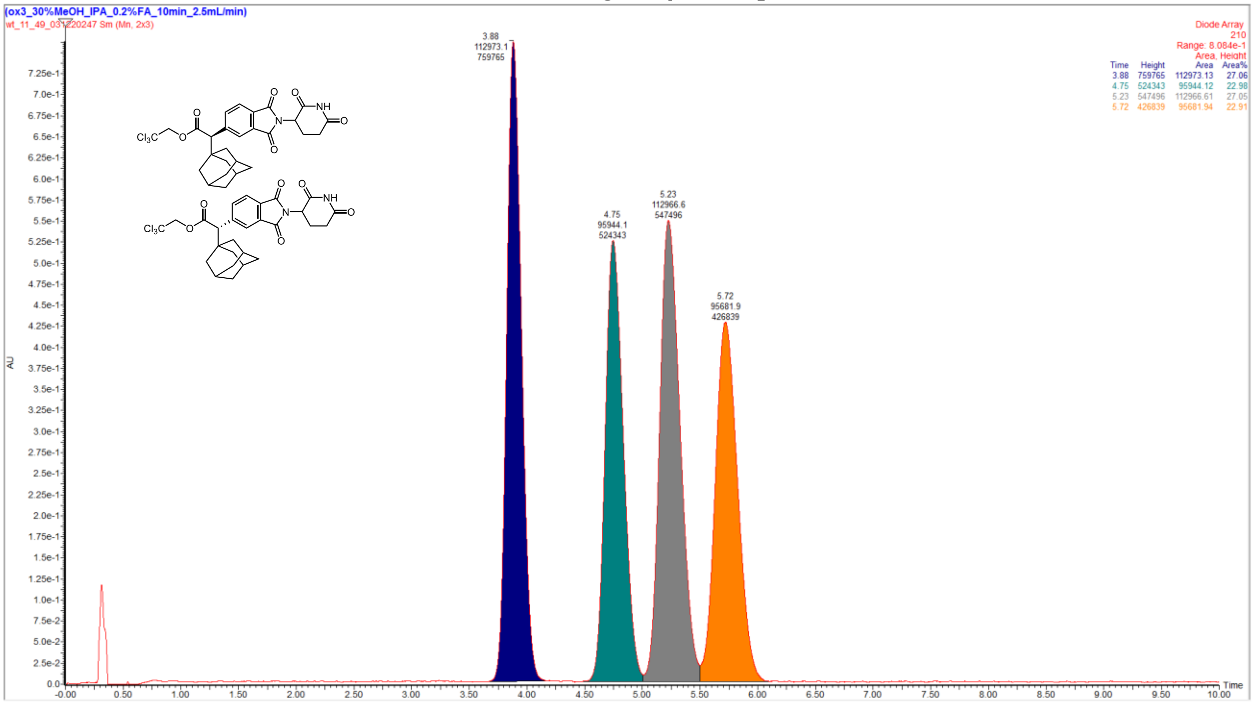

Chromatogram for Compound 17 Using GP3

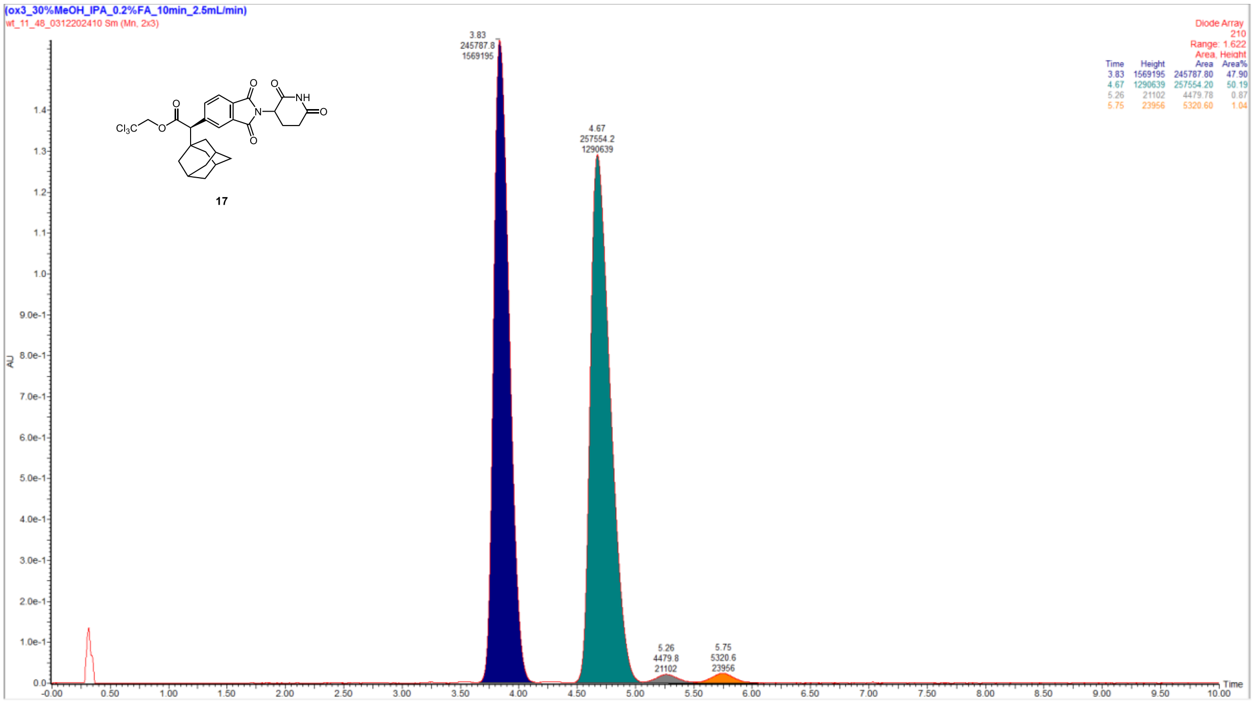

Racemic Chromatogram for Compound 18

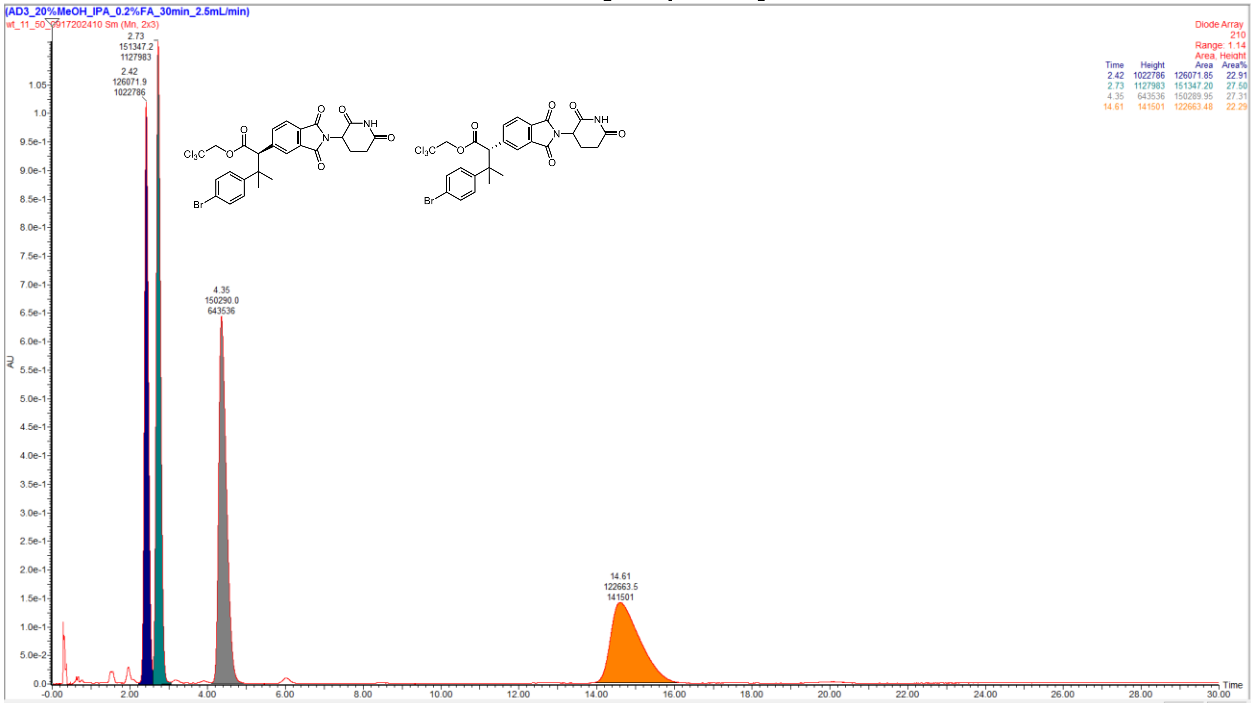

Chromatogram for Compound 18 Using GP3

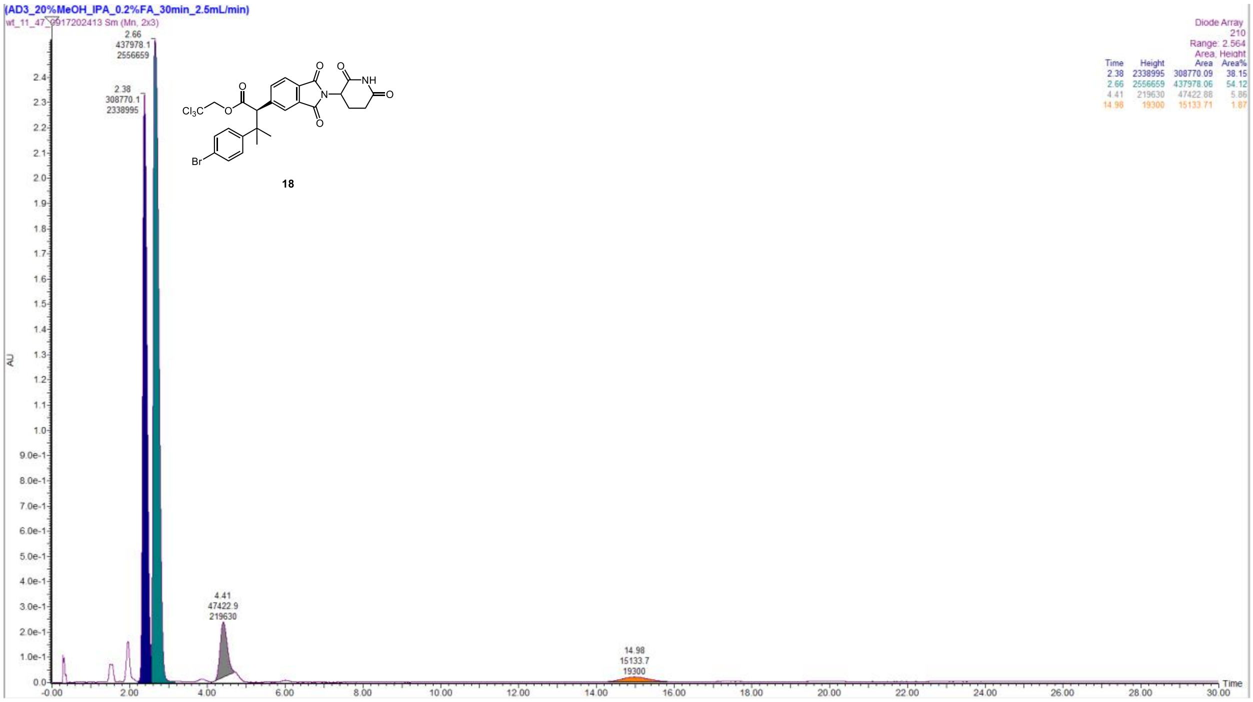

Racemic Chromatogram for Compound 19

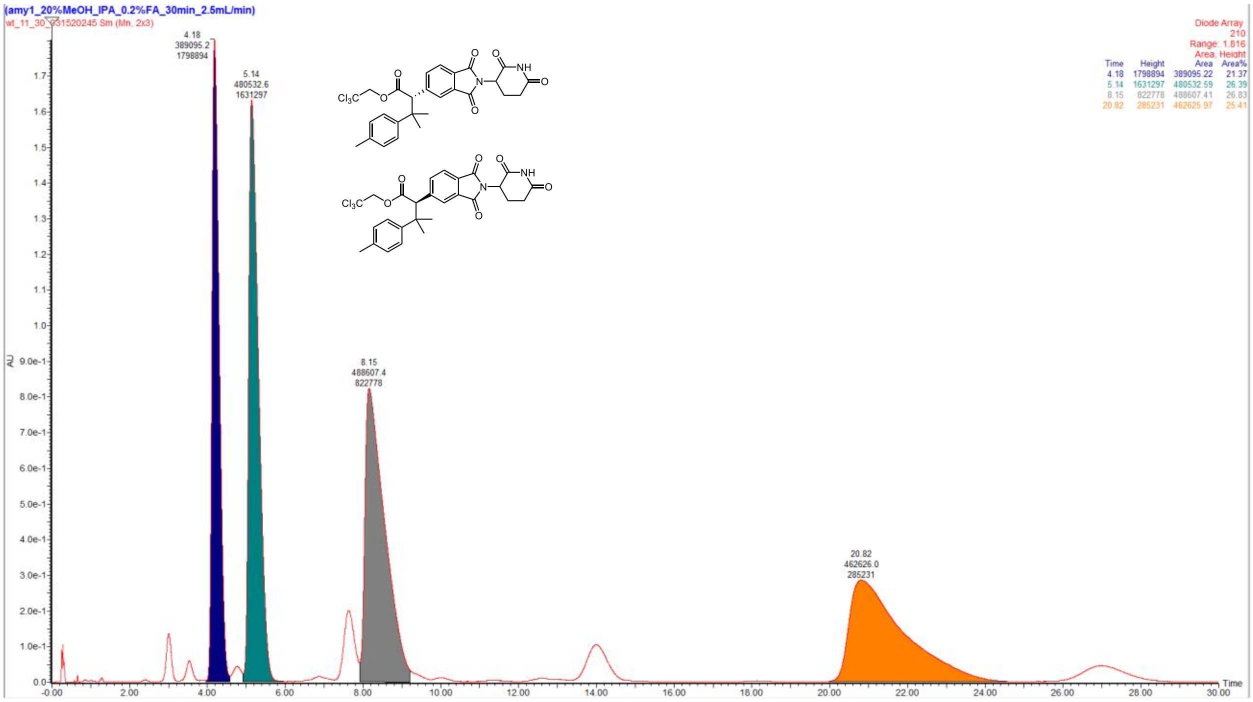

Chromatogram for Compound 19 Using GP1

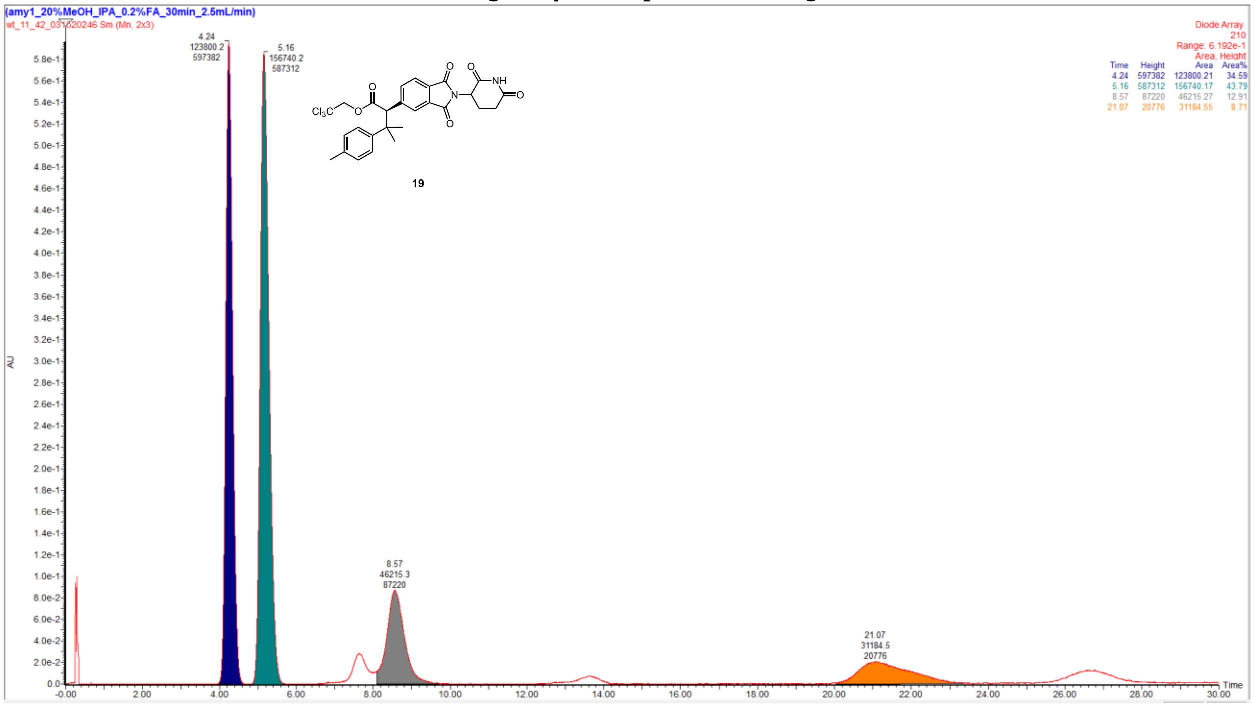

Chromatogram for Compound 19 Using GP3

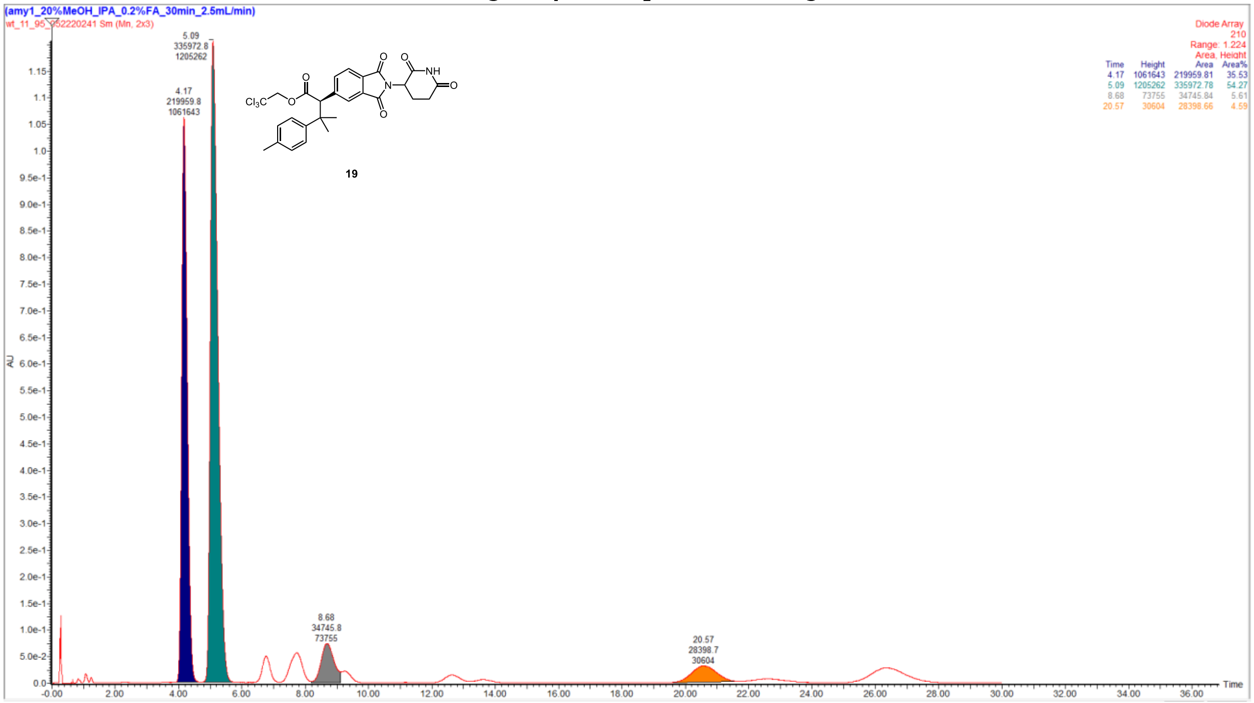

Racemic Chromatogram for Compound 20

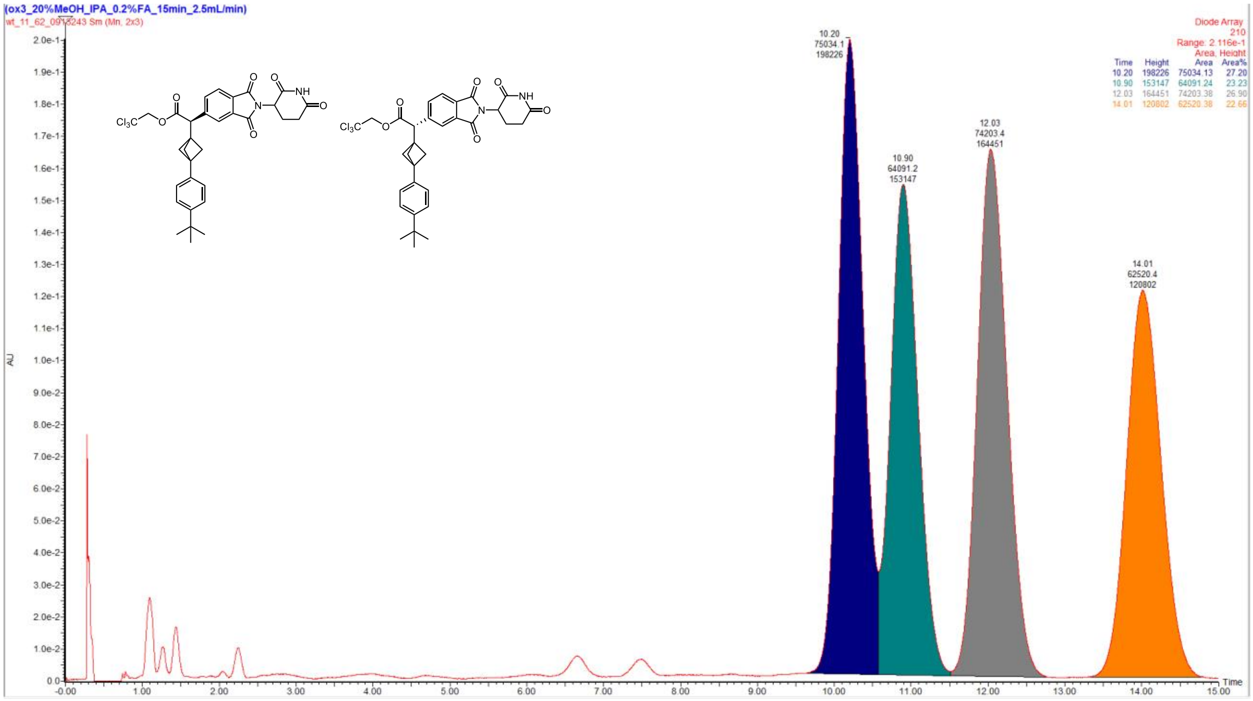

Chromatogram for Compound 20 Using GP3

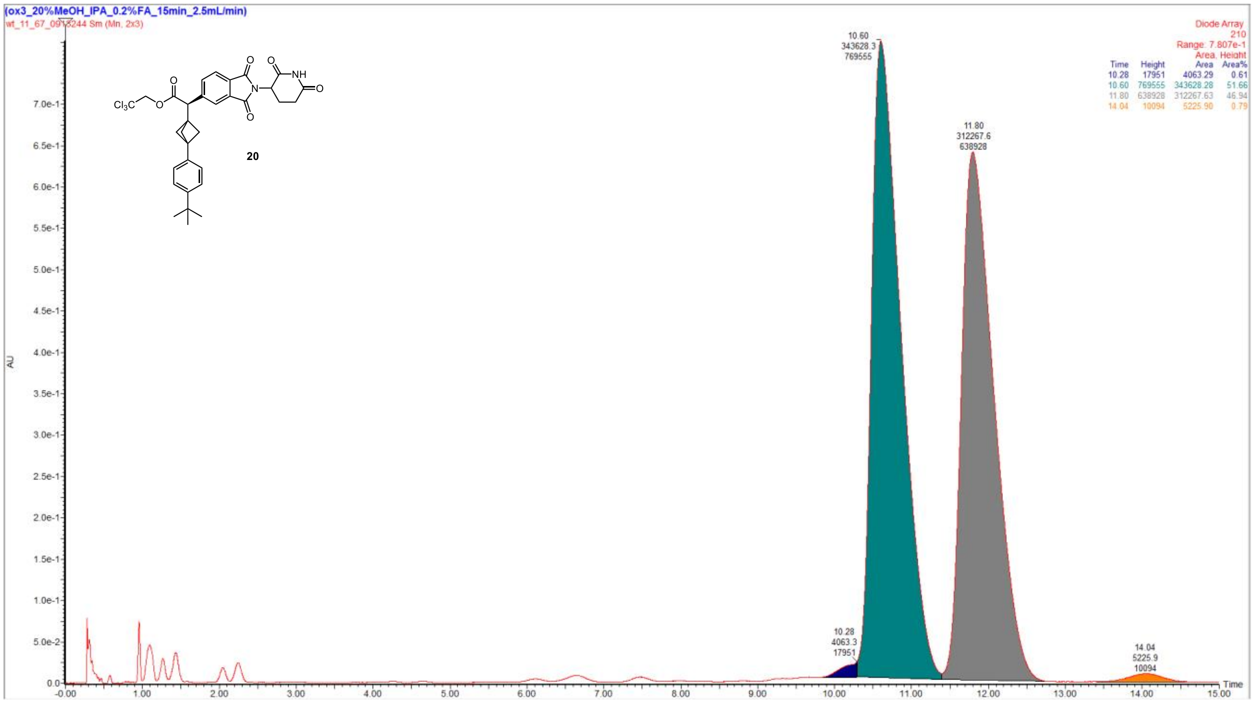

Racemic Chromatogram for Compound 21

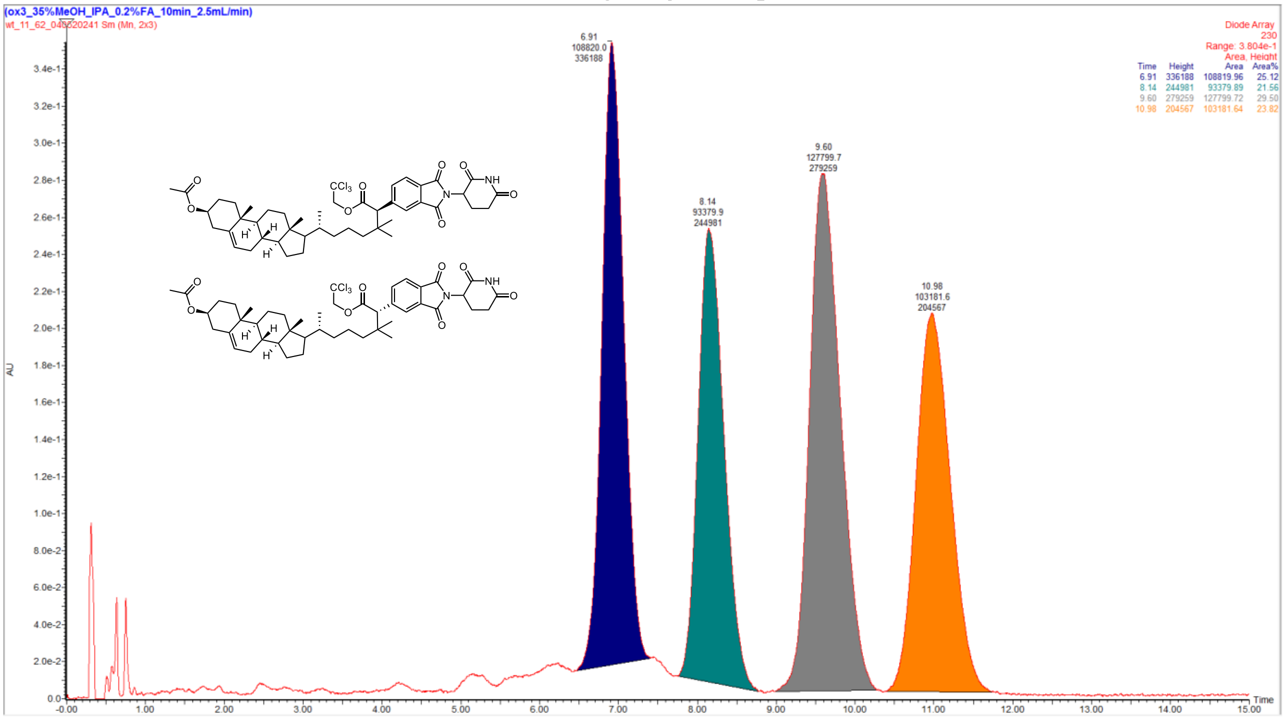

Chromatogram for Compound 21 Using GP3

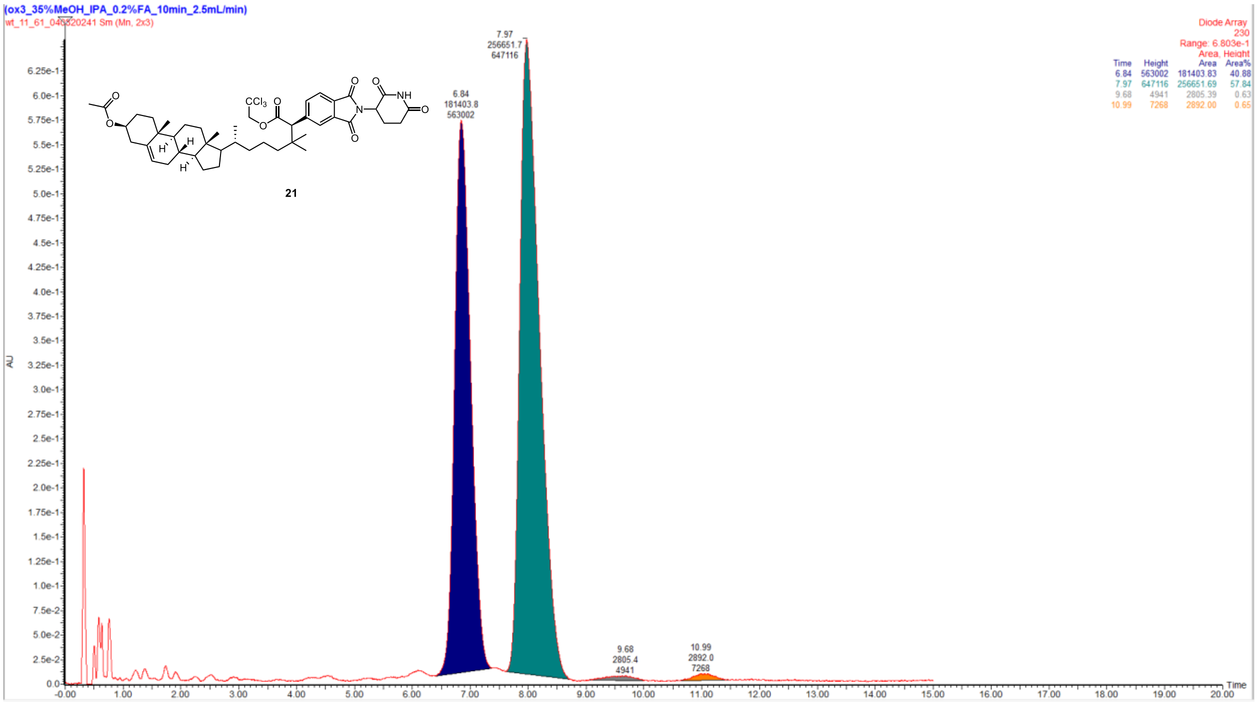

Racemic Chromatogram for Compound 22

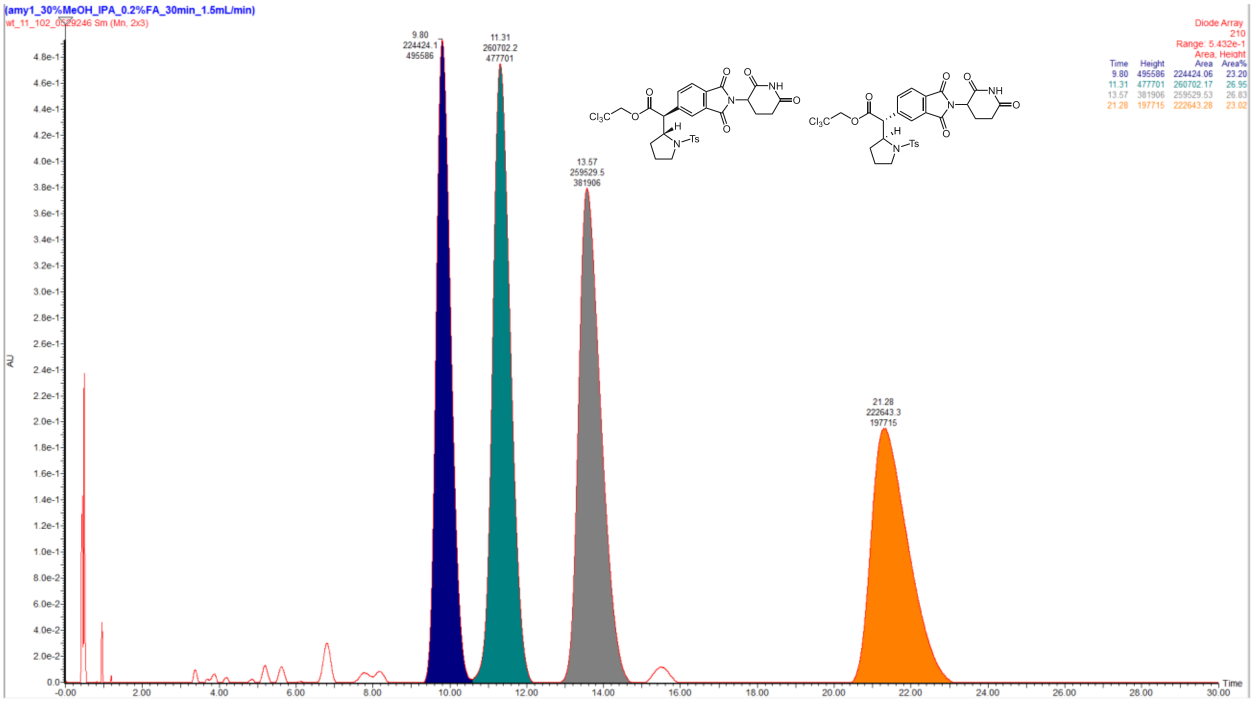

Time	Height	Area	Area%
9.80	495586	224424.06	23.20
11.31	477701	260702.17	26.95
13.57	381906	259529.53	26.83
21.28	197715	222643.28	23.02

Chromatogram for Compound 22 Using GP3

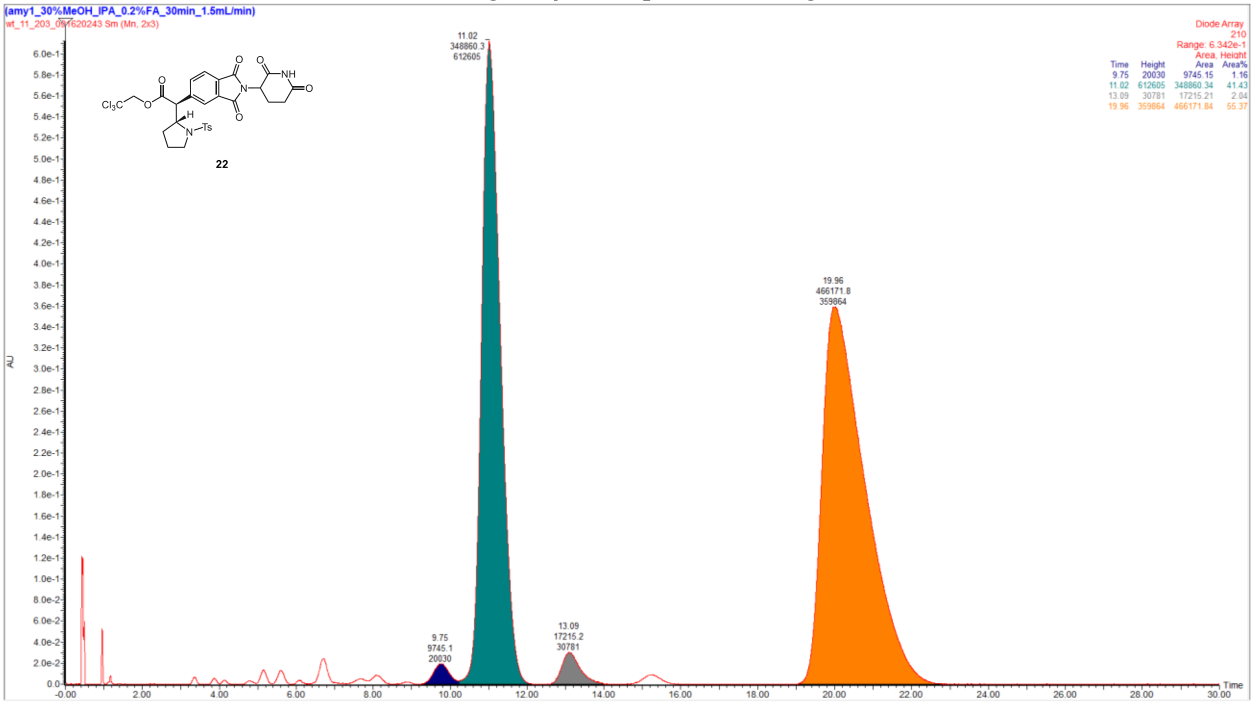

Time	Height	Area	Area%
9.75	20030	9745.15	1.16
11.02	612605	348860.34	41.43
13.09	30781	17215.21	2.04
19.96	359864	466171.84	55.37

Chromatogram for Compound 22 Using GP8

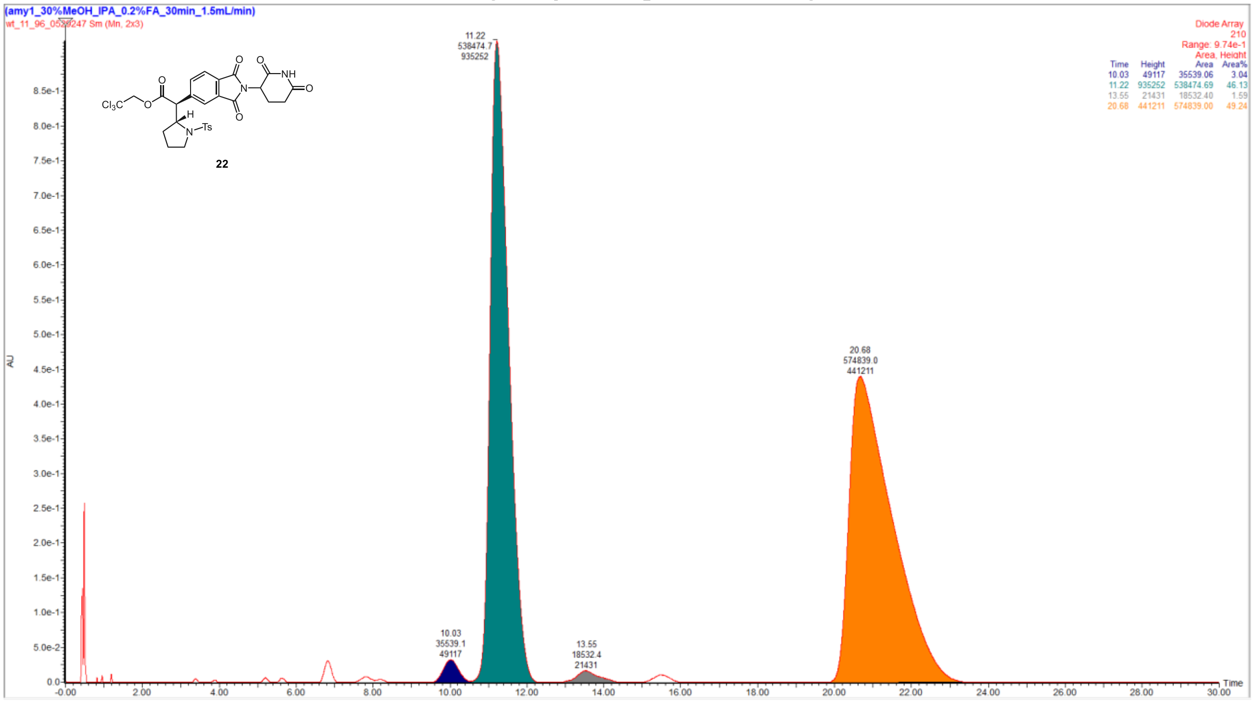

Racemic Chromatogram for Compound 24

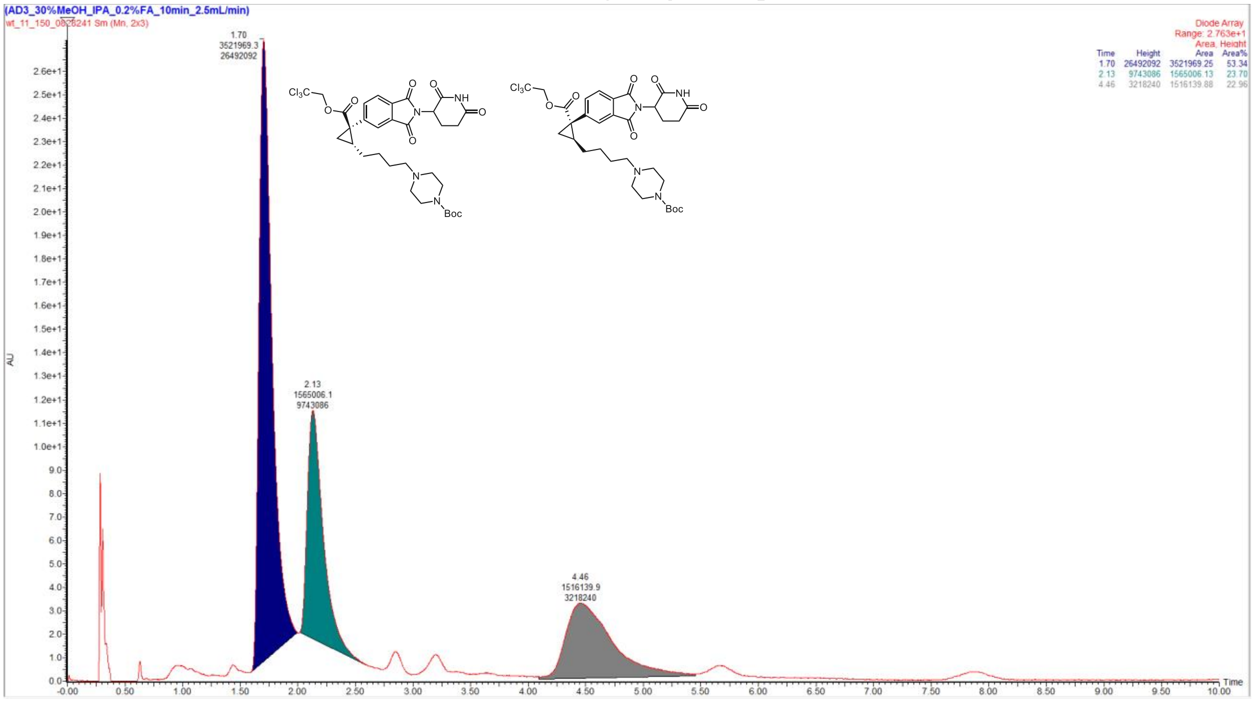

Chromatogram for Compound 24 Using GP5

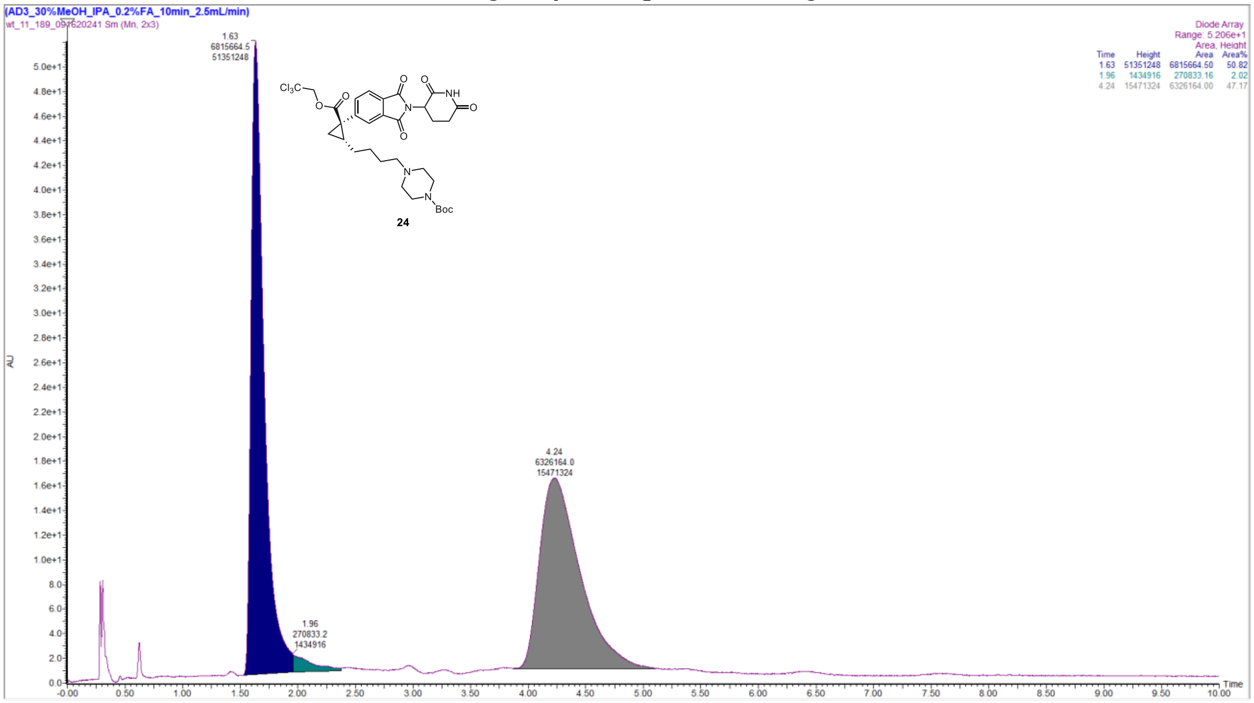

Chromatogram for Compound 24 Using GP8

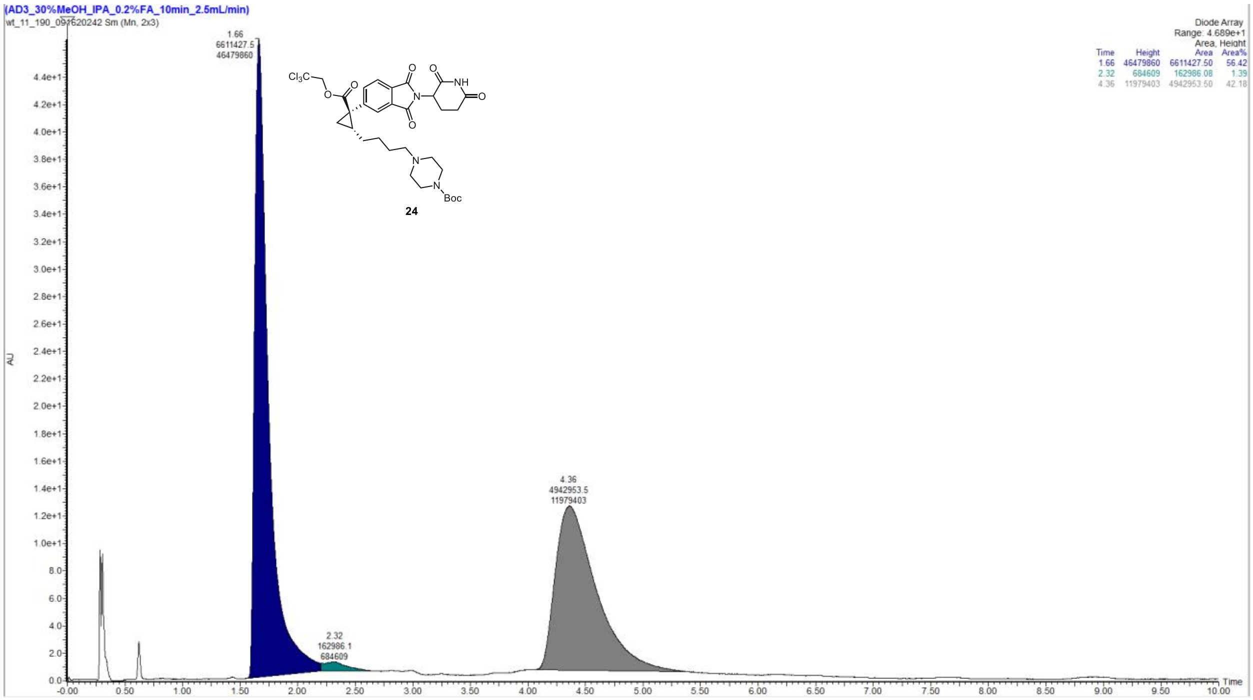

Chromatogram for Compound SI2 Using GP7

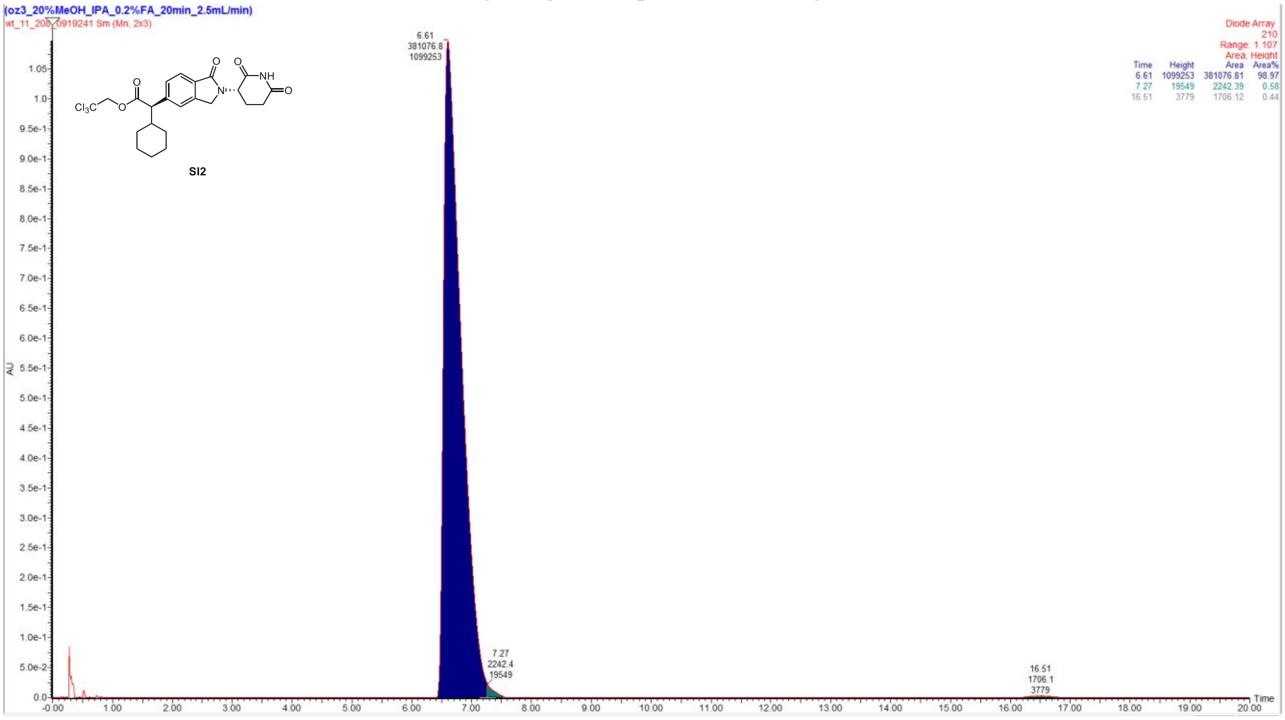

Racemic Chromatogram for Compound SI3

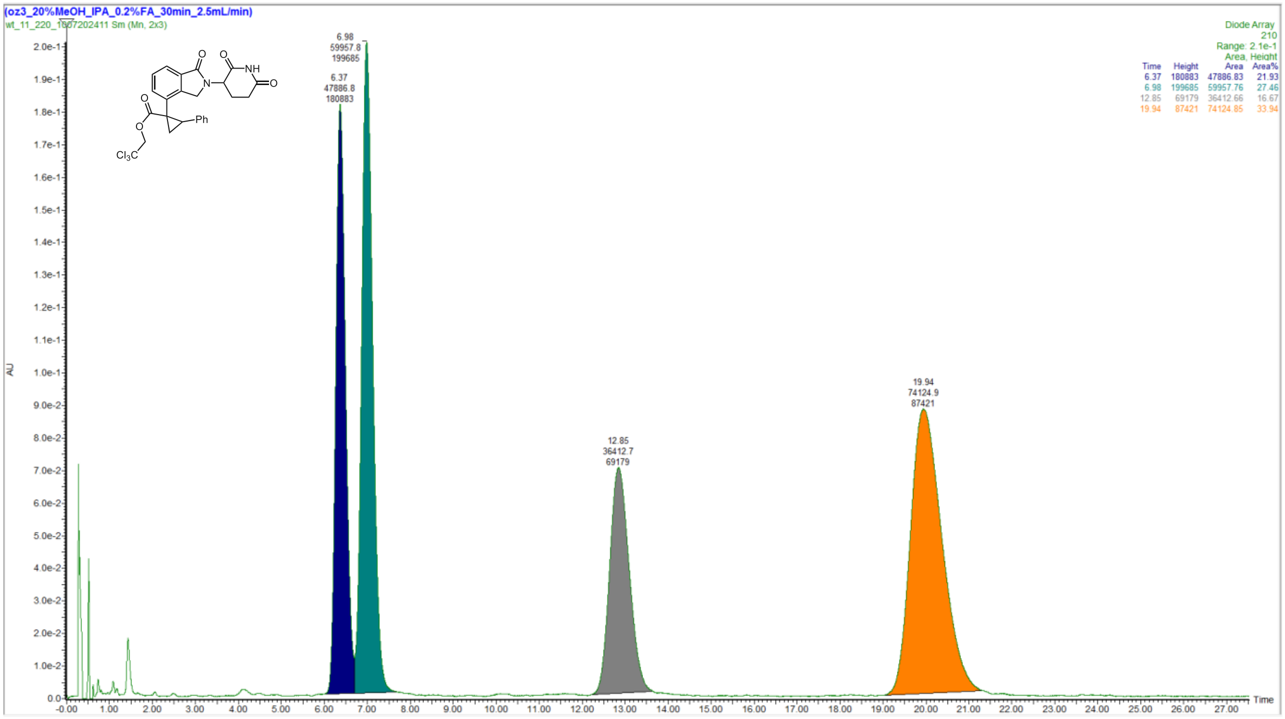

Chromatogram for Compound SI3 Using GP7

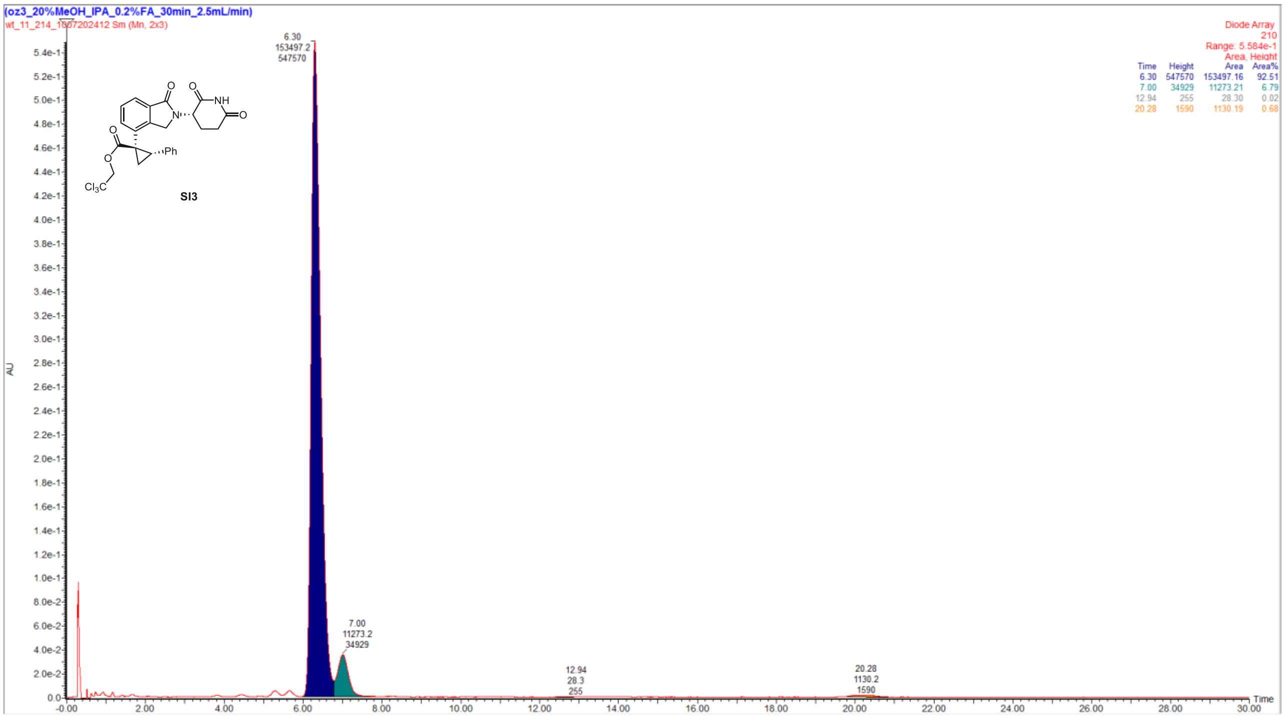

Chromatogram for Compound SI4 Using GP7

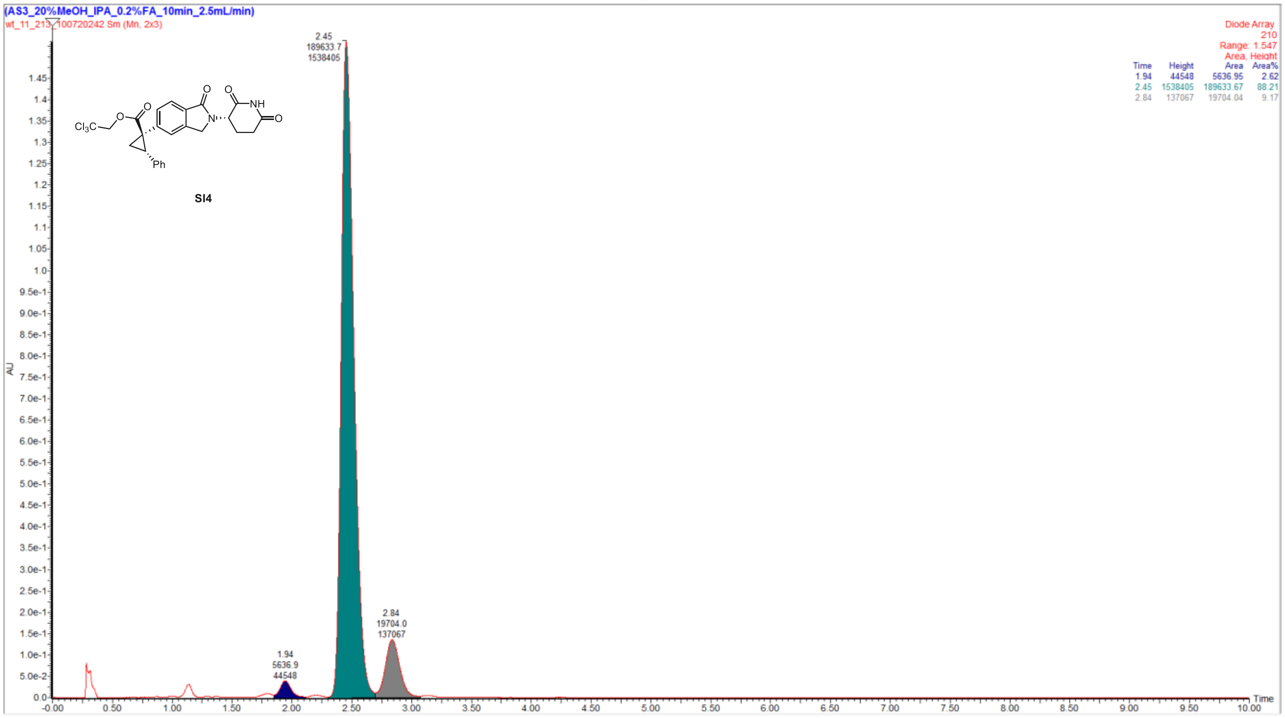

Section 6: Assay Protocols

The A549-BRD4-HiBiT cell line, created via CRISPR/Cas9, is used for detecting intracellular BRD4 protein degradation through a chemiluminescent signal. This cell line is based on the human A549 cell

line (CCL-185) which co-expresses chimeric BRD4 (the target) and HiBiT (detection tag) designed by Promaga. Routine passaging and assays are performed using RPMI 1640 medium with 10% heat-inactivated fetal bovine serum and 1X penicillin/streptomycin. Test compound detection is carried out on a 1536-well tissue culture plate (Corning #3727), with cells seeded in 3.5 µL of core-RPMI 1640 at a density of 0.20 million/mL. Pre-titrated test compounds in DMSO are dispensed onto the assay plate using the ECHO liquid handling system (Labcyte), which employs acoustic energy. The assay is incubated for 24 hours at 37°C with 5% CO2, followed by 30 minutes of plate cooling at room temperature. The detection reagent mix (Cat# N3050, Nano-Glo® HiBiT Lytic Detection System from Promega) is then added at 3.5 µL per well in a predefined proportion. Luminescence is measured using an EnVision reader (PerkinElmer) 60 minutes after the addition of the detection reagent mix. Raw data are collected and processed using Dotmatics (a data analysis tool) to determine the test compound's potency values, including EC_{50} and Y_{min}.

Section 7: References

(1) Garlets, Z. J.; Sanders, J. N.; Malik, H.; Gampe, C.; Houk, K. N.; Davies, H. M. L., Enantioselective C–H functionalization of bicyclo[1.1.1]pentanes. *Nature Catalysis* **2020,** *3*, 351-357.
(2) Tortoreto, C.; Rackl, D.; Davies, H. M. L., Metal-Free C–H Functionalization of Alkanes by Aryldiazoacetates. *Org. Lett.* **2017,** *19*, 770-773.
(3) Fu, L.; Mighion, J. D.; Voight, E. A.; Davies, H. M. L., Synthesis of 2,2,2,-Trichloroethyl Aryl- and Vinyldiazoacetates by Palladium-Catalyzed Cross-Coupling. *Chem. Eur. J.* **2017,** *23*, 3272-3275.
(4) Qin, C.; Davies, H. M. L., Role of Sterically Demanding Chiral Dirhodium Catalysts in Site-Selective C–H Functionalization of Activated Primary C–H Bonds. *J. Am. Chem. Soc.* **2014,** *136*, 9792-9796.
(5) Garlets, Z. J.; Boni, Y. T.; Sharland, J. C.; Kirby, R. P.; Fu, J.; Bacsa, J.; Davies, H. M. L., Design, Synthesis, and Evaluation of Extended C4–Symmetric Dirhodium Tetracarboxylate Catalysts. *ACS Catal.* **2022,** *12*, 10841-10848.
(6) Fu, J.; Ren, Z.; Bacsa, J.; Musaev, D. G.; Davies, H. M. L., Desymmetrization of cyclohexanes by site- and stereoselective C–H functionalization. *Nature* **2018,** *564*, 395-399.
(7) Green, S. P.; Wheelhouse, K. M.; Payne, A. D.; Hallett, J. P.; Miller, P. W.; Bull, J. A., Thermal Stability and Explosive Hazard Assessment of Diazo Compounds and Diazo Transfer Reagents. *Org. Process Res. Dev.* **2020,** *24*, 67-84.
(8) Frisch, M. J.; Trucks, G. W.; Schlegel, H. B.; Scuseria, G. E.; Robb, M. A.; Cheeseman, J. R.; Scalmani, G.; Barone, V.; Petersson, G. A.; Nakatsuji, H.; Li, X.; Caricato, M.; Marenich, A. V.; Bloino, J.; Janesko, B. G.; Gomperts, R.; Mennucci, B.; Hratchian, H. P.; Ortiz, J. V.; Izmaylov, A. F.; Sonnenberg, J. L.; Williams; Ding, F.; Lipparini, F.; Egidi, F.; Goings, J.; Peng, B.; Petrone, A.; Henderson, T.; Ranasinghe, D.; Zakrzewski, V. G.; Gao, J.; Rega, N.; Zheng, G.; Liang, W.; Hada, M.; Ehara, M.; Toyota, K.; Fukuda, R.; Hasegawa, J.; Ishida, M.; Nakajima, T.; Honda, Y.; Kitao, O.; Nakai, H.; Vreven, T.; Throssell, K.; Montgomery Jr., J. A.; Peralta, J. E.; Ogliaro, F.; Bearpark, M. J.; Heyd, J. J.; Brothers, E. N.; Kudin, K. N.; Staroverov, V. N.; Keith, T. A.; Kobayashi, R.; Normand, J.; Raghavachari, K.; Rendell, A. P.; Burant, J. C.; Iyengar, S. S.; Tomasi, J.; Cossi, M.; Millam, J. M.; Klene, M.;

Adamo, C.; Cammi, R.; Ochterski, J. W.; Martin, R. L.; Morokuma, K.; Farkas, O.; Foresman, J. B.; Fox, D. J., Gaussian 16 Rev. C.01. **2016**.
(9) Humphrey, W.; Dalke, A.; Schulten, K., VMD: Visual molecular dynamics. *Journal of Molecular Graphics* **1996,** *14*, 33-38.
(10) Momma, K.; Izumi, F., VESTA: a three-dimensional visualization system for electronic and structural analysis. *J. Appl. Crystallogr.* **2008,** *41*, 653-658.
(11) (a) Lee, C.; Yang, W.; Parr, R. G., Development of the Colle-Salvetti correlation-energy formula into a functional of the electron density. *Physical Review B* **1988,** *37*, 785-789; (b) Becke, A. D., Density-functional thermochemistry. III. The role of exact exchange. *The Journal of Chemical Physics* **1993,** *98*, 5648-5652; (c) Becke, A. D., A new mixing of Hartree–Fock and local density-functional theories. *The Journal of Chemical Physics* **1993,** *98*, 1372-1377; (d) Grimme, S.; Antony, J.; Ehrlich, S.; Krieg, H., A consistent and accurate ab initio parametrization of density functional dispersion correction (DFT-D) for the 94 elements H-Pu. *The Journal of Chemical Physics* **2010,** *132*, 154104; (e) Grimme, S.; Hansen, A.; Brandenburg, J. G.; Bannwarth, C., Dispersion-Corrected Mean-Field Electronic Structure Methods. *Chem. Rev.* **2016,** *116*, 5105-5154; (f) Johnson, E. R.; Becke, A. D., A post-Hartree-Fock model of intermolecular interactions: Inclusion of higher-order corrections. *The Journal of Chemical Physics* **2006,** *124*, 174104.
(12) (a) Hay, P. J.; Wadt, W. R., Ab initio effective core potentials for molecular calculations. Potentials for K to Au including the outermost core orbitals. *The Journal of Chemical Physics* **1985,** *82*, 299-310; (b) Roy, L. E.; Hay, P. J.; Martin, R. L., Revised Basis Sets for the LANL Effective Core Potentials. *Journal of Chemical Theory and Computation* **2008,** *4*, 1029-1031.
(13) (a) Hariharan, P. C.; Pople, J. A., The influence of polarization functions on molecular orbital hydrogenation energies. *Theoretica chimica acta* **1973,** *28*, 213-222; (b) Hehre, W. J.; Ditchfield, R.; Pople, J. A., Self—Consistent Molecular Orbital Methods. XII. Further Extensions of Gaussian—Type Basis Sets for Use in Molecular Orbital Studies of Organic Molecules. *The Journal of Chemical Physics* **1972,** *56*, 2257-2261.
(14) (a) Vreven, T.; Byun, K. S.; Komáromi, I.; Dapprich, S.; Montgomery, J. A., Jr.; Morokuma, K.; Frisch, M. J., Combining Quantum Mechanics Methods with Molecular Mechanics Methods in ONIOM. *Journal of Chemical Theory and Computation* **2006,** *2*, 815-826; (b) Dapprich, S.; Komáromi, I.; Byun, K. S.; Morokuma, K.; Frisch, M. J., A new ONIOM implementation in Gaussian98. Part I. The calculation of energies, gradients, vibrational frequencies and electric field derivatives1Dedicated to Professor Keiji Morokuma in celebration of his 65th birthday.1. *Journal of Molecular Structure: THEOCHEM* **1999,** *461-462*, 1-21.
(15) Rappe, A. K.; Casewit, C. J.; Colwell, K. S.; Goddard, W. A., III; Skiff, W. M., UFF, a full periodic table force field for molecular mechanics and molecular dynamics simulations. *J. Am. Chem. Soc.* **1992,** *114*, 10024-10035.
(16) (a) Cossi, M.; Rega, N.; Scalmani, G.; Barone, V., Energies, structures, and electronic properties of molecules in solution with the C-PCM solvation model. *J. Comput. Chem.* **2003,** *24*, 669-681; (b) Barone, V.; Cossi, M., Quantum Calculation of Molecular Energies and Energy Gradients in Solution by a Conductor Solvent Model. *The Journal of Physical Chemistry A* **1998,** *102*, 1995-2001.
(17) Ren, Z.; Musaev, D. G.; Davies, H. M. L., Key Selectivity Controlling Elements in Rhodium-Catalyzed C–H Functionalization with Donor/Acceptor Carbenes. *ACS Catal.* **2022,** *12*, 13446-13456.
